# mirrorCheck: an R package facilitating informed use of DESeq2’s lfcShrink() function for differential gene expression analysis of clinical samples

**DOI:** 10.1093/bioadv/vbaf070

**Published:** 2025-04-02

**Authors:** Katherine Elise Scull, Kiarash Behrouzfar, Daniella Brasacchio, Enid Yi Ni Lam, Dineika Chandrananda, Paul Yeh

**Affiliations:** Blood Cancer Biomarkers Lab, Department of Medicine, School of Clinical Sciences at Monash Health, Monash University, Clayton, Victoria, 3168, Australia; Blood Cancer Biomarkers Lab, Department of Medicine, School of Clinical Sciences at Monash Health, Monash University, Clayton, Victoria, 3168, Australia; Blood Cancer Biomarkers Lab, Department of Medicine, School of Clinical Sciences at Monash Health, Monash University, Clayton, Victoria, 3168, Australia; Peter MacCallum Cancer Centre, Melbourne, Victoria, 3000, Australia; Sir Peter MacCallum Department of Oncology, The University of Melbourne, Parkville, Victoria, 3010, Australia; Collaborative Centre for Genomic Medicine, Faculty of Medicine, Dentistry and Health Sciences, The University of Melbourne, Parkville, Victoria, 3010, Australia; Peter MacCallum Cancer Centre, Melbourne, Victoria, 3000, Australia; Sir Peter MacCallum Department of Oncology, The University of Melbourne, Parkville, Victoria, 3010, Australia; Collaborative Centre for Genomic Medicine, Faculty of Medicine, Dentistry and Health Sciences, The University of Melbourne, Parkville, Victoria, 3010, Australia; Department of Medicine, School of Clinical Sciences at Monash Health, Monash University, Clayton, Victoria, 3168, Australia; Blood Cancer Biomarkers Lab, Department of Medicine, School of Clinical Sciences at Monash Health, Monash University, Clayton, Victoria, 3168, Australia; Monash Haematology, Clayton, Victoria, 3168, Australia

## Abstract

**Motivation:**

The sophisticated lfcShrink() function implemented in the DESeq2 package for differential gene expression analysis aims to reduce noise from low read count and/or highly variable genes in bulk RNA-sequencing data, thus circumventing the need for arbitrary filtering thresholds. However, difficulties can arise when analysing clinical data with multiple biologically-relevant groupings. In particular, changing the reference group can dramatically alter the ranking of differentially expressed genes, instead of merely ‘mirroring’ the up- and down-regulated genes in reciprocal comparisons.

**Results:**

Here, we present mirrorCheck, an R package to automate methodical lfcShrink() usage and data visualization for quality control and data-driven decision-making during analysis.

**Availability and implementation:**

The source code, including documentation, is available on github at https://github.com/kescull/mirrorCheck.

## 1 Introduction

In differential gene expression analysis (DGEA), differences for certain genes may be statistically significant yet too small to denote a biologically significant finding (Love *et al.* 2014). Therefore, biologists often rank differentially expressed genes (DEGs) for further investigation by the logarithmic fold change (LFC). However, among lowly expressed or highly variable genes, LFCs are noisy and biased towards exaggerated values (Zhu *et al.* 2019). To avoid inappropriate rankings, workflows prefilter low-count genes, and/or shrink the LFC in various ways, to generate more accurate LFC estimates (Law *et al.* 2014, Love *et al.* 2014, Chen *et al.* 2016, Stephens 2017). DESeq2, one of the most common methods, originally incorporated LFC shrinkage estimation by default (Love *et al.* 2014); DESeq2 v1.16 introduced the lfcShrink() function, granting users control over whether they employ LFC shrinkage and with which algorithm (Love *et al.* 2024). lfcShrink() with the ‘apeglm’ algorithm represents a powerful method for appropriately ranking genes (Zhu *et al.* 2019); however, we encountered difficulties with multi-group clinical RNA-Seq data.

Using ‘apeglm’, users can only extract results for comparisons (‘contrasts’) including the reference group. With multiple groups, users may need to change the reference group and reanalyse to extract all desired results. To avoid errors, we automated this process via run_all_DESeq_contrasts(), which extracts results for all possible contrasts. Unexpectedly, volcano plots from reciprocal contrasts (e.g. Group1/Group2 and Group2/Group1) did not mirror each other, thus failing a ‘mirror-check’ and raising doubts about the reliability of DEGs.

Although ‘apeglm’ was intended to make prefiltering unnecessary (Zhu *et al.* 2019), we observed that multi-group data may require prefiltering to stabilize the shrinkage estimation, as discussed (Love 2021). This would be especially important in highly variable patient data. For such scenarios, we implemented the compare_reciprocal_contrasts() function to interrogate and visualize results from lfcShrink().

This short report aims to alert new DESeq2 users to the ‘mirror-check’ issue and present our mirrorCheck R package as an accessible quality control tool. mirrorCheck helps users run lfcShrink() and determine whether prefiltering or other types of data cleaning is required, particularly for multi-group clinical data.

## 2 Methods

Supplementary Files S1, S3, S5, and S9 contain full R codes for the analysis and visualization reported in this paper.

*COVID*, *BRCA*, and *Cell line* datasets were sourced as described in Data Availability. For *BRCA*, TCGABiolinks (Colaprico *et al.* 2016, Silva *et al.* 2016, Mounir *et al.* 2019) was used to download transcriptomic data analysed using the ‘STAR—Counts’ workflow; samples with unknown PAM50 subtype (Berger *et al.* 2018) were discarded, except for ‘Solid Tissue Normal’ samples retained as a healthy control. Counts tables and metadata were imported into DESeqDataSet or DGEList objects for analysis with DESeq2 v.1.44 or edgeR v4.2, respectively. Genes with adjusted *P*-value <.01 and absolute LFC >2 were considered DEGs. For prefiltering, edgeR’s filterByExpr() was used with default parameters. sva v3.52.0 (Leek *et al.* 2024) was used as described in Love *et al.* 2016). For GSEA, genes were ranked by LFC and GSEAPreranked was run using the cli script packaged with GSEA Desktop for Linux v. 4.3.3 with the following non-default parameters: Gene set database = h.all.v2024.1.Hs.symbols.gmt, 10 000 permutations, No Collapse (Supplementary Files S1 and S2 include the full command). Statistical testing was performed using ggpubr: global *P*-values and pairwise comparisons were calculated using Kruskal–Wallis and Wilcoxon tests, respectively.

### 2.1 mirrorCheck

mirrorCheck includes full documentation (Scull 2024). Briefly, the function run_all_DESeq_contrasts() requires a DESeqDataSet and design factor as input. By default, it uses lfcShrink() with the ‘apeglm’ algorithm; users can specify the ‘ashr’ or ‘normal’ lfcShrink() algorithm with the mode parameter, or use mode=‘none’ to run with results() instead of lfcShrink(). However, the package was designed to facilitate use of ‘apeglm’, as this is the only algorithm which requires users to change the reference level in order to extract all comparisons between multiple groups. run_all_DESeq_contrasts() outputs MA and volcano plots, and DEG lists and heatmaps, according to user-specified adjusted *P*-value and LFC thresholds. With the print.all parameter, it will additionally output the full gene lists without applying thresholds. compare_reciprocal_contrasts() requires DEG lists as produced by run_all_DESeq_contrasts(). It outputs Venn diagrams and concordant DEG lists for each reciprocal contrast, and diagnostic plots (stacked bar charts, and also density plots of the sum of LFCs for concordant DEGs, showing the deviation from 0 per contrast). Finally, an UpSet plot shows the overlap in DEGs from different comparisons, and the intersections are detailed in a csv, which may be of interest for further analysis. Supplementary Files S7 and S8 provide a tutorial-style example of how to use mirrorCheck, which uses a Bioconductor dataset for enhanced accessibility.

## 3 Results and discussion

**Figure 1.**
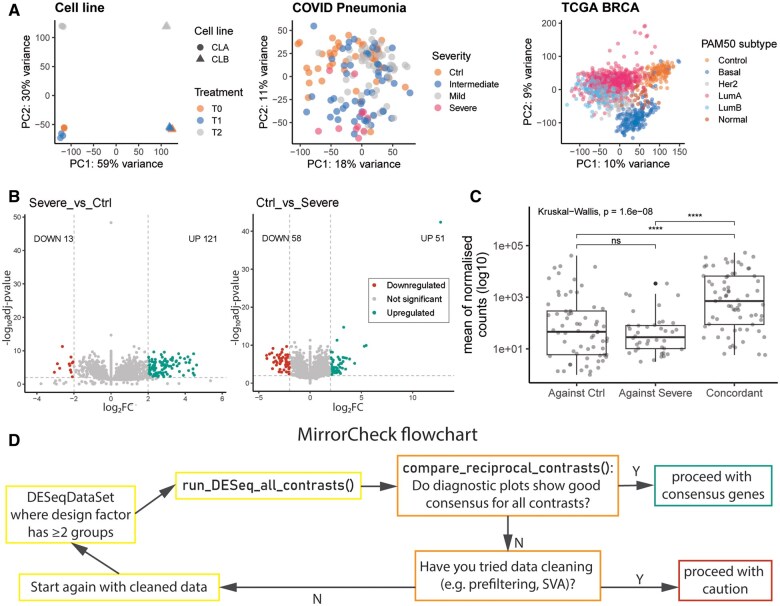
Public datasets illustrate how contrasts analysed with lfcShrink() can fail a ‘mirror-check’. (A) Principal component analysis was performed on transformed counts data using DESeq2’s vst() and plotPCA() functions. (B and C) Data for one reciprocal contrast (Severe-Ctrl) from *COVID*, analysed using lfcShrink() function with the ‘apeglm’ algorithm. Volcano plots in (B) are annotated with the number of up- and down-regulated differentially expressed genes (DEGs), which are marked in colour (adjusted *P*-value <.01, absolute value of LFC >2). The reference level was the Ctrl group in the lefthand plot and the Severe group in the righthand plot. (C) The relative expression levels of all DEGs from this contrast using the baseMean for each gene (i.e. the mean of normalized counts for the gene across all samples in the dataset) with a log10 transformation. Genes are categorized based on whether they were found to be DEGs only using a particular reference level (‘Against Ctrl’ or ‘Against Severe’) or both ways (‘Concordant’). Statistical testing was performed using ggpubr, with the global *P*-value calculated using a Kruskal–Wallis test while pairwise comparisons use Wilcoxon tests (ns: *P* > .05, ****: *P* ≤ .0001). (D) Proposed workflow for using mirrorCheck to perform DESeq2 differential gene expression analysis and to check whether lfcShrink() is suitable with or without data cleaning steps for a given dataset.

To demonstrate the ‘mirror-check’ issue, and how mirrorCheck can help monitor lfcShrink() performance, we sourced three publicly available datasets with multiple biologically-relevant groupings. We selected datasets which demonstrate a range of scenarios, from highly reproducible *in vitro* datasets to inherently variable clinical cohorts. The *Cell line* dataset includes data from two cell lines treated with one of two treatments or a control (totalling six groups) (Corchete *et al.* 2020). The *BRCA* dataset contains clinical data from the TCGA-BRCA project (Koboldt *et al.* 2012, Ciriello *et al.* 2015) filtered for tumour samples annotated with a PAM50 subtype (Berger *et al.* 2018), thus defining six groups including a control, with a large number of samples per group. The third study categorized COVID patients by the severity of their pneumonia (*COVID*, four groups) (Armignacco *et al.* 2024). We used principal component analysis to visualize the datasets, revealing a broad range of inter- and intra-group variability among the datasets (Fig. 1A). As expected, *Cell line* groups clustered tightly and the first principal component (PC1) captured most of the variance (59% variance), whereas the clinical datasets showed much greater variability (*COVID* PC1: 18% variance; *BRCA* PC1: 10% variance); *COVID* groups also clustered poorly, while *BRCA* had more distinct clusters.

We performed DGEA on each dataset using DESeq2 with the ‘apeglm’ algorithm for lfcShrink(), via mirrorCheck’s run_all_DESeq_contrasts() (Supplementary Files S1–S6). This function sets each group as the reference in turn and recursively extracts results for all possible contrasts, including reciprocal contrasts. Showing the Severe-Ctrl contrast from *COVID*, which will serve as an example throughout, Fig. 1B demonstrates the eponymous issue: these volcano plots fail to mirror each other vertically as expected. That is, we expect the genes that are up-regulated when using one group as the reference to be down-regulated when using the other group as the reference, but the numbers of DEGs annotated in Fig. 1B do not match (here, DEGs were defined as having adjusted *P*-value <.01, absolute value of LFC >2). To investigate further, we labelled DEGs ‘concordant’ if observed in both reciprocal contrasts, or ‘discordant’ if observed only with a particular reference. Plotting the mean of normalized counts for each DEG (Fig. 1C), we found that discordant DEGs exhibited significantly lower average expression than concordant DEGs, as expected if sparse counts exacerbate the discrepancy (Supplementary File S2 includes similar results for another *COVID* contrast). We present the mirrorCheck workflow (Fig. 1D) to quickly identify unreliable LFC shrinkage and the need for data cleaning, such as prefiltering of lowly expressed genes in multi-group data (Love 2021). Namely, run_DESeq_all_contrasts() straightforwardly produces volcano and MA plots, DEG lists and heatmaps for all contrasts. Users may then input the DEG lists into mirrorCheck’s diagnostic tool, compare_reciprocal_contrasts(). This function is a quality control tool, allowing users to rapidly evaluate concordance for each contrast, and outputs concordant DEG lists. We advise proceeding with the concordant DEGs (defined according to user-specified LFC and adjusted *P*-value thresholds), which indicate stable shrinkage estimation. When large discrepancies occur in one or more contrasts, users should appraise prefiltering and/or other appropriate data cleaning approaches, as we demonstrate below. In addition to the three datasets analysed here, Supplementary Files S7 and S8 demonstrate the use of mirrorCheck with the built-in Bioconductor ‘parathyroidSE’ dataset (Haglund *et al.* 2012), which readers can easily access to trial mirrorCheck for themselves.

**Figure 2.**
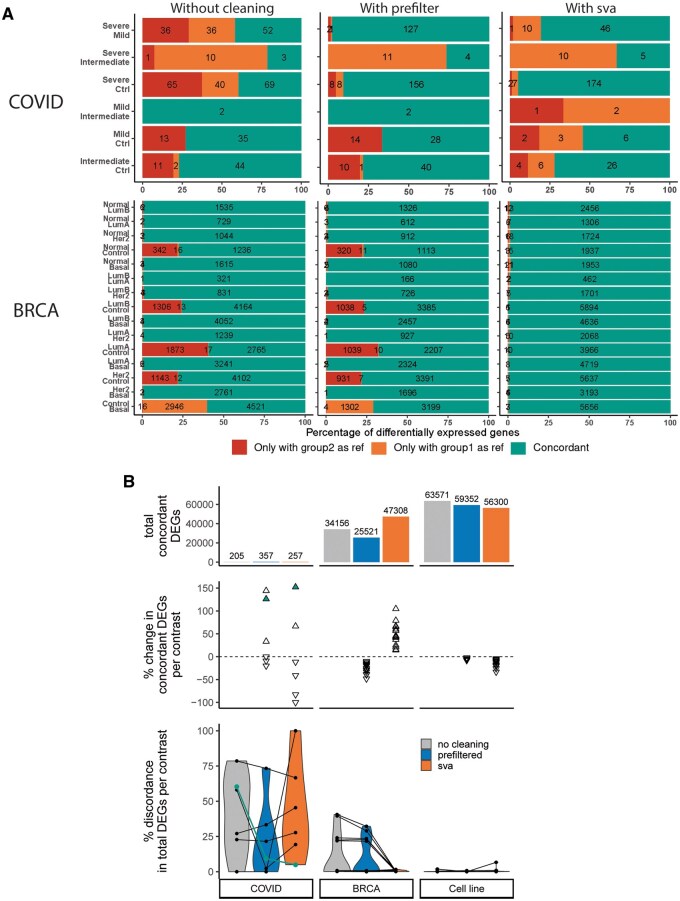
mirrorCheck reveals scenarios when data cleaning increases the number and/or proportion of concordant differentially expressed genes (DEGs) in reciprocal contrasts. DEGs were defined by adjusted *P*-value <.01, absolute value of LFC > 2. (A) mirrorCheck diagnostic plots for the *COVID* and *BRCA* datasets using different data cleaning: stacked bar charts show the degree of concordance for each pairwise comparison in green (the rightmost section of the bar), while DEGs found only when using a particular group as the reference level are shown in red/orange. Results without any data cleaning, after prefiltering, or using surrogate variable analysis (SVA) are shown on the left, centre, and right, respectively. (B) Plots summarizing results of mirrorCheck analysis across the three datasets. Bottom: The percentage of discordance between reciprocal contrasts (100 − (concordant DEGs/total × 100)) is plotted with a line connecting data from the same comparison without data cleaning, with prefiltering or with SVA, overlaid on violin plots. Middle: Percentage change in the number of concordant DEGs after data cleaning observed for each comparison (([prefiltered or SVA] − no cleaning)/no cleaning × 100). Top: Bar plot of summed concordant DEGs from all comparisons in each dataset, without data cleaning or with prefiltering or SVA. Data from the Severe-Ctrl contrast is highlighted in green.

Figure 2A showcases mirrorCheck’s stacked bar chart diagnostic plot, which immediately allows users to identify contrasts compromised by discordance. This demonstrates how mirrorCheck can help users determine whether their dataset requires a cleaning strategy and if so, which steps are most appropriate. Firstly, the lefthand and centre plots in Fig. 2A show that prefiltering greatly improved concordance in two of three affected contrasts in the *COVID* dataset. However, the corresponding plots for *BRCA* (Fig. 2A, bottom row, Supplementary Files S3 and S4) do not reveal a similar improvement after prefiltering. Since the *BRCA* dataset involves large numbers of samples, we next considered a data cleaning strategy aimed at controlling for unwanted variation, namely surrogate variable analysis (SVA). SVA is designed to identify hidden sources of variation, such as batch effects and other technical artefacts, in high-throughput experiments (Leek *et al.* 2012). Users can add the surrogate variables to the design formula before performing DGEA with DESeq (Love *et al.* 2016), which can reduce bias and spurious results (Supplementary Files S7 and S8 contain further examples and discussion regarding SVA). The *BRCA* diagnostic plots in Fig. 2A show that SVA dramatically improved concordance in the five discordant contrasts. Finally, to explore how data cleaning affects DGEA in datasets with little intra-group and more inter-group variability, we similarly analysed the *Cell line* dataset (Supplementary Files S5 and S6) and compared all the results. Fig 2B (Supplementary Files S9 and S10) shows that prefiltering *COVID* dramatically boosted both the number and proportion of concordant DEGs for two contrasts, whereas it was unclear whether SVA led to an overall benefit in this dataset. While prefiltering *BRCA* only slightly improved concordance for two contrasts at a cost of many DEGs overall, SVA eliminated discordance and increased the number of concordant DEGs. In contrast, *Cell line* displayed excellent concordance without any data cleaning; prefiltering deleted many concordant DEGs and SVA removed more DEGs while also slightly increasing discordance. Thus, with mirrorCheck’s assistance we can conclude that *COVID* benefits from prefiltering, *BRCA* requires SVA, and *Cell line* does not require data cleaning.

Lastly, we wished to confirm our assumption that concordant genes are more reliable, and that discordance will lead to less reproducible or valid results. However, we do not have the ground truth for this experimental data and also could not generate multi-group synthetic data for benchmarking with available tools. Instead, we focussed on how prefiltering affected the *COVID* analysis in two ways; firstly by comparing the results with those of another DGEA tool, and secondly by observing how discordance affected downstream analyses.

**Figure 3.**
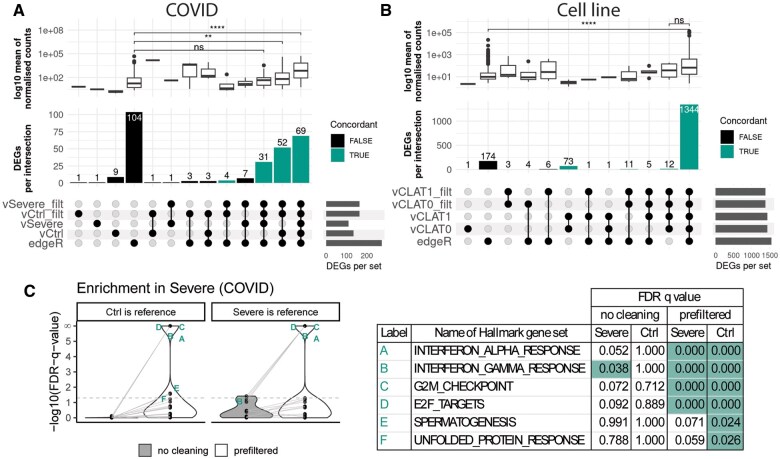
Improved mirrorCheck consensus enhances consistency with edgeR analysis and reproducibility of Gene Set Enrichment Analysis. *Data*: (A and C) Data from the Severe-Ctrl reciprocal contrasts from *COVID*. (B) Data from a *Cell line* reciprocal contrast, comparing groups where cell line A was treated with T1 or with a control (CLAT1-CLAT0). *Analysis*: (A and B) Comparisons of DEGs identified by a standard edgeR analysis with DEGs observed in reciprocal contrasts using DESeq2 with and without prefiltering. DEGs were defined by adjusted *P*-value <.01, absolute value of LFC > 2. Bottom: UpSet plots showing intersections between groups. DEGs found concordantly either with or without prefiltering are marked in green. Top: Boxplots overlaid with jittered points showing the log10 transformed baseMean for DEGs in the given UpSet intersection below. Statistical tests were performed using ggpubr (Wilcoxon; ns: *P* < .05, **: *P* ≤ .01, ****: *P* ≤ .0001). (C) Ranked gene lists were prepared from mirrorCheck’s output files based on the log2FoldChange statistic and GSEA performed using the Broad Institute’s GSEAPreranked script. On the left, violin plots shows enriched gene sets from ranked gene lists made with or without prefiltering the data, and using either group as the reference group for DGEA. Significance is defined as FDR *q*-value <.05, which is marked with a horizontal dotted line. Where the *q*-value was reported as .000, this is marked as ‘infinity’ in the log-transformed data for visualization. Significant gene sets are labelled with letters, and details given in the table on the right including the non-transformed *q*-values.

We used UpSet plots (Conway *et al.* 2017) to track how prefiltering affected lfcShrink()’s handling of individual genes, and to compare DESeq2 DEGs to those obtained by the popular DGEA tool, edgeR (Robinson *et al.* 2010, McCarthy *et al.* 2012, Chen *et al.* 2016) (Fig. 3A and B). A standard edgeR workflow including prefiltering and light shrinkage showed no discordance between reciprocal contrasts (Supplementary Files S2, S4, and S6). Due to edgeR’s lighter shrinkage, more genes were expected to meet the LFC threshold. As shown in Fig. 3A, edgeR found all 156 DESeq2 concordant DEGs for the *COVID* Severe-Ctrl comparison, whereas only half of the 26 discordant DEGs were identified by edgeR. A further 10 discordant DEGs were not observed after prefiltering. A comparatively large number of DEGs were found by edgeR but not DESeq2. For comparison, we repeated this analysis with a contrast from *Cell line*, which showed excellent concordance without prefiltering (Fig. 3B). Here, the vast majority of DEGs were observed in all sets. Approximately 10% were observed only by edgeR, while ∼5% were concordant DESeq2 DEGs but not observed by edgeR. Most of these concordant DEGs (73/88) displayed very low expression and were not found after prefiltering the data, implying that prefiltering also removed them in the edgeR analysis. This is consistent with original findings that ‘apeglm’ could identify true DEGs among low-count genes which would be filtered out in other workflows (Zhu *et al.* 2019). Interestingly, edgeR-only DEGs showed significantly lower expression in both contrasts than the prefiltered DESeq2 concordant DEGs (Fig. 3A and B), suggesting that DESeq2 also excludes some genes which narrowly meet the low-count filter threshold. Although discordant DEGs also exhibited relatively low gene expression (Fig. 3A and C), prefiltering made many of these genes concordant, rather than removing them. These results demonstrate how sparse data can negatively impact ‘apeglm’ shrinkage estimation across all genes, not just those with low counts. In summary, prefiltering reduced discordance in the *COVID* contrast, largely by shifting individual genes from the ‘discordant’ to ‘concordant’ category. This also made the results more consistent with those from edgeR, suggesting that the affected genes were valid DEGs.

To demonstrate the impact of discordance on downstream analyses, we performed Gene Set Enrichment Analysis (GSEA) on the *COVID* Severe-Ctrl comparison with or without prefiltering. We exported the whole gene list following DGEA using mirrorCheck’s ‘print.all’ parameter, and used the LFC to rank them prior to analysis with the UC San Diego and Broad Institute’s GSEAPreranked algorithm (Mootha *et al.* 2003, Subramanian *et al.* 2005). GSEAPreranked, which uses gene set permutation, is recommended when sample numbers are too low for GSEA using phenotype permutation directly on counts data (Reimand *et al.* 2019), and the LFC is a popular metric for ranking genes (Calura and Martini 2021). GSEA reports when gene sets are enriched either at the top or bottom of the gene list (termed positive and negative enrichment, respectively); thus, we could check if the gene sets that were positively enriched in Severe/Ctrl matched those negatively enriched in Ctrl/Severe, as well as whether the results were affected by prefiltering the data. As shown in the violin plots in Fig. 3C, the unfiltered data gave very different results depending on which group was the reference, and only one gene set was deemed significant by the recommended threshold (FDR *q*-value <.05) (Reimand *et al.* 2019). If we had simply extracted results for Severe/Ctrl from the unfiltered dataset, we would have observed no significant gene set enrichment. However, the reciprocal analyses of the prefiltered data showed greater consistency, including four highly significant gene sets (detailed in the table, Fig. 2C, right). Two of these are sets of genes up-regulated in response to immunomodulatory cytokines, which makes sense in the context of *COVID* infection with severe pneumonia and agrees with the findings of the original paper (Armignacco *et al.* 2024).

In this article, we have demonstrated the ‘mirror-check’ issue when using lfcShrink() with the ‘apeglm’ algorithm, where discordant results can arise simply by switching the reference group, as is required to extract all the desired contrasts from multi-group analyses when using this algorithm. We have also provided the mirrorCheck package as a tool for monitoring this issue and to help determine which data cleaning methods may help when the issue arises. The technical reasons underpinning how some datasets lead to spurious results with ‘apeglm’ are beyond the scope of this paper. Furthermore, we have not explored the extent to which the mirror-check issue arises when using the other algorithms implemented with lfcShrink() (i.e. ‘ashr’ and ‘normal’) in this article, because users can extract all comparisons without switching the reference level when using these algorithms. However, if the mirrorCheck issue does arise when using these algorithms, reproducibility would depend on users choosing the same reference group. mirrorCheck’s run_DESeq_all_contrasts() function allows users to run lfcShrink() with ‘ashr’ and ‘normal’, so mirrorCheck may also assist with data cleaning and design decisions when using these algorithms.

## 4 Conclusions

Clinical RNA-seq datasets frequently involve multiple groups, high biological variability, and subtle inter-group differences. In large studies, hidden and unwanted sources of technical variation can also lead to spurious DGEA results. Our findings highlight that although DESeq2’s lfcShrink() conservatively ranks DEGs without resorting to arbitrary filtering of genes with low counts, such datasets can distort shrinkage estimation so greatly that it calls the reliability of the DEGs into question and can affect downstream analyses. mirrorCheck automates the process of extracting concordant DEG lists from reciprocal contrasts for multiple groups, and simplifies assessment of the degree of concordance, an essential quality control step which can inform users whether data cleaning steps such as prefiltering or SVA are necessary. This report draws attention to the need for a ‘mirror-check’ when using DESeq2’s lfcShrink() and presents a simple tool for this purpose.

Key points

- This article alerts users of the popular DESeq2 tool for differential expression analysis to a potential issue when using the lfcShrink() function.
- When the experimental design includes multiple groups, a trivial change in the workflow can dramatically alter the ranking of differentially expressed genes, giving rise to the ‘mirror-check’ issue.
- Sparse data exacerbates this, so although lfcShrink() was intended to make prefiltering unnecessary, some datasets may require prefiltering.
- Our mirrorCheck package facilitates use of DESeq2 to compare multiple groups and provides a straightforward quality control tool to aid data-driven decision-making during analysis.

## Supplementary Material

vbaf070_Supplementary_Data

## Data Availability

The RNA-Seq datasets analysed during the current study are available in the following repositories. Data from the TCGA-BRCA project, originally from dbGaP study phs000178.v11.p8, were accessed via the NCI Genomic Data Commons (TCGA-BRCA). The *Cell line* and *COVID* datasets were accessed as per the data availability sections of the original studies (Corchete *et al.* 2020, Armignacco *et al.* 2024). Namely, for *Cell line*, GSE95077_RAW.tar was downloaded from the Gene Expression Omnibus under the accession number GSE95077 (Rojas *et al.* 2024). For *COVID*, ‘COUNTS_matrix_ForPublication.txt’ was sourced from the EMBL-EBI BioStudies repository (reference number: S-BSST1135), and the publication’s Supplementary file 9 and Supplementary Table S1 was downloaded Armignacco *et al.* (2024). The mirrorCheck R package is available on GitHub at https://github.com/kescull/mirrorCheck, including documentation and instructions for installation (Scull 2024) and archived in Zenodo at https://doi.org/10.5281/zenodo.15093488. It is platform-independent and provided under the MIT licence. Output files from the present analysis including mirrorCheck results are archived in Monash Bridges (Scull 2025) at https://bridges.monash.edu/articles/dataset/mirrorCheck_results_for_4_public_datasets/27289017, DOI: https://doi.org/10.26180/27289017.v1.

## References

[vbaf070-B1] Armignacco R , Carlier N, Jouinot A et al; COVIDeF group. Whole blood transcriptome signature predicts severe forms of COVID-19: results from the COVIDeF cohort study. Funct Integr Genomics 2024;24:107. 10.1007/s10142-024-01359-2 38772950 PMC11108918

[vbaf070-B2] Berger AC , Korkut A, Kanchi RS et al; Cancer Genome Atlas Research Network. A comprehensive pan-cancer molecular study of gynecologic and breast cancers. Cancer Cell 2018;33:690–705.e699. 10.1016/j.ccell.2018.03.014 29622464 PMC5959730

[vbaf070-B3] Calura E , Martini P. Summarizing RNA-Seq data or differentially expressed genes using gene set, network, or pathway analysis. Methods Mol Biol 2021;2284:147–79. 10.1007/978-1-0716-1307-8_9 33835442

[vbaf070-B4] Chen Y , Lun AT, Smyth GK. From reads to genes to pathways: differential expression analysis of RNA-Seq experiments using Rsubread and the edgeR quasi-likelihood pipeline. F1000Res 2016;5:1438. 10.12688/f1000research.8987.2 27508061 PMC4934518

[vbaf070-B5] Ciriello G , Gatza ML, Beck AH et al; TCGA Research Network. Comprehensive molecular portraits of invasive lobular breast cancer. Cell 2015;163:506–19. 10.1016/j.cell.2015.09.033 26451490 PMC4603750

[vbaf070-B6] Colaprico A , Silva TC, Olsen C et al TCGAbiolinks: an R/Bioconductor package for integrative analysis of TCGA data. Nucleic Acids Res 2016;44:e71. 10.1093/nar/gkv1507 26704973 PMC4856967

[vbaf070-B7] Conway JR , Lex A, Gehlenborg N. UpSetR: an R package for the visualization of intersecting sets and their properties. Bioinformatics 2017;33:2938–40. 10.1093/bioinformatics/btx364 28645171 PMC5870712

[vbaf070-B8] Corchete LA , Rojas EA, Alonso-López D et al Systematic comparison and assessment of RNA-seq procedures for gene expression quantitative analysis. Sci Rep 2020;10:19737. 10.1038/s41598-020-76881-x 33184454 PMC7665074

[vbaf070-B9] Haglund F , Ma R, Huss M et al Evidence of a functional estrogen receptor in parathyroid adenomas. J Clin Endocrinol Metab 2012;97:4631–9. 10.1210/jc.2012-2484 23024189

[vbaf070-B10] Koboldt DC , Fulton RS, McLellan MD et al Comprehensive molecular portraits of human breast tumours. Nature 2012;490:61–70. 10.1038/nature11412 23000897 PMC3465532

[vbaf070-B11] Law CW , Chen Y, Shi W et al voom: precision weights unlock linear model analysis tools for RNA-seq read counts. Genome Biol 2014;15:R29. 10.1186/gb-2014-15-2-r29 24485249 PMC4053721

[vbaf070-B12] Leek JJ , Parker WE, Fertig HS et al sva: Surrogate Variable Analysis [Software]. Release 3.52.0. 2024. https://bioconductor.org/packages/svadate

[vbaf070-B13] Leek JT , Johnson WE, Parker HS et al The sva package for removing batch effects and other unwanted variation in high-throughput experiments. Bioinformatics 2012;28:882–3. 10.1093/bioinformatics/bts034 22257669 PMC3307112

[vbaf070-B14] Love M. apeglm—Influence of Reference Level on logFC and svalues [Web page]. Bioconductor forum. 2021. https://support.bioconductor.org/p/p134551/#p134561 (20 September, date last accessed).

[vbaf070-B15] Love MI , Anders S, Huber W. Analyzing RNA-seq data with DESeq2 [Web page]. 2024. https://www.bioconductor.org/packages/release/bioc/vignettes/DESeq2/inst/doc/DESeq2.html (26 August, date last accessed).

[vbaf070-B16] Love MI , Anders S, Kim V et al RNA-Seq workflow: gene-level exploratory analysis and differential expression [version 2; peer review: 2 approved]. F1000Research 2016;4:1070. 10.12688/f1000research.7035.2 PMC4670015 26674615

[vbaf070-B17] Love MI , Huber W, Anders S. Moderated estimation of fold change and dispersion for RNA-seq data with DESeq2. Genome Biol 2014;15:550. 10.1186/s13059-014-0550-8 25516281 PMC4302049

[vbaf070-B18] McCarthy DJ , Chen Y, Smyth GK. Differential expression analysis of multifactor RNA-Seq experiments with respect to biological variation. Nucleic Acids Res 2012;40:4288–97. 10.1093/nar/gks042 22287627 PMC3378882

[vbaf070-B19] Mootha VK , Lindgren CM, Eriksson K-F et al PGC-1α-responsive genes involved in oxidative phosphorylation are coordinately downregulated in human diabetes. Nat Genet 2003;34:267–73. 10.1038/ng1180. 12808457

[vbaf070-B20] Mounir M , Lucchetta M, Silva TC et al New functionalities in the TCGAbiolinks package for the study and integration of cancer data from GDC and GTEx. PLoS Comput Biol 2019;15:e1006701. 10.1371/journal.pcbi.1006701 30835723 PMC6420023

[vbaf070-B21] Reimand J , Isserlin R, Voisin V et al Pathway enrichment analysis and visualization of omics data using g: Profiler, GSEA, Cytoscape and EnrichmentMap. Nat Protoc 2019;14:482–517. 10.1038/s41596-018-0103-9 30664679 PMC6607905

[vbaf070-B22] Robinson MD , McCarthy DJ, Smyth GK. edgeR: a Bioconductor package for differential expression analysis of digital gene expression data. Bioinformatics 2010;26:139–40. 10.1093/bioinformatics/btp616 19910308 PMC2796818

[vbaf070-B23] Rojas EC , Corchete LA, San-Segundo L et al Amiloride, an old diuretic drug, is a potential therapeutic agent for multiple myeloma. Gene Expression Omnibus Series GSE95077 [Dataset]. 2024. https://www.ncbi.nlm.nih.gov/geo/query/acc.cgi?acc=GSE95077 (3 June 2024, date last accessed). 10.1158/1078-0432.CCR-17-0678 28790111

[vbaf070-B24] S-BSST1135. Whole Blood Transcriptome Signature Predicts Severe Forms of COVID-19: Results from the COVIDeF Cohort Study [Dataset]. BioStudies. Release date: 29 September 2024. https://www.ebi.ac.uk/biostudies/studies/S-BSST1135?key=62d4dc30-e0d4-4f4b-891f-58c5777a0cd3 (24 May 2024, date last accessed). 10.1007/s10142-024-01359-2 PMC11108918 38772950

[vbaf070-B25] Scull KE. mirrorCheck Github repository [Software]. Release 1.0.0. 2024. https://github.com/kescull/mirrorCheck, 10.5281/zenodo.15093488 (27 March 2025, date last accessed).

[vbaf070-B26] Scull KE. mirrorCheck Results for 4 Public Datasets [Dataset]. 2025. https://bridges.monash.edu/articles/dataset/mirrorCheck_results_for_4_public_datasets/27289017, 10.26180/27289017.v1 (27 March 2025, date last accessed).

[vbaf070-B27] Silva TC , Colaprico A, Olsen C et al TCGA workflow: analyze cancer genomics and epigenomics data using bioconductor packages. F1000Res 2016;5:1542. 10.12688/f1000research.8923.2 28232861 PMC5302158

[vbaf070-B28] Stephens M. False discovery rates: a new deal. Biostatistics 2017;18:275–94. 10.1093/biostatistics/kxw041 27756721 PMC5379932

[vbaf070-B29] Subramanian A , Tamayo P, Mootha VK et al Gene set enrichment analysis: a knowledge-based approach for interpreting genome-wide expression profiles. Proc Natl Acad Sci U S A 2005;102:15545–50. 10.1073/pnas.0506580102 16199517 PMC1239896

[vbaf070-B30] TCGA-BRCA. National Institutes of Health GDC Data Portal Project [Dataset]. Version v.40.0. Release date: March 29, 2024 https://portal.gdc.cancer.gov/projects/TCGA-BRCA (29 July 2024, date last accessed).

[vbaf070-B31] Zhu A , Ibrahim JG, Love MI. Heavy-tailed prior distributions for sequence count data: removing the noise and preserving large differences. Bioinformatics 2019;35:2084–92. 10.1093/bioinformatics/bty895 30395178 PMC6581436

