# Supplementary material for: mirrorCheck: an R package facilitating informed use of DESeq2’s lfcShrink() function for differential gene expression analysis of clinical samples
Source: Bioinform Adv. 2025 Apr 2;5(1):vbaf070. doi: 10.1093/bioadv/vbaf070 (PMC12089695; doi:10.1093/bioadv/vbaf070)
Supplement: vbaf070_Supplementary_Data [file vbaf070_supplementary_data.zip › final supps/S10_Supplementary_summary_figure_report.pdf]

Summary plots

Kate Scull

Summarising the three example mirrorCheck analyses

This analysis visualises results from the three example analyses found in Quartos TCGABRCA.qmd, Cellline.qmd and Covid.qmd. To render this Quarto, first render these three qmd files. Each of these qmds will create two rds files with results from mirrorCheck's `compare_reciprocal_contrasts()`, from DESeq analyses without cleaning, with prefiltering of the counts data, or using SVA to identify hidden sources of variation. Place these files in the same folder as this qmd file:

BRCA_noclean.rds, BRCA_prefilt.rds, BRCA_sva.rds, cell_noclean.rds, cell_prefilt.rds, covid_noclean.rds, covid_prefilt.rds

Prepare data

```r
library(tidyverse)
```

```
-- Attaching core tidyverse packages ------------------------ tidyverse 2.0.0 --
v dplyr     1.1.4     v readr     2.1.5
v forcats   1.0.0     v stringr   1.5.1
v ggplot2   3.5.1     v tibble    3.2.1
v lubridate 1.9.4     v tidyr     1.3.1
v purrr     1.0.2
-- Conflicts ------------------------------------------ tidyverse_conflicts() --
x dplyr::filter() masks stats::filter()
x dplyr::lag()    masks stats::lag()
i Use the conflicted package (<http://conflicted.r-lib.org/>) to force all conflicts to becom
```

```r
library(ggpubr)

fns <- c("BRCA_noclean.rds", "BRCA_prefilt.rds","BRCA_sva.rds",
         "cell_noclean.rds", "cell_prefilt.rds","cell_sva.rds",
         "covid_noclean.rds", "covid_prefilt.rds", "covid_sva.rds")
simple_names <- sub(".rds","",fns)
sets <- lapply(fns,readRDS)
names(sets) <- simple_names

sets <- lapply(sets,function(x) lapply(x, function(y) y %>% select(partition)))
sets <- lapply(sets,function(x) x %>% bind_rows(.id = "contrast"))
all.sets <- bind_rows(sets, .id = "group")

totals <- all.sets %>%
  group_by(group,contrast) %>%
  summarise(total.per.contrast = n()) %>%
  ungroup()
```

```
`summarise()` has grouped output by 'group'. You can override using the
`.groups` argument.
```

```r
head(totals)
```

```
# A tibble: 6 x 3
  group        contrast      total.per.contrast
  <chr>        <chr>                      <int>
1 BRCA_noclean Control.Basal               7483
2 BRCA_noclean Her2.Basal                  2766
3 BRCA_noclean Her2.Control                5257
4 BRCA_noclean LumA.Basal                  3249
5 BRCA_noclean LumA.Control                4655
6 BRCA_noclean LumA.Her2                   1243
```

```r
plottable <- all.sets %>%
  group_by(group,contrast) %>%
  count(partition) %>%
  pivot_wider(values_from = n, names_from = partition, values_fill = 0) %>%
  pivot_longer(c(concordant,group1ref,group2ref),names_to = "partition",
               values_to = "n") %>%
  left_join(totals) %>%
```

```r
  filter(partition == "concordant") %>%
  select(-partition) %>%
  rowwise() %>%
  mutate(discordance = 100 - (n/total.per.contrast*100),
         experiment = case_when(
           str_split_1(group,"_")[1] == "BRCA" ~ "BRCA",
           str_split_1(group,"_")[1] == "covid" ~ "COVID",
           str_split_1(group,"_")[1] == "cell" ~ "Cell line"),
         cleanup = case_when(
           str_split_1(group,"_")[2] == "noclean"~"no cleaning",
           str_split_1(group,"_")[2] == "prefilt"~"prefiltered",
           str_split_1(group,"_")[2] == "sva" ~ "sva")) %>%
  ungroup() %>%
  mutate(across(c(cleanup, experiment,contrast), as_factor),
         experiment = fct_relevel(experiment,"COVID","BRCA","Cell line"))
```

```
Joining with `by = join_by(group, contrast)`
```

```r
str(plottable)
```

```
tibble [108 x 7] (S3: tbl_df/tbl/data.frame)
 $ group             : chr [1:108] "BRCA_noclean" "BRCA_noclean" "BRCA_noclean" "BRCA_noclean
 $ contrast          : Factor w/ 36 levels "Control.Basal",..: 1 2 3 4 5 6 7 8 9 10 ...
 $ n                 : int [1:108] 4521 2761 4102 3241 2765 1239 4052 4164 831 321 ...
 $ total.per.contrast: int [1:108] 7483 2766 5257 3249 4655 1243 4059 5483 838 322 ...
 $ discordance       : num [1:108] 39.583 0.181 21.971 0.246 40.602 ...
 $ experiment        : Factor w/ 3 levels "COVID","BRCA",..: 2 2 2 2 2 2 2 2 2 2 ...
 $ cleanup           : Factor w/ 3 levels "no cleaning",..: 1 1 1 1 1 1 1 1 1 1 ...
```

```r
head(plottable)
```

```
# A tibble: 6 x 7
  group        contrast      n total.per.contrast discordance experiment cleanup
  <chr>        <fct>     <int>              <int>       <dbl> <fct>      <fct>
1 BRCA_noclean Control.~  4521               7483      39.6   BRCA       no cle~
2 BRCA_noclean Her2.Bas~  2761               2766       0.181 BRCA       no cle~
3 BRCA_noclean Her2.Con~  4102               5257      22.0   BRCA       no cle~
4 BRCA_noclean LumA.Bas~  3241               3249       0.246 BRCA       no cle~
5 BRCA_noclean LumA.Con~  2765               4655      40.6   BRCA       no cle~
6 BRCA_noclean LumA.Her2  1239               1243       0.322 BRCA       no cle~
```

Plot it

```
colours <- c(sva = '#EE7733',
             prefiltered = '#0077BB',
             "no cleaning" = '#BBBBBB')
discord <- ggplot(plottable, aes(x=cleanup, y=discordance, fill = cleanup)) +
  geom_violin(scale = "width") +
  geom_point(size = 2, show.legend = F) +
  facet_grid(~experiment, switch = "x") +
  scale_fill_manual(values = colours) +
  geom_line(aes(group = contrast)) +
  geom_point(data = plottable[plottable$contrast %in% "Severe.Ctrl", ],
             size = 3, color = "#009988",show.legend = F) +
  geom_line(data = plottable[plottable$contrast %in% "Severe.Ctrl", ],
            aes(group = contrast),color = "#009988", linewidth = 1) +
  labs(y = "% discordance\nin total DEGs per contrast") +
  theme_classic(base_size = 20) +
  theme(axis.ticks.x = element_blank(),
        axis.title.x = element_blank(),
        axis.text.x = element_blank(),
        legend.title = element_blank(),
        legend.position = "inside",
        legend.position.inside = c(0.8,0.8)) +
  guides(color = "none")

summed.concordant <- plottable %>%
  summarise(total = sum(n), .by = c(cleanup,experiment))
summed.concordant
```

```
# A tibble: 9 x 3
  cleanup     experiment total
  <fct>       <fct>      <int>
1 no cleaning BRCA       34156
2 prefiltered BRCA       25521
3 sva         BRCA       47308
4 no cleaning Cell line  63571
5 prefiltered Cell line  59352
6 sva         Cell line  56300
7 no cleaning COVID        205
8 prefiltered COVID        357
9 sva         COVID        257
```

```r
summed <- ggplot(summed.concordant,aes(y = total,
                                       x = cleanup,
                                       fill = cleanup)) +
  scale_fill_manual(values = colours) +
  geom_col() +
  facet_grid(~experiment,scale = "free") +
  geom_text(aes(label=total), vjust=-0.5, size = 5) +
  labs(y = "total\nconcordant\nDEGs") +
  expand_limits(y = 75000) +
  theme_classic(base_size = 20) +
  theme(legend.position = "none",
        axis.ticks.x = element_blank(),
        axis.title.x = element_blank(),
        axis.text.x = element_blank(),
        strip.background = element_blank(),
        strip.text.x = element_blank())

#or change in concordant DEGs per contrast
changes <- plottable %>% select(contrast,n,experiment,cleanup) %>%
  pivot_wider(names_from = cleanup,values_from = n) %>%
  rename(no_clean = "no cleaning") %>%
  rowwise() %>%
  mutate(change_prefilt = (prefiltered-no_clean)/no_clean*100,
         direction_prefilt = if_else(change_prefilt > 0, "UP","DOWN"),
         change_sva = (sva-no_clean)/no_clean*100,
         direction_sva =  if_else(change_sva > 0, "UP","DOWN")) %>%
  dplyr::select(-c(prefiltered,sva,no_clean)) %>%
  pivot_longer(!c(contrast,experiment),
               names_to = c(".value","cleanup"), names_sep = "_") %>%
  drop_na()

diff <- ggplot(changes,aes(y = change,
                           x = factor(cleanup,
                                      levels = c("no_clean","prefilt","sva")),
                           shape = direction)) +
  geom_point(size = 3, stroke = 0.8) +
  scale_shape_manual(values = c("UP" = 24,"DOWN" = 25)) +
  scale_x_discrete("cleanup", drop=FALSE) +
  geom_point(data = changes[changes$contrast %in% "Severe.Ctrl", ],
             size = 3, stroke = 0.8, fill = "#009988", na.rm=T) +
  facet_grid(~experiment, scale = "free") +
  theme_classic(base_size = 20) +
```

```
  theme(legend.position = "none",
        axis.ticks.x = element_blank(),
        axis.title.x = element_blank(),
        axis.text.x = element_blank(),
        strip.background = element_blank(),
        strip.text.x = element_blank()) +
  ylab("% change in\nconcordant DEGs\nper contrast") +
  geom_hline(yintercept = 0, linetype = "dashed")

figure <- ggarrange(summed,diff,discord,
                    ncol = 1, nrow = 3, heights = c(1,1.5,2), align = "v")
figure
```

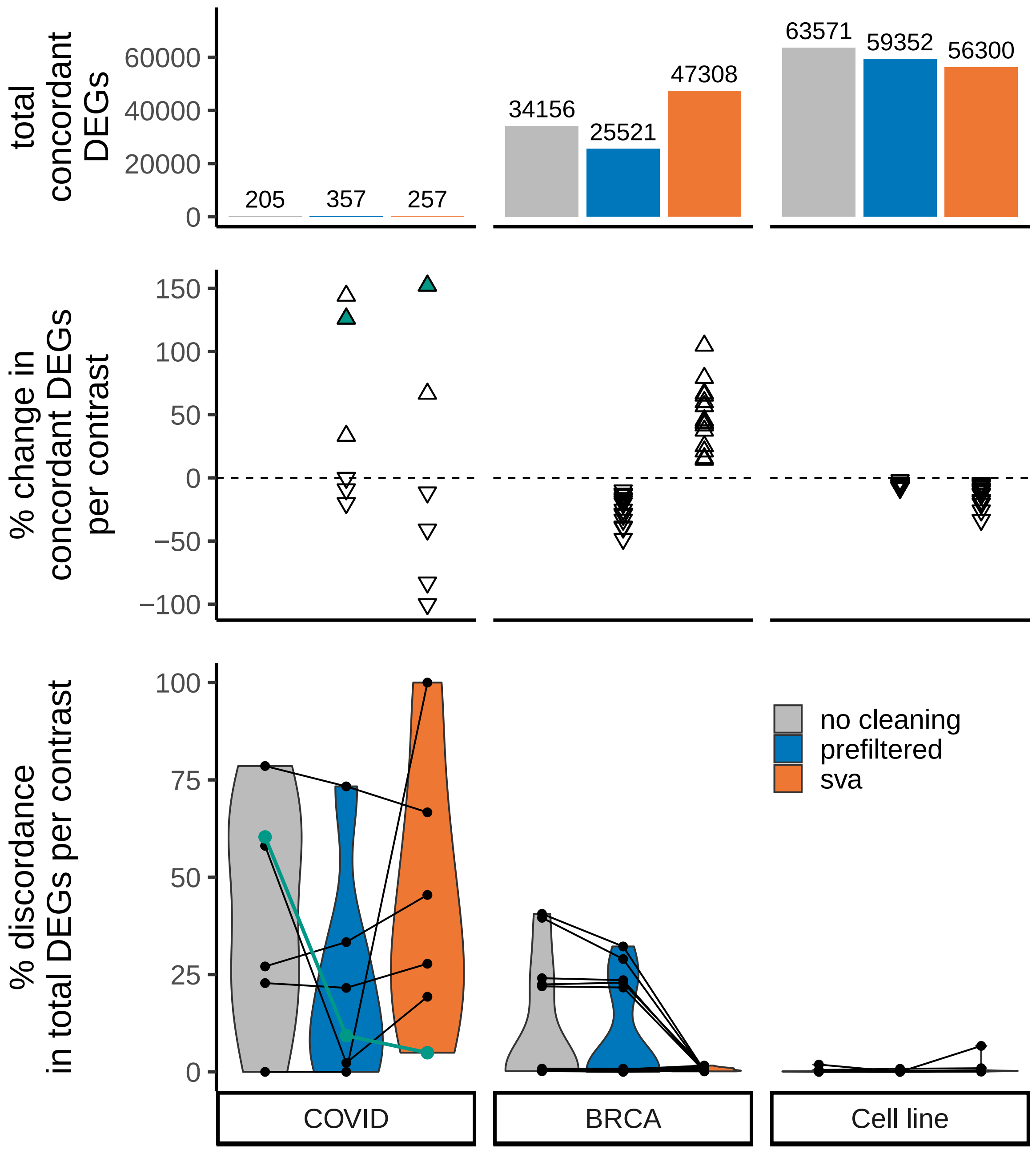

Session info

```r
sessionInfo()
```

```
R version 4.4.2 (2024-10-31)
Platform: x86_64-pc-linux-gnu
Running under: Ubuntu 22.04.5 LTS

Matrix products: default
BLAS:   /usr/lib/x86_64-linux-gnu/blas/libblas.so.3.10.0
LAPACK: /usr/lib/x86_64-linux-gnu/lapack/liblapack.so.3.10.0

locale:
 [1] LC_CTYPE=en_AU.UTF-8       LC_NUMERIC=C
 [3] LC_TIME=en_AU.UTF-8        LC_COLLATE=en_AU.UTF-8
 [5] LC_MONETARY=en_AU.UTF-8    LC_MESSAGES=en_AU.UTF-8
 [7] LC_PAPER=en_AU.UTF-8       LC_NAME=C
 [9] LC_ADDRESS=C               LC_TELEPHONE=C
[11] LC_MEASUREMENT=en_AU.UTF-8 LC_IDENTIFICATION=C

time zone: Australia/Melbourne
tzcode source: system (glibc)

attached base packages:
[1] stats     graphics  grDevices utils     datasets  methods   base

other attached packages:
 [1] ggpubr_0.6.0    lubridate_1.9.4 forcats_1.0.0   stringr_1.5.1
 [5] dplyr_1.1.4     purrr_1.0.2     readr_2.1.5     tidyr_1.3.1
 [9] tibble_3.2.1    ggplot2_3.5.1   tidyverse_2.0.0

loaded via a namespace (and not attached):
 [1] utf8_1.2.4        generics_0.1.3    rstatix_0.7.2     stringi_1.8.4
 [5] hms_1.1.3         digest_0.6.37     magrittr_2.0.3    evaluate_1.0.3
 [9] grid_4.4.2        timechange_0.3.0  fastmap_1.2.0     jsonlite_1.8.9
[13] backports_1.5.0   Formula_1.2-5     scales_1.3.0      abind_1.4-8
[17] cli_3.6.4         rlang_1.1.5       cowplot_1.1.3     munsell_0.5.1
[21] withr_3.0.2       yaml_2.3.10       tools_4.4.2       tzdb_0.4.0
[25] ggsignif_0.6.4    colorspace_2.1-1  broom_1.0.7       vctrs_0.6.5
[29] R6_2.6.1          lifecycle_1.0.4   car_3.1-3         pkgconfig_2.0.3
[33] pillar_1.10.1     gtable_0.3.6      glue_1.8.0        xfun_0.50
[37] tidyselect_1.2.1  knitr_1.49        farver_2.1.2      htmltools_0.5.8.1
[41] rmarkdown_2.29    carData_3.0-5     labeling_0.4.3    compiler_4.4.2
```
