# Supplementary material for: mirrorCheck: an R package facilitating informed use of DESeq2’s lfcShrink() function for differential gene expression analysis of clinical samples
Source: Bioinform Adv. 2025 Apr 2;5(1):vbaf070. doi: 10.1093/bioadv/vbaf070 (PMC12089695; doi:10.1093/bioadv/vbaf070)
Supplement: vbaf070_Supplementary_Data [file vbaf070_supplementary_data.zip › final supps/S2_Supplementary_Covid_report.pdf]

COVID

Kate Scull

COVID example data

This analysis uses data from Armignacco et al. (2024), https://doi.org/10.1007/s10142-024-01359-2.

As per the data availability section of Armignacco et al, “COUNTS_matrix_ForPublication.txt” was sourced from the EMBL-EBI BioStudies repository (reference number: S-BSST1135; https://www.ebi.ac.uk/biostudies/studies/S-BSST1135?key=62d4dc30-e0d4-4f4b-891f-58c5777a0cd3). The metadata file was converted to csv format from Supplementary file 9 - Supplementary Table S1, sourced from here.

To reproduce the Quarto document and other output files, download your own copies of these data files and place the qmd file in the same folder before rendering. You will need to ensure to following R libraries are installed, including mirrorCheck from the github repository kescull/mirrorCheck: Facilitator functions for getting and assessing DESeq2 lfcShrink results (github.com). The bash script at line **x** assumes you are running on a Linux machine and you will need to download GSEA and ensure the path on **line x r**efers to the correct executable on your computer. Alternatively, delete the gsea-prepare code chunk before rendering and run GSEA separately.

MirrorCheck

Setup:

```
library(mirrorCheck)
library(DESeq2)
library(tidyverse)
library(edgeR)
library(ggpubr)
library(UpSetR)
library(ComplexUpset)
library(ggh4x)
```

```
library(sva)
library(ggrepel)

test_match_order <- function(x,y) {
  if (isTRUE(all.equal(x,y))) print('Perfect match in same order')
  if (!isTRUE(all.equal(x,y)) && isTRUE(all.equal(sort(x),sort(y))))
    print('Perfect match in wrong order')
  if (!isTRUE(all.equal(x,y)) && !isTRUE(all.equal(sort(x),sort(y))))
    print('No match')
}
```

```
mat <- read.csv("COUNTS_matrix_ForPublication.txt", sep = "\t")
metadata <- read.csv("metadata.csv")
rownames(metadata) <- metadata$Sample
metadata$Pneumonia.type.and.evolution <-
  as.factor(metadata$Pneumonia.type.and.evolution)
test_match_order(rownames(metadata),colnames(mat))
```

```
[1] "Perfect match in same order"
```

```
dds <- DESeqDataSetFromMatrix(countData = mat,
                              colData = metadata,
                              design = ~Pneumonia.type.and.evolution)
```

Principal component analysis:

```
vsd <- vst(dds)
pdata <- DESeq2::plotPCA(vsd, ntop = length(vsd),
                         intgroup= "Pneumonia.type.and.evolution",
                         pcsToUse = 1:2, returnData=T)
```

```
using ntop=30633 top features by variance
```

```
percentVar <- attr(pdata,"percentVar")
colour_blind_friendly <- c('#EE7733', '#0077BB','#BBBBBB','#EE3377',
                           '#33BBEE', '#CC3311', '#009988' )
p <- ggplot(pdata, aes(x=PC1,y=PC2,color=Pneumonia.type.and.evolution)) +
  geom_point(size = 4,alpha = 0.6) +
```

```
  ggtitle("COVID Pneumonia") +
  labs(x = paste0("PC1: ",round(percentVar[1]*100),"% variance"),
       y = paste0("PC2: ",round(percentVar[2]*100),"% variance"),
       color = "Severity") +
  theme_classic(base_size = 16) +
  theme(plot.title = element_text(face = "bold")) +
  force_panelsizes(rows = unit(2.5,"in"),
                   cols = unit(2.5,"in")) +
  scale_colour_manual(values = colour_blind_friendly)
p
```

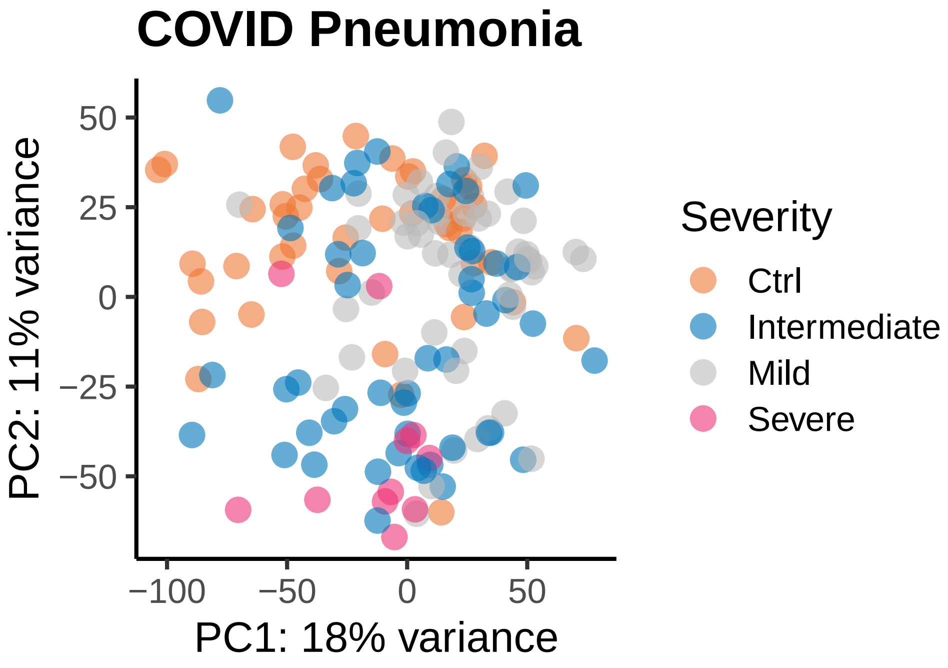

Run DESeq2

Run DESeq2 using lfcShrink, facilitated by mirrorCheck, *without further clean-up steps.* This creates output csv tables and pdf reports in a folder alongside this qmd file.

```
folder <- "DESeq_noclean"
dir.create(folder)

run_DESeq_all_contrasts(dds,folder,condition = "Pneumonia.type.and.evolution",
                        p.cutoff = 0.01,
```

```
                        #top.n = 0,
                        print.all = T,
                        useDingbats = T)
```

```
estimating size factors

estimating dispersions

gene-wise dispersion estimates

mean-dispersion relationship

final dispersion estimates

fitting model and testing

-- replacing outliers and refitting for 388 genes
-- DESeq argument 'minReplicatesForReplace' = 7
-- original counts are preserved in counts(dds)

estimating dispersions

fitting model and testing

using 'apeglm' for LFC shrinkage. If used in published research, please cite:
    Zhu, A., Ibrahim, J.G., Love, M.I. (2018) Heavy-tailed prior distributions for
    sequence count data: removing the noise and preserving large differences.
    Bioinformatics. https://doi.org/10.1093/bioinformatics/bty895
using 'apeglm' for LFC shrinkage. If used in published research, please cite:
    Zhu, A., Ibrahim, J.G., Love, M.I. (2018) Heavy-tailed prior distributions for
    sequence count data: removing the noise and preserving large differences.
    Bioinformatics. https://doi.org/10.1093/bioinformatics/bty895
using 'apeglm' for LFC shrinkage. If used in published research, please cite:
    Zhu, A., Ibrahim, J.G., Love, M.I. (2018) Heavy-tailed prior distributions for
    sequence count data: removing the noise and preserving large differences.
    Bioinformatics. https://doi.org/10.1093/bioinformatics/bty895
```

```
Warning in nbinomGLM(x = x, Y = YNZ, size = size, weights = weightsNZ, offset =
offsetNZ, : the line search routine failed, unable to sufficiently decrease the
function value
Warning in nbinomGLM(x = x, Y = YNZ, size = size, weights = weightsNZ, offset =
offsetNZ, : the line search routine failed, unable to sufficiently decrease the
function value
Warning in nbinomGLM(x = x, Y = YNZ, size = size, weights = weightsNZ, offset =
offsetNZ, : the line search routine failed, unable to sufficiently decrease the
function value
Warning in nbinomGLM(x = x, Y = YNZ, size = size, weights = weightsNZ, offset =
offsetNZ, : the line search routine failed, unable to sufficiently decrease the
function value

Warning in nbinomGLM(x = x, Y = YNZ, size = size, weights = weightsNZ, offset =
offsetNZ, : the line search routine failed, possibly due to insufficient
numeric precision
Warning in nbinomGLM(x = x, Y = YNZ, size = size, weights = weightsNZ, offset =
offsetNZ, : the line search routine failed, possibly due to insufficient
numeric precision

Warning in nbinomGLM(x = x, Y = YNZ, size = size, weights = weightsNZ, offset =
offsetNZ, : the line search routine failed, unable to sufficiently decrease the
function value

Warning in nbinomGLM(x = x, Y = YNZ, size = size, weights = weightsNZ, offset =
offsetNZ, : the line search routine failed, unable to sufficiently decrease the
function value
Warning in nbinomGLM(x = x, Y = YNZ, size = size, weights = weightsNZ, offset =
offsetNZ, : the line search routine failed, unable to sufficiently decrease the
function value
Warning in nbinomGLM(x = x, Y = YNZ, size = size, weights = weightsNZ, offset =
offsetNZ, : the line search routine failed, unable to sufficiently decrease the
function value
Warning in nbinomGLM(x = x, Y = YNZ, size = size, weights = weightsNZ, offset =
offsetNZ, : the line search routine failed, unable to sufficiently decrease the
function value

Warning in nbinomGLM(x = x, Y = YNZ, size = size, weights = weightsNZ, offset =
offsetNZ, : the line search routine failed, possibly due to insufficient
numeric precision
Warning in nbinomGLM(x = x, Y = YNZ, size = size, weights = weightsNZ, offset =
```

```
offsetNZ, : the line search routine failed, possibly due to insufficient
numeric precision

Warning in nbinomGLM(x = x, Y = YNZ, size = size, weights = weightsNZ, offset =
offsetNZ, : the line search routine failed, unable to sufficiently decrease the
function value
Warning in nbinomGLM(x = x, Y = YNZ, size = size, weights = weightsNZ, offset =
offsetNZ, : the line search routine failed, unable to sufficiently decrease the
function value
Warning in nbinomGLM(x = x, Y = YNZ, size = size, weights = weightsNZ, offset =
offsetNZ, : the line search routine failed, unable to sufficiently decrease the
function value
Warning in nbinomGLM(x = x, Y = YNZ, size = size, weights = weightsNZ, offset =
offsetNZ, : the line search routine failed, unable to sufficiently decrease the
function value
Warning in nbinomGLM(x = x, Y = YNZ, size = size, weights = weightsNZ, offset =
offsetNZ, : the line search routine failed, unable to sufficiently decrease the
function value

found results columns, replacing these
using 'apeglm' for LFC shrinkage. If used in published research, please cite:
    Zhu, A., Ibrahim, J.G., Love, M.I. (2018) Heavy-tailed prior distributions for
    sequence count data: removing the noise and preserving large differences.
    Bioinformatics. https://doi.org/10.1093/bioinformatics/bty895
using 'apeglm' for LFC shrinkage. If used in published research, please cite:
    Zhu, A., Ibrahim, J.G., Love, M.I. (2018) Heavy-tailed prior distributions for
    sequence count data: removing the noise and preserving large differences.
    Bioinformatics. https://doi.org/10.1093/bioinformatics/bty895
using 'apeglm' for LFC shrinkage. If used in published research, please cite:
    Zhu, A., Ibrahim, J.G., Love, M.I. (2018) Heavy-tailed prior distributions for
    sequence count data: removing the noise and preserving large differences.
    Bioinformatics. https://doi.org/10.1093/bioinformatics/bty895

Warning in nbinomGLM(x = x, Y = YNZ, size = size, weights = weightsNZ, offset =
offsetNZ, : the line search routine failed, possibly due to insufficient
numeric precision

Warning in nbinomGLM(x = x, Y = YNZ, size = size, weights = weightsNZ, offset =
offsetNZ, : the line search routine failed, unable to sufficiently decrease the
function value
Warning in nbinomGLM(x = x, Y = YNZ, size = size, weights = weightsNZ, offset =
```

```
offsetNZ, : the line search routine failed, unable to sufficiently decrease the
function value

Warning in nbinomGLM(x = x, Y = YNZ, size = size, weights = weightsNZ, offset =
offsetNZ, : the line search routine failed, possibly due to insufficient
numeric precision

Warning in nbinomGLM(x = x, Y = YNZ, size = size, weights = weightsNZ, offset =
offsetNZ, : the line search routine failed, unable to sufficiently decrease the
function value

Warning in nbinomGLM(x = x, Y = YNZ, size = size, weights = weightsNZ, offset =
offsetNZ, : the line search routine failed, possibly due to insufficient
numeric precision
Warning in nbinomGLM(x = x, Y = YNZ, size = size, weights = weightsNZ, offset =
offsetNZ, : the line search routine failed, possibly due to insufficient
numeric precision

Warning in nbinomGLM(x = x, Y = YNZ, size = size, weights = weightsNZ, offset =
offsetNZ, : the line search routine failed, unable to sufficiently decrease the
function value

Warning in nbinomGLM(x = x, Y = YNZ, size = size, weights = weightsNZ, offset =
offsetNZ, : the line search routine failed, possibly due to insufficient
numeric precision
Warning in nbinomGLM(x = x, Y = YNZ, size = size, weights = weightsNZ, offset =
offsetNZ, : the line search routine failed, possibly due to insufficient
numeric precision

Warning in nbinomGLM(x = x, Y = YNZ, size = size, weights = weightsNZ, offset =
offsetNZ, : the line search routine failed, unable to sufficiently decrease the
function value
Warning in nbinomGLM(x = x, Y = YNZ, size = size, weights = weightsNZ, offset =
offsetNZ, : the line search routine failed, unable to sufficiently decrease the
function value
Warning in nbinomGLM(x = x, Y = YNZ, size = size, weights = weightsNZ, offset =
offsetNZ, : the line search routine failed, unable to sufficiently decrease the
function value
```

```
Warning in nbinomGLM(x = x, Y = YNZ, size = size, weights = weightsNZ, offset =
offsetNZ, : the line search routine failed, unable to sufficiently decrease the
function value
Warning in nbinomGLM(x = x, Y = YNZ, size = size, weights = weightsNZ, offset =
offsetNZ, : the line search routine failed, unable to sufficiently decrease the
function value
Warning in nbinomGLM(x = x, Y = YNZ, size = size, weights = weightsNZ, offset =
offsetNZ, : the line search routine failed, unable to sufficiently decrease the
function value
Warning in nbinomGLM(x = x, Y = YNZ, size = size, weights = weightsNZ, offset =
offsetNZ, : the line search routine failed, unable to sufficiently decrease the
function value
Warning in nbinomGLM(x = x, Y = YNZ, size = size, weights = weightsNZ, offset =
offsetNZ, : the line search routine failed, unable to sufficiently decrease the
function value
Warning in nbinomGLM(x = x, Y = YNZ, size = size, weights = weightsNZ, offset =
offsetNZ, : the line search routine failed, unable to sufficiently decrease the
function value

found results columns, replacing these
using 'apeglm' for LFC shrinkage. If used in published research, please cite:
    Zhu, A., Ibrahim, J.G., Love, M.I. (2018) Heavy-tailed prior distributions for
    sequence count data: removing the noise and preserving large differences.
    Bioinformatics. https://doi.org/10.1093/bioinformatics/bty895
using 'apeglm' for LFC shrinkage. If used in published research, please cite:
    Zhu, A., Ibrahim, J.G., Love, M.I. (2018) Heavy-tailed prior distributions for
    sequence count data: removing the noise and preserving large differences.
    Bioinformatics. https://doi.org/10.1093/bioinformatics/bty895
using 'apeglm' for LFC shrinkage. If used in published research, please cite:
    Zhu, A., Ibrahim, J.G., Love, M.I. (2018) Heavy-tailed prior distributions for
    sequence count data: removing the noise and preserving large differences.
    Bioinformatics. https://doi.org/10.1093/bioinformatics/bty895

Warning in nbinomGLM(x = x, Y = YNZ, size = size, weights = weightsNZ, offset =
offsetNZ, : the line search routine failed, possibly due to insufficient
numeric precision

Warning in nbinomGLM(x = x, Y = YNZ, size = size, weights = weightsNZ, offset =
offsetNZ, : the line search routine failed, possibly due to insufficient
numeric precision
```

```
Warning in nbinomGLM(x = x, Y = YNZ, size = size, weights = weightsNZ, offset =
offsetNZ, : the line search routine failed, unable to sufficiently decrease the
function value
Warning in nbinomGLM(x = x, Y = YNZ, size = size, weights = weightsNZ, offset =
offsetNZ, : the line search routine failed, unable to sufficiently decrease the
function value
Warning in nbinomGLM(x = x, Y = YNZ, size = size, weights = weightsNZ, offset =
offsetNZ, : the line search routine failed, unable to sufficiently decrease the
function value
Warning in nbinomGLM(x = x, Y = YNZ, size = size, weights = weightsNZ, offset =
offsetNZ, : the line search routine failed, unable to sufficiently decrease the
function value
Warning in nbinomGLM(x = x, Y = YNZ, size = size, weights = weightsNZ, offset =
offsetNZ, : the line search routine failed, unable to sufficiently decrease the
function value
Warning in nbinomGLM(x = x, Y = YNZ, size = size, weights = weightsNZ, offset =
offsetNZ, : the line search routine failed, unable to sufficiently decrease the
function value

Warning in nbinomGLM(x = x, Y = YNZ, size = size, weights = weightsNZ, offset =
offsetNZ, : the line search routine failed, possibly due to insufficient
numeric precision

Warning in nbinomGLM(x = x, Y = YNZ, size = size, weights = weightsNZ, offset =
offsetNZ, : the line search routine failed, unable to sufficiently decrease the
function value
Warning in nbinomGLM(x = x, Y = YNZ, size = size, weights = weightsNZ, offset =
offsetNZ, : the line search routine failed, unable to sufficiently decrease the
function value
Warning in nbinomGLM(x = x, Y = YNZ, size = size, weights = weightsNZ, offset =
offsetNZ, : the line search routine failed, unable to sufficiently decrease the
function value
Warning in nbinomGLM(x = x, Y = YNZ, size = size, weights = weightsNZ, offset =
offsetNZ, : the line search routine failed, unable to sufficiently decrease the
function value

Warning in nbinomGLM(x = x, Y = YNZ, size = size, weights = weightsNZ, offset =
offsetNZ, : the line search routine failed, unable to sufficiently decrease the
function value
Warning in nbinomGLM(x = x, Y = YNZ, size = size, weights = weightsNZ, offset =
offsetNZ, : the line search routine failed, unable to sufficiently decrease the
function value
```

```
Warning in nbinomGLM(x = x, Y = YNZ, size = size, weights = weightsNZ, offset =
offsetNZ, : the line search routine failed, unable to sufficiently decrease the
function value
Warning in nbinomGLM(x = x, Y = YNZ, size = size, weights = weightsNZ, offset =
offsetNZ, : the line search routine failed, unable to sufficiently decrease the
function value
Warning in nbinomGLM(x = x, Y = YNZ, size = size, weights = weightsNZ, offset =
offsetNZ, : the line search routine failed, unable to sufficiently decrease the
function value

Warning in nbinomGLM(x = x, Y = YNZ, size = size, weights = weightsNZ, offset =
offsetNZ, : the line search routine failed, possibly due to insufficient
numeric precision

Warning in nbinomGLM(x = x, Y = YNZ, size = size, weights = weightsNZ, offset =
offsetNZ, : the line search routine failed, unable to sufficiently decrease the
function value
Warning in nbinomGLM(x = x, Y = YNZ, size = size, weights = weightsNZ, offset =
offsetNZ, : the line search routine failed, unable to sufficiently decrease the
function value

found results columns, replacing these
using 'apeglm' for LFC shrinkage. If used in published research, please cite:
    Zhu, A., Ibrahim, J.G., Love, M.I. (2018) Heavy-tailed prior distributions for
    sequence count data: removing the noise and preserving large differences.
    Bioinformatics. https://doi.org/10.1093/bioinformatics/bty895

Warning in nbinomGLM(x = x, Y = YNZ, size = size, weights = weightsNZ, offset =
offsetNZ, : the line search routine failed, unable to sufficiently decrease the
function value

Warning in nbinomGLM(x = x, Y = YNZ, size = size, weights = weightsNZ, offset =
offsetNZ, : the line search routine failed, unable to sufficiently decrease the
function value
Warning in nbinomGLM(x = x, Y = YNZ, size = size, weights = weightsNZ, offset =
offsetNZ, : the line search routine failed, unable to sufficiently decrease the
function value

Warning in nbinomGLM(x = x, Y = YNZ, size = size, weights = weightsNZ, offset =
offsetNZ, : the line search routine failed, possibly due to insufficient
numeric precision
```

```
Warning in nbinomGLM(x = x, Y = YNZ, size = size, weights = weightsNZ, offset =
offsetNZ, : the line search routine failed, unable to sufficiently decrease the
function value
Warning in nbinomGLM(x = x, Y = YNZ, size = size, weights = weightsNZ, offset =
offsetNZ, : the line search routine failed, unable to sufficiently decrease the
function value
Warning in nbinomGLM(x = x, Y = YNZ, size = size, weights = weightsNZ, offset =
offsetNZ, : the line search routine failed, unable to sufficiently decrease the
function value
Warning in nbinomGLM(x = x, Y = YNZ, size = size, weights = weightsNZ, offset =
offsetNZ, : the line search routine failed, unable to sufficiently decrease the
function value
Warning in nbinomGLM(x = x, Y = YNZ, size = size, weights = weightsNZ, offset =
offsetNZ, : the line search routine failed, unable to sufficiently decrease the
function value
Warning in nbinomGLM(x = x, Y = YNZ, size = size, weights = weightsNZ, offset =
offsetNZ, : the line search routine failed, unable to sufficiently decrease the
function value
Warning in nbinomGLM(x = x, Y = YNZ, size = size, weights = weightsNZ, offset =
offsetNZ, : the line search routine failed, unable to sufficiently decrease the
function value
Warning in nbinomGLM(x = x, Y = YNZ, size = size, weights = weightsNZ, offset =
offsetNZ, : the line search routine failed, unable to sufficiently decrease the
function value

Warning in nbinomGLM(x = x, Y = YNZ, size = size, weights = weightsNZ, offset =
offsetNZ, : the line search routine failed, possibly due to insufficient
numeric precision

Warning in nbinomGLM(x = x, Y = YNZ, size = size, weights = weightsNZ, offset =
offsetNZ, : the line search routine failed, unable to sufficiently decrease the
function value

Warning in nbinomGLM(x = x, Y = YNZ, size = size, weights = weightsNZ, offset =
offsetNZ, : the line search routine failed, possibly due to insufficient
numeric precision

Warning in nbinomGLM(x = x, Y = YNZ, size = size, weights = weightsNZ, offset =
offsetNZ, : the line search routine failed, unable to sufficiently decrease the
function value
```

```
Warning in nbinomGLM(x = x, Y = YNZ, size = size, weights = weightsNZ, offset =
offsetNZ, : the line search routine failed, possibly due to insufficient
numeric precision

Warning in nbinomGLM(x = x, Y = YNZ, size = size, weights = weightsNZ, offset =
offsetNZ, : the line search routine failed, unable to sufficiently decrease the
function value
Warning in nbinomGLM(x = x, Y = YNZ, size = size, weights = weightsNZ, offset =
offsetNZ, : the line search routine failed, unable to sufficiently decrease the
function value
Warning in nbinomGLM(x = x, Y = YNZ, size = size, weights = weightsNZ, offset =
offsetNZ, : the line search routine failed, unable to sufficiently decrease the
function value
Warning in nbinomGLM(x = x, Y = YNZ, size = size, weights = weightsNZ, offset =
offsetNZ, : the line search routine failed, unable to sufficiently decrease the
function value
Warning in nbinomGLM(x = x, Y = YNZ, size = size, weights = weightsNZ, offset =
offsetNZ, : the line search routine failed, unable to sufficiently decrease the
function value

Warning in nbinomGLM(x = x, Y = YNZ, size = size, weights = weightsNZ, offset =
offsetNZ, : the line search routine failed, possibly due to insufficient
numeric precision

using 'apeglm' for LFC shrinkage. If used in published research, please cite:
    Zhu, A., Ibrahim, J.G., Love, M.I. (2018) Heavy-tailed prior distributions for
    sequence count data: removing the noise and preserving large differences.
    Bioinformatics. https://doi.org/10.1093/bioinformatics/bty895

Warning in nbinomGLM(x = x, Y = YNZ, size = size, weights = weightsNZ, offset =
offsetNZ, : the line search routine failed, unable to sufficiently decrease the
function value

Warning in nbinomGLM(x = x, Y = YNZ, size = size, weights = weightsNZ, offset =
offsetNZ, : the line search routine failed, unable to sufficiently decrease the
function value
Warning in nbinomGLM(x = x, Y = YNZ, size = size, weights = weightsNZ, offset =
offsetNZ, : the line search routine failed, unable to sufficiently decrease the
function value
Warning in nbinomGLM(x = x, Y = YNZ, size = size, weights = weightsNZ, offset =
offsetNZ, : the line search routine failed, unable to sufficiently decrease the
```

```
function value
Warning in nbinomGLM(x = x, Y = YNZ, size = size, weights = weightsNZ, offset =
offsetNZ, : the line search routine failed, unable to sufficiently decrease the
function value
Warning in nbinomGLM(x = x, Y = YNZ, size = size, weights = weightsNZ, offset =
offsetNZ, : the line search routine failed, unable to sufficiently decrease the
function value
Warning in nbinomGLM(x = x, Y = YNZ, size = size, weights = weightsNZ, offset =
offsetNZ, : the line search routine failed, unable to sufficiently decrease the
function value
Warning in nbinomGLM(x = x, Y = YNZ, size = size, weights = weightsNZ, offset =
offsetNZ, : the line search routine failed, unable to sufficiently decrease the
function value
Warning in nbinomGLM(x = x, Y = YNZ, size = size, weights = weightsNZ, offset =
offsetNZ, : the line search routine failed, unable to sufficiently decrease the
function value
Warning in nbinomGLM(x = x, Y = YNZ, size = size, weights = weightsNZ, offset =
offsetNZ, : the line search routine failed, unable to sufficiently decrease the
function value
Warning in nbinomGLM(x = x, Y = YNZ, size = size, weights = weightsNZ, offset =
offsetNZ, : the line search routine failed, unable to sufficiently decrease the
function value

Warning in nbinomGLM(x = x, Y = YNZ, size = size, weights = weightsNZ, offset =
offsetNZ, : the line search routine failed, possibly due to insufficient
numeric precision
Warning in nbinomGLM(x = x, Y = YNZ, size = size, weights = weightsNZ, offset =
offsetNZ, : the line search routine failed, possibly due to insufficient
numeric precision
Warning in nbinomGLM(x = x, Y = YNZ, size = size, weights = weightsNZ, offset =
offsetNZ, : the line search routine failed, possibly due to insufficient
numeric precision

Warning in nbinomGLM(x = x, Y = YNZ, size = size, weights = weightsNZ, offset =
offsetNZ, : the line search routine failed, unable to sufficiently decrease the
function value

Warning in nbinomGLM(x = x, Y = YNZ, size = size, weights = weightsNZ, offset =
offsetNZ, : the line search routine failed, possibly due to insufficient
numeric precision
```

```
Warning in nbinomGLM(x = x, Y = YNZ, size = size, weights = weightsNZ, offset =
offsetNZ, : the line search routine failed, unable to sufficiently decrease the
function value

Warning in nbinomGLM(x = x, Y = YNZ, size = size, weights = weightsNZ, offset =
offsetNZ, : the line search routine failed, possibly due to insufficient
numeric precision
Warning in nbinomGLM(x = x, Y = YNZ, size = size, weights = weightsNZ, offset =
offsetNZ, : the line search routine failed, possibly due to insufficient
numeric precision

Warning in nbinomGLM(x = x, Y = YNZ, size = size, weights = weightsNZ, offset =
offsetNZ, : the line search routine failed, unable to sufficiently decrease the
function value
Warning in nbinomGLM(x = x, Y = YNZ, size = size, weights = weightsNZ, offset =
offsetNZ, : the line search routine failed, unable to sufficiently decrease the
function value
Warning in nbinomGLM(x = x, Y = YNZ, size = size, weights = weightsNZ, offset =
offsetNZ, : the line search routine failed, unable to sufficiently decrease the
function value

Warning in nbinomGLM(x = x, Y = YNZ, size = size, weights = weightsNZ, offset =
offsetNZ, : the line search routine failed, possibly due to insufficient
numeric precision

using 'apeglm' for LFC shrinkage. If used in published research, please cite:
    Zhu, A., Ibrahim, J.G., Love, M.I. (2018) Heavy-tailed prior distributions for
    sequence count data: removing the noise and preserving large differences.
    Bioinformatics. https://doi.org/10.1093/bioinformatics/bty895

Warning in nbinomGLM(x = x, Y = YNZ, size = size, weights = weightsNZ, offset =
offsetNZ, : the line search routine failed, unable to sufficiently decrease the
function value

Warning in nbinomGLM(x = x, Y = YNZ, size = size, weights = weightsNZ, offset =
offsetNZ, : the line search routine failed, unable to sufficiently decrease the
function value
Warning in nbinomGLM(x = x, Y = YNZ, size = size, weights = weightsNZ, offset =
offsetNZ, : the line search routine failed, unable to sufficiently decrease the
function value
Warning in nbinomGLM(x = x, Y = YNZ, size = size, weights = weightsNZ, offset =
```

```
offsetNZ, : the line search routine failed, unable to sufficiently decrease the
function value
Warning in nbinomGLM(x = x, Y = YNZ, size = size, weights = weightsNZ, offset =
offsetNZ, : the line search routine failed, unable to sufficiently decrease the
function value
Warning in nbinomGLM(x = x, Y = YNZ, size = size, weights = weightsNZ, offset =
offsetNZ, : the line search routine failed, unable to sufficiently decrease the
function value

Warning in nbinomGLM(x = x, Y = YNZ, size = size, weights = weightsNZ, offset =
offsetNZ, : the line search routine failed, unable to sufficiently decrease the
function value
Warning in nbinomGLM(x = x, Y = YNZ, size = size, weights = weightsNZ, offset =
offsetNZ, : the line search routine failed, unable to sufficiently decrease the
function value
Warning in nbinomGLM(x = x, Y = YNZ, size = size, weights = weightsNZ, offset =
offsetNZ, : the line search routine failed, unable to sufficiently decrease the
function value
Warning in nbinomGLM(x = x, Y = YNZ, size = size, weights = weightsNZ, offset =
offsetNZ, : the line search routine failed, unable to sufficiently decrease the
function value
Warning in nbinomGLM(x = x, Y = YNZ, size = size, weights = weightsNZ, offset =
offsetNZ, : the line search routine failed, unable to sufficiently decrease the
function value
Warning in nbinomGLM(x = x, Y = YNZ, size = size, weights = weightsNZ, offset =
offsetNZ, : the line search routine failed, unable to sufficiently decrease the
function value
Warning in nbinomGLM(x = x, Y = YNZ, size = size, weights = weightsNZ, offset =
offsetNZ, : the line search routine failed, unable to sufficiently decrease the
function value
Warning in nbinomGLM(x = x, Y = YNZ, size = size, weights = weightsNZ, offset =
offsetNZ, : the line search routine failed, unable to sufficiently decrease the
function value
Warning in nbinomGLM(x = x, Y = YNZ, size = size, weights = weightsNZ, offset =
offsetNZ, : the line search routine failed, unable to sufficiently decrease the
function value
Warning in nbinomGLM(x = x, Y = YNZ, size = size, weights = weightsNZ, offset =
offsetNZ, : the line search routine failed, unable to sufficiently decrease the
function value

Warning in nbinomGLM(x = x, Y = YNZ, size = size, weights = weightsNZ, offset =
offsetNZ, : the line search routine failed, possibly due to insufficient
```

```
numeric precision
```

```
group <- levels(as.factor(dds$Pneumonia.type.and.evolution))
set <- compare_reciprocal_contrasts(group,folder)
saveRDS(set,"covid_noclean.rds")
```

Print resized diagnostic stacked bar chart for publication:

```
make_diagnostic <- function(this_set) {
  ready.for.overlap.check <- lapply(this_set,function(x) x %>%
                                   select(partition) %>%
                                   mutate(total = n()) %>%
                                   group_by(partition) %>%
                                   summarise(n = n(),
                                             total = sum(total)/n,
                                             pc = n/total *100))
  all.overlap <- bind_rows(ready.for.overlap.check, .id = "group")

  group.labels <- gsub("\\.","\n", all.overlap$group)
  names(group.labels) <- all.overlap$group
  legend.labels <- c("Concordant",
                     "Only with group1 as ref",
                     "Only with group2 as ref")

  barPlot <- ggplot(all.overlap, aes(x = group,
                                  y = pc,
                                  fill = partition,
                                  label = n)) +
    geom_bar(stat = "identity") +
    xlab(element_blank()) +
    ylab("Percentage of differentially expressed genes") +
    scale_x_discrete(labels=group.labels) +
    theme_classic(base_size = 13) +
    theme(legend.position = "right",
        legend.box.spacing = unit(0,"cm"),
        axis.text = element_text(face="bold")) +
    guides(fill = guide_legend(title = element_blank(),reverse=T)) +
    scale_fill_manual(labels = legend.labels,
                    values = c('#009988', '#EE7733','#CC3311')) +
    geom_text(position = position_stack(vjust = 0.5),size = 4) +
    coord_flip()
  barPlot
```

```
}
make_diagnostic(set)
```

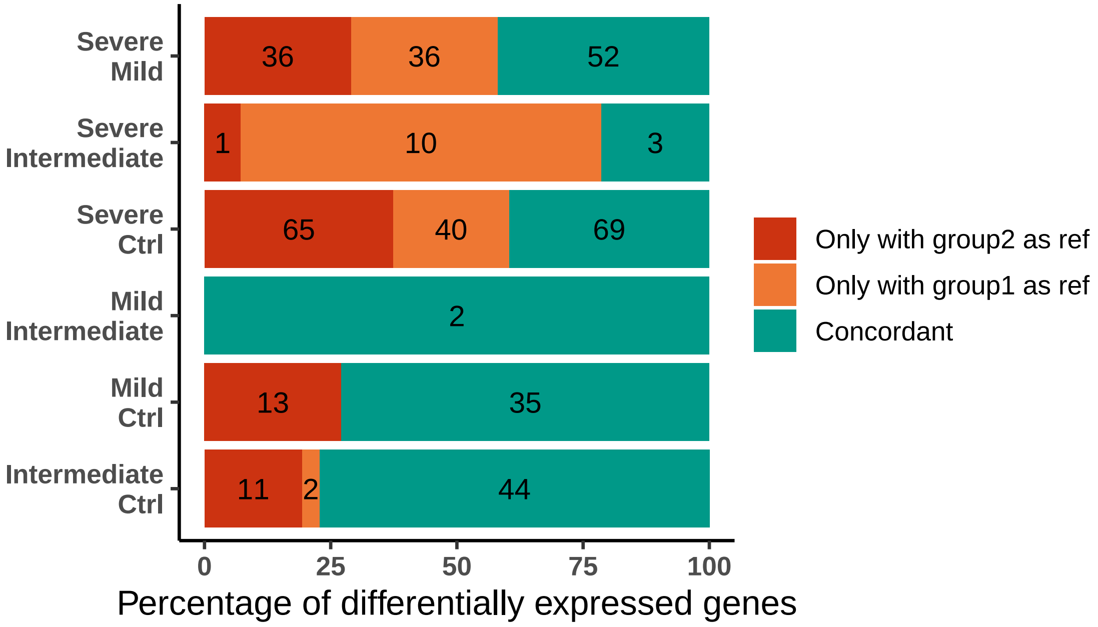

Next run DESeq2 using lfcShrink, facilitated by mirrorCheck, *after prefiltering.* This creates output csv tables and pdf reports in a folder alongside this qmd file.

```
folder <- "DESeq_prefilt"
dir.create(folder)

expr.filter <- edgeR::filterByExpr(dds,
                                   group = dds$Pneumonia.type.and.evolution)
dds.filtered <- dds[expr.filter, ]

run_DESeq_all_contrasts(dds.filtered,folder,
                        condition = "Pneumonia.type.and.evolution",
                        p.cutoff = 0.01,
                        #top.n = 0,
                        print.all = T,
                        useDingbats = T)
```

```
estimating size factors
```

```
estimating dispersions
```

```
gene-wise dispersion estimates

mean-dispersion relationship

final dispersion estimates

fitting model and testing

-- replacing outliers and refitting for 243 genes
-- DESeq argument 'minReplicatesForReplace' = 7
-- original counts are preserved in counts(dds)

estimating dispersions

fitting model and testing

using 'apeglm' for LFC shrinkage. If used in published research, please cite:
    Zhu, A., Ibrahim, J.G., Love, M.I. (2018) Heavy-tailed prior distributions for
    sequence count data: removing the noise and preserving large differences.
    Bioinformatics. https://doi.org/10.1093/bioinformatics/bty895
using 'apeglm' for LFC shrinkage. If used in published research, please cite:
    Zhu, A., Ibrahim, J.G., Love, M.I. (2018) Heavy-tailed prior distributions for
    sequence count data: removing the noise and preserving large differences.
    Bioinformatics. https://doi.org/10.1093/bioinformatics/bty895
using 'apeglm' for LFC shrinkage. If used in published research, please cite:
    Zhu, A., Ibrahim, J.G., Love, M.I. (2018) Heavy-tailed prior distributions for
    sequence count data: removing the noise and preserving large differences.
    Bioinformatics. https://doi.org/10.1093/bioinformatics/bty895

found results columns, replacing these

using 'apeglm' for LFC shrinkage. If used in published research, please cite:
    Zhu, A., Ibrahim, J.G., Love, M.I. (2018) Heavy-tailed prior distributions for
    sequence count data: removing the noise and preserving large differences.
    Bioinformatics. https://doi.org/10.1093/bioinformatics/bty895
using 'apeglm' for LFC shrinkage. If used in published research, please cite:
    Zhu, A., Ibrahim, J.G., Love, M.I. (2018) Heavy-tailed prior distributions for
    sequence count data: removing the noise and preserving large differences.
    Bioinformatics. https://doi.org/10.1093/bioinformatics/bty895
using 'apeglm' for LFC shrinkage. If used in published research, please cite:
```

```
    Zhu, A., Ibrahim, J.G., Love, M.I. (2018) Heavy-tailed prior distributions for
    sequence count data: removing the noise and preserving large differences.
    Bioinformatics. https://doi.org/10.1093/bioinformatics/bty895

found results columns, replacing these

using 'apeglm' for LFC shrinkage. If used in published research, please cite:
    Zhu, A., Ibrahim, J.G., Love, M.I. (2018) Heavy-tailed prior distributions for
    sequence count data: removing the noise and preserving large differences.
    Bioinformatics. https://doi.org/10.1093/bioinformatics/bty895
using 'apeglm' for LFC shrinkage. If used in published research, please cite:
    Zhu, A., Ibrahim, J.G., Love, M.I. (2018) Heavy-tailed prior distributions for
    sequence count data: removing the noise and preserving large differences.
    Bioinformatics. https://doi.org/10.1093/bioinformatics/bty895
using 'apeglm' for LFC shrinkage. If used in published research, please cite:
    Zhu, A., Ibrahim, J.G., Love, M.I. (2018) Heavy-tailed prior distributions for
    sequence count data: removing the noise and preserving large differences.
    Bioinformatics. https://doi.org/10.1093/bioinformatics/bty895

found results columns, replacing these

using 'apeglm' for LFC shrinkage. If used in published research, please cite:
    Zhu, A., Ibrahim, J.G., Love, M.I. (2018) Heavy-tailed prior distributions for
    sequence count data: removing the noise and preserving large differences.
    Bioinformatics. https://doi.org/10.1093/bioinformatics/bty895
using 'apeglm' for LFC shrinkage. If used in published research, please cite:
    Zhu, A., Ibrahim, J.G., Love, M.I. (2018) Heavy-tailed prior distributions for
    sequence count data: removing the noise and preserving large differences.
    Bioinformatics. https://doi.org/10.1093/bioinformatics/bty895
using 'apeglm' for LFC shrinkage. If used in published research, please cite:
    Zhu, A., Ibrahim, J.G., Love, M.I. (2018) Heavy-tailed prior distributions for
    sequence count data: removing the noise and preserving large differences.
    Bioinformatics. https://doi.org/10.1093/bioinformatics/bty895
```

```r
group <- levels(as.factor(dds.filtered$Pneumonia.type.and.evolution))
set_prefilt <- compare_reciprocal_contrasts(group,folder)
saveRDS(set_prefilt,"covid_prefilt.rds")
```

Print resized diagnostic stacked bar chart for publication:

```r
make_diagnostic(set_prefilt)
```

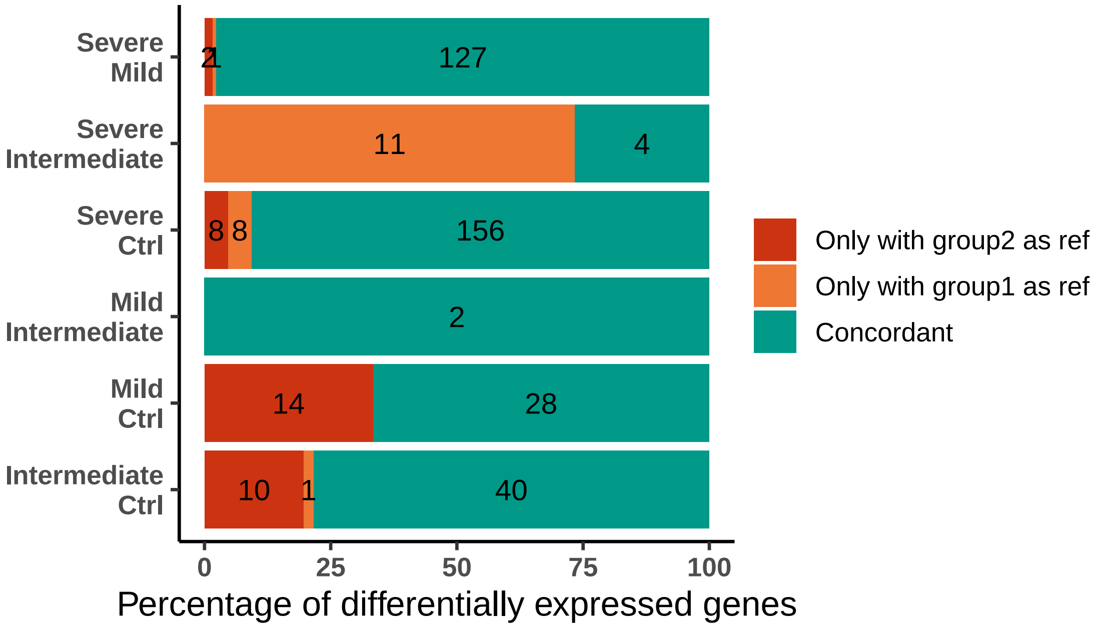

Finally, we try *surrogate variable analysis* (SVA) to remove unwanted/hidden sources of variation. The presence of unknown sources of variation can interfere with DGEA and increase discordance in reciprocal contrasts, so we use SVA to see if we can identify these as per the method in Love et al. (2016), before running DESeq with mirrorCheck. This creates output csv tables and pdf reports in a folder alongside this qmd file.

```r
folder <- "DESeq_sva"
dir.create(folder)

dds <- DESeq(dds)
```

```
estimating size factors

estimating dispersions

gene-wise dispersion estimates

mean-dispersion relationship

final dispersion estimates
```

```
fitting model and testing

-- replacing outliers and refitting for 388 genes
-- DESeq argument 'minReplicatesForReplace' = 7
-- original counts are preserved in counts(dds)

estimating dispersions

fitting model and testing
```

```
dat <- counts(dds, normalized=TRUE)
idx <- rowMeans(dat) > 1
dat <- dat[idx,]
mod <- model.matrix(~ Pneumonia.type.and.evolution, colData(dds))
mod0 <- model.matrix(~ 1, colData(dds))

#find probable number of unwanted variables
num_sv <- num.sv(dat,mod,method = "be")
num_sv
```

```
[1] 3
```

```
#num_sv = 3
svseq <- svaseq(dat, mod, mod0, n.sv=num_sv)
```

```
Number of significant surrogate variables is:  3
Iteration (out of 5 ):1  2  3  4  5
```

```
dds.sva <- dds
dds.sva$SV1 <- svseq$sv[,1]
dds.sva$SV2 <- svseq$sv[,2]
dds.sva$SV3 <- svseq$sv[,3]
design(dds.sva) <- ~ SV1 + SV2 + SV3 + Pneumonia.type.and.evolution

run_DESeq_all_contrasts(dds.sva,folder,
                        condition = "Pneumonia.type.and.evolution",
                        p.cutoff = 0.01,
                        #top.n = 0,
                        print.all = T,
                        useDingbats = T)
```

```
using pre-existing size factors

estimating dispersions

found already estimated dispersions, replacing these

gene-wise dispersion estimates

mean-dispersion relationship

final dispersion estimates

fitting model and testing

using 'apeglm' for LFC shrinkage. If used in published research, please cite:
    Zhu, A., Ibrahim, J.G., Love, M.I. (2018) Heavy-tailed prior distributions for
    sequence count data: removing the noise and preserving large differences.
    Bioinformatics. https://doi.org/10.1093/bioinformatics/bty895
using 'apeglm' for LFC shrinkage. If used in published research, please cite:
    Zhu, A., Ibrahim, J.G., Love, M.I. (2018) Heavy-tailed prior distributions for
    sequence count data: removing the noise and preserving large differences.
    Bioinformatics. https://doi.org/10.1093/bioinformatics/bty895
using 'apeglm' for LFC shrinkage. If used in published research, please cite:
    Zhu, A., Ibrahim, J.G., Love, M.I. (2018) Heavy-tailed prior distributions for
    sequence count data: removing the noise and preserving large differences.
    Bioinformatics. https://doi.org/10.1093/bioinformatics/bty895

found results columns, replacing these

using 'apeglm' for LFC shrinkage. If used in published research, please cite:
    Zhu, A., Ibrahim, J.G., Love, M.I. (2018) Heavy-tailed prior distributions for
    sequence count data: removing the noise and preserving large differences.
    Bioinformatics. https://doi.org/10.1093/bioinformatics/bty895
using 'apeglm' for LFC shrinkage. If used in published research, please cite:
    Zhu, A., Ibrahim, J.G., Love, M.I. (2018) Heavy-tailed prior distributions for
    sequence count data: removing the noise and preserving large differences.
    Bioinformatics. https://doi.org/10.1093/bioinformatics/bty895
using 'apeglm' for LFC shrinkage. If used in published research, please cite:
    Zhu, A., Ibrahim, J.G., Love, M.I. (2018) Heavy-tailed prior distributions for
    sequence count data: removing the noise and preserving large differences.
    Bioinformatics. https://doi.org/10.1093/bioinformatics/bty895
```

```
found results columns, replacing these

using 'apeglm' for LFC shrinkage. If used in published research, please cite:
    Zhu, A., Ibrahim, J.G., Love, M.I. (2018) Heavy-tailed prior distributions for
    sequence count data: removing the noise and preserving large differences.
    Bioinformatics. https://doi.org/10.1093/bioinformatics/bty895
using 'apeglm' for LFC shrinkage. If used in published research, please cite:
    Zhu, A., Ibrahim, J.G., Love, M.I. (2018) Heavy-tailed prior distributions for
    sequence count data: removing the noise and preserving large differences.
    Bioinformatics. https://doi.org/10.1093/bioinformatics/bty895
using 'apeglm' for LFC shrinkage. If used in published research, please cite:
    Zhu, A., Ibrahim, J.G., Love, M.I. (2018) Heavy-tailed prior distributions for
    sequence count data: removing the noise and preserving large differences.
    Bioinformatics. https://doi.org/10.1093/bioinformatics/bty895

found results columns, replacing these

1 rows did not converge in beta, labelled in mcols(object)$betaConv. Use larger maxit argumen

using 'apeglm' for LFC shrinkage. If used in published research, please cite:
    Zhu, A., Ibrahim, J.G., Love, M.I. (2018) Heavy-tailed prior distributions for
    sequence count data: removing the noise and preserving large differences.
    Bioinformatics. https://doi.org/10.1093/bioinformatics/bty895
using 'apeglm' for LFC shrinkage. If used in published research, please cite:
    Zhu, A., Ibrahim, J.G., Love, M.I. (2018) Heavy-tailed prior distributions for
    sequence count data: removing the noise and preserving large differences.
    Bioinformatics. https://doi.org/10.1093/bioinformatics/bty895
using 'apeglm' for LFC shrinkage. If used in published research, please cite:
    Zhu, A., Ibrahim, J.G., Love, M.I. (2018) Heavy-tailed prior distributions for
    sequence count data: removing the noise and preserving large differences.
    Bioinformatics. https://doi.org/10.1093/bioinformatics/bty895
```

```
group <- levels(as.factor(dds.sva$Pneumonia.type.and.evolution))
set_sva <- compare_reciprocal_contrasts(group,folder)
```

```
[1] "No overlap of regulated genes using different reference level for Mild and Intermediate"
```

```
saveRDS(set_sva,"covid_sva.rds")
```

Print resized diagnostic stacked bar chart for publication:

```r
make_diagnostic(set_sva)
```

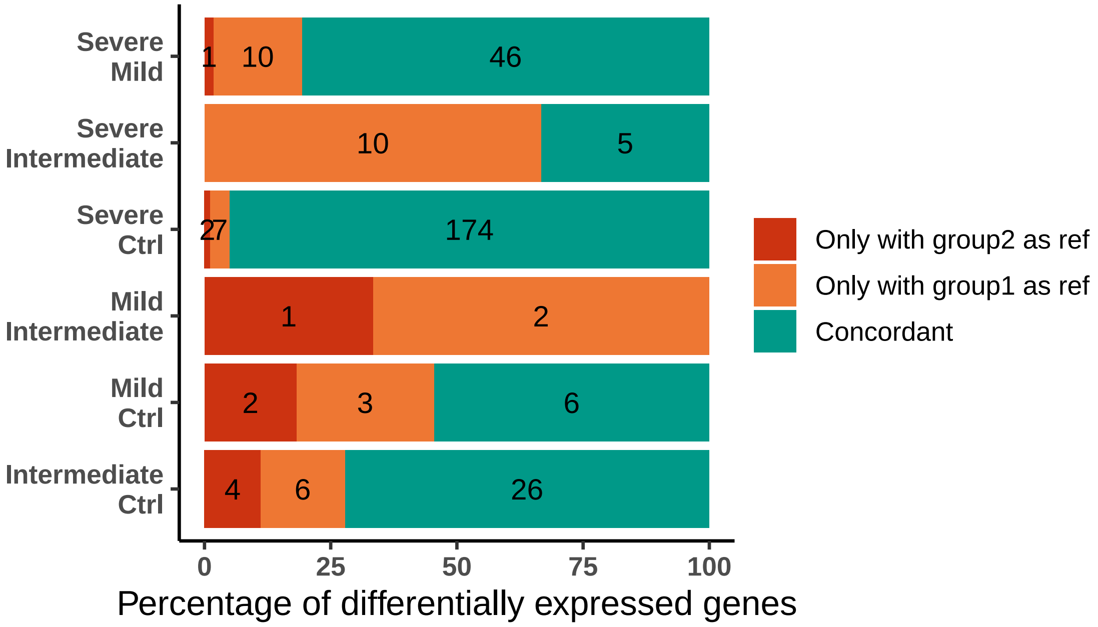

From the diagnostic plots (both here and reproduced in the three results folders in pdf files titled 'diagnostic plots for reciprocal contrasts'), we can see that without prefiltering this data, some reciprocal contrasts had very poor agreement. Some contrasts show much improved concordance using different reference levels when we use the edgeR prefilter for low expression genes. For others prefiltering does not help improve concordance much. In contrast, *sva* did not result in improved concordance in this dataset.

Question: do the discordant genes have lower expression levels than the concordant genes?

To help answer this, we examine a couple of contrasts with relatively large numbers of discordant genes (i.e. genes which are found to be DEGs only when using a particular group as the reference level). We retrieve details of the DEGs from the results output by compare_reciprocal_contrasts() above, using the unfiltered dds, and use the baseMean to provide an indication of gene expression levels. The baseMean is the average of the normalised counts over all samples in the dds.

Contrast Severe and Ctrl:

```
Severe_Ctrl <- set$Severe.Ctrl
# Check understanding that baseMean is the same in Against_Ctrl and
# Against_Severe reciprocal contrasts, but it is not reported in the results if
# the gene isn't a DEG (hence NAs)
any(is.na(Severe_Ctrl$baseMeanAgainst_Ctrl))
```

```
[1] TRUE
```

```
any(is.na(Severe_Ctrl$baseMeanAgainst_Severe))
```

```
[1] TRUE
```

```
test <- Severe_Ctrl %>% filter(!is.na(Severe_Ctrl$baseMeanAgainst_Severe) &
                                 !is.na(Severe_Ctrl$baseMeanAgainst_Ctrl))
any(test$baseMeanAgainst_Ctrl != test$baseMeanAgainst_Severe)
```

```
[1] FALSE
```

```
# This understanding was correct so we take the baseMean from either contrast:
Severe_Ctrl_neat <- Severe_Ctrl %>%
  mutate(baseMean = if_else(!is.na(baseMeanAgainst_Ctrl),
                            baseMeanAgainst_Ctrl,baseMeanAgainst_Severe),
         partition_display=str_replace_all(partition,
                                           c("group2ref" = "Against Ctrl",
                                             "group1ref" = "Against Severe",
                                             "concordant" = "Concordant"))) %>%
  select(name,baseMean,partition_display)
my_comparisons <- list( c("Against Ctrl","Against Severe"),
                        c("Against Ctrl", "Concordant"),
                        c("Against Severe","Concordant") )

p <- ggplot(Severe_Ctrl_neat, aes(x = partition_display, y = baseMean)) +
  geom_boxplot() +
  geom_point(position = position_jitter(), alpha = 0.25) +
  scale_y_continuous(trans='log10') +
  labs(y = "mean of normalised\ncounts (log10)", x = element_blank()) +
  stat_compare_means(comparisons = my_comparisons,
```

```
                          label = "p.signif",
                          size = 4,
                          label.y = c(4.8,5.2,5.7),
                          vjust= 0.1) +
  stat_compare_means(label.x = 0.8,label.y = 6.2, size = 4) +
  theme_classic(base_size = 16)
p
```

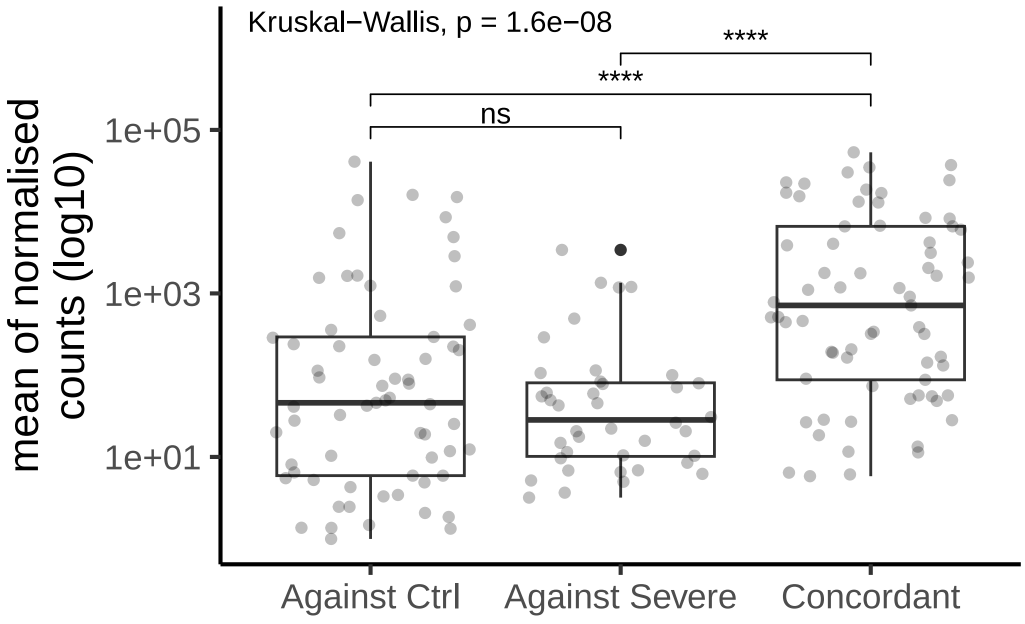

Contrast Severe and Mild:

```
Severe_Mild <- set$Severe.Mild

Severe_Mild_neat <- Severe_Mild %>%
  mutate(baseMean = if_else(!is.na(baseMeanAgainst_Mild),
                            baseMeanAgainst_Mild,baseMeanAgainst_Severe),
         partition_display=str_replace_all(partition,
                                           c("group2ref" = "Against Mild",
                                             "group1ref" = "Against Severe",
                                             "concordant" = "Concordant"))) %>%
  select(name,baseMean,partition_display)
```

```r
my_comparisons <- list( c("Against Mild","Against Severe"),
                        c("Against Mild", "Concordant"),
                        c("Against Severe","Concordant") )

p <- ggplot(Severe_Mild_neat, aes(x = partition_display, y = baseMean)) +
  geom_boxplot() +
  geom_point(position = position_jitter(), alpha = 0.25) +
  scale_y_continuous(trans='log10') +
  scale_x_discrete(labels=c("concordant" = "Concordant")) +
  labs(y = "mean of normalised\ncounts (log10)", x = element_blank()) +
  stat_compare_means(comparisons = my_comparisons,
                     label = "p.signif", size = 4,
                     label.y = c(4.4,4.8,5.3),
                     vjust= 0.1) +
  stat_compare_means(label.x = 0.8,label.y = 6, size = 4) +
  theme_classic(base_size = 16)
p
```

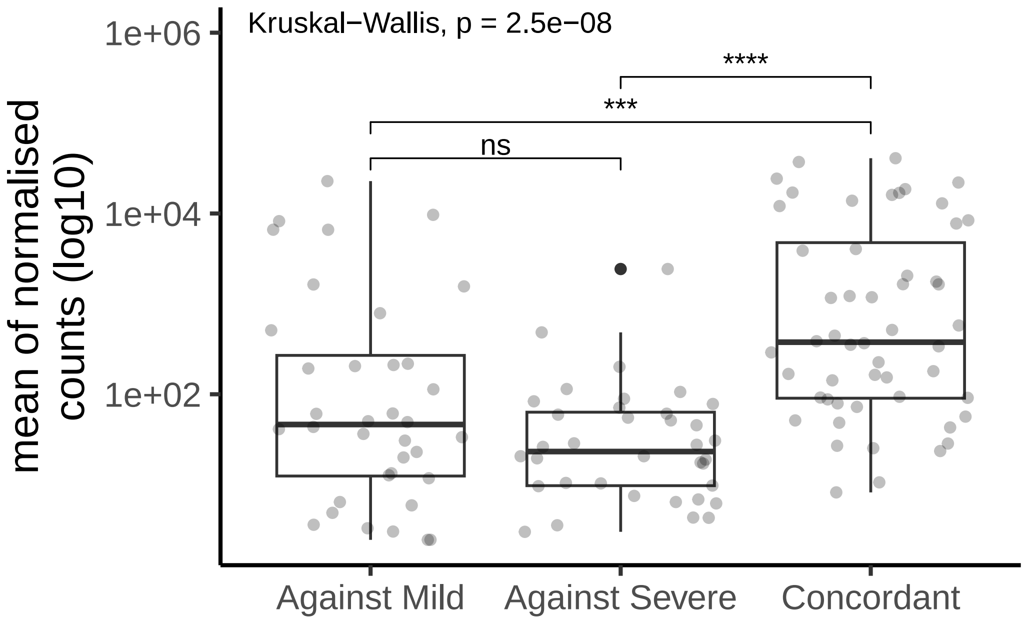

For both contrasts, we see that the concordant DEGs had significantly higher average normalised counts than DEGs found only using a particular reference level.

Question: How does this compare to edgeR? Are the discordant DEGs found to be DEGs with edgeR? Are the concordant DEGs also found with edgeR?

edgeR is another well-regarded tool for differential expression analysis. Filtering the data to eliminate low count genes is an important part of the standard edgeR workflow, and edgeR includes a specialised function to help make the filtering more data-dependent and sophisticated (which is why we borrowed it to test prefiltering of the dds, above). While edgeR does adjust the raw log fold change values using a "prior count" strategy, this is not intended to replace filtration. Here (and in the associated Quartos) we ran edgeR as recommended and with default parameters and did not observe discordant results for reciprocal contrasts. Therefore, after checking this is true, we compare two sets of DESeq2 results for a reciprocal contrast to the only set of edgeR DEGs.

Run edgeR

```
folder <- "edgeR"
dir.create(folder)
```

First, severe v ctrl

```
group <- metadata$Pneumonia.type.and.evolution
y <- DGEList(counts=mat,group=group)
dim(y$counts)
```

```
[1] 30633   159
```

```
keep <- filterByExpr(y)
y <- y[keep,,keep.lib.sizes=FALSE]
dim(y$counts)
```

```
[1] 18201   159
```

```
y <- normLibSizes(y)

design <- model.matrix(~group)
rownames(design) <- colnames(y)

y <- estimateDisp(y, design)
fit <- glmQLFit(y,design)
```

```r
# coef = 4 is Severe vs Ctrl
qlf <- glmQLFTest(fit,coef=4)
result_SeverevsCtrl <- topTags(qlf, n=nrow(y))
# check for missing adjusted p-values
any(is.na(as.data.frame(result_SeverevsCtrl)$FDR))
```

```
[1] FALSE
```

```r
# Adapt mirrorCheck's (private) plot_Volcano() to work for edgeR results
plot_Volcano_edgeR <- function(r,title, rowname2symbol = NULL,
                         p.cutoff = 0.1, fc.cutoff = 2, top.n = 30) {
  padj <- as.data.frame(r) %>%
    dplyr::filter(!is.na(FDR)) %>%
    dplyr::mutate(log10padj = -log10(FDR),
                  diff.expressed = dplyr::case_when(
                    logFC > fc.cutoff & FDR < p.cutoff ~ "UP",
                    logFC < -fc.cutoff & FDR < p.cutoff ~ "DOWN",
                    .default = "NO")) %>%
    tibble::rownames_to_column("rowname")
  if (is.null(rowname2symbol)) {
    padj <- padj %>%
      dplyr::mutate(g_symbol = rowname)
  } else {
    padj <- padj  %>%
      dplyr::left_join(rowname2symbol)
  }
  diff.ex <- padj %>% dplyr::filter(diff.expressed != "NO")

  sums <- table(diff.ex$diff.expressed)
  maxy <- max(padj$log10padj)
  minx <- min(padj$logFC)
  maxx <- max(padj$logFC)
  padj$labels <- ifelse(padj$rowname %in% head(diff.ex[order(diff.ex$FDR),
                                                        "rowname"], top.n),
                        padj$g_symbol, NA)
  group.colors <- c(DOWN = '#CC3311',NO = "grey",UP = '#009988')
  group.labels <- c(DOWN = "Downregulated",
                    NO = "Not significant",
                    UP = "Upregulated")

  p <- ggplot2::ggplot(padj, ggplot2::aes(x = logFC,
```

```
                                          y = log10padj,
                                          col = diff.expressed,
                                          label = labels)) +
    ggplot2::theme_classic(base_size = 14) +
    ggplot2::labs(title = title,
                  x = expression("log"[2]*"FC"),
                  y = expression("-log"[10]*"adj-pvalue")) +
    ggplot2::theme(legend.title = element_blank()) +
    ggplot2::geom_vline(xintercept = c(-fc.cutoff, fc.cutoff),
                        col = "gray", linetype = 'dashed') +
    ggplot2::geom_hline(yintercept = -log10(p.cutoff),
                        col = "gray", linetype = 'dashed') +
    ggplot2::geom_point(size = 1.5) +
    ggplot2::scale_color_manual(values = group.colors,
                                labels = group.labels) +
    ggrepel::geom_text_repel(max.overlaps = Inf, size = 3,
                             color = "black", na.rm = T) +
    ggplot2::annotate("text",x = minx + 2, y = maxy - 1,
                      label = paste("DOWN",sums["DOWN"]),size = 4.5) +
    ggplot2::annotate("text",x = maxx - 1, y = maxy - 1,
                      label = paste("UP",sums["UP"]), size = 4.5)
}

p <- plot_Volcano_edgeR(result_SeverevsCtrl, "Severe_vs_Ctrl edgeR",
                        p.cutoff = 0.01,
                        top.n = 0)
p
```

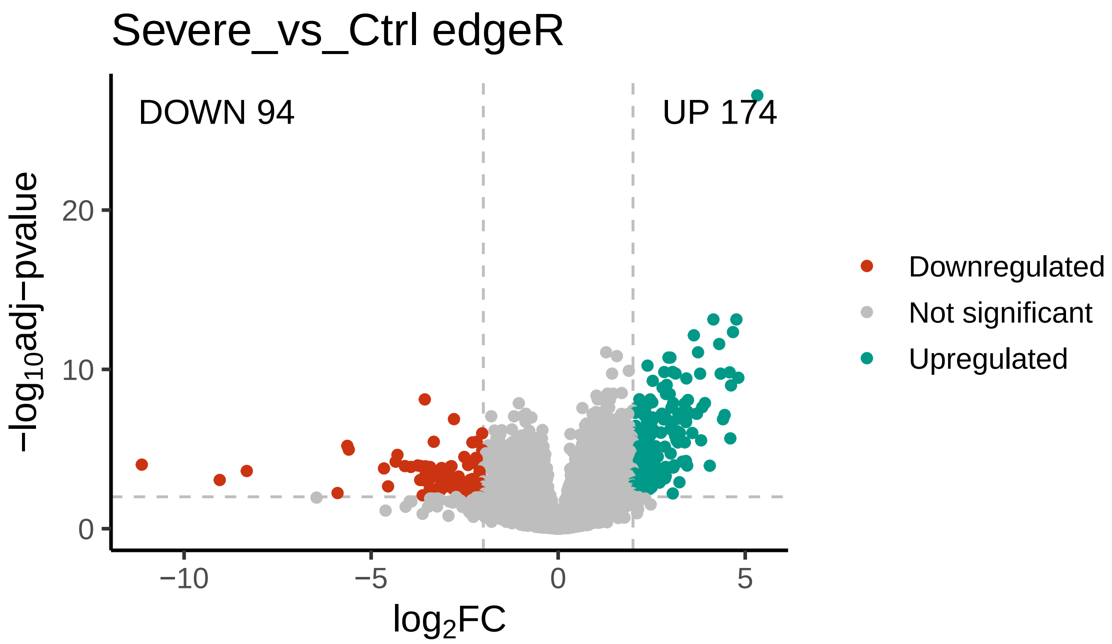

Second, relevel for comparison to Severe

```
group <- relevel(group, ref = "Severe")
levels(group)
```

```
[1] "Severe"       "Ctrl"         "Intermediate" "Mild"
```

```
y <- DGEList(counts=mat,group=group)
dim(y$counts)
```

```
[1] 30633   159
```

```
keep <- filterByExpr(y)
y <- y[keep,,keep.lib.sizes=FALSE]
dim(y$counts)
```

```
[1] 18201   159
```

```r
y <- normLibSizes(y)

design <- model.matrix(~group)
rownames(design) <- colnames(y)

y <- estimateDisp(y, design)
fit <- glmQLFit(y,design)
# coef = 2 is Ctrl vs Severe
qlf2 <- glmQLFTest(fit,coef=2)
result_CtrlvsSevere <- topTags(qlf2, n=nrow(y))
# check for missing adjusted p-values
any(is.na(as.data.frame(result_CtrlvsSevere)$FDR))
```

```
[1] FALSE
```

```r
p <- plot_Volcano_edgeR(result_CtrlvsSevere, "Ctrl_vs_Severe edgeR",
                        p.cutoff = 0.01,
                        top.n = 0)
p
```

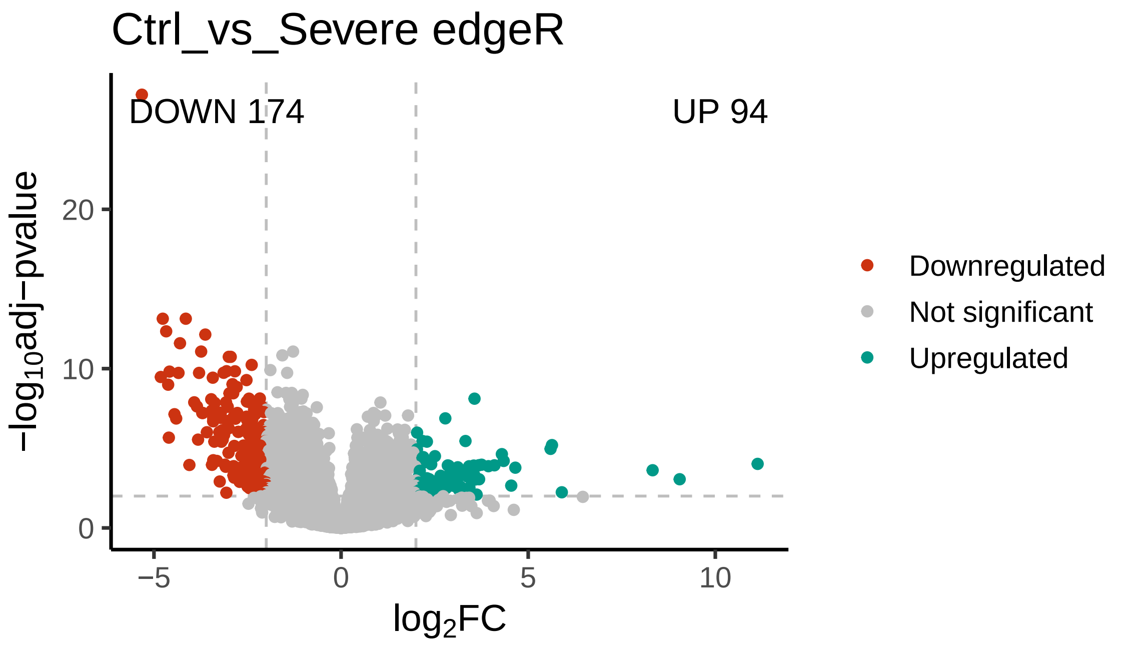

```r
# Write results tables
write.csv(as.data.frame(result_CtrlvsSevere),
          file = file.path(folder,"CtrlvSevere.csv"))
write.csv(as.data.frame(result_SeverevsCtrl),
          file = file.path(folder,"SeverevCtrl.csv"))
```

Check: these results are perfectly mirrored so they give the same set of DEGs at FDR < 0.01 and logFC > 2 | logFC < 2

```r
result_CtrlvsSevere_df <- as.data.frame(result_CtrlvsSevere)
result_SeverevsCtrl_df <- as.data.frame(result_SeverevsCtrl)

deg_CvS <- result_CtrlvsSevere_df %>%
  filter(FDR < 0.01 & (logFC < -2 | logFC > 2)) %>%
  rownames_to_column("name") %>%
  pull(name)
str(deg_CvS)
```

```
 chr [1:268] "IGHV3-19" "USP41" "TRDV3" "LINC02574" "LARP1P1" "ERICH3" ...
```

```r
deg_SvC <- result_SeverevsCtrl_df %>%
  filter(FDR < 0.01 & (logFC < -2 | logFC > 2)) %>%
  rownames_to_column("name") %>%
  pull(name)
all(deg_CvS == deg_SvC)
```

```
[1] TRUE
```

```r
edger_DEG <- deg_CvS
```

DESeq2 DEGs compared to edgeR DEGs - without prefiltering dds

```r
deseq_CvS <- read.csv(file.path("DESeq_noclean","Ctrl_vs_Severe_DG.csv"))
table(deseq_CvS$padj < 0.01 &
        (deseq_CvS$log2FoldChange < -2 | deseq_CvS$log2FoldChange > 2))
```

```
TRUE
 109
```

```
deseq_SvC <- read.csv(file.path("DESeq_noclean","Severe_vs_Ctrl_DG.csv"))
table(deseq_SvC$padj < 0.01 &
        (deseq_SvC$log2FoldChange < -2 | deseq_SvC$log2FoldChange > 2))
```

```
TRUE
 134
```

```
deseq_CvS_deg <- deseq_CvS %>% pull(name)
deseq_SvC_deg <- deseq_SvC %>% pull(name)

#unfiltered upset
list_with_unfiltdeseq <- list(edgeR = edger_DEG,
                              DESeq2_vCtrl = deseq_SvC_deg,
                              DESeq2_vSevere = deseq_CvS_deg)
str(list_with_unfiltdeseq)
```

```
List of 3
 $ edgeR         : chr [1:268] "IGHV3-19" "USP41" "TRDV3" "LINC02574" ...
 $ DESeq2_vCtrl  : chr [1:134] "PID1" "ZNF684" "UBQLNL" "NRIR" ...
 $ DESeq2_vSevere: chr [1:109] "RPL36A-HNRNPH2" "CACNA2D3" "PID1" "TSPEAR" ...
```

```
# the following function was sourced from github UpsetR issue 85, solution
# provided by 'docmanny' in September 2017
newFromList <- function (input) {
  # Same as original fromList()...
  elements <- unique(unlist(input))
  data <- unlist(lapply(input, function(x) {
    x <- as.vector(match(elements, x))
  }))
  data[is.na(data)] <- as.integer(0)
  data[data != 0] <- as.integer(1)
  data <- data.frame(matrix(data, ncol = length(input), byrow = F))
  data <- data[which(rowSums(data) != 0), ]
  names(data) <- names(input)
  # ... Except now it conserves your original value names!
  row.names(data) <- elements
  return(data)
}
```

```
for_upset_unfilt <- newFromList(list_with_unfiltdeseq) %>%
  mutate(concordant = if_else(DESeq2_vCtrl == 1 & DESeq2_vSevere == 1, T, F))

upset_unfilt <- UpSetR::upset(for_upset_unfilt,
                              sets = c("edgeR",
                                       "DESeq2_vCtrl",
                                       "DESeq2_vSevere"),
                              nintersects = NA,
                              mainbar.y.label = "Number of DEGs",
                              sets.x.label = "Results set",
                              text.scale = 2,
                              empty.intersections = T,
                              keep.order = T,
                              mb.ratio = c(0.6, 0.4))
upset_unfilt
```

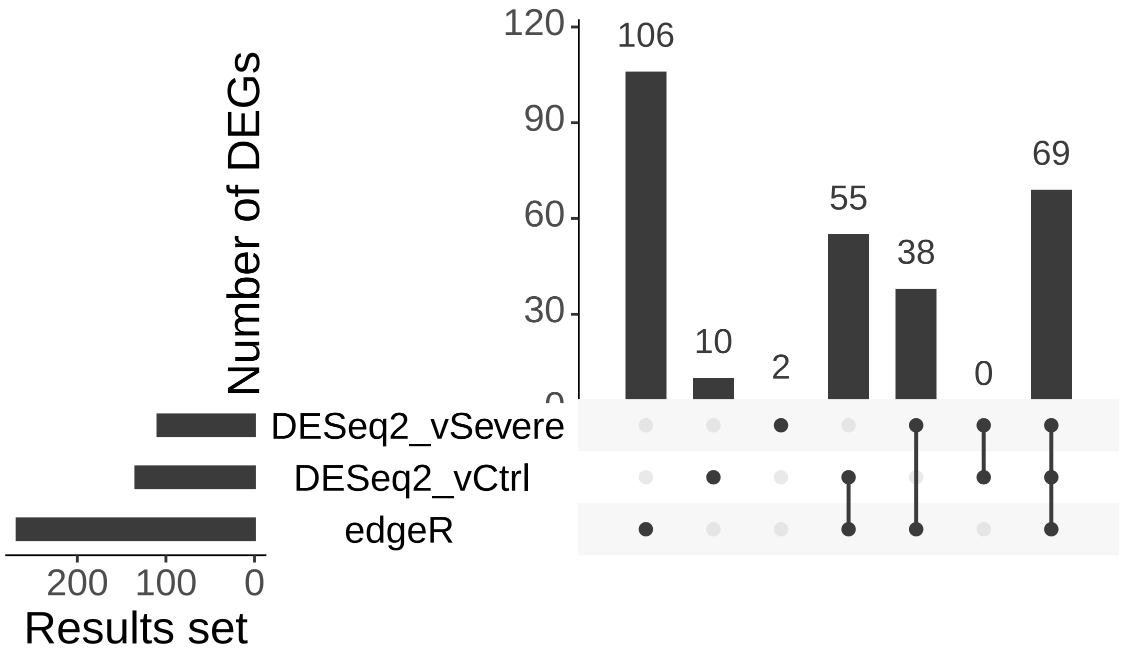

DESeq2 DEGs compared to edgeR DEGs - prefiltering dds

```
deseq_CvS_filt <- read.csv(file.path("DESeq_prefilt","Ctrl_vs_Severe_DG.csv"))
table(deseq_CvS_filt$padj < 0.01 &
        (deseq_CvS_filt$log2FoldChange<-2 | deseq_CvS_filt$log2FoldChange > 2))
```

```
TRUE
 164
```

```
deseq_SvC_filt <- read.csv(file.path("DESeq_prefilt","Severe_vs_Ctrl_DG.csv"))
table(deseq_SvC_filt$padj < 0.01 &
        (deseq_SvC_filt$log2FoldChange<-2 | deseq_SvC_filt$log2FoldChange > 2))
```

```
TRUE
 164
```

```
deseq_CvS_deg_filt <- deseq_CvS_filt %>% pull(name)
deseq_SvC_deg_filt <- deseq_SvC_filt %>% pull(name)
all(deseq_CvS_deg_filt == deseq_SvC_deg_filt)
```

```
[1] FALSE
```

```
#filtered upset
list_with_filtdeseq <- list(edgeR = edger_DEG,
                            DESeq2_vCtrl = deseq_SvC_deg_filt,
                            DESeq2_vSevere = deseq_CvS_deg_filt)
str(list_with_filtdeseq)
```

```
List of 3
 $ edgeR         : chr [1:268] "IGHV3-19" "USP41" "TRDV3" "LINC02574" ...
 $ DESeq2_vCtrl  : chr [1:164] "CACNA2D3" "PID1" "TSPEAR" "LYPD2" ...
 $ DESeq2_vSevere: chr [1:164] "RPL36A-HNRNPH2" "CACNA2D3" "PID1" "TSPEAR" ...
```

```
for_upset_filt <- newFromList(list_with_filtdeseq) %>%
  mutate(concordant = if_else(DESeq2_vCtrl == 1 & DESeq2_vSevere == 1, T, F))

upset_filt <- UpSetR::upset(for_upset_filt, sets = c("edgeR",
                                                      "DESeq2_vCtrl",
                                                      "DESeq2_vSevere"),
                            nintersects = NA,
                            mainbar.y.label = "Number of DEGs",
                            sets.x.label = "Results set",
                            text.scale = 2,
                            empty.intersections = T,
```

```
                                    keep.order = T,
                                    mb.ratio = c(0.6, 0.4))
upset_filt
```

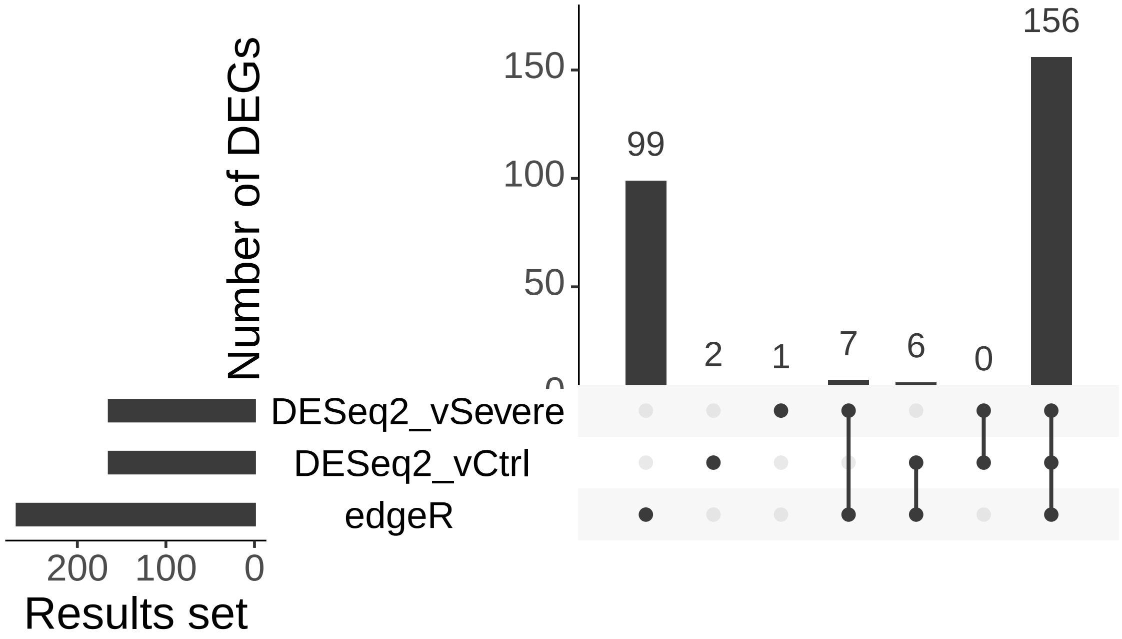

DESeq2 DEGs from prefiltered and non-prefiltered dds compared to edgeR DEGs on the same UpSet plot, with gene expression levels

```
list_with_bothdeseq <- list(edgeR = edger_DEG,
                            vCtrl = deseq_SvC_deg,
                            vSevere = deseq_CvS_deg,
                            vCtrl_filt = deseq_SvC_deg_filt,
                            vSevere_filt = deseq_CvS_deg_filt)
str(list_with_bothdeseq)
```

```
List of 5
 $ edgeR       : chr [1:268] "IGHV3-19" "USP41" "TRDV3" "LINC02574" ...
 $ vCtrl       : chr [1:134] "PID1" "ZNF684" "UBQLNL" "NRIR" ...
 $ vSevere     : chr [1:109] "RPL36A-HNRNPH2" "CACNA2D3" "PID1" "TSPEAR" ...
 $ vCtrl_filt  : chr [1:164] "CACNA2D3" "PID1" "TSPEAR" "LYPD2" ...
 $ vSevere_filt: chr [1:164] "RPL36A-HNRNPH2" "CACNA2D3" "PID1" "TSPEAR" ...
```

```r
for_upset_both <- newFromList(list_with_bothdeseq) %>%
  mutate(Concordant = if_else((vCtrl == 1 & vSevere == 1) |
                               (vCtrl_filt == 1 & vSevere_filt == 1),
                             T, F))
write.csv(for_upset_both,
              file = file.path(folder,"DESeq_edger_DEG_intersections.csv"),
              na="")

#Get DESeq2 results to easily extract mean normalised counts (baseMeans) for
#genes across samples
dds <- DESeq(dds)
```

```
using pre-existing size factors

estimating dispersions

found already estimated dispersions, replacing these

gene-wise dispersion estimates

mean-dispersion relationship

final dispersion estimates

fitting model and testing

-- replacing outliers and refitting for 388 genes
-- DESeq argument 'minReplicatesForReplace' = 7
-- original counts are preserved in counts(dds)

estimating dispersions

fitting model and testing
```

```r
res <- results(dds)
baseMeans <- as.data.frame(res) %>%
  rownames_to_column("name") %>%
  select(name,baseMean)
head(baseMeans)
```

```
       name     baseMean
1      A1BG 149.66992430
2 A1BG-AS1 324.74736064
3      A1CF   0.08445563
4       A2M   0.26743510
5   A2M-AS1  32.74269919
6     A2ML1   4.26052253
```

```
withNames <- for_upset_both %>% rownames_to_column("name")
forComplex <- withNames %>% left_join(baseMeans)
```

```
Joining with `by = join_by(name)`
```

```
head(forComplex)
```

```
       name edgeR vCtrl vSevere vCtrl_filt vSevere_filt Concordant  baseMean
1  IGHV3-19     1     0       0          0            0      FALSE  2.552801
2     USP41     1     1       0          1            1       TRUE  6.463873
3     TRDV3     1     1       1          1            1       TRUE 13.361861
4 LINC02574     1     1       0          1            1       TRUE  8.080137
5   LARP1P1     1     1       1          1            1       TRUE  6.420679
6    ERICH3     1     1       0          1            1       TRUE 25.312889
```

```
my_comparisons <- list( c(4,11), c(4,12),c(4,13))
ComplexUpset::upset(forComplex,c("edgeR",
                                 "vCtrl",
                                 "vSevere",
                                 "vCtrl_filt",
                                 "vSevere_filt"),
                    name = element_blank(),
                    sort_intersections_by=c("degree","ratio"),
                    sort_intersections = "ascending",
                    sort_sets=F,
                    annotations = list(
                      "log10 mean of\nnormalised counts"=list(
                        aes=aes(x=intersection, y=baseMean),
                        geom=list(geom_boxplot(),
                                  scale_y_continuous(trans='log10'),
                                  expand_limits(y = 1000000000),
                                  stat_compare_means(comparisons=my_comparisons,
```

```
                                                    label = "p.signif",
                                                    size = 4,
                                                    label.y = c(5,6.1,7.2),
                                                    vjust=0.1),
                                   theme(axis.text.y = element_text(size = 10),
                                         axis.title.y=element_text(face="bold"))
                                   )
                         )
                     ),
                     base_annotations=list(
                       'DEGs\nper intersection'=intersection_size(
                         mapping=aes(fill=Concordant)) +
                         scale_fill_manual(values =c("TRUE" = "#009988",
                                                   "FALSE" = "black")) +
                         theme(axis.text.y = element_text(size = 10),
                               axis.title.y = element_text(face="bold"))
                         ),
                     set_sizes=(
                       upset_set_size(position='right')
                       + ylab("DEGs per set") +
                         xlab(element_blank())
                     ),
                     themes=upset_modify_themes(
                       list('intersections_matrix'=theme(
                         axis.text.y=element_text(size=12)
                         )
                       )
                     ),
                     width_ratio=0.2,
                     height_ratio=0.65,
                     guides='over'
                   ) & theme(legend.margin=margin(margin(t = 180, r = 0,
                                                          b = 0, l = 0,
                                                          unit = "pt")))
```

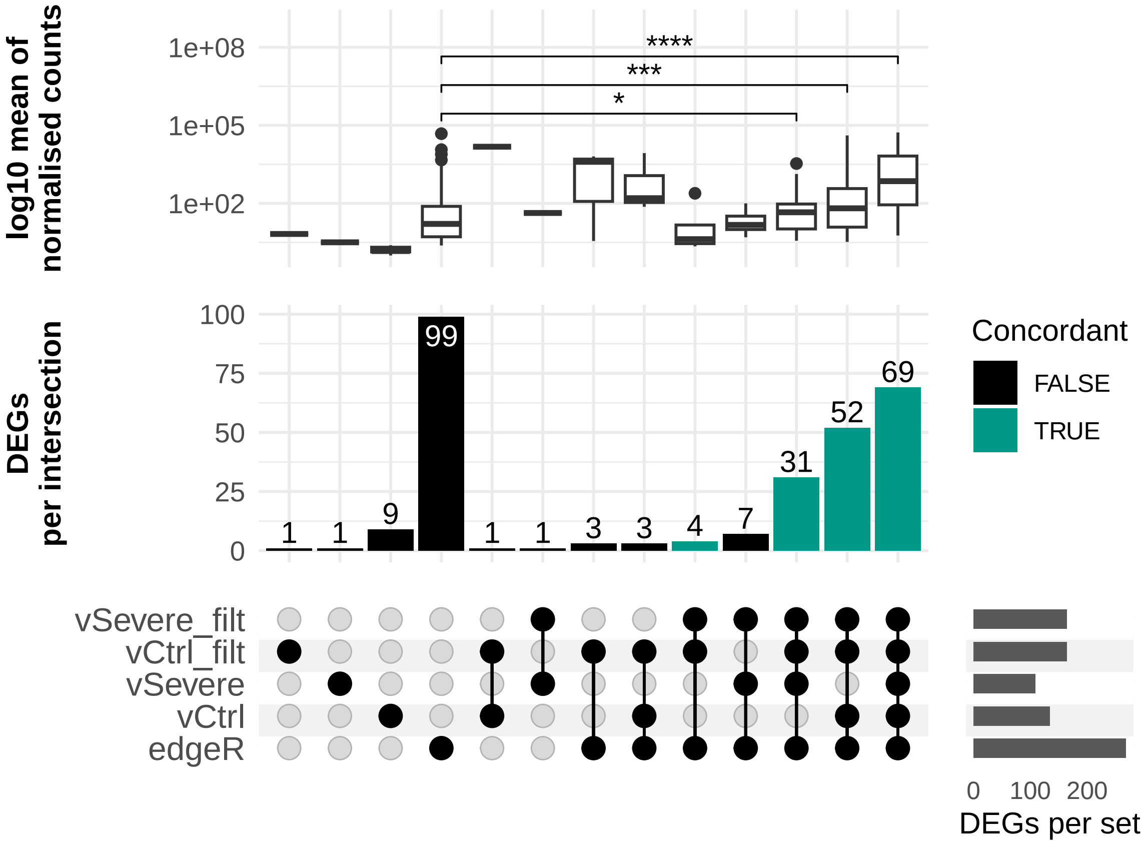

Question: What impact does discordance have on further analyses such as pathway analysis or gene set analysis?

Gene Set Enrichment Analysis (GSEA) uses expression data from the whole gene list to find enriched gene sets, without using thresholds to determine DEGs. Genes are ranked, then scored based on differential expression of defined gene sets. The GSEA software from the Broad Institute can perform DGEA and ranking using the phenotype permutation approach when there are sufficient sample numbers per condition (phenotype). However, many studies need to use the gene set permutation approach, where DGEA is done first, then GSEA performed on a preranked gene list. The logFC can be used to rank the genes (e.g. Calura et al.; others recommend ranking by `sign(log2FoldChange) * -log10(pvalue)` e.g. Reimand et al.). Since mirrorCheck discordance is more likely to affect ranking when using the logFC, we'll try that here.

Preparing for GSEA

We'll prepare 4 ranked lists from the Severe-Ctrl contrast DGEA; Severe_v_Ctrl and Ctrl_v_Severe from the filtered dataset (good concordance) and Severe_v_Ctrl and Ctrl_v_Severe from the dataset without clean-up (poor concordance).

```
make_rankedGenes <- function(fn, folder = ".", outdir = ".") {
  res <- read.csv(file.path(folder,fn)) %>%
    mutate(rank = log2FoldChange) %>%
    filter(!is.na(rank)) %>%
    dplyr::rename(GeneName = name) %>%
    filter(!is.na(GeneName) & GeneName != "") %>%
    dplyr::select(GeneName,rank)
  if(length(unique(res$GeneName)) != length(res$GeneName)) {
    stop("Problem: There are duplicate GeneNames in the list")
  }
  res <- res %>%
    arrange(desc(rank)) %>%
    group_by(rank) %>%
    arrange(GeneName, .by_group = T) %>%
    mutate(modifier = row_number() * 0.000000001) %>%
    ungroup() %>%
    mutate(rank_mod = rank + modifier) %>%
    arrange(desc(modifier)) %>%
    dplyr::select(-c(rank,modifier)) %>%
    dplyr::rename(rank = rank_mod)
  descriptor <- sub("DESeq_","",folder)
  out <- paste0("ranked_",descriptor,"_",sub("_all\\.csv","\\.rnk",fn))
  write.table(res,file = file.path(outdir,out),
              row.names = F, quote = F, sep = "\t")
}
chosen_files <- c("Ctrl_vs_Severe_all.csv","Severe_vs_Ctrl_all.csv")

outdir = "GSEA"
dir.create(outdir)
lapply(chosen_files,make_rankedGenes, folder = "DESeq_prefilt", outdir = outdir)
```

```
[[1]]
NULL

[[2]]
NULL
```

```
lapply(chosen_files,make_rankedGenes, folder = "DESeq_noclean", outdir = outdir)
```

```
[[1]]
NULL

[[2]]
NULL
```

We then run GSEA using the Broad Institute's cli script. (If you are rendering this Quarto on a OS other than Linux, you may need to delete this code chunk.)

```
#!/bin/bash

cd GSEA/
filenames=$(find "." -name "*.rnk" -exec basename {} \; | sort)

for f in $filenames; do
    echo $f
    tmp=${f#ranked_}
    label=${tmp%\.rnk}
#replace with the path to your copy of GSEA. If the command does not work,
#check it within the GSEA GUI as per the software instructions.
    /home/kate/Downloads/GSEA_Linux_4.3.3/gsea-cli.sh GSEAPreranked -gmx \
    ftp.broadinstitute.org://pub/gsea/msigdb/human/gene_sets/h.all.v2024.1.Hs.symbols.gmt \
    -collapse No_Collapse -mode Abs_max_of_probes -norm meandiv -nperm 10000 \
    -rnd_seed timestamp -rnk $f -scoring_scheme weighted -rpt_label $label \
    -create_svgs false -include_only_symbols true -make_sets true \
    -plot_top_x 20 -set_max 500 -set_min 15 -zip_report false -out GSEA_output
done
```

Finally, we plot the differences in identified pathways (identity and significance). Note that some pathways had a calculated FDR-q-value of 0, meaning that when we performed a -10log(FDR) transformation for ease of visualisation, these data points became `Inf`, which is unplottable. We converted `Inf` to a value greater than the other datapoints and renamed the `breaks` so that this value shows up as the infinity symbol.

```
getwd()
```

```
[1] "/home/kate/my_shared_drive/Lab members/Kate/mirrorCheck_publication/working_for_review_
```

```r
fns <- list.files(path="GSEA/GSEA_output",pattern = "gsea_report.*tsv",
                  recursive = T)
fn.info <- data.frame(fns = fns) %>%
  mutate(front = sub("\\..*","",fns),
         end = if_else(grepl("neg",fns),"neg","pos"),
         name = paste(front,end,sep = "_"))
all.gsea <- lapply(file.path("GSEA/GSEA_output",fns),read.csv, sep = "\t")
names(all.gsea) <- fn.info$name
all.gsea <- lapply(all.gsea, function(x) x %>% select(NAME,FDR.q.val))
all.gsea <- bind_rows(all.gsea,.id = "fn")
all.gsea <- all.gsea %>% rowwise() %>%
  mutate(cleanup = sub("_.*","",fn),
         ref_group = if_else(grepl("vs_Ctrl",fn),"Ctrl","Severe"),
         up = case_when(grepl("vs_Ctrl",fn) && grepl("pos",fn) ~"Severe",
                        grepl("vs_Ctrl",fn) && grepl("neg",fn) ~"Ctrl",
                        grepl("vs_Severe",fn) && grepl("pos",fn) ~"Ctrl",
                        grepl("vs_Severe",fn) && grepl("neg",fn) ~"Severe"))
all.gsea.to.plot <- all.gsea %>%
  mutate(logfdr = -log10(FDR.q.val),
         logfdr = replace(logfdr, logfdr == Inf, 6))

print(all.gsea)
```

```
# A tibble: 200 x 6
# Rowwise:
   fn                          NAME             FDR.q.val cleanup ref_group up
   <chr>                       <chr>                <dbl> <chr>   <chr>     <chr>
 1 noclean_Ctrl_vs_Severe_neg HALLMARK_INTERF~    0.0512 noclean Severe    Seve~
 2 noclean_Ctrl_vs_Severe_neg HALLMARK_INTERF~    0.0381 noclean Severe    Seve~
 3 noclean_Ctrl_vs_Severe_neg HALLMARK_G2M_CH~    0.0922 noclean Severe    Seve~
 4 noclean_Ctrl_vs_Severe_neg HALLMARK_E2F_TA~    0.0709 noclean Severe    Seve~
 5 noclean_Ctrl_vs_Severe_neg HALLMARK_MTORC1~    0.420  noclean Severe    Seve~
 6 noclean_Ctrl_vs_Severe_neg HALLMARK_MITOTI~    0.354  noclean Severe    Seve~
 7 noclean_Ctrl_vs_Severe_neg HALLMARK_MYC_TA~    0.357  noclean Severe    Seve~
 8 noclean_Ctrl_vs_Severe_neg HALLMARK_DNA_RE~    0.335  noclean Severe    Seve~
 9 noclean_Ctrl_vs_Severe_neg HALLMARK_CHOLES~    0.797  noclean Severe    Seve~
10 noclean_Ctrl_vs_Severe_neg HALLMARK_ALLOGR~    0.873  noclean Severe    Seve~
# i 190 more rows
```

```r
p <- ggplot(all.gsea.to.plot, aes(x = ref_group,y = logfdr, fill = cleanup)) +
  geom_violin(scale = "width") +
```

```r
  geom_point(show.legend = F, colour = "gray",size = 0.8,
             position=position_jitterdodge(jitter.width = 0.1)) +
  facet_grid(~up, labeller = as_labeller(c(Ctrl = "Up in Ctrl",
                                           Severe = "Up in Severe")),
             scales = "free") +
  geom_hline(yintercept = -log10(0.05),
             col = "gray", linetype = 'dashed') +
  theme_classic() +
  ylab("-log10(FDR-q-value)") +
  xlab("group used as reference group") +
  scale_y_continuous(n.breaks = 7, labels=c(seq(0,5),expression(infinity))) +

  #coord_trans(y = neglogtransform) +
  scale_fill_discrete(labels = c(noclean = "no cleaning",
                                 prefilt = "prefiltered"),
                    type = c("noclean" = "darkgrey",
                               "prefilt" = "white")) +
  theme(legend.title = element_blank())
p
```

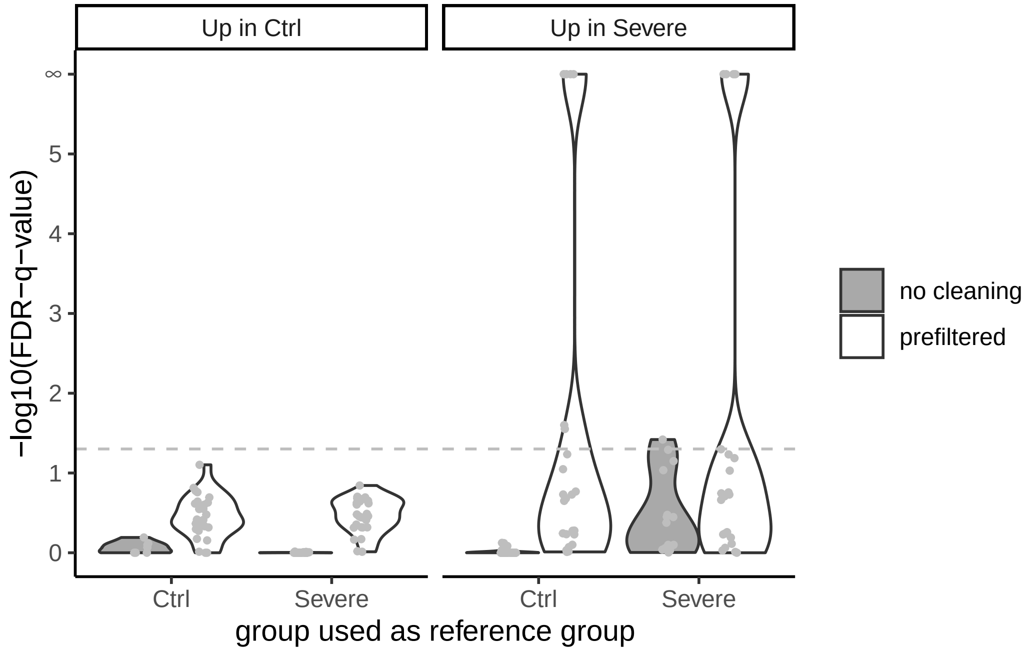

Without data clean-up, few or no pathways are confidently observed to be enriched (FDR Q value < 0.05), whereas in cleaned data, we see enriched pathways in the group with severe COVID. These include strong results for interferon signalling, which makes sense in the context

of a viral infection. Also, without cleaning the data, we observe inconsistent results when the analysis used different reference levels, whereas the results from the filtered data are similar regardless of the reference level. For the manuscript, we plot the “Up in Severe” data on its own:

```
simpler <- all.gsea.to.plot %>% filter(up == "Severe") %>%
  mutate(signif = if_else(logfdr > -log10(0.05), T,F))
#Make labels for significant pathways
pathway_labels <- simpler %>%
  filter(signif) %>%
  arrange(FDR.q.val) %>%
  select(NAME) %>%
  unique() %>%
  ungroup() %>%
  mutate(labels = LETTERS[row_number()])
simpler <- simpler %>%
  left_join(pathway_labels) %>%
  mutate(labels = if_else(!signif, NA, labels))
```

```
Joining with `by = join_by(NAME)`
```

```
p2 <- ggplot(simpler, aes(x = cleanup, y = logfdr, fill = cleanup)) +
  geom_violin(scale = "width") +
  geom_point(alpha = 0.7, show.legend = F) +
  geom_line(aes(group = NAME), linewidth = 0.2, colour = "gray") +
  theme_classic(base_size = 16) +
  theme(axis.ticks.x = element_blank(),
        axis.title.x = element_blank(),
        axis.text.x = element_blank(),
        legend.title = element_blank(),
        legend.position = "bottom") +
  facet_grid(~ref_group, labeller = as_labeller(c(Ctrl = "Ctrl is reference",
                                                   Severe = "Severe is reference")),
             scales = "free") +
  ggtitle("Enrichment in Severe (COVID)") +
  scale_fill_discrete(labels = c(noclean = "no cleaning", prefilt = "prefiltered"),
                      type = c(noclean = "darkgrey",
                               prefilt = "white")) +
  scale_y_continuous(n.breaks = 7, labels=c(seq(0,5),expression(infinity))) +
  geom_hline(yintercept = -log10(0.05),
             col = "gray", linetype = 'dashed') +
  geom_text_repel(aes(label = labels), na.rm = T,
```

```
                     min.segment.length = 10, color = "#009988",
                     fontface = "bold") +
  xlab(element_blank()) +
  ylab("-log10(FDR-q-value)")
p2
```

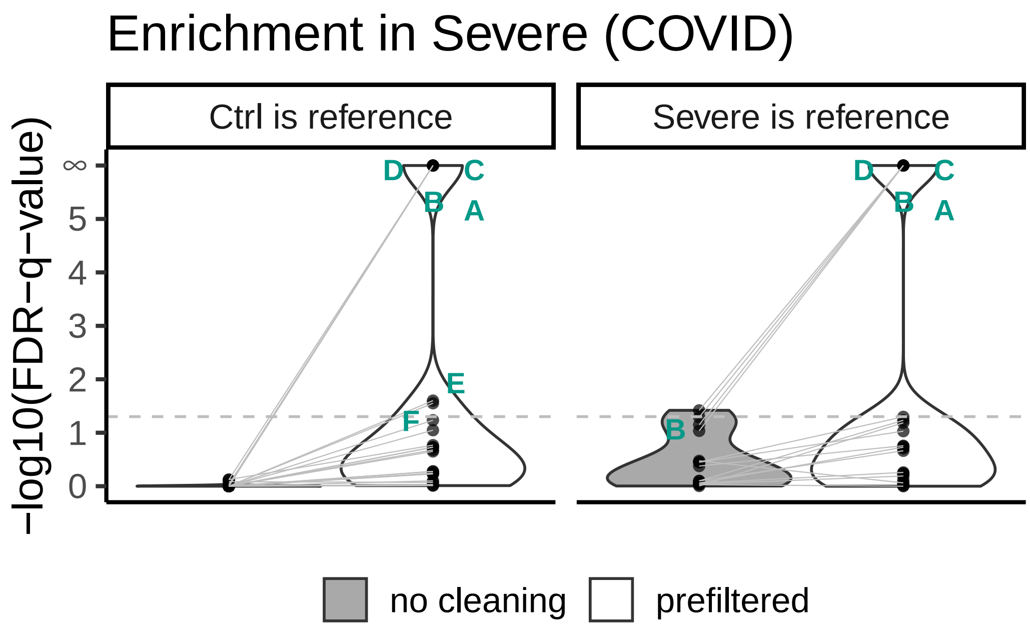


```
#print significant pathways
for_table <- pathway_labels %>% left_join(all.gsea, by = join_by(NAME)) %>%
  mutate(analysis = paste(cleanup,ref_group,sep = "_")) %>%
  select(NAME,FDR.q.val,analysis,labels) %>%
  pivot_wider(names_from = analysis, values_from = FDR.q.val) %>%
  relocate(labels)
for_table
```

```
# A tibble: 6 x 6
  labels NAME             noclean_Severe noclean_Ctrl prefilt_Severe prefilt_Ctrl
  <chr>  <chr>                     <dbl>        <dbl>          <dbl>        <dbl>
1 A      HALLMARK_INTER~          0.0512        1              0            0
2 B      HALLMARK_INTER~          0.0381        1              0            0
3 C      HALLMARK_G2M_C~          0.0922        0.929          0            0
4 D      HALLMARK_E2F_T~          0.0709        0.757          0            0
5 E      HALLMARK_SPERM~          0.992         1              0.0651       0.0251
```

```
6 F      HALLMARK_UNFOL~         0.797         1             0.0585        0.0281
```

```
write.csv(for_table, "significant_pathways.csv")
```

Session info and citations

Calura, E., & Martini, P. (2021). Summarizing RNA-Seq Data or Differentially Expressed Genes Using Gene Set, Network, or Pathway Analysis. Methods in Molecular Biology, 2284, 147–179. https://doi.org/10.1007/978-1-0716-1307-8_9/FIGURES/8

Love, M. I., Anders, S., Kim, V., & Huber, W. (2016). RNA-Seq workflow: gene-level exploratory analysis and differential expression. F1000Research 2016 4:1070, 4, 1070. https://doi.org/10.12688/f1000research.7035.2

Reimand, J., Isserlin, R., Voisin, V., Kucera, M., Tannus-Lopes, C., Rostamianfar, A., Wadi, L., Meyer, M., Wong, J., Xu, C., Merico, D., & Bader, G. D. (2019). Pathway enrichment analysis and visualization of omics data using g:Profiler, GSEA, Cytoscape and EnrichmentMap. *Nature Protocols*, *14*(2), 482–517. https://doi.org/10.1038/s41596-018-0103-9


```
citation("DESeq2")
```

```
To cite package 'DESeq2' in publications use:

  Love, M.I., Huber, W., Anders, S. Moderated estimation of fold change
  and dispersion for RNA-seq data with DESeq2 Genome Biology 15(12):550
  (2014)

A BibTeX entry for LaTeX users is

  @Article{,
    title = {Moderated estimation of fold change and dispersion for RNA-seq data with DESeq2
    author = {Michael I. Love and Wolfgang Huber and Simon Anders},
    year = {2014},
    journal = {Genome Biology},
    doi = {10.1186/s13059-014-0550-8},
    volume = {15},
    issue = {12},
    pages = {550},
  }
```

```r
citation("edgeR")
```

```
See Section 1.2 in the User's Guide for more detail about how to cite
the different edgeR pipelines.

  Chen Y, Chen L, Lun ATL, Baldoni PL, Smyth GK (2024). edgeR 4.0:
  powerful differential analysis of sequencing data with expanded
  functionality and improved support for small counts and larger
  datasets. bioRxiv doi: 10.1101/2024.01.21.576131

  Chen Y, Lun ATL, Smyth GK (2016). From reads to genes to pathways:
  differential expression analysis of RNA-Seq experiments using
  Rsubread and the edgeR quasi-likelihood pipeline. F1000Research 5,
  1438

  McCarthy DJ, Chen Y and Smyth GK (2012). Differential expression
  analysis of multifactor RNA-Seq experiments with respect to
  biological variation. Nucleic Acids Research 40(10), 4288-4297

  Robinson MD, McCarthy DJ and Smyth GK (2010). edgeR: a Bioconductor
  package for differential expression analysis of digital gene
  expression data. Bioinformatics 26(1), 139-140

To see these entries in BibTeX format, use 'print(<citation>,
bibtex=TRUE)', 'toBibtex(.)', or set
'options(citation.bibtex.max=999)'.
```

```r
citation("apeglm")
```

```
To cite package 'apeglm' in publications use:

  Zhu, A., Ibrahim, J.G., Love, M.I. Heavy-tailed prior distributions
  for sequence count data: removing the noise and preserving large
  differences Bioinformatics (2018)

A BibTeX entry for LaTeX users is

  @Article{,
    title = {Heavy-tailed prior distributions for sequence count data: removing the noise and
    author = {Anqi Zhu and Joseph G. Ibrahim and Michael I. Love},
    year = {2018},
```

```
    journal = {Bioinformatics},
    doi = {10.1093/bioinformatics/bty895},
  }
```

```r
citation("sva")
```

```
To cite package 'sva' in publications use:

  Leek JT, Johnson WE, Parker HS, Fertig EJ, Jaffe AE, Zhang Y, Storey
  JD, Torres LC (2024). _sva: Surrogate Variable Analysis_.
  doi:10.18129/B9.bioc.sva <https://doi.org/10.18129/B9.bioc.sva>, R
  package version 3.52.0, <https://bioconductor.org/packages/sva>.

A BibTeX entry for LaTeX users is

  @Manual{,
    title = {sva: Surrogate Variable Analysis},
    author = {Jeffrey T. Leek and W. Evan Johnson and Hilary S. Parker and Elana J. Fertig a
    year = {2024},
    note = {R package version 3.52.0},
    url = {https://bioconductor.org/packages/sva},
    doi = {10.18129/B9.bioc.sva},
  }

ATTENTION: This citation information has been auto-generated from the
package DESCRIPTION file and may need manual editing, see
'help("citation")'.
```

```r
sessionInfo()
```

```
R version 4.4.2 (2024-10-31)
Platform: x86_64-pc-linux-gnu
Running under: Ubuntu 22.04.5 LTS

Matrix products: default
BLAS:   /usr/lib/x86_64-linux-gnu/blas/libblas.so.3.10.0
LAPACK: /usr/lib/x86_64-linux-gnu/lapack/liblapack.so.3.10.0

locale:
 [1] LC_CTYPE=en_AU.UTF-8       LC_NUMERIC=C
 [3] LC_TIME=en_AU.UTF-8        LC_COLLATE=en_AU.UTF-8
```

```
 [5] LC_MONETARY=en_AU.UTF-8    LC_MESSAGES=en_AU.UTF-8
 [7] LC_PAPER=en_AU.UTF-8       LC_NAME=C
 [9] LC_ADDRESS=C               LC_TELEPHONE=C
[11] LC_MEASUREMENT=en_AU.UTF-8 LC_IDENTIFICATION=C

time zone: Australia/Melbourne
tzcode source: system (glibc)

attached base packages:
[1] stats4    stats     graphics  grDevices utils     datasets  methods
[8] base

other attached packages:
 [1] ggrepel_0.9.6               sva_3.52.0
 [3] BiocParallel_1.38.0         genefilter_1.86.0
 [5] mgcv_1.9-1                  nlme_3.1-167
 [7] ggh4x_0.3.0                 ComplexUpset_1.3.3
 [9] UpSetR_1.4.0                ggpubr_0.6.0
[11] edgeR_4.2.2                 limma_3.60.6
[13] lubridate_1.9.4             forcats_1.0.0
[15] stringr_1.5.1               dplyr_1.1.4
[17] purrr_1.0.2                 readr_2.1.5
[19] tidyr_1.3.1                 tibble_3.2.1
[21] ggplot2_3.5.1               tidyverse_2.0.0
[23] DESeq2_1.44.0               SummarizedExperiment_1.34.0
[25] Biobase_2.64.0              MatrixGenerics_1.16.0
[27] matrixStats_1.5.0           GenomicRanges_1.56.2
[29] GenomeInfoDb_1.40.1         IRanges_2.38.1
[31] S4Vectors_0.42.1            BiocGenerics_0.50.0
[33] mirrorCheck_0.0.1.0

loaded via a namespace (and not attached):
 [1] DBI_1.2.3               formatR_1.14            gridExtra_2.3
 [4] rlang_1.1.5             magrittr_2.0.3          compiler_4.4.2
 [7] RSQLite_2.3.9           png_0.1-8               vctrs_0.6.5
[10] pkgconfig_2.0.3         crayon_1.5.3            fastmap_1.2.0
[13] backports_1.5.0         XVector_0.44.0          labeling_0.4.3
[16] utf8_1.2.4              rmarkdown_2.29          tzdb_0.4.0
[19] UCSC.utils_1.0.0        bit_4.5.0.1             xfun_0.50
[22] cachem_1.1.0            zlibbioc_1.50.0         jsonlite_1.8.9
[25] blob_1.2.4              DelayedArray_0.30.1     broom_1.0.7
[28] parallel_4.4.2          R6_2.6.1                RColorBrewer_1.1-3
[31] stringi_1.8.4           car_3.1-3               numDeriv_2016.8-1.1
```

```
[34] Rcpp_1.0.14          knitr_1.49             VennDiagram_1.7.3
[37] Matrix_1.7-2         splines_4.4.2          timechange_0.3.0
[40] tidyselect_1.2.1     rstudioapi_0.17.1      abind_1.4-8
[43] yaml_2.3.10          codetools_0.2-19       lattice_0.22-5
[46] plyr_1.8.9           KEGGREST_1.44.1        withr_3.0.2
[49] coda_0.19-4.1        evaluate_1.0.3         lambda.r_1.2.4
[52] survival_3.8-3       futile.logger_1.4.3    Biostrings_2.72.1
[55] pillar_1.10.1        carData_3.0-5          generics_0.1.3
[58] emdbook_1.3.13       hms_1.1.3              munsell_0.5.1
[61] scales_1.3.0         xtable_1.8-4           glue_1.8.0
[64] pheatmap_1.0.12      apeglm_1.26.1          tools_4.4.2
[67] annotate_1.82.0      locfit_1.5-9.11        ggsignif_0.6.4
[70] mvtnorm_1.3-3        XML_3.99-0.18          grid_4.4.2
[73] bbmle_1.0.25.1       bdsmatrix_1.3-7        AnnotationDbi_1.66.0
[76] colorspace_2.1-1     GenomeInfoDbData_1.2.12 patchwork_1.3.0
[79] Formula_1.2-5        cli_3.6.4              futile.options_1.0.1
[82] S4Arrays_1.4.1       gtable_0.3.6           rstatix_0.7.2
[85] digest_0.6.37        SparseArray_1.4.8      farver_2.1.2
[88] memoise_2.0.1        htmltools_0.5.8.1      lifecycle_1.0.4
[91] httr_1.4.7           statmod_1.5.0          MASS_7.3-64
[94] bit64_4.6.0-1
```
