# Supplementary material for: mirrorCheck: an R package facilitating informed use of DESeq2’s lfcShrink() function for differential gene expression analysis of clinical samples
Source: Bioinform Adv. 2025 Apr 2;5(1):vbaf070. doi: 10.1093/bioadv/vbaf070 (PMC12089695; doi:10.1093/bioadv/vbaf070)
Supplement: vbaf070_Supplementary_Data [file vbaf070_supplementary_data.zip › final supps/S4_Supplementary_TCGABRCA_report.pdf]

TCGA BRCA

Kate Scull

TCGA Example Data

This analysis uses data from the Genomic Data Commons (GDC) database. It downloads data from over 1000 samples using the TCGAbiolinks R package. Although we have tried to minimise the size of R objects stored in memory, you'll need a computer with at least 32G RAM to render this Quarto. You will also need to ensure to following R libraries are installed, including mirrorCheck from the github repository kescull/mirrorCheck: Facilitator functions for getting and assessing DESeq2 lfcShrink results (github.com)

MirrorCheck

Setup and download:

```
library(mirrorCheck)
library(TCGAbiolinks)
library(DESeq2)
library(tidyverse)
library(ggh4x)
library(edgeR)
library(UpSetR)
library(ComplexUpset)
library(ggpubr)
library(ggh4x)
library(sva)

BRCA.query <- GDCquery(project = "TCGA-BRCA",
                       data.category = "Transcriptome Profiling",
                       data.type = "Gene Expression Quantification",
                       workflow.type = "STAR - Counts",
                       access = "open",
                       data.format = "TSV",
```

```r
                            experimental.strategy = "RNA-Seq")

GDCdownload(BRCA.query, directory = "BRCA",files.per.chunk = 10)
se <- GDCprepare(BRCA.query,directory = "BRCA")
```

Prepare dds, keeping raw counts data:

```r
dds <- DESeqDataSet(se,design = ~1)
```

```
renaming the first element in assays to 'counts'
```

```r
dds
```

```
class: DESeqDataSet
dim: 60660 1231
metadata(2): data_release version
assays(6): counts stranded_first ... fpkm_unstrand fpkm_uq_unstrand
rownames(60660): ENSG00000000003.15 ENSG00000000005.6 ...
  ENSG00000288674.1 ENSG00000288675.1
rowData names(10): source type ... hgnc_id havana_gene
colnames(1231): TCGA-A2-A25D-01A-12R-A16F-07
  TCGA-BH-A201-01A-11R-A14M-07 ... TCGA-A2-A04W-01A-31R-A115-07
  TCGA-AR-A240-01A-11R-A169-07
colData names(97): barcode patient ... paper_PARADIGM Clusters
  paper_Pan-Gyn Clusters
```

```r
assayNames(dds)
```

```
[1] "counts"           "stranded_first"   "stranded_second"  "tpm_unstrand"
[5] "fpkm_unstrand"    "fpkm_uq_unstrand"
```

```r
assays(dds)[2:6] <- NULL
dds
```

```
class: DESeqDataSet
dim: 60660 1231
metadata(2): data_release version
assays(1): counts
rownames(60660): ENSG00000000003.15 ENSG00000000005.6 ...
```

```
  ENSG00000288674.1 ENSG00000288675.1
rowData names(10): source type ... hgnc_id havana_gene
colnames(1231): TCGA-A2-A25D-01A-12R-A16F-07
  TCGA-BH-A201-01A-11R-A14M-07 ... TCGA-A2-A04W-01A-31R-A115-07
  TCGA-AR-A240-01A-11R-A169-07
colData names(97): barcode patient ... paper_PARADIGM Clusters
  paper_Pan-Gyn Clusters
```

```r
gc()
```

```
            used   (Mb) gc trigger    (Mb)   max used    (Mb)
Ncells   8464689  452.1   15175877   810.5   12072821   644.8
Vcells 355233976 2710.3 1800964670 13740.3 2251205837 17175.4
```

```r
metadata_check <- colData(dds)[,c("barcode",
                                  "paper_BRCA_Subtype_PAM50","sample_type")]
metadata_check <- as.data.frame(metadata_check)
any(is.na(metadata_check$barcode))
```

```
[1] FALSE
```

```r
any(is.na(metadata_check$sample_type))
```

```
[1] FALSE
```

```r
sum(is.na(metadata_check$paper_BRCA_Subtype_PAM50))
```

```
[1] 132
```

```r
table(metadata_check$sample_type,metadata_check$paper_BRCA_Subtype_PAM50)
```

```
                      Basal Her2 LumA LumB Normal
  Metastatic              0    0    0    0      0
  Primary Tumor         197   82  571  209     40
  Solid Tissue Normal     0    0    0    0      0
```

```r
sum(metadata_check$sample_type == "Metastatic")
```

```
[1] 7
```

```r
# discard all samples with NA for paper_BRCA_Subtype_PAM50, except for samples
# with sample_type "Solid Tissue Normal" - keep these as control
discards <- metadata_check %>%
  filter(is.na(paper_BRCA_Subtype_PAM50) &
           sample_type != "Solid Tissue Normal") %>%
  pull(barcode)
str(discards)
```

```
 chr [1:19] "TCGA-EW-A1PD-01A-11R-A144-07" "TCGA-EW-A6SA-01A-21R-A32P-07" ...
```

```r
dds
```

```
class: DESeqDataSet
dim: 60660 1231
metadata(2): data_release version
assays(1): counts
rownames(60660): ENSG00000000003.15 ENSG00000000005.6 ...
  ENSG00000288674.1 ENSG00000288675.1
rowData names(10): source type ... hgnc_id havana_gene
colnames(1231): TCGA-A2-A25D-01A-12R-A16F-07
  TCGA-BH-A201-01A-11R-A14M-07 ... TCGA-A2-A04W-01A-31R-A115-07
  TCGA-AR-A24O-01A-11R-A169-07
colData names(97): barcode patient ... paper_PARADIGM Clusters
  paper_Pan-Gyn Clusters
```

```r
dds <- dds[,!(dds$barcode %in% discards)]
dds
```

```
class: DESeqDataSet
dim: 60660 1212
metadata(2): data_release version
assays(1): counts
rownames(60660): ENSG00000000003.15 ENSG00000000005.6 ...
  ENSG00000288674.1 ENSG00000288675.1
rowData names(10): source type ... hgnc_id havana_gene
```

```
colnames(1212): TCGA-A2-A25D-01A-12R-A16F-07
  TCGA-BH-A201-01A-11R-A14M-07 ... TCGA-A2-A04W-01A-31R-A115-07
  TCGA-AR-A240-01A-11R-A169-07
colData names(97): barcode patient ... paper_PARADIGM Clusters
  paper_Pan-Gyn Clusters
```

```
making_group <- colData(dds)[,c("barcode",
                                "paper_BRCA_Subtype_PAM50","sample_type")]
making_group <- as.data.frame(making_group)
table(making_group$sample_type)
```

```

      Primary Tumor Solid Tissue Normal 
               1099                 113 
```

```
making_group <- making_group %>%
  mutate(group = case_when(sample_type == "Solid Tissue Normal" ~ "Control",
                           .default = paper_BRCA_Subtype_PAM50),
         across(group,as.factor))
table(making_group$group)
```

```

  Basal Control    Her2    LumA    LumB  Normal 
    197     113      82     571     209      40 
```

```
test_match_order <- function(x,y) {
  if (isTRUE(all.equal(x,y))) print('Perfect match in same order')
  if (!isTRUE(all.equal(x,y)) && isTRUE(all.equal(sort(x),sort(y))))
    print('Perfect match in wrong order')
  if (!isTRUE(all.equal(x,y)) && !isTRUE(all.equal(sort(x),sort(y))))
    print('No match')
}

test_match_order(making_group$barcode,dds$barcode)
```

```
[1] "Perfect match in same order"
```

```
dds$group <- making_group$group
dds$group <- relevel(dds$group, ref = "Control")
design(dds) <- ~group
saveRDS(dds, file = "quartodds.rds")
rm(list = ls())
gc()
```

```
           used  (Mb) gc trigger    (Mb)  max used    (Mb)
Ncells  8226930 439.4   15175877   810.5  12072821   644.8
Vcells 14808205 113.0 1440771736 10992.3 2251205837 17175.4
```

Principal component analysis:

```
dds <- readRDS("quartodds.rds")
vsd <- vst(dds)
pdata <- DESeq2::plotPCA(vsd, ntop = length(vsd),
                         intgroup= "group",
                         pcsToUse = 1:2, returnData=T)
```

```
using ntop=60660 top features by variance
```

```
percentVar <- attr(pdata,"percentVar")
colour_blind_friendly <- c('#EE7733', '#0077BB','#BBBBBB','#EE3377',
                           '#33BBEE', '#CC3311', '#009988' )
p <- ggplot(pdata, aes(x=PC1,y=PC2,color=group)) +
  geom_point(size = 2, alpha = 0.5) +
  ggtitle("TCGA BRCA") +
  labs(x = paste0("PC1: ",round(percentVar[1]*100),"% variance"),
       y = paste0("PC2: ",round(percentVar[2]*100),"% variance"),
       color = "PAM50 subtype") +
  theme_classic(base_size = 16) +
  theme(plot.title = element_text(face = "bold")) +
  force_panelsizes(rows = unit(2.5,"in"),
                   cols = unit(2.5,"in")) +
  scale_colour_manual(values = colour_blind_friendly)
p
```

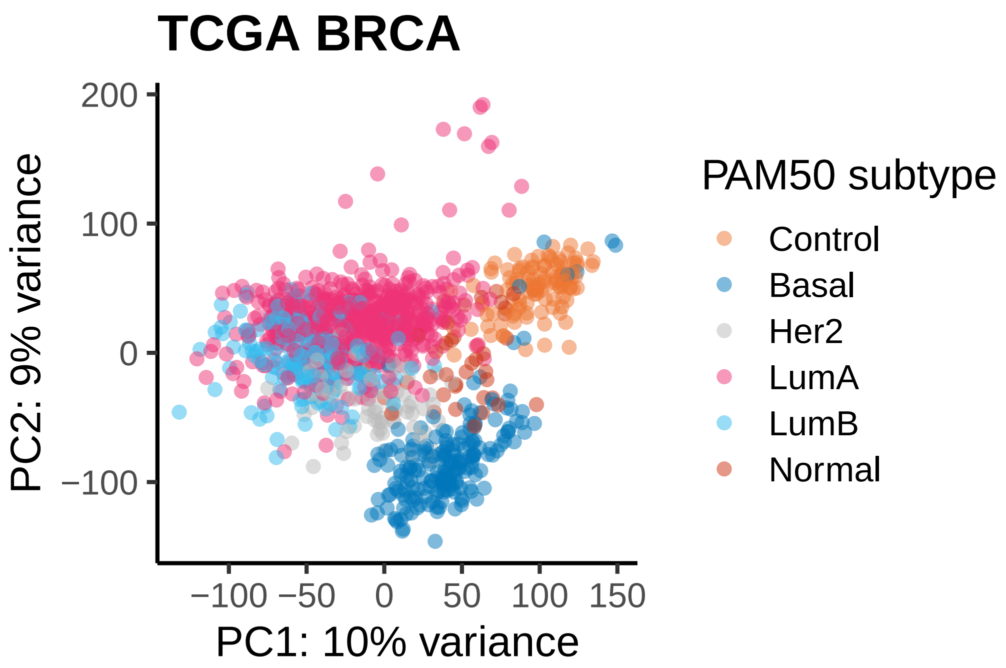


```r
rm(vsd)
rm(pdata)
rm(p)
gc()
```

```
           used  (Mb) gc trigger   (Mb)   max used    (Mb)
Ncells  8656938 462.4   15175877  810.5   15175877   810.5
Vcells 53704115 409.8 1152617389 8793.8 2251205837 17175.4
```

Run DESeq2

Run DESeq2 using lfcShrink, facilitated by mirrorCheck, *without any further clean-up steps* This creates output csv tables and pdf reports in a folder alongside this qmd file.

```r
folder <- "DESeq_noclean"
dir.create(folder)
```

```
Warning in dir.create(folder): 'DESeq_noclean' already exists
```

```r
rownam2symbol = as.data.frame(rowData(dds)[, colnames(rowData(dds)) %in%
                                            c("gene_id","gene_name")]) %>%
  rename(rowname = gene_id,
         g_symbol = gene_name)
head(rowname2symbol)
```

```
                              rowname g_symbol
ENSG00000000003.15 ENSG00000000003.15   TSPAN6
ENSG00000000005.6   ENSG00000000005.6     TNMD
ENSG00000000419.13 ENSG00000000419.13     DPM1
ENSG00000000457.14 ENSG00000000457.14    SCYL3
ENSG00000000460.17 ENSG00000000460.17 C1orf112
ENSG00000000938.13 ENSG00000000938.13      FGR
```

```r
run_DESeq_all_contrasts(dds, folder, condition = "group",
                        rowname2symbol = rowname2symbol,
                        p.cutoff = 0.01,
                        top.n = 0,
                        useDingbats = T)
```

```
estimating size factors
```

```
estimating dispersions
```

```
gene-wise dispersion estimates
```

```
mean-dispersion relationship
```

```
final dispersion estimates
```

```
fitting model and testing
```

```
-- replacing outliers and refitting for 9848 genes
-- DESeq argument 'minReplicatesForReplace' = 7
-- original counts are preserved in counts(dds)
```

```
estimating dispersions
```

```
fitting model and testing

using 'apeglm' for LFC shrinkage. If used in published research, please cite:
    Zhu, A., Ibrahim, J.G., Love, M.I. (2018) Heavy-tailed prior distributions for
    sequence count data: removing the noise and preserving large differences.
    Bioinformatics. https://doi.org/10.1093/bioinformatics/bty895
using 'apeglm' for LFC shrinkage. If used in published research, please cite:
    Zhu, A., Ibrahim, J.G., Love, M.I. (2018) Heavy-tailed prior distributions for
    sequence count data: removing the noise and preserving large differences.
    Bioinformatics. https://doi.org/10.1093/bioinformatics/bty895
using 'apeglm' for LFC shrinkage. If used in published research, please cite:
    Zhu, A., Ibrahim, J.G., Love, M.I. (2018) Heavy-tailed prior distributions for
    sequence count data: removing the noise and preserving large differences.
    Bioinformatics. https://doi.org/10.1093/bioinformatics/bty895
using 'apeglm' for LFC shrinkage. If used in published research, please cite:
    Zhu, A., Ibrahim, J.G., Love, M.I. (2018) Heavy-tailed prior distributions for
    sequence count data: removing the noise and preserving large differences.
    Bioinformatics. https://doi.org/10.1093/bioinformatics/bty895
using 'apeglm' for LFC shrinkage. If used in published research, please cite:
    Zhu, A., Ibrahim, J.G., Love, M.I. (2018) Heavy-tailed prior distributions for
    sequence count data: removing the noise and preserving large differences.
    Bioinformatics. https://doi.org/10.1093/bioinformatics/bty895

Joining with `by = join_by(rowname)`
Joining with `by = join_by(rowname)`
Joining with `by = join_by(rowname)`
Joining with `by = join_by(rowname)`
Joining with `by = join_by(rowname)`
Joining with `by = join_by(name)`
Joining with `by = join_by(name)`
Joining with `by = join_by(name)`
Joining with `by = join_by(name)`
Joining with `by = join_by(name)`
found results columns, replacing these
using 'apeglm' for LFC shrinkage. If used in published research, please cite:
Zhu, A., Ibrahim, J.G., Love, M.I. (2018) Heavy-tailed prior distributions for
sequence count data: removing the noise and preserving large differences.
Bioinformatics. https://doi.org/10.1093/bioinformatics/bty895
using 'apeglm' for LFC shrinkage. If used in published research, please cite:
Zhu, A., Ibrahim, J.G., Love, M.I. (2018) Heavy-tailed prior distributions for
sequence count data: removing the noise and preserving large differences.
Bioinformatics. https://doi.org/10.1093/bioinformatics/bty895
```

```
using 'apeglm' for LFC shrinkage. If used in published research, please cite:
Zhu, A., Ibrahim, J.G., Love, M.I. (2018) Heavy-tailed prior distributions for
sequence count data: removing the noise and preserving large differences.
Bioinformatics. https://doi.org/10.1093/bioinformatics/bty895
using 'apeglm' for LFC shrinkage. If used in published research, please cite:
Zhu, A., Ibrahim, J.G., Love, M.I. (2018) Heavy-tailed prior distributions for
sequence count data: removing the noise and preserving large differences.
Bioinformatics. https://doi.org/10.1093/bioinformatics/bty895
using 'apeglm' for LFC shrinkage. If used in published research, please cite:
Zhu, A., Ibrahim, J.G., Love, M.I. (2018) Heavy-tailed prior distributions for
sequence count data: removing the noise and preserving large differences.
Bioinformatics. https://doi.org/10.1093/bioinformatics/bty895
Joining with `by = join_by(rowname)`
Joining with `by = join_by(rowname)`
Joining with `by = join_by(rowname)`
Joining with `by = join_by(rowname)`
Joining with `by = join_by(rowname)`
Joining with `by = join_by(name)`
Joining with `by = join_by(name)`
Joining with `by = join_by(name)`
Joining with `by = join_by(name)`
Joining with `by = join_by(name)`
found results columns, replacing these
using 'apeglm' for LFC shrinkage. If used in published research, please cite:
Zhu, A., Ibrahim, J.G., Love, M.I. (2018) Heavy-tailed prior distributions for
sequence count data: removing the noise and preserving large differences.
Bioinformatics. https://doi.org/10.1093/bioinformatics/bty895
using 'apeglm' for LFC shrinkage. If used in published research, please cite:
Zhu, A., Ibrahim, J.G., Love, M.I. (2018) Heavy-tailed prior distributions for
sequence count data: removing the noise and preserving large differences.
Bioinformatics. https://doi.org/10.1093/bioinformatics/bty895
using 'apeglm' for LFC shrinkage. If used in published research, please cite:
Zhu, A., Ibrahim, J.G., Love, M.I. (2018) Heavy-tailed prior distributions for
sequence count data: removing the noise and preserving large differences.
Bioinformatics. https://doi.org/10.1093/bioinformatics/bty895
using 'apeglm' for LFC shrinkage. If used in published research, please cite:
Zhu, A., Ibrahim, J.G., Love, M.I. (2018) Heavy-tailed prior distributions for
sequence count data: removing the noise and preserving large differences.
Bioinformatics. https://doi.org/10.1093/bioinformatics/bty895
using 'apeglm' for LFC shrinkage. If used in published research, please cite:
Zhu, A., Ibrahim, J.G., Love, M.I. (2018) Heavy-tailed prior distributions for
sequence count data: removing the noise and preserving large differences.
Bioinformatics. https://doi.org/10.1093/bioinformatics/bty895
```

```
Joining with `by = join_by(rowname)`
Joining with `by = join_by(rowname)`
Joining with `by = join_by(rowname)`
Joining with `by = join_by(rowname)`
Joining with `by = join_by(rowname)`
Joining with `by = join_by(name)`
Joining with `by = join_by(name)`
Joining with `by = join_by(name)`
Joining with `by = join_by(name)`
Joining with `by = join_by(name)`
found results columns, replacing these
using 'apeglm' for LFC shrinkage. If used in published research, please cite:
Zhu, A., Ibrahim, J.G., Love, M.I. (2018) Heavy-tailed prior distributions for
sequence count data: removing the noise and preserving large differences.
Bioinformatics. https://doi.org/10.1093/bioinformatics/bty895
using 'apeglm' for LFC shrinkage. If used in published research, please cite:
Zhu, A., Ibrahim, J.G., Love, M.I. (2018) Heavy-tailed prior distributions for
sequence count data: removing the noise and preserving large differences.
Bioinformatics. https://doi.org/10.1093/bioinformatics/bty895
using 'apeglm' for LFC shrinkage. If used in published research, please cite:
Zhu, A., Ibrahim, J.G., Love, M.I. (2018) Heavy-tailed prior distributions for
sequence count data: removing the noise and preserving large differences.
Bioinformatics. https://doi.org/10.1093/bioinformatics/bty895
using 'apeglm' for LFC shrinkage. If used in published research, please cite:
Zhu, A., Ibrahim, J.G., Love, M.I. (2018) Heavy-tailed prior distributions for
sequence count data: removing the noise and preserving large differences.
Bioinformatics. https://doi.org/10.1093/bioinformatics/bty895
using 'apeglm' for LFC shrinkage. If used in published research, please cite:
Zhu, A., Ibrahim, J.G., Love, M.I. (2018) Heavy-tailed prior distributions for
sequence count data: removing the noise and preserving large differences.
Bioinformatics. https://doi.org/10.1093/bioinformatics/bty895
Joining with `by = join_by(rowname)`
Joining with `by = join_by(rowname)`
Joining with `by = join_by(rowname)`
Joining with `by = join_by(rowname)`
Joining with `by = join_by(rowname)`
Joining with `by = join_by(name)`
Joining with `by = join_by(name)`
Joining with `by = join_by(name)`
Joining with `by = join_by(name)`
Joining with `by = join_by(name)`
found results columns, replacing these
using 'apeglm' for LFC shrinkage. If used in published research, please cite:
```

```
Zhu, A., Ibrahim, J.G., Love, M.I. (2018) Heavy-tailed prior distributions for
sequence count data: removing the noise and preserving large differences.
Bioinformatics. https://doi.org/10.1093/bioinformatics/bty895
using 'apeglm' for LFC shrinkage. If used in published research, please cite:
Zhu, A., Ibrahim, J.G., Love, M.I. (2018) Heavy-tailed prior distributions for
sequence count data: removing the noise and preserving large differences.
Bioinformatics. https://doi.org/10.1093/bioinformatics/bty895
using 'apeglm' for LFC shrinkage. If used in published research, please cite:
Zhu, A., Ibrahim, J.G., Love, M.I. (2018) Heavy-tailed prior distributions for
sequence count data: removing the noise and preserving large differences.
Bioinformatics. https://doi.org/10.1093/bioinformatics/bty895
using 'apeglm' for LFC shrinkage. If used in published research, please cite:
Zhu, A., Ibrahim, J.G., Love, M.I. (2018) Heavy-tailed prior distributions for
sequence count data: removing the noise and preserving large differences.
Bioinformatics. https://doi.org/10.1093/bioinformatics/bty895
using 'apeglm' for LFC shrinkage. If used in published research, please cite:
Zhu, A., Ibrahim, J.G., Love, M.I. (2018) Heavy-tailed prior distributions for
sequence count data: removing the noise and preserving large differences.
Bioinformatics. https://doi.org/10.1093/bioinformatics/bty895
Joining with `by = join_by(rowname)`
Joining with `by = join_by(rowname)`
Joining with `by = join_by(rowname)`
Joining with `by = join_by(rowname)`
Joining with `by = join_by(rowname)`
Joining with `by = join_by(name)`
Joining with `by = join_by(name)`
Joining with `by = join_by(name)`
Joining with `by = join_by(name)`
Joining with `by = join_by(name)`
found results columns, replacing these
using 'apeglm' for LFC shrinkage. If used in published research, please cite:
Zhu, A., Ibrahim, J.G., Love, M.I. (2018) Heavy-tailed prior distributions for
sequence count data: removing the noise and preserving large differences.
Bioinformatics. https://doi.org/10.1093/bioinformatics/bty895
using 'apeglm' for LFC shrinkage. If used in published research, please cite:
Zhu, A., Ibrahim, J.G., Love, M.I. (2018) Heavy-tailed prior distributions for
sequence count data: removing the noise and preserving large differences.
Bioinformatics. https://doi.org/10.1093/bioinformatics/bty895
using 'apeglm' for LFC shrinkage. If used in published research, please cite:
Zhu, A., Ibrahim, J.G., Love, M.I. (2018) Heavy-tailed prior distributions for
sequence count data: removing the noise and preserving large differences.
Bioinformatics. https://doi.org/10.1093/bioinformatics/bty895
using 'apeglm' for LFC shrinkage. If used in published research, please cite:
```

```
Zhu, A., Ibrahim, J.G., Love, M.I. (2018) Heavy-tailed prior distributions for
sequence count data: removing the noise and preserving large differences.
Bioinformatics. https://doi.org/10.1093/bioinformatics/bty895
using 'apeglm' for LFC shrinkage. If used in published research, please cite:
Zhu, A., Ibrahim, J.G., Love, M.I. (2018) Heavy-tailed prior distributions for
sequence count data: removing the noise and preserving large differences.
Bioinformatics. https://doi.org/10.1093/bioinformatics/bty895
Joining with `by = join_by(rowname)`
Joining with `by = join_by(rowname)`
Joining with `by = join_by(rowname)`
Joining with `by = join_by(rowname)`
Joining with `by = join_by(rowname)`
Joining with `by = join_by(name)`
Joining with `by = join_by(name)`
Joining with `by = join_by(name)`
Joining with `by = join_by(name)`
Joining with `by = join_by(name)`
```

```
group <- levels(dds$group)
rm(dds)
set <- compare_reciprocal_contrasts(group,folder)
```

```
Warning: Removed 1 row containing non-finite outside the scale range
(`stat_density()`).
```

```
saveRDS(set,"BRCA_noclean.rds")
rm(list = ls())
gc()
```

```
           used  (Mb) gc trigger   (Mb)   max used    (Mb)
Ncells  8906687 475.7   15175883  810.5   15175883   810.5
Vcells 17539609 133.9 1261124121 9621.7 2251205837 17175.4
```

Print resized diagnostic stacked bar chart for publication:

```
make_diagnostic <- function(this_set) {
  ready.for.overlap.check <- lapply(this_set,function(x) x %>%
                                      select(partition) %>%
                                      mutate(total = n()) %>%
                                      group_by(partition) %>%
```

```
                                    summarise(n = n(),
                                              total = sum(total)/n,
                                              pc = n/total *100))
  all.overlap <- bind_rows(ready.for.overlap.check, .id = "group")

  group.labels <- gsub("\\.","\n", all.overlap$group)
  names(group.labels) <- all.overlap$group
  legend.labels <- c("Concordant",
                     "Only with group1 as ref",
                     "Only with group2 as ref")

  barPlot <- ggplot(all.overlap, aes(x = group,
                                     y = pc,
                                     fill = partition,
                                     label = n)) +
    geom_bar(stat = "identity") +
    xlab(element_blank()) +
    ylab("Percentage of differentially expressed genes") +
    scale_x_discrete(labels=group.labels) +
    theme_classic(base_size = 13) +
    theme(legend.position = "right",
        legend.box.spacing = unit(0,"cm"),
        axis.text = element_text(face="bold",lineheight = 0.6)) +
    guides(fill = guide_legend(title = element_blank(),reverse=T)) +
    scale_fill_manual(labels = legend.labels,
                      values = c('#009988', '#EE7733','#CC3311')) +
    geom_text(position = position_stack(vjust = 0.5),size = 4) +
    coord_flip()
  barPlot
}
set <- readRDS("BRCA_noclean.rds")
make_diagnostic(set)
```

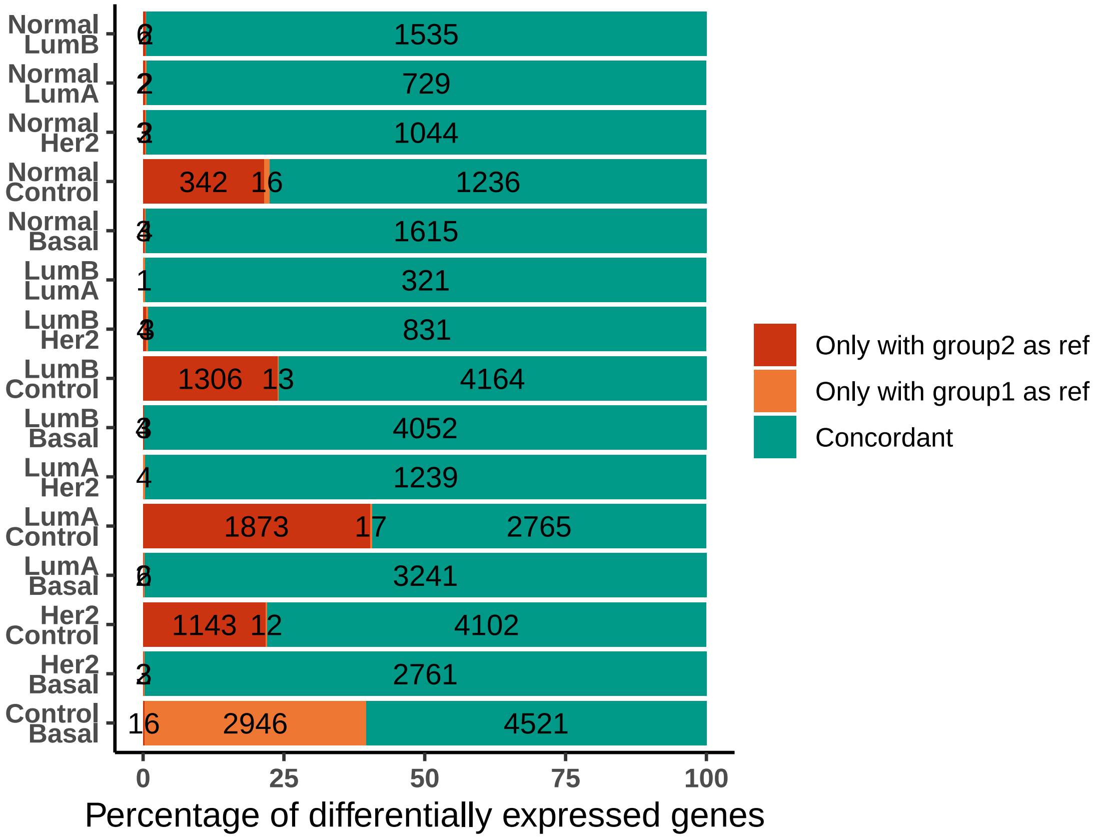


```
rm(set)
```

Run DESeq2 using lfcShrink, facilitated by mirrorCheck, *after prefiltering.* This creates output csv tables and pdf reports in a folder alongside this qmd file.

```
folder <- "DESeq_prefilt"
dir.create(folder)
```

```
Warning in dir.create(folder): 'DESeq_prefilt' already exists
```

```
dds <- readRDS("quartodds.rds")
rowname2symbol = as.data.frame(rowData(dds)[, colnames(rowData(dds)) %in%
                                              c("gene_id","gene_name")]) %>%
  rename(rowname = gene_id,
         g_symbol = gene_name)
head(rowname2symbol)
```

```
                              rowname g_symbol
ENSG00000000003.15 ENSG00000000003.15   TSPAN6
ENSG00000000005.6   ENSG00000000005.6     TNMD
ENSG00000000419.13 ENSG00000000419.13     DPM1
ENSG00000000457.14 ENSG00000000457.14    SCYL3
ENSG00000000460.17 ENSG00000000460.17 C1orf112
ENSG00000000938.13 ENSG00000000938.13      FGR
```

```r
expr.filter <- edgeR::filterByExpr(dds, group = dds$group)
dds.filt <- dds[expr.filter, ]
dds.filt
```

```
class: DESeqDataSet
dim: 30954 1212
metadata(2): data_release version
assays(1): counts
rownames(30954): ENSG00000000003.15 ENSG00000000005.6 ...
  ENSG00000288674.1 ENSG00000288675.1
rowData names(10): source type ... hgnc_id havana_gene
colnames(1212): TCGA-A2-A25D-01A-12R-A16F-07
  TCGA-BH-A201-01A-11R-A14M-07 ... TCGA-A2-A04W-01A-31R-A115-07
  TCGA-AR-A240-01A-11R-A169-07
colData names(98): barcode patient ... paper_Pan-Gyn Clusters group
```

```r
rm(dds)
gc()
```

```
           used  (Mb) gc trigger   (Mb)   max used    (Mb)
Ncells  9105187 486.3   15175883  810.5   15175883   810.5
Vcells 37475371 286.0 1008899297 7697.3 2251205837 17175.4
```

```r
run_DESeq_all_contrasts(dds.filt, folder, condition = "group",
                        rowname2symbol = rowname2symbol,
                        p.cutoff = 0.01,
                        top.n = 0,
                        useDingbats = T)
```

```
estimating size factors
```

```
estimating dispersions
```

```
gene-wise dispersion estimates

mean-dispersion relationship

final dispersion estimates

fitting model and testing

-- replacing outliers and refitting for 3788 genes
-- DESeq argument 'minReplicatesForReplace' = 7
-- original counts are preserved in counts(dds)

estimating dispersions

fitting model and testing

using 'apeglm' for LFC shrinkage. If used in published research, please cite:
    Zhu, A., Ibrahim, J.G., Love, M.I. (2018) Heavy-tailed prior distributions for
    sequence count data: removing the noise and preserving large differences.
    Bioinformatics. https://doi.org/10.1093/bioinformatics/bty895
using 'apeglm' for LFC shrinkage. If used in published research, please cite:
    Zhu, A., Ibrahim, J.G., Love, M.I. (2018) Heavy-tailed prior distributions for
    sequence count data: removing the noise and preserving large differences.
    Bioinformatics. https://doi.org/10.1093/bioinformatics/bty895
using 'apeglm' for LFC shrinkage. If used in published research, please cite:
    Zhu, A., Ibrahim, J.G., Love, M.I. (2018) Heavy-tailed prior distributions for
    sequence count data: removing the noise and preserving large differences.
    Bioinformatics. https://doi.org/10.1093/bioinformatics/bty895
using 'apeglm' for LFC shrinkage. If used in published research, please cite:
    Zhu, A., Ibrahim, J.G., Love, M.I. (2018) Heavy-tailed prior distributions for
    sequence count data: removing the noise and preserving large differences.
    Bioinformatics. https://doi.org/10.1093/bioinformatics/bty895
using 'apeglm' for LFC shrinkage. If used in published research, please cite:
    Zhu, A., Ibrahim, J.G., Love, M.I. (2018) Heavy-tailed prior distributions for
    sequence count data: removing the noise and preserving large differences.
    Bioinformatics. https://doi.org/10.1093/bioinformatics/bty895
```

```
Joining with `by = join_by(rowname)`
Joining with `by = join_by(rowname)`
Joining with `by = join_by(rowname)`
Joining with `by = join_by(rowname)`
Joining with `by = join_by(rowname)`
Joining with `by = join_by(name)`
Joining with `by = join_by(name)`
Joining with `by = join_by(name)`
Joining with `by = join_by(name)`
Joining with `by = join_by(name)`
found results columns, replacing these
using 'apeglm' for LFC shrinkage. If used in published research, please cite:
Zhu, A., Ibrahim, J.G., Love, M.I. (2018) Heavy-tailed prior distributions for
sequence count data: removing the noise and preserving large differences.
Bioinformatics. https://doi.org/10.1093/bioinformatics/bty895
using 'apeglm' for LFC shrinkage. If used in published research, please cite:
Zhu, A., Ibrahim, J.G., Love, M.I. (2018) Heavy-tailed prior distributions for
sequence count data: removing the noise and preserving large differences.
Bioinformatics. https://doi.org/10.1093/bioinformatics/bty895
using 'apeglm' for LFC shrinkage. If used in published research, please cite:
Zhu, A., Ibrahim, J.G., Love, M.I. (2018) Heavy-tailed prior distributions for
sequence count data: removing the noise and preserving large differences.
Bioinformatics. https://doi.org/10.1093/bioinformatics/bty895
using 'apeglm' for LFC shrinkage. If used in published research, please cite:
Zhu, A., Ibrahim, J.G., Love, M.I. (2018) Heavy-tailed prior distributions for
sequence count data: removing the noise and preserving large differences.
Bioinformatics. https://doi.org/10.1093/bioinformatics/bty895
using 'apeglm' for LFC shrinkage. If used in published research, please cite:
Zhu, A., Ibrahim, J.G., Love, M.I. (2018) Heavy-tailed prior distributions for
sequence count data: removing the noise and preserving large differences.
Bioinformatics. https://doi.org/10.1093/bioinformatics/bty895
Joining with `by = join_by(rowname)`
Joining with `by = join_by(rowname)`
Joining with `by = join_by(rowname)`
Joining with `by = join_by(rowname)`
Joining with `by = join_by(rowname)`
Joining with `by = join_by(name)`
Joining with `by = join_by(name)`
Joining with `by = join_by(name)`
Joining with `by = join_by(name)`
Joining with `by = join_by(name)`
found results columns, replacing these
using 'apeglm' for LFC shrinkage. If used in published research, please cite:
```

```
Zhu, A., Ibrahim, J.G., Love, M.I. (2018) Heavy-tailed prior distributions for
sequence count data: removing the noise and preserving large differences.
Bioinformatics. https://doi.org/10.1093/bioinformatics/bty895
using 'apeglm' for LFC shrinkage. If used in published research, please cite:
Zhu, A., Ibrahim, J.G., Love, M.I. (2018) Heavy-tailed prior distributions for
sequence count data: removing the noise and preserving large differences.
Bioinformatics. https://doi.org/10.1093/bioinformatics/bty895
using 'apeglm' for LFC shrinkage. If used in published research, please cite:
Zhu, A., Ibrahim, J.G., Love, M.I. (2018) Heavy-tailed prior distributions for
sequence count data: removing the noise and preserving large differences.
Bioinformatics. https://doi.org/10.1093/bioinformatics/bty895
using 'apeglm' for LFC shrinkage. If used in published research, please cite:
Zhu, A., Ibrahim, J.G., Love, M.I. (2018) Heavy-tailed prior distributions for
sequence count data: removing the noise and preserving large differences.
Bioinformatics. https://doi.org/10.1093/bioinformatics/bty895
using 'apeglm' for LFC shrinkage. If used in published research, please cite:
Zhu, A., Ibrahim, J.G., Love, M.I. (2018) Heavy-tailed prior distributions for
sequence count data: removing the noise and preserving large differences.
Bioinformatics. https://doi.org/10.1093/bioinformatics/bty895
Joining with `by = join_by(rowname)`
Joining with `by = join_by(rowname)`
Joining with `by = join_by(rowname)`
Joining with `by = join_by(rowname)`
Joining with `by = join_by(rowname)`
Joining with `by = join_by(name)`
Joining with `by = join_by(name)`
Joining with `by = join_by(name)`
Joining with `by = join_by(name)`
Joining with `by = join_by(name)`
found results columns, replacing these
using 'apeglm' for LFC shrinkage. If used in published research, please cite:
Zhu, A., Ibrahim, J.G., Love, M.I. (2018) Heavy-tailed prior distributions for
sequence count data: removing the noise and preserving large differences.
Bioinformatics. https://doi.org/10.1093/bioinformatics/bty895
using 'apeglm' for LFC shrinkage. If used in published research, please cite:
Zhu, A., Ibrahim, J.G., Love, M.I. (2018) Heavy-tailed prior distributions for
sequence count data: removing the noise and preserving large differences.
Bioinformatics. https://doi.org/10.1093/bioinformatics/bty895
using 'apeglm' for LFC shrinkage. If used in published research, please cite:
Zhu, A., Ibrahim, J.G., Love, M.I. (2018) Heavy-tailed prior distributions for
sequence count data: removing the noise and preserving large differences.
Bioinformatics. https://doi.org/10.1093/bioinformatics/bty895
using 'apeglm' for LFC shrinkage. If used in published research, please cite:
```

```
Zhu, A., Ibrahim, J.G., Love, M.I. (2018) Heavy-tailed prior distributions for
sequence count data: removing the noise and preserving large differences.
Bioinformatics. https://doi.org/10.1093/bioinformatics/bty895
using 'apeglm' for LFC shrinkage. If used in published research, please cite:
Zhu, A., Ibrahim, J.G., Love, M.I. (2018) Heavy-tailed prior distributions for
sequence count data: removing the noise and preserving large differences.
Bioinformatics. https://doi.org/10.1093/bioinformatics/bty895
Joining with `by = join_by(rowname)`
Joining with `by = join_by(rowname)`
Joining with `by = join_by(rowname)`
Joining with `by = join_by(rowname)`
Joining with `by = join_by(rowname)`
Joining with `by = join_by(name)`
Joining with `by = join_by(name)`
Joining with `by = join_by(name)`
Joining with `by = join_by(name)`
Joining with `by = join_by(name)`
found results columns, replacing these
using 'apeglm' for LFC shrinkage. If used in published research, please cite:
Zhu, A., Ibrahim, J.G., Love, M.I. (2018) Heavy-tailed prior distributions for
sequence count data: removing the noise and preserving large differences.
Bioinformatics. https://doi.org/10.1093/bioinformatics/bty895
using 'apeglm' for LFC shrinkage. If used in published research, please cite:
Zhu, A., Ibrahim, J.G., Love, M.I. (2018) Heavy-tailed prior distributions for
sequence count data: removing the noise and preserving large differences.
Bioinformatics. https://doi.org/10.1093/bioinformatics/bty895
using 'apeglm' for LFC shrinkage. If used in published research, please cite:
Zhu, A., Ibrahim, J.G., Love, M.I. (2018) Heavy-tailed prior distributions for
sequence count data: removing the noise and preserving large differences.
Bioinformatics. https://doi.org/10.1093/bioinformatics/bty895
using 'apeglm' for LFC shrinkage. If used in published research, please cite:
Zhu, A., Ibrahim, J.G., Love, M.I. (2018) Heavy-tailed prior distributions for
sequence count data: removing the noise and preserving large differences.
Bioinformatics. https://doi.org/10.1093/bioinformatics/bty895
using 'apeglm' for LFC shrinkage. If used in published research, please cite:
Zhu, A., Ibrahim, J.G., Love, M.I. (2018) Heavy-tailed prior distributions for
sequence count data: removing the noise and preserving large differences.
Bioinformatics. https://doi.org/10.1093/bioinformatics/bty895
Joining with `by = join_by(rowname)`
Joining with `by = join_by(rowname)`
Joining with `by = join_by(rowname)`
Joining with `by = join_by(rowname)`
Joining with `by = join_by(rowname)`
```

```
Joining with `by = join_by(name)`
Joining with `by = join_by(name)`
Joining with `by = join_by(name)`
Joining with `by = join_by(name)`
Joining with `by = join_by(name)`
found results columns, replacing these
using 'apeglm' for LFC shrinkage. If used in published research, please cite:
Zhu, A., Ibrahim, J.G., Love, M.I. (2018) Heavy-tailed prior distributions for
sequence count data: removing the noise and preserving large differences.
Bioinformatics. https://doi.org/10.1093/bioinformatics/bty895
using 'apeglm' for LFC shrinkage. If used in published research, please cite:
Zhu, A., Ibrahim, J.G., Love, M.I. (2018) Heavy-tailed prior distributions for
sequence count data: removing the noise and preserving large differences.
Bioinformatics. https://doi.org/10.1093/bioinformatics/bty895
using 'apeglm' for LFC shrinkage. If used in published research, please cite:
Zhu, A., Ibrahim, J.G., Love, M.I. (2018) Heavy-tailed prior distributions for
sequence count data: removing the noise and preserving large differences.
Bioinformatics. https://doi.org/10.1093/bioinformatics/bty895
using 'apeglm' for LFC shrinkage. If used in published research, please cite:
Zhu, A., Ibrahim, J.G., Love, M.I. (2018) Heavy-tailed prior distributions for
sequence count data: removing the noise and preserving large differences.
Bioinformatics. https://doi.org/10.1093/bioinformatics/bty895
using 'apeglm' for LFC shrinkage. If used in published research, please cite:
Zhu, A., Ibrahim, J.G., Love, M.I. (2018) Heavy-tailed prior distributions for
sequence count data: removing the noise and preserving large differences.
Bioinformatics. https://doi.org/10.1093/bioinformatics/bty895
Joining with `by = join_by(rowname)`
Joining with `by = join_by(rowname)`
Joining with `by = join_by(rowname)`
Joining with `by = join_by(rowname)`
Joining with `by = join_by(rowname)`
Joining with `by = join_by(name)`
Joining with `by = join_by(name)`
Joining with `by = join_by(name)`
Joining with `by = join_by(name)`
Joining with `by = join_by(name)`
```

```r
group <- levels(dds.filt$group)
set_prefilt <- compare_reciprocal_contrasts(group,folder)
```

```
Warning: Removed 1 row containing non-finite outside the scale range
(`stat_density()`).
```

```r
saveRDS(set_prefilt,"BRCA_prefilt.rds")
```

Print resized diagnostic stacked bar chart for publication:

```r
make_diagnostic(set_prefilt)
```

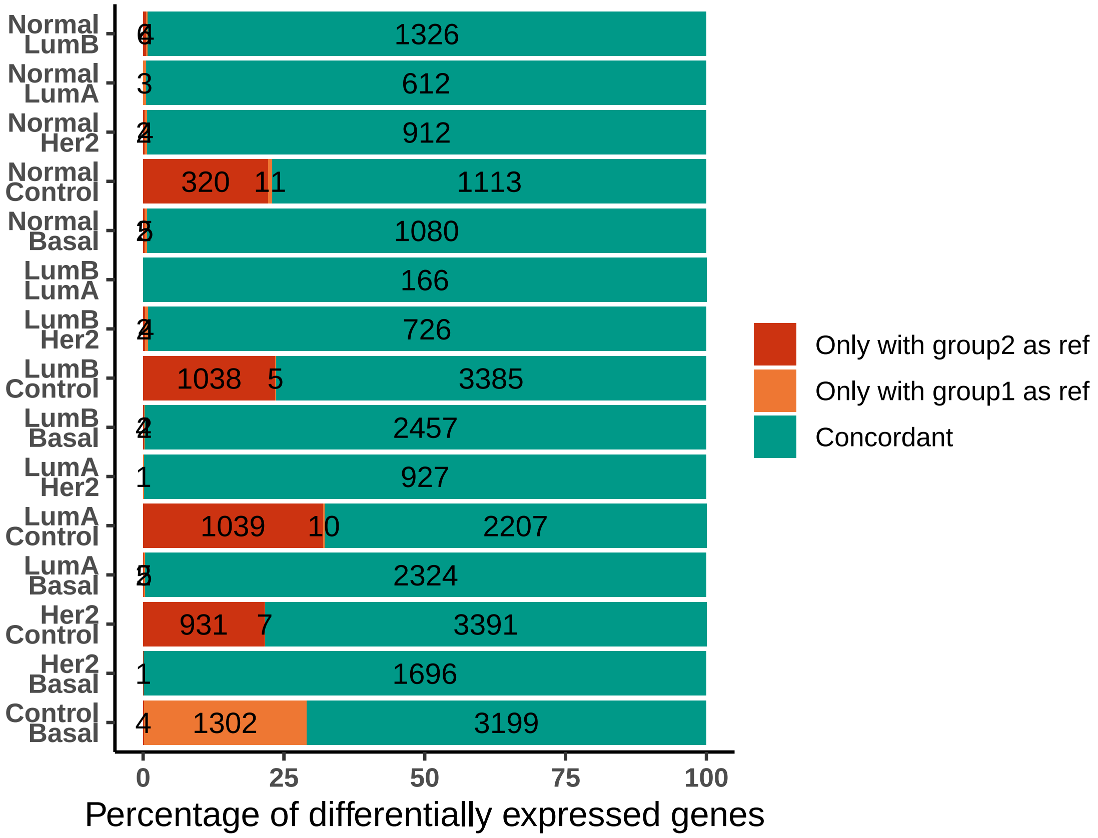


```r
rm(dds.filt)
rm(set_prefilt)
gc()
```

```
           used   (Mb) gc trigger   (Mb)   max used    (Mb)
Ncells  9011571  481.3   15175890  810.5   15175890   810.5
Vcells 17565690  134.1  842677707 6429.2 2251205837 17175.4
```

Finally, we try *surrogate variable analysis* (SVA) to remove unwanted/hidden sources of variation. The presence of unknown sources of variation can interfere with DGEA and increase

discordance in reciprocal contrasts, so we use SVA to see if we can identify these as per the method in Love et al. (2016), before running DESeq with mirrorCheck. This creates output csv tables and pdf reports in a folder alongside this qmd file.

```
folder <- "DESeq_sva"
dir.create(folder)
```

```
Warning in dir.create(folder): 'DESeq_sva' already exists
```

```
dds <- readRDS("quartodds.rds")
rowname2symbol = as.data.frame(rowData(dds)[, colnames(rowData(dds)) %in%
                                              c("gene_id","gene_name")]) %>%
  rename(rowname = gene_id,
         g_symbol = gene_name)
dds <- DESeq(dds)
```

```
estimating size factors
```

```
estimating dispersions
```

```
gene-wise dispersion estimates
```

```
mean-dispersion relationship
```

```
final dispersion estimates
```

```
fitting model and testing
```

```
-- replacing outliers and refitting for 9848 genes
-- DESeq argument 'minReplicatesForReplace' = 7
-- original counts are preserved in counts(dds)
```

```
estimating dispersions
```

```
fitting model and testing
```

```
dat <- counts(dds, normalized=TRUE)
idx <- rowMeans(dat) > 1
dat <- dat[idx,]
mod <- model.matrix(~ group, colData(dds))
mod0 <- model.matrix(~ 1, colData(dds))

#find probable number of unwanted variables
num_sv <- num.sv(dat,mod,method = "be")
num_sv
```

```
[1] 4
```

```
#num_sv = 4
svseq <- svaseq(dat, mod, mod0, n.sv=num_sv)
```

```
Number of significant surrogate variables is:  4
Iteration (out of 5 ):1  2  3  4  5
```

```
dds.sva <- dds
dds.sva$SV1 <- svseq$sv[,1]
dds.sva$SV2 <- svseq$sv[,2]
dds.sva$SV3 <- svseq$sv[,3]
dds.sva$SV4 <- svseq$sv[,4]
design(dds.sva) <- ~ SV1 + SV2 + SV3 + SV4 + group

run_DESeq_all_contrasts(dds.sva,folder,
                        condition = "group",
                        p.cutoff = 0.01,
                        top.n = 0,
                        print.all = T,
                        useDingbats = T)
```

```
using pre-existing size factors

estimating dispersions

found already estimated dispersions, replacing these

gene-wise dispersion estimates
```

```
mean-dispersion relationship

final dispersion estimates

fitting model and testing

362 rows did not converge in beta, labelled in mcols(object)$betaConv. Use larger maxit argu

using 'apeglm' for LFC shrinkage. If used in published research, please cite:
    Zhu, A., Ibrahim, J.G., Love, M.I. (2018) Heavy-tailed prior distributions for
    sequence count data: removing the noise and preserving large differences.
    Bioinformatics. https://doi.org/10.1093/bioinformatics/bty895
using 'apeglm' for LFC shrinkage. If used in published research, please cite:
    Zhu, A., Ibrahim, J.G., Love, M.I. (2018) Heavy-tailed prior distributions for
    sequence count data: removing the noise and preserving large differences.
    Bioinformatics. https://doi.org/10.1093/bioinformatics/bty895
using 'apeglm' for LFC shrinkage. If used in published research, please cite:
    Zhu, A., Ibrahim, J.G., Love, M.I. (2018) Heavy-tailed prior distributions for
    sequence count data: removing the noise and preserving large differences.
    Bioinformatics. https://doi.org/10.1093/bioinformatics/bty895
using 'apeglm' for LFC shrinkage. If used in published research, please cite:
    Zhu, A., Ibrahim, J.G., Love, M.I. (2018) Heavy-tailed prior distributions for
    sequence count data: removing the noise and preserving large differences.
    Bioinformatics. https://doi.org/10.1093/bioinformatics/bty895
using 'apeglm' for LFC shrinkage. If used in published research, please cite:
    Zhu, A., Ibrahim, J.G., Love, M.I. (2018) Heavy-tailed prior distributions for
    sequence count data: removing the noise and preserving large differences.
    Bioinformatics. https://doi.org/10.1093/bioinformatics/bty895

found results columns, replacing these

234 rows did not converge in beta, labelled in mcols(object)$betaConv. Use larger maxit argu

using 'apeglm' for LFC shrinkage. If used in published research, please cite:
    Zhu, A., Ibrahim, J.G., Love, M.I. (2018) Heavy-tailed prior distributions for
    sequence count data: removing the noise and preserving large differences.
    Bioinformatics. https://doi.org/10.1093/bioinformatics/bty895
using 'apeglm' for LFC shrinkage. If used in published research, please cite:
    Zhu, A., Ibrahim, J.G., Love, M.I. (2018) Heavy-tailed prior distributions for
    sequence count data: removing the noise and preserving large differences.
```

```
    Bioinformatics. https://doi.org/10.1093/bioinformatics/bty895
using 'apeglm' for LFC shrinkage. If used in published research, please cite:
    Zhu, A., Ibrahim, J.G., Love, M.I. (2018) Heavy-tailed prior distributions for
    sequence count data: removing the noise and preserving large differences.
    Bioinformatics. https://doi.org/10.1093/bioinformatics/bty895
using 'apeglm' for LFC shrinkage. If used in published research, please cite:
    Zhu, A., Ibrahim, J.G., Love, M.I. (2018) Heavy-tailed prior distributions for
    sequence count data: removing the noise and preserving large differences.
    Bioinformatics. https://doi.org/10.1093/bioinformatics/bty895
using 'apeglm' for LFC shrinkage. If used in published research, please cite:
    Zhu, A., Ibrahim, J.G., Love, M.I. (2018) Heavy-tailed prior distributions for
    sequence count data: removing the noise and preserving large differences.
    Bioinformatics. https://doi.org/10.1093/bioinformatics/bty895

found results columns, replacing these

364 rows did not converge in beta, labelled in mcols(object)$betaConv. Use larger maxit argu

using 'apeglm' for LFC shrinkage. If used in published research, please cite:
    Zhu, A., Ibrahim, J.G., Love, M.I. (2018) Heavy-tailed prior distributions for
    sequence count data: removing the noise and preserving large differences.
    Bioinformatics. https://doi.org/10.1093/bioinformatics/bty895
using 'apeglm' for LFC shrinkage. If used in published research, please cite:
    Zhu, A., Ibrahim, J.G., Love, M.I. (2018) Heavy-tailed prior distributions for
    sequence count data: removing the noise and preserving large differences.
    Bioinformatics. https://doi.org/10.1093/bioinformatics/bty895
using 'apeglm' for LFC shrinkage. If used in published research, please cite:
    Zhu, A., Ibrahim, J.G., Love, M.I. (2018) Heavy-tailed prior distributions for
    sequence count data: removing the noise and preserving large differences.
    Bioinformatics. https://doi.org/10.1093/bioinformatics/bty895
using 'apeglm' for LFC shrinkage. If used in published research, please cite:
    Zhu, A., Ibrahim, J.G., Love, M.I. (2018) Heavy-tailed prior distributions for
    sequence count data: removing the noise and preserving large differences.
    Bioinformatics. https://doi.org/10.1093/bioinformatics/bty895
using 'apeglm' for LFC shrinkage. If used in published research, please cite:
    Zhu, A., Ibrahim, J.G., Love, M.I. (2018) Heavy-tailed prior distributions for
    sequence count data: removing the noise and preserving large differences.
    Bioinformatics. https://doi.org/10.1093/bioinformatics/bty895

found results columns, replacing these

52 rows did not converge in beta, labelled in mcols(object)$betaConv. Use larger maxit argum
```

```
using 'apeglm' for LFC shrinkage. If used in published research, please cite:
    Zhu, A., Ibrahim, J.G., Love, M.I. (2018) Heavy-tailed prior distributions for
    sequence count data: removing the noise and preserving large differences.
    Bioinformatics. https://doi.org/10.1093/bioinformatics/bty895
using 'apeglm' for LFC shrinkage. If used in published research, please cite:
    Zhu, A., Ibrahim, J.G., Love, M.I. (2018) Heavy-tailed prior distributions for
    sequence count data: removing the noise and preserving large differences.
    Bioinformatics. https://doi.org/10.1093/bioinformatics/bty895
using 'apeglm' for LFC shrinkage. If used in published research, please cite:
    Zhu, A., Ibrahim, J.G., Love, M.I. (2018) Heavy-tailed prior distributions for
    sequence count data: removing the noise and preserving large differences.
    Bioinformatics. https://doi.org/10.1093/bioinformatics/bty895
using 'apeglm' for LFC shrinkage. If used in published research, please cite:
    Zhu, A., Ibrahim, J.G., Love, M.I. (2018) Heavy-tailed prior distributions for
    sequence count data: removing the noise and preserving large differences.
    Bioinformatics. https://doi.org/10.1093/bioinformatics/bty895
using 'apeglm' for LFC shrinkage. If used in published research, please cite:
    Zhu, A., Ibrahim, J.G., Love, M.I. (2018) Heavy-tailed prior distributions for
    sequence count data: removing the noise and preserving large differences.
    Bioinformatics. https://doi.org/10.1093/bioinformatics/bty895

found results columns, replacing these

222 rows did not converge in beta, labelled in mcols(object)$betaConv. Use larger maxit argu

using 'apeglm' for LFC shrinkage. If used in published research, please cite:
    Zhu, A., Ibrahim, J.G., Love, M.I. (2018) Heavy-tailed prior distributions for
    sequence count data: removing the noise and preserving large differences.
    Bioinformatics. https://doi.org/10.1093/bioinformatics/bty895
using 'apeglm' for LFC shrinkage. If used in published research, please cite:
    Zhu, A., Ibrahim, J.G., Love, M.I. (2018) Heavy-tailed prior distributions for
    sequence count data: removing the noise and preserving large differences.
    Bioinformatics. https://doi.org/10.1093/bioinformatics/bty895
using 'apeglm' for LFC shrinkage. If used in published research, please cite:
    Zhu, A., Ibrahim, J.G., Love, M.I. (2018) Heavy-tailed prior distributions for
    sequence count data: removing the noise and preserving large differences.
    Bioinformatics. https://doi.org/10.1093/bioinformatics/bty895
using 'apeglm' for LFC shrinkage. If used in published research, please cite:
    Zhu, A., Ibrahim, J.G., Love, M.I. (2018) Heavy-tailed prior distributions for
    sequence count data: removing the noise and preserving large differences.
    Bioinformatics. https://doi.org/10.1093/bioinformatics/bty895
using 'apeglm' for LFC shrinkage. If used in published research, please cite:
```

```
    Zhu, A., Ibrahim, J.G., Love, M.I. (2018) Heavy-tailed prior distributions for
    sequence count data: removing the noise and preserving large differences.
    Bioinformatics. https://doi.org/10.1093/bioinformatics/bty895

found results columns, replacing these

356 rows did not converge in beta, labelled in mcols(object)$betaConv. Use larger maxit argu

using 'apeglm' for LFC shrinkage. If used in published research, please cite:
    Zhu, A., Ibrahim, J.G., Love, M.I. (2018) Heavy-tailed prior distributions for
    sequence count data: removing the noise and preserving large differences.
    Bioinformatics. https://doi.org/10.1093/bioinformatics/bty895
using 'apeglm' for LFC shrinkage. If used in published research, please cite:
    Zhu, A., Ibrahim, J.G., Love, M.I. (2018) Heavy-tailed prior distributions for
    sequence count data: removing the noise and preserving large differences.
    Bioinformatics. https://doi.org/10.1093/bioinformatics/bty895
using 'apeglm' for LFC shrinkage. If used in published research, please cite:
    Zhu, A., Ibrahim, J.G., Love, M.I. (2018) Heavy-tailed prior distributions for
    sequence count data: removing the noise and preserving large differences.
    Bioinformatics. https://doi.org/10.1093/bioinformatics/bty895
using 'apeglm' for LFC shrinkage. If used in published research, please cite:
    Zhu, A., Ibrahim, J.G., Love, M.I. (2018) Heavy-tailed prior distributions for
    sequence count data: removing the noise and preserving large differences.
    Bioinformatics. https://doi.org/10.1093/bioinformatics/bty895
using 'apeglm' for LFC shrinkage. If used in published research, please cite:
    Zhu, A., Ibrahim, J.G., Love, M.I. (2018) Heavy-tailed prior distributions for
    sequence count data: removing the noise and preserving large differences.
    Bioinformatics. https://doi.org/10.1093/bioinformatics/bty895
```

```r
group <- levels(as.factor(dds.sva$group))
set_sva <- compare_reciprocal_contrasts(group,folder)
```

```
Warning: Removed 32 rows containing non-finite outside the scale range
(`stat_density()`).
```

```r
saveRDS(set_sva,"BRCA_sva.rds")
```

Print resized diagnostic stacked bar chart for publication:

```r
make_diagnostic(set_sva)
```

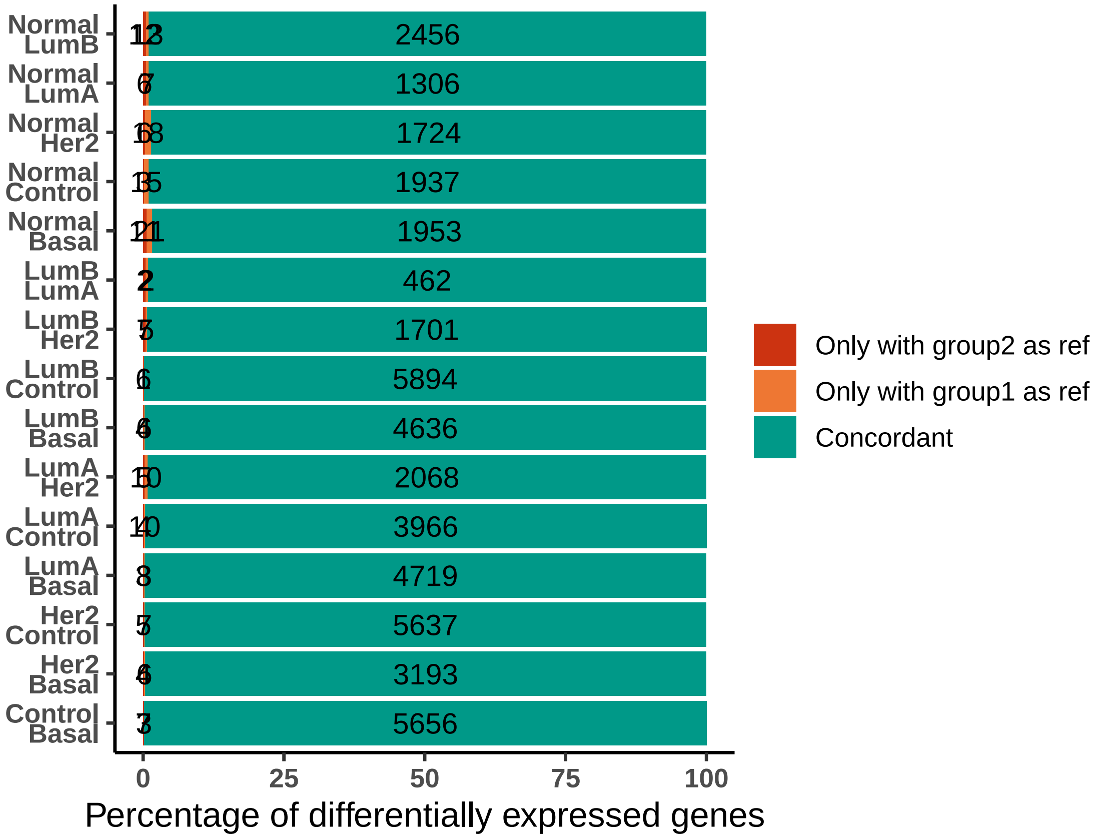


```r
rm(list = ls())
gc()
```

```
           used  (Mb) gc trigger    (Mb)   max used    (Mb)
Ncells  8911340 476.0   15175895   810.5   15175895   810.5
Vcells 17806538 135.9 1843218620 14062.7 2577630939 19665.8
```

Question: How does this compare to edgeR? Are the discordant DEGs found to be DEGs with edgeR? Are the concordant DEGs also found with edgeR?

edgeR is another well-regarded tool for differential expression analysis. Filtering the data to eliminate low count genes is an important part of the standard edgeR workflow, and edgeR includes a specialised function to help make the filtering more data-dependent and sophisticated (which is why we borrowed it to test prefiltering of the dds, above). While edgeR does

adjust the raw log fold change values using a "prior count" strategy, this is not intended to replace filtration. Here (and in the associated Quartos) we ran edgeR as recommended and with default parameters and did not observe discordant results for reciprocal contrasts. Therefore, after checking this is true, we compare two sets of DESeq2 results for a reciprocal contrast to the only set of edgeR DEGs. We picked a contrast with some discordance in the DESeq2 reciprocal results.

Run edgeR

```
folder <- "edgeR_for_pub"
dir.create(folder)
```

```
Warning in dir.create(folder): 'edgeR_for_pub' already exists
```

First, LumB v Control:

```
dds <- readRDS("quartodds.rds")
group <- dds$group
mat <- counts(dds)
rm(dds)
gc()
```

```
           used  (Mb) gc trigger    (Mb)   max used    (Mb)
Ncells  8950453 478.1   15175895   810.5   15175895   810.5
Vcells 54793174 418.1 1474574896 11250.2 2577630939 19665.8
```

```
y <- DGEList(counts=mat,group=group)
dim(y$counts)
```

```
[1] 60660  1212
```

```
keep <- filterByExpr(y)
y <- y[keep,,keep.lib.sizes=FALSE]
dim(y$counts)
```

```
[1] 30954  1212
```

```r
y <- normLibSizes(y)

design <- model.matrix(~group)
rownames(design) <- colnames(y)

y <- estimateDisp(y, design)
fit <- glmQLFit(y,design)
colnames(fit$design)
```

```
[1] "(Intercept)" "groupBasal"  "groupHer2"   "groupLumA"   "groupLumB"
[6] "groupNormal"
```

```r
# coef = 5 is LumB vs Control
qlf <- glmQLFTest(fit,coef=5)
result_LumBvsControl <- topTags(qlf, n=nrow(y))
# check for missing adjusted p-values
any(is.na(as.data.frame(result_LumBvsControl)$FDR))
```

```
[1] FALSE
```

```r
# Adapt mirrorCheck's (private) plot_Volcano() to work for edgeR results
plot_Volcano_edgeR <- function(r,title, rowname2symbol = NULL,
                         p.cutoff = 0.1, fc.cutoff = 2, top.n = 30) {
  padj <- as.data.frame(r) %>%
    dplyr::filter(!is.na(FDR)) %>%
    dplyr::mutate(log10padj = -log10(FDR),
                  diff.expressed = dplyr::case_when(
                    logFC > fc.cutoff & FDR < p.cutoff ~ "UP",
                    logFC < -fc.cutoff & FDR < p.cutoff ~ "DOWN",
                    .default = "NO")) %>%
    tibble::rownames_to_column("rowname")
  if (is.null(rowname2symbol)) {
    padj <- padj %>%
      dplyr::mutate(g_symbol = rowname)
  } else {
    padj <- padj  %>%
      dplyr::left_join(rowname2symbol)
  }
  diff.ex <- padj %>% dplyr::filter(diff.expressed != "NO")
```

```r
  sums <- table(diff.ex$diff.expressed)
  maxy <- max(padj$log10padj)
  minx <- min(padj$logFC)
  maxx <- max(padj$logFC)
  padj$labels <- ifelse(padj$rowname %in% head(diff.ex[order(diff.ex$FDR),
                                                        "rowname"], top.n),
                        padj$g_symbol, NA)
  group.colors <- c(DOWN = '#CC3311',NO = "grey",UP = '#009988')
  group.labels <- c(DOWN = "Downregulated",
                    NO = "Not significant",
                    UP = "Upregulated")

  p <- ggplot2::ggplot(padj, ggplot2::aes(x = logFC,
                                          y = log10padj,
                                          col = diff.expressed,
                                          label = labels)) +
    ggplot2::theme_classic(base_size = 14) +
    ggplot2::labs(title = title,
                  x = expression("log"[2]*"FC"),
                  y = expression("-log"[10]*"adj-pvalue")) +
    ggplot2::theme(legend.title = element_blank()) +
    ggplot2::geom_vline(xintercept = c(-fc.cutoff, fc.cutoff),
                        col = "gray", linetype = 'dashed') +
    ggplot2::geom_hline(yintercept = -log10(p.cutoff),
                        col = "gray", linetype = 'dashed') +
    ggplot2::geom_point(size = 1.5) +
    ggplot2::scale_color_manual(values = group.colors,
                                labels = group.labels) +
    ggrepel::geom_text_repel(max.overlaps = Inf, size = 3,
                             color = "black", na.rm = T) +
    ggplot2::annotate("text",x = minx + 3, y = maxy - 1,
                      label = paste("DOWN",sums["DOWN"]),size = 4.5) +
    ggplot2::annotate("text",x = maxx - 2, y = maxy - 1,
                      label = paste("UP",sums["UP"]), size = 4.5)
}

p <- plot_Volcano_edgeR(result_LumBvsControl, "LumB_vs_Control edgeR",
                        p.cutoff = 0.01,
                        top.n = 0)
p
```

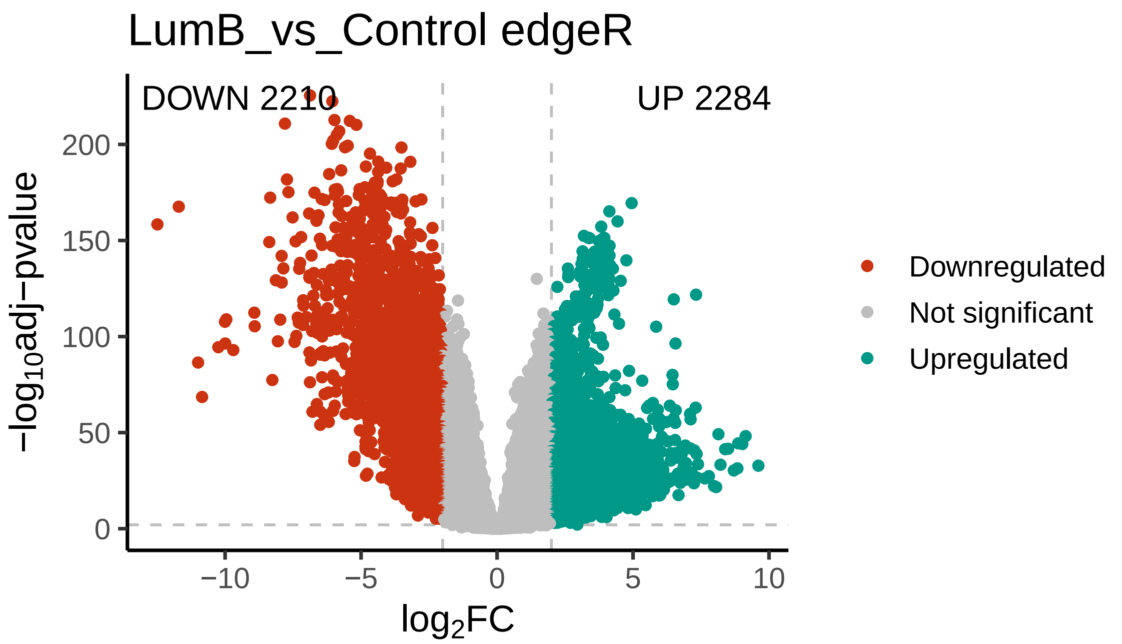

Second, relevel for comparison to LumB

```
group <- relevel(group, ref = "LumB")
levels(group)
```

```
[1] "LumB"    "Control" "Basal"   "Her2"    "LumA"    "Normal"
```

```
y <- DGEList(counts=mat,group=group)
dim(y$counts)
```

```
[1] 60660  1212
```

```
keep <- filterByExpr(y)
y <- y[keep,,keep.lib.sizes=FALSE]
dim(y$counts)
```

```
[1] 30954  1212
```

```
y <- normLibSizes(y)

design <- model.matrix(~group)
rownames(design) <- colnames(y)

y <- estimateDisp(y, design)
fit <- glmQLFit(y,design)
colnames(fit$design)
```

```
[1] "(Intercept)"  "groupControl" "groupBasal"   "groupHer2"    "groupLumA"
[6] "groupNormal"
```

```
# coef = 2 is  Control vs LumB
qlf2 <- glmQLFTest(fit,coef=2)
result_ControlvsLumB <- topTags(qlf2, n=nrow(y))
# check for missing adjusted p-values
any(is.na(as.data.frame(result_ControlvsLumB)$FDR))
```

```
[1] FALSE
```

```
p <- plot_Volcano_edgeR(result_ControlvsLumB, "Control_vs_LumB edgeR",
                        p.cutoff = 0.01,
                        top.n = 0)
p
```

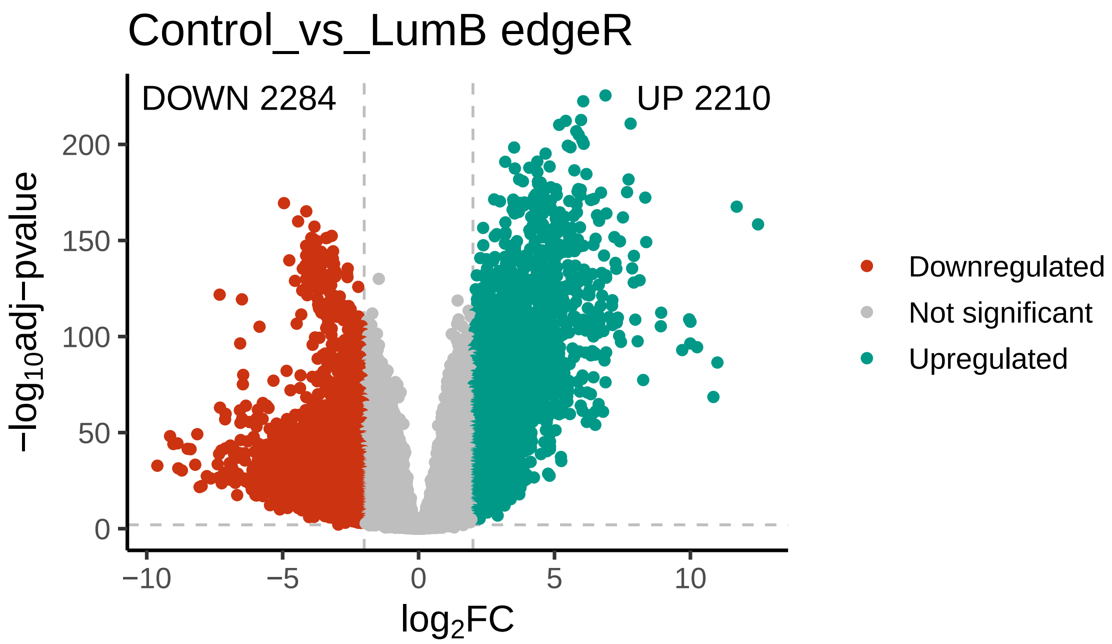


```
# Write results tables
write.csv(as.data.frame(result_LumBvsControl),
          file = file.path(folder,"LumBvsControl.csv"))
write.csv(as.data.frame(result_ControlvsLumB),
          file = file.path(folder,"ControlvsLumB.csv"))

rm(y,fit,qlf,qlf2)
gc()
```

```
           used  (Mb) gc trigger   (Mb)   max used    (Mb)
Ncells  8986817 480.0   15175895  810.5   15175895   810.5
Vcells 55146180 420.8  756066828 5768.4 2577630939 19665.8
```

Check: these results are perfectly mirrored so they give the same set of DEGs at FDR < 0.01 and logFC > 2 | logFC < 2

```
deg_LvC <- as.data.frame(result_LumBvsControl) %>%
  filter(FDR < 0.01 & (logFC < -2 | logFC > 2)) %>%
  rownames_to_column("name") %>%
  pull(name)
str(deg_LvC)
```

```
 chr [1:4494] "ENSG00000277737.3" "ENSG00000286322.1" "ENSG00000186466.5" ...
```

```
deg_CvL <- as.data.frame(result_ControlvsLumB) %>%
  filter(FDR < 0.01 & (logFC < -2 | logFC > 2)) %>%
  rownames_to_column("name") %>%
  pull(name)
all(deg_CvL == deg_LvC)
```

```
[1] TRUE
```

```
edger_DEG <- deg_LvC
```

DESeq2 DEGs compared to edgeR DEGs - without prefiltering dds

```
deseq_LvC <- read.csv(file.path("DESeq_noclean","LumB_vs_Control_DG.csv"))
table(deseq_LvC$padj < 0.01 &
        (deseq_LvC$log2FoldChange < -2 | deseq_LvC$log2FoldChange > 2))
```

```
TRUE
5470
```

```
deseq_CvL <- read.csv(file.path("DESeq_noclean","Control_vs_LumB_DG.csv"))
table(deseq_CvL$padj < 0.01 &
        (deseq_CvL$log2FoldChange < -2 | deseq_CvL$log2FoldChange > 2))
```

```
TRUE
4177
```

```
deseq_LvC_deg <- deseq_LvC %>% pull(name)
deseq_CvL_deg <- deseq_CvL %>% pull(name)

#unfiltered upset
list_with_unfiltdeseq <- list(edgeR = edger_DEG,
                              DESeq2_vControl = deseq_LvC_deg,
                              DESeq2_vLumB = deseq_CvL_deg)
str(list_with_unfiltdeseq)
```

```
List of 3
 $ edgeR          : chr [1:4494] "ENSG00000277737.3" "ENSG00000286322.1" "ENSG00000186466.5"
 $ DESeq2_vControl: chr [1:5470] "ENSG00000077152.11" "ENSG00000088325.16" "ENSG00000090889.
 $ DESeq2_vLumB   : chr [1:4177] "ENSG00000077152.11" "ENSG00000088325.16" "ENSG00000090889.
```

```
# the following function was sourced from github UpsetR issue 85, solution
# provided by 'docmanny' in September 2017
newFromList <- function (input) {
  # Same as original fromList()...
  elements <- unique(unlist(input))
  data <- unlist(lapply(input, function(x) {
    x <- as.vector(match(elements, x))
  }))
  data[is.na(data)] <- as.integer(0)
  data[data != 0] <- as.integer(1)
  data <- data.frame(matrix(data, ncol = length(input), byrow = F))
  data <- data[which(rowSums(data) != 0), ]
  names(data) <- names(input)
  # ... Except now it conserves your original value names!
  row.names(data) <- elements
  return(data)
}

for_upset_unfilt <- newFromList(list_with_unfiltdeseq) %>%
  mutate(concordant = if_else(DESeq2_vControl == 1 & DESeq2_vLumB == 1, T, F))

upset_unfilt <- UpSetR::upset(for_upset_unfilt,
                              sets = c("edgeR",
                                       "DESeq2_vControl",
                                       "DESeq2_vLumB"),
                              nintersects = NA,
                              mainbar.y.label = "DEGs per\nintersection",
                              sets.x.label = "Results set",
                              text.scale = c(2,2,2,1.5,2,2),
                              empty.intersections = T,
                              keep.order = T,
                              mb.ratio = c(0.65, 0.35))
upset_unfilt
```

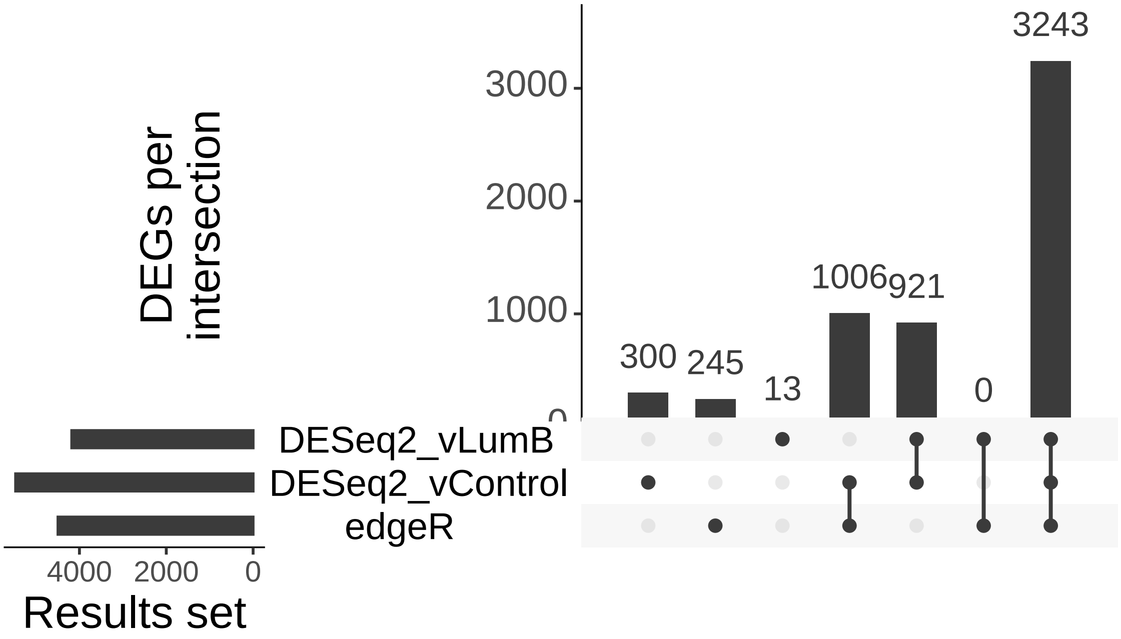

DESeq2 DEGs compared to edgeR DEGs - prefiltering dds

```
deseq_LvC_filt <- read.csv(file.path("DESeq_prefilt","LumB_vs_Control_DG.csv"))
table(deseq_LvC_filt$padj < 0.01 & (deseq_LvC_filt$log2FoldChange < -2 |
                                    deseq_LvC_filt$log2FoldChange > 2))
```

```
TRUE
4423
```

```
deseq_CvL_filt <- read.csv(file.path("DESeq_prefilt","Control_vs_LumB_DG.csv"))
table(deseq_CvL_filt$padj < 0.01 & (deseq_CvL_filt$log2FoldChange < -2 |
                                    deseq_CvL_filt$log2FoldChange > 2))
```

```
TRUE
3390
```

```r
deseq_LvC_deg_filt <- deseq_LvC_filt %>% pull(name)
deseq_CvL_deg_filt <- deseq_CvL_filt %>% pull(name)

#filtered upset
list_with_filtdeseq <- list(edgeR = edger_DEG,
                            DESeq2_vControl = deseq_LvC_deg_filt,
                            DESeq2_vLumB = deseq_CvL_deg_filt)
str(list_with_filtdeseq)
```

```
List of 3
 $ edgeR          : chr [1:4494] "ENSG00000277737.3" "ENSG00000286322.1" "ENSG00000186466.5"
 $ DESeq2_vControl: chr [1:4423] "ENSG00000077152.11" "ENSG00000088325.16" "ENSG00000090889.
 $ DESeq2_vLumB   : chr [1:3390] "ENSG00000077152.11" "ENSG00000088325.16" "ENSG00000090889.
```

```r
for_upset_filt <- newFromList(list_with_filtdeseq) %>%
  mutate(concordant = if_else(DESeq2_vControl == 1 & DESeq2_vLumB == 1, T, F))

upset_filt <- UpSetR::upset(for_upset_filt, sets = c("edgeR",
                                                     "DESeq2_vControl",
                                                     "DESeq2_vLumB"),
                            nintersects = NA,
                            mainbar.y.label = "DEGs per\nintersection",
                            sets.x.label = "Results set",
                            text.scale = c(2,2,2,1.2,2,2),
                            empty.intersections = T,
                            keep.order = T,
                            mb.ratio = c(0.65, 0.35))
upset_filt
```

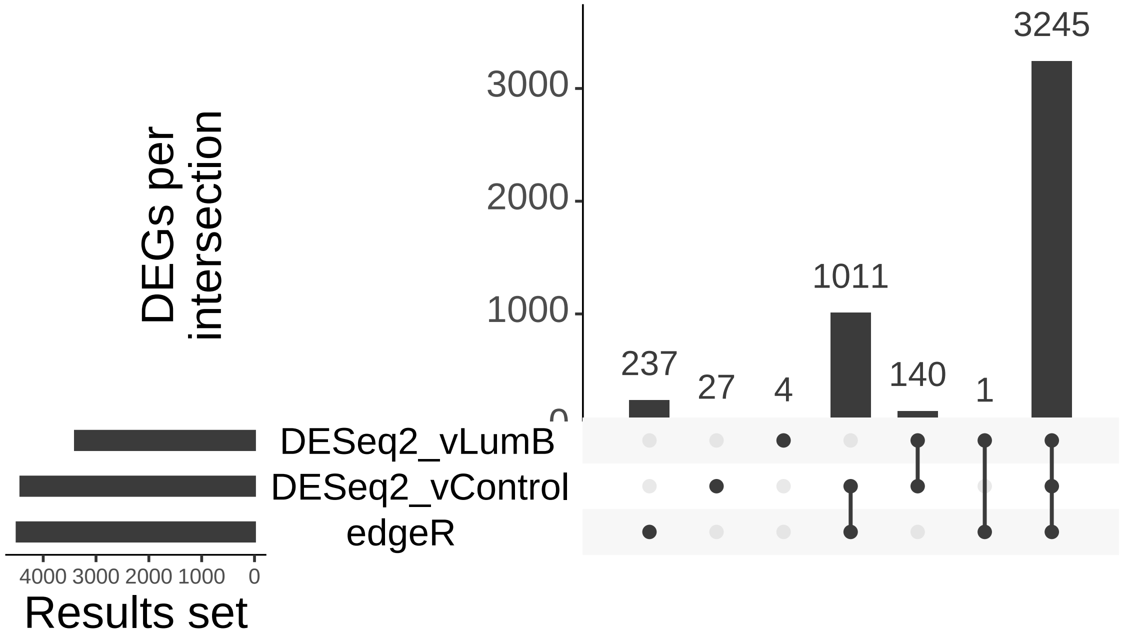

DESeq2 DEGs from prefiltered and non-prefiltered dds compared to edgeR DEGs on the same UpSet plot, with gene expression levels

```
list_with_bothdeseq <- list(edgeR = edger_DEG,
                            vControl = deseq_LvC_deg,
                            vLumB = deseq_CvL_deg,
                            vControl_filt = deseq_LvC_deg_filt,
                            vLumB_filt = deseq_CvL_deg_filt)
str(list_with_bothdeseq)
```

```
List of 5
 $ edgeR        : chr [1:4494] "ENSG00000277737.3" "ENSG00000286322.1" "ENSG00000186466.5" "I
 $ vControl     : chr [1:5470] "ENSG00000077152.11" "ENSG00000088325.16" "ENSG00000090889.12
 $ vLumB        : chr [1:4177] "ENSG00000077152.11" "ENSG00000088325.16" "ENSG00000090889.12
 $ vControl_filt: chr [1:4423] "ENSG00000077152.11" "ENSG00000088325.16" "ENSG00000090889.12
 $ vLumB_filt   : chr [1:3390] "ENSG00000077152.11" "ENSG00000088325.16" "ENSG00000090889.12
```

```
for_upset_both <- newFromList(list_with_bothdeseq) %>%
  mutate(Concordant = if_else((vControl == 1 & vLumB == 1) |
                              (vControl_filt == 1 & vLumB_filt == 1),
```

```
                              T, F))
write.csv(for_upset_both,
              file = file.path(folder,"DESeq_edger_DEG_intersections.csv"),
              na="")

#Get results to extract mean normalised counts (baseMeans) for genes across
#samples
dds <- readRDS("quartodds.rds")
dds <- DESeq(dds)
```

```
estimating size factors

estimating dispersions

gene-wise dispersion estimates

mean-dispersion relationship

final dispersion estimates

fitting model and testing

-- replacing outliers and refitting for 9848 genes
-- DESeq argument 'minReplicatesForReplace' = 7
-- original counts are preserved in counts(dds)

estimating dispersions

fitting model and testing
```

```
res <- results(dds)
baseMeans <- as.data.frame(res) %>%
  rownames_to_column("name") %>%
  select(name,baseMean)
head(baseMeans)
```

```
                 name  baseMean
1 ENSG00000000003.15 3101.7306
2  ENSG00000000005.6  155.5862
3 ENSG00000000419.13 2300.5750
4 ENSG00000000457.14 1569.0616
5 ENSG00000000460.17  694.7248
6 ENSG00000000938.13  580.4270
```

```
withNames <- for_upset_both %>% rownames_to_column("name")
forComplex <- withNames %>% left_join(baseMeans)
```

```
Joining with `by = join_by(name)`
```

```
head(forComplex)
```

```
                name edgeR vControl vLumB vControl_filt vLumB_filt Concordant
1 ENSG00000277737.3     1        1     1             1          1       TRUE
2 ENSG00000286322.1     1        1     1             1          1       TRUE
3 ENSG00000186466.5     1        1     1             1          1       TRUE
4 ENSG00000226005.5     1        1     1             1          1       TRUE
5 ENSG00000174429.4     1        1     0             1          0      FALSE
6 ENSG00000276365.1     1        1     1             1          1       TRUE
   baseMean
1 22.667343
2 24.011559
3 20.576072
4 13.613743
5  2.557098
6 17.915798
```

```
my_comparisons <- list( c(5,17), c(14,16), c(14,17))

ComplexUpset::upset(forComplex,c("edgeR",
                                  "vControl",
                                  "vLumB",
                                  "vControl_filt",
                                  "vLumB_filt"),
                    name = element_blank(),
                    sort_intersections_by=c("degree","ratio"),
                    sort_intersections = "ascending",
```

```
          sort_sets=F,
          annotations = list(
            "log10 mean of\nnormalised counts"=list(
              aes=aes(x=intersection, y=baseMean),
              geom=list(geom_boxplot(),
                        scale_y_continuous(trans='log10'),
                        expand_limits(y = 100000000),
                        stat_compare_means(comparisons=my_comparisons,
                                           label = "p.signif",
                                           size = 4,
                                           label.y = c(5,6,7,8)),
                        theme(axis.text.y = element_text(size = 10),
                              axis.title.y=element_text(face="bold"))
                        )
            )
          ),
          base_annotations=list(
            'DEGs\nper intersection'=intersection_size(
              mapping=aes(fill=Concordant),
              text_colors = c(on_background = "black",
                              on_bar = "black"),
              text=list(size = 3)) +
              scale_fill_manual(values =c("TRUE" = "#009988",
                                          "FALSE" = "black")) +
              theme(axis.text.y = element_text(size = 10),
                    axis.title.y = element_text(face="bold"))
          ),
          set_sizes=(
            upset_set_size(position='right')
            + ylab("DEGs per set")
          ),
          themes=upset_modify_themes(
            list('intersections_matrix'=theme(
              axis.text.y=element_text(size=12),
              ))
            ),

          width_ratio=0.2,
          height_ratio=0.9,
          guides='over'
        ) & theme(legend.margin=margin(margin(t = 180, r = 0,
                                             b = 0, l = 0,
```

```
                                          unit = "pt")))
```

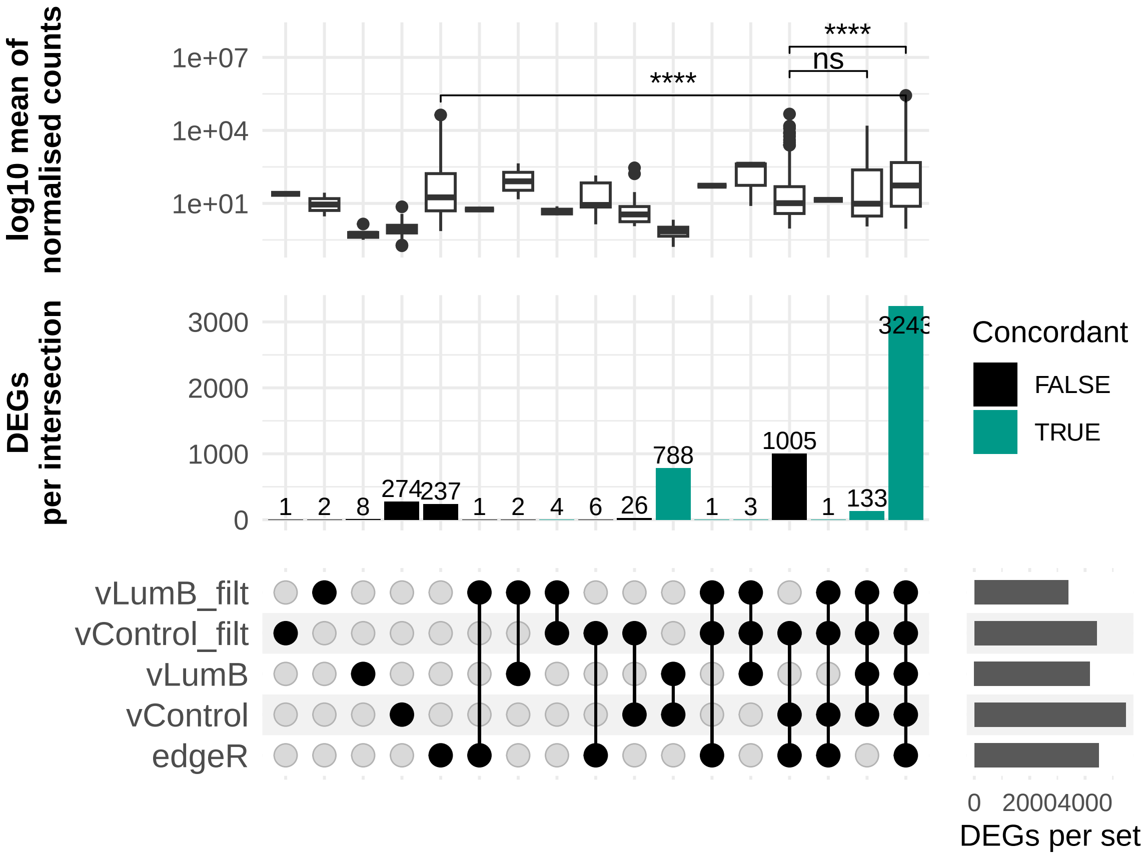

Session info and citations

Love, M. I., Anders, S., Kim, V., & Huber, W. (2016). RNA-Seq workflow: gene-level exploratory analysis and differential expression. F1000Research 2016 4:1070, 4, 1070. https://doi.org/10.12688/f1000research.7035.2


```
citation("DESeq2")
```

```
To cite package 'DESeq2' in publications use:

  Love, M.I., Huber, W., Anders, S. Moderated estimation of fold change
  and dispersion for RNA-seq data with DESeq2 Genome Biology 15(12):550
  (2014)
```

```
A BibTeX entry for LaTeX users is

  @Article{,
    title = {Moderated estimation of fold change and dispersion for RNA-seq data with DESeq2}
    author = {Michael I. Love and Wolfgang Huber and Simon Anders},
    year = {2014},
    journal = {Genome Biology},
    doi = {10.1186/s13059-014-0550-8},
    volume = {15},
    issue = {12},
    pages = {550},
  }
```

```r
citation("edgeR")
```

```
See Section 1.2 in the User's Guide for more detail about how to cite
the different edgeR pipelines.

  Chen Y, Chen L, Lun ATL, Baldoni PL, Smyth GK (2024). edgeR 4.0:
  powerful differential analysis of sequencing data with expanded
  functionality and improved support for small counts and larger
  datasets. bioRxiv doi: 10.1101/2024.01.21.576131

  Chen Y, Lun ATL, Smyth GK (2016). From reads to genes to pathways:
  differential expression analysis of RNA-Seq experiments using
  Rsubread and the edgeR quasi-likelihood pipeline. F1000Research 5,
  1438

  McCarthy DJ, Chen Y and Smyth GK (2012). Differential expression
  analysis of multifactor RNA-Seq experiments with respect to
  biological variation. Nucleic Acids Research 40(10), 4288-4297

  Robinson MD, McCarthy DJ and Smyth GK (2010). edgeR: a Bioconductor
  package for differential expression analysis of digital gene
  expression data. Bioinformatics 26(1), 139-140

To see these entries in BibTeX format, use 'print(<citation>,
bibtex=TRUE)', 'toBibtex(.)', or set
'options(citation.bibtex.max=999)'.
```

```r
citation("apeglm")
```

```
To cite package 'apeglm' in publications use:

  Zhu, A., Ibrahim, J.G., Love, M.I. Heavy-tailed prior distributions
  for sequence count data: removing the noise and preserving large
  differences Bioinformatics (2018)

A BibTeX entry for LaTeX users is

  @Article{,
    title = {Heavy-tailed prior distributions for sequence count data: removing the noise an[illegible]
    author = {Anqi Zhu and Joseph G. Ibrahim and Michael I. Love},
    year = {2018},
    journal = {Bioinformatics},
    doi = {10.1093/bioinformatics/bty895},
  }
```

```r
citation("sva")
```

```
To cite package 'sva' in publications use:

  Leek JT, Johnson WE, Parker HS, Fertig EJ, Jaffe AE, Zhang Y, Storey
  JD, Torres LC (2024). _sva: Surrogate Variable Analysis_.
  doi:10.18129/B9.bioc.sva <https://doi.org/10.18129/B9.bioc.sva>, R
  package version 3.52.0, <https://bioconductor.org/packages/sva>.

A BibTeX entry for LaTeX users is

  @Manual{,
    title = {sva: Surrogate Variable Analysis},
    author = {Jeffrey T. Leek and W. Evan Johnson and Hilary S. Parker and Elana J. Fertig a[illegible]
    year = {2024},
    note = {R package version 3.52.0},
    url = {https://bioconductor.org/packages/sva},
    doi = {10.18129/B9.bioc.sva},
  }

ATTENTION: This citation information has been auto-generated from the
package DESCRIPTION file and may need manual editing, see
'help("citation")'.
```

```r
sessionInfo()
```

```
R version 4.4.2 (2024-10-31)
Platform: x86_64-pc-linux-gnu
Running under: Ubuntu 22.04.5 LTS

Matrix products: default
BLAS:   /usr/lib/x86_64-linux-gnu/blas/libblas.so.3.10.0
LAPACK: /usr/lib/x86_64-linux-gnu/lapack/liblapack.so.3.10.0

locale:
 [1] LC_CTYPE=en_AU.UTF-8       LC_NUMERIC=C
 [3] LC_TIME=en_AU.UTF-8        LC_COLLATE=en_AU.UTF-8
 [5] LC_MONETARY=en_AU.UTF-8    LC_MESSAGES=en_AU.UTF-8
 [7] LC_PAPER=en_AU.UTF-8       LC_NAME=C
 [9] LC_ADDRESS=C               LC_TELEPHONE=C
[11] LC_MEASUREMENT=en_AU.UTF-8 LC_IDENTIFICATION=C

time zone: Australia/Melbourne
tzcode source: system (glibc)

attached base packages:
[1] stats4    stats     graphics  grDevices utils     datasets  methods
[8] base

other attached packages:
 [1] sva_3.52.0                  BiocParallel_1.38.0
 [3] genefilter_1.86.0           mgcv_1.9-1
 [5] nlme_3.1-167                ggpubr_0.6.0
 [7] ComplexUpset_1.3.3          UpSetR_1.4.0
 [9] edgeR_4.2.2                 limma_3.60.6
[11] ggh4x_0.3.0                 lubridate_1.9.4
[13] forcats_1.0.0               stringr_1.5.1
[15] dplyr_1.1.4                 purrr_1.0.2
[17] readr_2.1.5                 tidyr_1.3.1
[19] tibble_3.2.1                ggplot2_3.5.1
[21] tidyverse_2.0.0             DESeq2_1.44.0
[23] SummarizedExperiment_1.34.0 Biobase_2.64.0
[25] MatrixGenerics_1.16.0       matrixStats_1.5.0
[27] GenomicRanges_1.56.2        GenomeInfoDb_1.40.1
[29] IRanges_2.38.1              S4Vectors_0.42.1
[31] BiocGenerics_0.50.0         TCGAbiolinks_2.32.0
```

```
[33] mirrorCheck_0.0.1.0

loaded via a namespace (and not attached):
  [1] RColorBrewer_1.1-3          rstudioapi_0.17.1
  [3] jsonlite_1.8.9              magrittr_2.0.3
  [5] farver_2.1.2                rmarkdown_2.29
  [7] zlibbioc_1.50.0             vctrs_0.6.5
  [9] memoise_2.0.1               rstatix_0.7.2
 [11] htmltools_0.5.8.1           S4Arrays_1.4.1
 [13] progress_1.2.3              lambda.r_1.2.4
 [15] curl_6.2.0                  broom_1.0.7
 [17] SparseArray_1.4.8           Formula_1.2-5
 [19] plyr_1.8.9                  httr2_1.1.0
 [21] futile.options_1.0.1        cachem_1.1.0
 [23] lifecycle_1.0.4             pkgconfig_2.0.3
 [25] Matrix_1.7-2                R6_2.6.1
 [27] fastmap_1.2.0               GenomeInfoDbData_1.2.12
 [29] numDeriv_2016.8-1.1         digest_0.6.37
 [31] colorspace_2.1-1            patchwork_1.3.0
 [33] AnnotationDbi_1.66.0        RSQLite_2.3.9
 [35] filelock_1.0.3              labeling_0.4.3
 [37] timechange_0.3.0            httr_1.4.7
 [39] abind_1.4-8                 compiler_4.4.2
 [41] bit64_4.6.0-1               withr_3.0.2
 [43] downloader_0.4              backports_1.5.0
 [45] carData_3.0-5               DBI_1.2.3
 [47] ggsignif_0.6.4              biomaRt_2.60.1
 [49] MASS_7.3-64                 rappdirs_0.3.3
 [51] DelayedArray_0.30.1         tools_4.4.2
 [53] VennDiagram_1.7.3           glue_1.8.0
 [55] grid_4.4.2                  generics_0.1.3
 [57] gtable_0.3.6                tzdb_0.4.0
 [59] data.table_1.16.4           hms_1.1.3
 [61] xml2_1.3.6                  car_3.1-3
 [63] XVector_0.44.0              ggrepel_0.9.6
 [65] pillar_1.10.1               emdbook_1.3.13
 [67] splines_4.4.2               BiocFileCache_2.12.0
 [69] lattice_0.22-5              survival_3.8-3
 [71] bit_4.5.0.1                 annotate_1.82.0
 [73] tidyselect_1.2.1            locfit_1.5-9.11
 [75] Biostrings_2.72.1           knitr_1.49
 [77] gridExtra_2.3               futile.logger_1.4.3
 [79] xfun_0.50                   statmod_1.5.0
```

```
 [81] pheatmap_1.0.12             stringi_1.8.4
 [83] UCSC.utils_1.0.0            yaml_2.3.10
 [85] TCGAbiolinksGUI.data_1.24.0 evaluate_1.0.3
 [87] codetools_0.2-19            bbmle_1.0.25.1
 [89] cli_3.6.4                   xtable_1.8-4
 [91] munsell_0.5.1               Rcpp_1.0.14
 [93] dbplyr_2.5.0                coda_0.19-4.1
 [95] png_0.1-8                   bdsmatrix_1.3-7
 [97] XML_3.99-0.18               parallel_4.4.2
 [99] blob_1.2.4                  prettyunits_1.2.0
[101] mvtnorm_1.3-3               apeglm_1.26.1
[103] scales_1.3.0                crayon_1.5.3
[105] rlang_1.1.5                 formatR_1.14
[107] KEGGREST_1.44.1             rvest_1.0.4
```
