# Supplementary material for: mirrorCheck: an R package facilitating informed use of DESeq2’s lfcShrink() function for differential gene expression analysis of clinical samples
Source: Bioinform Adv. 2025 Apr 2;5(1):vbaf070. doi: 10.1093/bioadv/vbaf070 (PMC12089695; doi:10.1093/bioadv/vbaf070)
Supplement: vbaf070_Supplementary_Data [file vbaf070_supplementary_data.zip › final supps/S6_Supplementary_Cellline_report.pdf]

Cell lines

Kate Scull

Cell line example data

This analysis uses data from Corchette et al. (2020), https://doi.org/10.1038/s41598-020-76881-x. As per the data availability section of Corchette et al, the raw counts files were accessed from the Gene Expression Omnibus under the accession number GSE95077. GSE95077_RAW.tar was downloaded and the txt files were extracted.

To reproduce the Quarto document and other output files, download your own copies of these data files and place the qmd file in the same folder before rendering. You will also need to ensure to following R libraries are installed, including mirrorCheck from the github repository kescull/mirrorCheck: Facilitator functions for getting and assessing DESeq2 lfcShrink results (github.com).

MirrorCheck

Setup:

```
library(mirrorCheck)
library(DESeq2)
library(tidyverse)
library(edgeR)
library(UpSetR)
library(ComplexUpset)
library(ggpubr)
library(ggh4x)
library(sva)

test_match_order <- function(x,y) {
  if (isTRUE(all.equal(x,y))) print('Perfect match in same order')
  if (!isTRUE(all.equal(x,y)) && isTRUE(all.equal(sort(x),sort(y))))
    print('Perfect match in wrong order')
```

```
  if (!isTRUE(all.equal(x,y)) && !isTRUE(all.equal(sort(x),sort(y))))
    print('No match')
}
```

```
fn <- c("GSM2495761_BM_CTRL_141051_INTER-STR_counts.txt",
        "GSM2495762_BM_CTRL_141057_INTER-STR_counts.txt",
        "GSM2495763_BM_CTRL_141063_INTER-STR_counts.txt",
        "GSM2495764_BM_AMIL_141053_INTER-STR_counts.txt",
        "GSM2495765_BM_AMIL_141059_INTER-STR_counts.txt",
        "GSM2495766_BM_AMIL_141065_INTER-STR_counts.txt",
        "GSM2495767_JJ_CTRL_141048_INTER-Str_counts.txt",
        "GSM2495768_JJ_CTRL_141054_INTER-Str_counts.txt",
        "GSM2495769_JJ_CTRL_141060_INTER-Str_counts.txt",
        "GSM2495770_JJ_AMIL_141050_INTER-Str_counts.txt",
        "GSM2495771_JJ_AMIL_141056_INTER-Str_counts.txt",
        "GSM2495772_JJ_AMIL_141062_INTER-Str_counts.txt",
        "GSM3215905_BM_TG_141052_INTER-STR_counts.txt",
        "GSM3215906_BM_TG_141058_INTER-STR_counts.txt",
        "GSM3215907_BM_TG_141064_INTER-STR_counts.txt",
        "GSM3215908_JJ_TG_141049_INTER-STR_counts.txt",
        "GSM3215909_JJ_TG_141055_INTER-STR_counts.txt",
        "GSM3215910_JJ_TG_141061_INTER-STR_counts.txt")
pretty_fn <- sub("_INTER-STR_counts.txt","",fn, ignore.case = T)

mat <- lapply(fn,read.csv, sep="\t",header = F)
mat <- mapply(function(x,y) x %>% rename(!!y := V2),
              mat,pretty_fn, SIMPLIFY = F)
# Need to remove some extra summary data lines after gene counts
mat.all <- mat %>% reduce(full_join,by = "V1") %>%
  filter(substr(V1,1,4) == "ENSG")
rownames(mat.all) <- mat.all$V1
mat.all <- mat.all %>%
  select(-V1) %>%
  as.matrix()
str(mat.all)
```

```
 int [1:57905, 1:18] 0 0 1637 453 719 2 2 61 2354 3025 ...
 - attr(*, "dimnames")=List of 2
  ..$ : chr [1:57905] "ENSG00000000003" "ENSG00000000005" "ENSG00000000419" "ENSG00000000457
  ..$ : chr [1:18] "GSM2495761_BM_CTRL_141051" "GSM2495762_BM_CTRL_141057" "GSM2495763_BM_CT
```

```
metadata <- data.frame(sample=pretty_fn)
metadata <- metadata %>%
  mutate(cell_line = case_when(grepl("_BM_",sample) ~ "CLA",
                               grepl("_JJ_",sample) ~ "CLB"),
         treatment = case_when(grepl("_CTRL_",sample) ~ "T0",
                               grepl("_AMIL_",sample) ~ "T1",
                               grepl("_TG_",sample) ~ "T2"),
         group = paste(cell_line,treatment,sep = "_"))
metadata$group <- as.factor(metadata$group)
print(metadata)
```

```
                     sample cell_line treatment  group
1  GSM2495761_BM_CTRL_141051       CLA        T0 CLA_T0
2  GSM2495762_BM_CTRL_141057       CLA        T0 CLA_T0
3  GSM2495763_BM_CTRL_141063       CLA        T0 CLA_T0
4  GSM2495764_BM_AMIL_141053       CLA        T1 CLA_T1
5  GSM2495765_BM_AMIL_141059       CLA        T1 CLA_T1
6  GSM2495766_BM_AMIL_141065       CLA        T1 CLA_T1
7  GSM2495767_JJ_CTRL_141048       CLB        T0 CLB_T0
8  GSM2495768_JJ_CTRL_141054       CLB        T0 CLB_T0
9  GSM2495769_JJ_CTRL_141060       CLB        T0 CLB_T0
10 GSM2495770_JJ_AMIL_141050       CLB        T1 CLB_T1
11 GSM2495771_JJ_AMIL_141056       CLB        T1 CLB_T1
12 GSM2495772_JJ_AMIL_141062       CLB        T1 CLB_T1
13   GSM3215905_BM_TG_141052       CLA        T2 CLA_T2
14   GSM3215906_BM_TG_141058       CLA        T2 CLA_T2
15   GSM3215907_BM_TG_141064       CLA        T2 CLA_T2
16   GSM3215908_JJ_TG_141049       CLB        T2 CLB_T2
17   GSM3215909_JJ_TG_141055       CLB        T2 CLB_T2
18   GSM3215910_JJ_TG_141061       CLB        T2 CLB_T2
```

```
test_match_order(metadata$sample,colnames(mat.all))
```

```
[1] "Perfect match in same order"
```

```
dds <- DESeqDataSetFromMatrix(countData = mat.all,
                              colData = metadata,
                              design = ~group)
```

Principal component analysis

```r
vsd <- vst(dds)
pdata <- DESeq2::plotPCA(vsd, ntop = length(vsd), intgroup= "group",
                         returnData=T)
```

```
using ntop=57905 top features by variance
```

```r
percentVar <- attr(pdata,"percentVar")
colour_blind_friendly <- c('#EE7733', '#0077BB','#BBBBBB','#EE3377',
                           '#33BBEE', '#CC3311', '#009988' )
joiner <- metadata %>% select(name = sample, cell_line,treatment)
pdata <- pdata %>% left_join(joiner, by = "name")

p <- ggplot(pdata, aes(x=PC1,y=PC2,color=treatment,shape = cell_line)) +
  geom_point(size = 4, alpha = 0.6) +
  ggtitle("Cell line") +
  labs(x = paste0("PC1: ",round(percentVar[1]*100),"% variance"),
       y = paste0("PC2: ",round(percentVar[2]*100),"% variance"),
       shape = "Cell line",
       color = "Treatment") +
  theme_classic(base_size = 16) +
  theme(plot.title = element_text(face = "bold")) +
  force_panelsizes(rows = unit(2.5,"in"),
                   cols = unit(2.5,"in")) +
  scale_colour_manual(values = colour_blind_friendly)
p
```

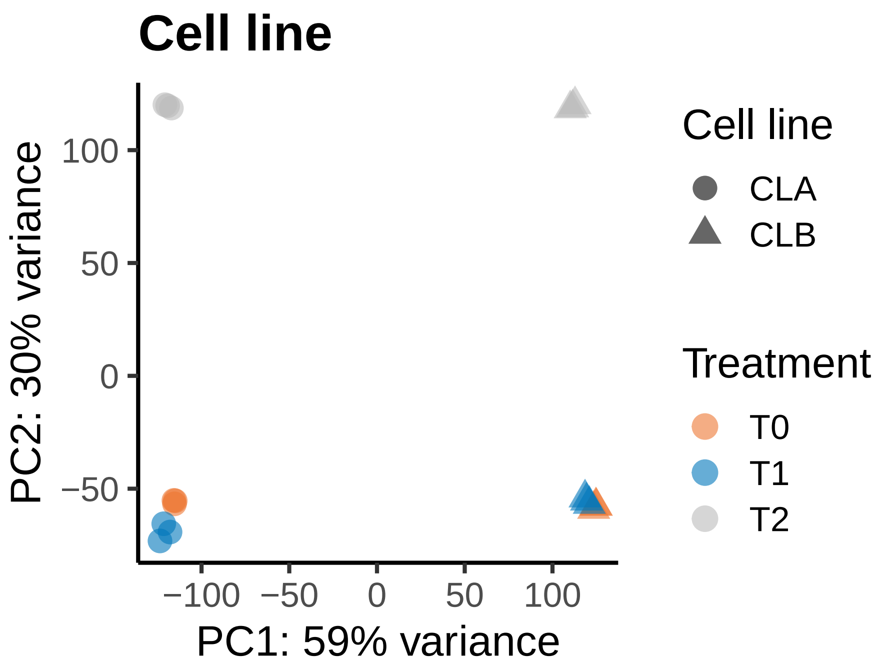

Run DESeq2

Run DESeq2 using lfcShrink, facilitated by mirrorCheck, *without any further clean-up steps.* This creates output csv tables and pdf reports in a folder alongside this qmd file.

```
folder <- "DESeq_noclean"
dir.create(folder)
```

```
Warning in dir.create(folder): 'DESeq_noclean' already exists
```

```
dds
```

```
class: DESeqDataSet
dim: 57905 18
metadata(1): version
assays(1): counts
rownames(57905): ENSG00000000003 ENSG00000000005 ... ENSG00000273492
  ENSG00000273493
rowData names(0):
colnames(18): GSM2495761_BM_CTRL_141051 GSM2495762_BM_CTRL_141057 ...
  GSM3215909_JJ_TG_141055 GSM3215910_JJ_TG_141061
colData names(4): sample cell_line treatment group
```

```r
run_DESeq_all_contrasts(dds,folder,
                        condition = "group",
                        p.cutoff = 0.01,
                        top.n = 0,
                        print.all = T,
                        useDingbats = T)
```

```
estimating size factors

estimating dispersions

gene-wise dispersion estimates

mean-dispersion relationship

final dispersion estimates

fitting model and testing

using 'apeglm' for LFC shrinkage. If used in published research, please cite:
    Zhu, A., Ibrahim, J.G., Love, M.I. (2018) Heavy-tailed prior distributions for
    sequence count data: removing the noise and preserving large differences.
    Bioinformatics. https://doi.org/10.1093/bioinformatics/bty895
using 'apeglm' for LFC shrinkage. If used in published research, please cite:
    Zhu, A., Ibrahim, J.G., Love, M.I. (2018) Heavy-tailed prior distributions for
    sequence count data: removing the noise and preserving large differences.
    Bioinformatics. https://doi.org/10.1093/bioinformatics/bty895
using 'apeglm' for LFC shrinkage. If used in published research, please cite:
    Zhu, A., Ibrahim, J.G., Love, M.I. (2018) Heavy-tailed prior distributions for
    sequence count data: removing the noise and preserving large differences.
    Bioinformatics. https://doi.org/10.1093/bioinformatics/bty895
using 'apeglm' for LFC shrinkage. If used in published research, please cite:
    Zhu, A., Ibrahim, J.G., Love, M.I. (2018) Heavy-tailed prior distributions for
    sequence count data: removing the noise and preserving large differences.
    Bioinformatics. https://doi.org/10.1093/bioinformatics/bty895
using 'apeglm' for LFC shrinkage. If used in published research, please cite:
    Zhu, A., Ibrahim, J.G., Love, M.I. (2018) Heavy-tailed prior distributions for
    sequence count data: removing the noise and preserving large differences.
    Bioinformatics. https://doi.org/10.1093/bioinformatics/bty895
```

```
found results columns, replacing these

using 'apeglm' for LFC shrinkage. If used in published research, please cite:
    Zhu, A., Ibrahim, J.G., Love, M.I. (2018) Heavy-tailed prior distributions for
    sequence count data: removing the noise and preserving large differences.
    Bioinformatics. https://doi.org/10.1093/bioinformatics/bty895
using 'apeglm' for LFC shrinkage. If used in published research, please cite:
    Zhu, A., Ibrahim, J.G., Love, M.I. (2018) Heavy-tailed prior distributions for
    sequence count data: removing the noise and preserving large differences.
    Bioinformatics. https://doi.org/10.1093/bioinformatics/bty895
using 'apeglm' for LFC shrinkage. If used in published research, please cite:
    Zhu, A., Ibrahim, J.G., Love, M.I. (2018) Heavy-tailed prior distributions for
    sequence count data: removing the noise and preserving large differences.
    Bioinformatics. https://doi.org/10.1093/bioinformatics/bty895
using 'apeglm' for LFC shrinkage. If used in published research, please cite:
    Zhu, A., Ibrahim, J.G., Love, M.I. (2018) Heavy-tailed prior distributions for
    sequence count data: removing the noise and preserving large differences.
    Bioinformatics. https://doi.org/10.1093/bioinformatics/bty895
using 'apeglm' for LFC shrinkage. If used in published research, please cite:
    Zhu, A., Ibrahim, J.G., Love, M.I. (2018) Heavy-tailed prior distributions for
    sequence count data: removing the noise and preserving large differences.
    Bioinformatics. https://doi.org/10.1093/bioinformatics/bty895

found results columns, replacing these

using 'apeglm' for LFC shrinkage. If used in published research, please cite:
    Zhu, A., Ibrahim, J.G., Love, M.I. (2018) Heavy-tailed prior distributions for
    sequence count data: removing the noise and preserving large differences.
    Bioinformatics. https://doi.org/10.1093/bioinformatics/bty895
using 'apeglm' for LFC shrinkage. If used in published research, please cite:
    Zhu, A., Ibrahim, J.G., Love, M.I. (2018) Heavy-tailed prior distributions for
    sequence count data: removing the noise and preserving large differences.
    Bioinformatics. https://doi.org/10.1093/bioinformatics/bty895
using 'apeglm' for LFC shrinkage. If used in published research, please cite:
    Zhu, A., Ibrahim, J.G., Love, M.I. (2018) Heavy-tailed prior distributions for
    sequence count data: removing the noise and preserving large differences.
    Bioinformatics. https://doi.org/10.1093/bioinformatics/bty895
using 'apeglm' for LFC shrinkage. If used in published research, please cite:
    Zhu, A., Ibrahim, J.G., Love, M.I. (2018) Heavy-tailed prior distributions for
    sequence count data: removing the noise and preserving large differences.
    Bioinformatics. https://doi.org/10.1093/bioinformatics/bty895
using 'apeglm' for LFC shrinkage. If used in published research, please cite:
```

```
    Zhu, A., Ibrahim, J.G., Love, M.I. (2018) Heavy-tailed prior distributions for
    sequence count data: removing the noise and preserving large differences.
    Bioinformatics. https://doi.org/10.1093/bioinformatics/bty895

found results columns, replacing these

using 'apeglm' for LFC shrinkage. If used in published research, please cite:
    Zhu, A., Ibrahim, J.G., Love, M.I. (2018) Heavy-tailed prior distributions for
    sequence count data: removing the noise and preserving large differences.
    Bioinformatics. https://doi.org/10.1093/bioinformatics/bty895
using 'apeglm' for LFC shrinkage. If used in published research, please cite:
    Zhu, A., Ibrahim, J.G., Love, M.I. (2018) Heavy-tailed prior distributions for
    sequence count data: removing the noise and preserving large differences.
    Bioinformatics. https://doi.org/10.1093/bioinformatics/bty895
using 'apeglm' for LFC shrinkage. If used in published research, please cite:
    Zhu, A., Ibrahim, J.G., Love, M.I. (2018) Heavy-tailed prior distributions for
    sequence count data: removing the noise and preserving large differences.
    Bioinformatics. https://doi.org/10.1093/bioinformatics/bty895
using 'apeglm' for LFC shrinkage. If used in published research, please cite:
    Zhu, A., Ibrahim, J.G., Love, M.I. (2018) Heavy-tailed prior distributions for
    sequence count data: removing the noise and preserving large differences.
    Bioinformatics. https://doi.org/10.1093/bioinformatics/bty895
using 'apeglm' for LFC shrinkage. If used in published research, please cite:
    Zhu, A., Ibrahim, J.G., Love, M.I. (2018) Heavy-tailed prior distributions for
    sequence count data: removing the noise and preserving large differences.
    Bioinformatics. https://doi.org/10.1093/bioinformatics/bty895

found results columns, replacing these

using 'apeglm' for LFC shrinkage. If used in published research, please cite:
    Zhu, A., Ibrahim, J.G., Love, M.I. (2018) Heavy-tailed prior distributions for
    sequence count data: removing the noise and preserving large differences.
    Bioinformatics. https://doi.org/10.1093/bioinformatics/bty895
using 'apeglm' for LFC shrinkage. If used in published research, please cite:
    Zhu, A., Ibrahim, J.G., Love, M.I. (2018) Heavy-tailed prior distributions for
    sequence count data: removing the noise and preserving large differences.
    Bioinformatics. https://doi.org/10.1093/bioinformatics/bty895
using 'apeglm' for LFC shrinkage. If used in published research, please cite:
    Zhu, A., Ibrahim, J.G., Love, M.I. (2018) Heavy-tailed prior distributions for
    sequence count data: removing the noise and preserving large differences.
    Bioinformatics. https://doi.org/10.1093/bioinformatics/bty895
```

```
using 'apeglm' for LFC shrinkage. If used in published research, please cite:
    Zhu, A., Ibrahim, J.G., Love, M.I. (2018) Heavy-tailed prior distributions for
    sequence count data: removing the noise and preserving large differences.
    Bioinformatics. https://doi.org/10.1093/bioinformatics/bty895
using 'apeglm' for LFC shrinkage. If used in published research, please cite:
    Zhu, A., Ibrahim, J.G., Love, M.I. (2018) Heavy-tailed prior distributions for
    sequence count data: removing the noise and preserving large differences.
    Bioinformatics. https://doi.org/10.1093/bioinformatics/bty895
```

```
found results columns, replacing these
```

```
using 'apeglm' for LFC shrinkage. If used in published research, please cite:
    Zhu, A., Ibrahim, J.G., Love, M.I. (2018) Heavy-tailed prior distributions for
    sequence count data: removing the noise and preserving large differences.
    Bioinformatics. https://doi.org/10.1093/bioinformatics/bty895
using 'apeglm' for LFC shrinkage. If used in published research, please cite:
    Zhu, A., Ibrahim, J.G., Love, M.I. (2018) Heavy-tailed prior distributions for
    sequence count data: removing the noise and preserving large differences.
    Bioinformatics. https://doi.org/10.1093/bioinformatics/bty895
using 'apeglm' for LFC shrinkage. If used in published research, please cite:
    Zhu, A., Ibrahim, J.G., Love, M.I. (2018) Heavy-tailed prior distributions for
    sequence count data: removing the noise and preserving large differences.
    Bioinformatics. https://doi.org/10.1093/bioinformatics/bty895
using 'apeglm' for LFC shrinkage. If used in published research, please cite:
    Zhu, A., Ibrahim, J.G., Love, M.I. (2018) Heavy-tailed prior distributions for
    sequence count data: removing the noise and preserving large differences.
    Bioinformatics. https://doi.org/10.1093/bioinformatics/bty895
using 'apeglm' for LFC shrinkage. If used in published research, please cite:
    Zhu, A., Ibrahim, J.G., Love, M.I. (2018) Heavy-tailed prior distributions for
    sequence count data: removing the noise and preserving large differences.
    Bioinformatics. https://doi.org/10.1093/bioinformatics/bty895
```

```r
group <- levels(as.factor(dds$group))
set <- compare_reciprocal_contrasts(group,folder)
```

```
Warning: Removed 2585 rows containing non-finite outside the scale range
(`stat_density()`).
```

```r
saveRDS(set,"cell_noclean.rds")
```

Run DESeq2 using lfcShrink, facilitated by mirrorCheck, *after prefiltering.* This creates output csv tables and pdf reports in a folder alongside this qmd file.

```
folder <- "DESeq_prefilt"
dir.create(folder)
```

```
Warning in dir.create(folder): 'DESeq_prefilt' already exists
```

```
expr.filter <- edgeR::filterByExpr(dds, group = dds$group)
dds.filtered <- dds[expr.filter, ]
dds.filtered
```

```
class: DESeqDataSet
dim: 17772 18
metadata(1): version
assays(1): counts
rownames(17772): ENSG00000000419 ENSG00000000457 ... ENSG00000273486
  ENSG00000273488
rowData names(0):
colnames(18): GSM2495761_BM_CTRL_141051 GSM2495762_BM_CTRL_141057 ...
  GSM3215909_JJ_TG_141055 GSM3215910_JJ_TG_141061
colData names(4): sample cell_line treatment group
```

```
run_DESeq_all_contrasts(dds.filtered,folder,
                        condition = "group",
                        p.cutoff = 0.01,
                        top.n = 0,
                        print.all = T,
                        useDingbats = T)
```

```
estimating size factors

estimating dispersions

gene-wise dispersion estimates

mean-dispersion relationship

final dispersion estimates
```

```
fitting model and testing

using 'apeglm' for LFC shrinkage. If used in published research, please cite:
    Zhu, A., Ibrahim, J.G., Love, M.I. (2018) Heavy-tailed prior distributions for
    sequence count data: removing the noise and preserving large differences.
    Bioinformatics. https://doi.org/10.1093/bioinformatics/bty895
using 'apeglm' for LFC shrinkage. If used in published research, please cite:
    Zhu, A., Ibrahim, J.G., Love, M.I. (2018) Heavy-tailed prior distributions for
    sequence count data: removing the noise and preserving large differences.
    Bioinformatics. https://doi.org/10.1093/bioinformatics/bty895
using 'apeglm' for LFC shrinkage. If used in published research, please cite:
    Zhu, A., Ibrahim, J.G., Love, M.I. (2018) Heavy-tailed prior distributions for
    sequence count data: removing the noise and preserving large differences.
    Bioinformatics. https://doi.org/10.1093/bioinformatics/bty895
using 'apeglm' for LFC shrinkage. If used in published research, please cite:
    Zhu, A., Ibrahim, J.G., Love, M.I. (2018) Heavy-tailed prior distributions for
    sequence count data: removing the noise and preserving large differences.
    Bioinformatics. https://doi.org/10.1093/bioinformatics/bty895
using 'apeglm' for LFC shrinkage. If used in published research, please cite:
    Zhu, A., Ibrahim, J.G., Love, M.I. (2018) Heavy-tailed prior distributions for
    sequence count data: removing the noise and preserving large differences.
    Bioinformatics. https://doi.org/10.1093/bioinformatics/bty895

found results columns, replacing these

using 'apeglm' for LFC shrinkage. If used in published research, please cite:
    Zhu, A., Ibrahim, J.G., Love, M.I. (2018) Heavy-tailed prior distributions for
    sequence count data: removing the noise and preserving large differences.
    Bioinformatics. https://doi.org/10.1093/bioinformatics/bty895
using 'apeglm' for LFC shrinkage. If used in published research, please cite:
    Zhu, A., Ibrahim, J.G., Love, M.I. (2018) Heavy-tailed prior distributions for
    sequence count data: removing the noise and preserving large differences.
    Bioinformatics. https://doi.org/10.1093/bioinformatics/bty895
using 'apeglm' for LFC shrinkage. If used in published research, please cite:
    Zhu, A., Ibrahim, J.G., Love, M.I. (2018) Heavy-tailed prior distributions for
    sequence count data: removing the noise and preserving large differences.
    Bioinformatics. https://doi.org/10.1093/bioinformatics/bty895
using 'apeglm' for LFC shrinkage. If used in published research, please cite:
    Zhu, A., Ibrahim, J.G., Love, M.I. (2018) Heavy-tailed prior distributions for
    sequence count data: removing the noise and preserving large differences.
    Bioinformatics. https://doi.org/10.1093/bioinformatics/bty895
using 'apeglm' for LFC shrinkage. If used in published research, please cite:
```

```
    Zhu, A., Ibrahim, J.G., Love, M.I. (2018) Heavy-tailed prior distributions for
    sequence count data: removing the noise and preserving large differences.
    Bioinformatics. https://doi.org/10.1093/bioinformatics/bty895
```

```
found results columns, replacing these
```

```
using 'apeglm' for LFC shrinkage. If used in published research, please cite:
    Zhu, A., Ibrahim, J.G., Love, M.I. (2018) Heavy-tailed prior distributions for
    sequence count data: removing the noise and preserving large differences.
    Bioinformatics. https://doi.org/10.1093/bioinformatics/bty895
using 'apeglm' for LFC shrinkage. If used in published research, please cite:
    Zhu, A., Ibrahim, J.G., Love, M.I. (2018) Heavy-tailed prior distributions for
    sequence count data: removing the noise and preserving large differences.
    Bioinformatics. https://doi.org/10.1093/bioinformatics/bty895
using 'apeglm' for LFC shrinkage. If used in published research, please cite:
    Zhu, A., Ibrahim, J.G., Love, M.I. (2018) Heavy-tailed prior distributions for
    sequence count data: removing the noise and preserving large differences.
    Bioinformatics. https://doi.org/10.1093/bioinformatics/bty895
using 'apeglm' for LFC shrinkage. If used in published research, please cite:
    Zhu, A., Ibrahim, J.G., Love, M.I. (2018) Heavy-tailed prior distributions for
    sequence count data: removing the noise and preserving large differences.
    Bioinformatics. https://doi.org/10.1093/bioinformatics/bty895
using 'apeglm' for LFC shrinkage. If used in published research, please cite:
    Zhu, A., Ibrahim, J.G., Love, M.I. (2018) Heavy-tailed prior distributions for
    sequence count data: removing the noise and preserving large differences.
    Bioinformatics. https://doi.org/10.1093/bioinformatics/bty895
```

```
found results columns, replacing these
```

```
using 'apeglm' for LFC shrinkage. If used in published research, please cite:
    Zhu, A., Ibrahim, J.G., Love, M.I. (2018) Heavy-tailed prior distributions for
    sequence count data: removing the noise and preserving large differences.
    Bioinformatics. https://doi.org/10.1093/bioinformatics/bty895
using 'apeglm' for LFC shrinkage. If used in published research, please cite:
    Zhu, A., Ibrahim, J.G., Love, M.I. (2018) Heavy-tailed prior distributions for
    sequence count data: removing the noise and preserving large differences.
    Bioinformatics. https://doi.org/10.1093/bioinformatics/bty895
using 'apeglm' for LFC shrinkage. If used in published research, please cite:
    Zhu, A., Ibrahim, J.G., Love, M.I. (2018) Heavy-tailed prior distributions for
    sequence count data: removing the noise and preserving large differences.
    Bioinformatics. https://doi.org/10.1093/bioinformatics/bty895
```

```
using 'apeglm' for LFC shrinkage. If used in published research, please cite:
    Zhu, A., Ibrahim, J.G., Love, M.I. (2018) Heavy-tailed prior distributions for
    sequence count data: removing the noise and preserving large differences.
    Bioinformatics. https://doi.org/10.1093/bioinformatics/bty895
using 'apeglm' for LFC shrinkage. If used in published research, please cite:
    Zhu, A., Ibrahim, J.G., Love, M.I. (2018) Heavy-tailed prior distributions for
    sequence count data: removing the noise and preserving large differences.
    Bioinformatics. https://doi.org/10.1093/bioinformatics/bty895

found results columns, replacing these

using 'apeglm' for LFC shrinkage. If used in published research, please cite:
    Zhu, A., Ibrahim, J.G., Love, M.I. (2018) Heavy-tailed prior distributions for
    sequence count data: removing the noise and preserving large differences.
    Bioinformatics. https://doi.org/10.1093/bioinformatics/bty895
using 'apeglm' for LFC shrinkage. If used in published research, please cite:
    Zhu, A., Ibrahim, J.G., Love, M.I. (2018) Heavy-tailed prior distributions for
    sequence count data: removing the noise and preserving large differences.
    Bioinformatics. https://doi.org/10.1093/bioinformatics/bty895
using 'apeglm' for LFC shrinkage. If used in published research, please cite:
    Zhu, A., Ibrahim, J.G., Love, M.I. (2018) Heavy-tailed prior distributions for
    sequence count data: removing the noise and preserving large differences.
    Bioinformatics. https://doi.org/10.1093/bioinformatics/bty895
using 'apeglm' for LFC shrinkage. If used in published research, please cite:
    Zhu, A., Ibrahim, J.G., Love, M.I. (2018) Heavy-tailed prior distributions for
    sequence count data: removing the noise and preserving large differences.
    Bioinformatics. https://doi.org/10.1093/bioinformatics/bty895
using 'apeglm' for LFC shrinkage. If used in published research, please cite:
    Zhu, A., Ibrahim, J.G., Love, M.I. (2018) Heavy-tailed prior distributions for
    sequence count data: removing the noise and preserving large differences.
    Bioinformatics. https://doi.org/10.1093/bioinformatics/bty895

found results columns, replacing these

using 'apeglm' for LFC shrinkage. If used in published research, please cite:
    Zhu, A., Ibrahim, J.G., Love, M.I. (2018) Heavy-tailed prior distributions for
    sequence count data: removing the noise and preserving large differences.
    Bioinformatics. https://doi.org/10.1093/bioinformatics/bty895
using 'apeglm' for LFC shrinkage. If used in published research, please cite:
    Zhu, A., Ibrahim, J.G., Love, M.I. (2018) Heavy-tailed prior distributions for
    sequence count data: removing the noise and preserving large differences.
```

```
    Bioinformatics. https://doi.org/10.1093/bioinformatics/bty895
using 'apeglm' for LFC shrinkage. If used in published research, please cite:
    Zhu, A., Ibrahim, J.G., Love, M.I. (2018) Heavy-tailed prior distributions for
    sequence count data: removing the noise and preserving large differences.
    Bioinformatics. https://doi.org/10.1093/bioinformatics/bty895
using 'apeglm' for LFC shrinkage. If used in published research, please cite:
    Zhu, A., Ibrahim, J.G., Love, M.I. (2018) Heavy-tailed prior distributions for
    sequence count data: removing the noise and preserving large differences.
    Bioinformatics. https://doi.org/10.1093/bioinformatics/bty895
using 'apeglm' for LFC shrinkage. If used in published research, please cite:
    Zhu, A., Ibrahim, J.G., Love, M.I. (2018) Heavy-tailed prior distributions for
    sequence count data: removing the noise and preserving large differences.
    Bioinformatics. https://doi.org/10.1093/bioinformatics/bty895
```

```
group <- levels(as.factor(dds.filtered$group))
set <- compare_reciprocal_contrasts(group,folder)
```

```
Warning: Removed 2615 rows containing non-finite outside the scale range
(`stat_density()`).
```

```
saveRDS(set,"cell_prefilt.rds")
```

Finally, we try *surrogate variable analysis* (SVA) to remove unwanted/hidden sources of variation. The presence of unknown sources of variation can interfere with DGEA and increase discordance in reciprocal contrasts, so we use SVA to see if we can identify these as per the method in Love et al. (2016), before running DESeq with mirrorCheck. This creates output csv tables and pdf reports in a folder alongside this qmd file.

```
folder <- "DESeq_sva"
dir.create(folder)
```

```
Warning in dir.create(folder): 'DESeq_sva' already exists
```

```
dds <- DESeqDataSetFromMatrix(countData = mat.all,
                              colData = metadata,
                              design = ~group)
dds <- DESeq(dds)
```

```
estimating size factors
```

```
estimating dispersions

gene-wise dispersion estimates

mean-dispersion relationship

final dispersion estimates

fitting model and testing
```

```
dat <- counts(dds, normalized=TRUE)
idx <- rowMeans(dat) > 1
dat <- dat[idx,]
mod <- model.matrix(~ group, colData(dds))
mod0 <- model.matrix(~ 1, colData(dds))

#find probable number of unwanted variables
num_sv <- num.sv(dat,mod,method = "be")
print(num_sv)
```

```
[1] 1
```

```
#num_sv = 4
svseq <- svaseq(dat, mod, mod0, n.sv=4)
```

```
Number of significant surrogate variables is:  4
Iteration (out of 5 ):1  2  3  4  5
```

```
dds.sva <- dds
dds.sva$SV1 <- svseq$sv[,1]
dds.sva$SV2 <- svseq$sv[,2]
dds.sva$SV3 <- svseq$sv[,3]
dds.sva$SV4 <- svseq$sv[,4]
design(dds.sva) <- ~ SV1 + SV2 + SV3 + SV4 + group

run_DESeq_all_contrasts(dds.sva,folder,
                        condition = "group",
                        p.cutoff = 0.01,
                        top.n = 0,
                        print.all = T,
                        useDingbats = T)
```

```
using pre-existing size factors

estimating dispersions

found already estimated dispersions, replacing these

gene-wise dispersion estimates

mean-dispersion relationship

final dispersion estimates

fitting model and testing

1 rows did not converge in beta, labelled in mcols(object)$betaConv. Use larger maxit argume

using 'apeglm' for LFC shrinkage. If used in published research, please cite:
    Zhu, A., Ibrahim, J.G., Love, M.I. (2018) Heavy-tailed prior distributions for
    sequence count data: removing the noise and preserving large differences.
    Bioinformatics. https://doi.org/10.1093/bioinformatics/bty895
using 'apeglm' for LFC shrinkage. If used in published research, please cite:
    Zhu, A., Ibrahim, J.G., Love, M.I. (2018) Heavy-tailed prior distributions for
    sequence count data: removing the noise and preserving large differences.
    Bioinformatics. https://doi.org/10.1093/bioinformatics/bty895
using 'apeglm' for LFC shrinkage. If used in published research, please cite:
    Zhu, A., Ibrahim, J.G., Love, M.I. (2018) Heavy-tailed prior distributions for
    sequence count data: removing the noise and preserving large differences.
    Bioinformatics. https://doi.org/10.1093/bioinformatics/bty895
using 'apeglm' for LFC shrinkage. If used in published research, please cite:
    Zhu, A., Ibrahim, J.G., Love, M.I. (2018) Heavy-tailed prior distributions for
    sequence count data: removing the noise and preserving large differences.
    Bioinformatics. https://doi.org/10.1093/bioinformatics/bty895
using 'apeglm' for LFC shrinkage. If used in published research, please cite:
    Zhu, A., Ibrahim, J.G., Love, M.I. (2018) Heavy-tailed prior distributions for
    sequence count data: removing the noise and preserving large differences.
    Bioinformatics. https://doi.org/10.1093/bioinformatics/bty895

found results columns, replacing these
```

```
using 'apeglm' for LFC shrinkage. If used in published research, please cite:
    Zhu, A., Ibrahim, J.G., Love, M.I. (2018) Heavy-tailed prior distributions for
    sequence count data: removing the noise and preserving large differences.
    Bioinformatics. https://doi.org/10.1093/bioinformatics/bty895
using 'apeglm' for LFC shrinkage. If used in published research, please cite:
    Zhu, A., Ibrahim, J.G., Love, M.I. (2018) Heavy-tailed prior distributions for
    sequence count data: removing the noise and preserving large differences.
    Bioinformatics. https://doi.org/10.1093/bioinformatics/bty895
using 'apeglm' for LFC shrinkage. If used in published research, please cite:
    Zhu, A., Ibrahim, J.G., Love, M.I. (2018) Heavy-tailed prior distributions for
    sequence count data: removing the noise and preserving large differences.
    Bioinformatics. https://doi.org/10.1093/bioinformatics/bty895
using 'apeglm' for LFC shrinkage. If used in published research, please cite:
    Zhu, A., Ibrahim, J.G., Love, M.I. (2018) Heavy-tailed prior distributions for
    sequence count data: removing the noise and preserving large differences.
    Bioinformatics. https://doi.org/10.1093/bioinformatics/bty895
using 'apeglm' for LFC shrinkage. If used in published research, please cite:
    Zhu, A., Ibrahim, J.G., Love, M.I. (2018) Heavy-tailed prior distributions for
    sequence count data: removing the noise and preserving large differences.
    Bioinformatics. https://doi.org/10.1093/bioinformatics/bty895

found results columns, replacing these

using 'apeglm' for LFC shrinkage. If used in published research, please cite:
    Zhu, A., Ibrahim, J.G., Love, M.I. (2018) Heavy-tailed prior distributions for
    sequence count data: removing the noise and preserving large differences.
    Bioinformatics. https://doi.org/10.1093/bioinformatics/bty895
using 'apeglm' for LFC shrinkage. If used in published research, please cite:
    Zhu, A., Ibrahim, J.G., Love, M.I. (2018) Heavy-tailed prior distributions for
    sequence count data: removing the noise and preserving large differences.
    Bioinformatics. https://doi.org/10.1093/bioinformatics/bty895
using 'apeglm' for LFC shrinkage. If used in published research, please cite:
    Zhu, A., Ibrahim, J.G., Love, M.I. (2018) Heavy-tailed prior distributions for
    sequence count data: removing the noise and preserving large differences.
    Bioinformatics. https://doi.org/10.1093/bioinformatics/bty895
using 'apeglm' for LFC shrinkage. If used in published research, please cite:
    Zhu, A., Ibrahim, J.G., Love, M.I. (2018) Heavy-tailed prior distributions for
    sequence count data: removing the noise and preserving large differences.
    Bioinformatics. https://doi.org/10.1093/bioinformatics/bty895
using 'apeglm' for LFC shrinkage. If used in published research, please cite:
    Zhu, A., Ibrahim, J.G., Love, M.I. (2018) Heavy-tailed prior distributions for
    sequence count data: removing the noise and preserving large differences.
```

```
    Bioinformatics. https://doi.org/10.1093/bioinformatics/bty895

found results columns, replacing these

1 rows did not converge in beta, labelled in mcols(object)$betaConv. Use larger maxit argumen

using 'apeglm' for LFC shrinkage. If used in published research, please cite:
    Zhu, A., Ibrahim, J.G., Love, M.I. (2018) Heavy-tailed prior distributions for
    sequence count data: removing the noise and preserving large differences.
    Bioinformatics. https://doi.org/10.1093/bioinformatics/bty895
using 'apeglm' for LFC shrinkage. If used in published research, please cite:
    Zhu, A., Ibrahim, J.G., Love, M.I. (2018) Heavy-tailed prior distributions for
    sequence count data: removing the noise and preserving large differences.
    Bioinformatics. https://doi.org/10.1093/bioinformatics/bty895
using 'apeglm' for LFC shrinkage. If used in published research, please cite:
    Zhu, A., Ibrahim, J.G., Love, M.I. (2018) Heavy-tailed prior distributions for
    sequence count data: removing the noise and preserving large differences.
    Bioinformatics. https://doi.org/10.1093/bioinformatics/bty895
using 'apeglm' for LFC shrinkage. If used in published research, please cite:
    Zhu, A., Ibrahim, J.G., Love, M.I. (2018) Heavy-tailed prior distributions for
    sequence count data: removing the noise and preserving large differences.
    Bioinformatics. https://doi.org/10.1093/bioinformatics/bty895
using 'apeglm' for LFC shrinkage. If used in published research, please cite:
    Zhu, A., Ibrahim, J.G., Love, M.I. (2018) Heavy-tailed prior distributions for
    sequence count data: removing the noise and preserving large differences.
    Bioinformatics. https://doi.org/10.1093/bioinformatics/bty895

found results columns, replacing these

using 'apeglm' for LFC shrinkage. If used in published research, please cite:
    Zhu, A., Ibrahim, J.G., Love, M.I. (2018) Heavy-tailed prior distributions for
    sequence count data: removing the noise and preserving large differences.
    Bioinformatics. https://doi.org/10.1093/bioinformatics/bty895
using 'apeglm' for LFC shrinkage. If used in published research, please cite:
    Zhu, A., Ibrahim, J.G., Love, M.I. (2018) Heavy-tailed prior distributions for
    sequence count data: removing the noise and preserving large differences.
    Bioinformatics. https://doi.org/10.1093/bioinformatics/bty895
using 'apeglm' for LFC shrinkage. If used in published research, please cite:
    Zhu, A., Ibrahim, J.G., Love, M.I. (2018) Heavy-tailed prior distributions for
    sequence count data: removing the noise and preserving large differences.
    Bioinformatics. https://doi.org/10.1093/bioinformatics/bty895
```

```
using 'apeglm' for LFC shrinkage. If used in published research, please cite:
    Zhu, A., Ibrahim, J.G., Love, M.I. (2018) Heavy-tailed prior distributions for
    sequence count data: removing the noise and preserving large differences.
    Bioinformatics. https://doi.org/10.1093/bioinformatics/bty895
using 'apeglm' for LFC shrinkage. If used in published research, please cite:
    Zhu, A., Ibrahim, J.G., Love, M.I. (2018) Heavy-tailed prior distributions for
    sequence count data: removing the noise and preserving large differences.
    Bioinformatics. https://doi.org/10.1093/bioinformatics/bty895
```

```
found results columns, replacing these
```

```
1 rows did not converge in beta, labelled in mcols(object)$betaConv. Use larger maxit argumen
```

```
using 'apeglm' for LFC shrinkage. If used in published research, please cite:
    Zhu, A., Ibrahim, J.G., Love, M.I. (2018) Heavy-tailed prior distributions for
    sequence count data: removing the noise and preserving large differences.
    Bioinformatics. https://doi.org/10.1093/bioinformatics/bty895
using 'apeglm' for LFC shrinkage. If used in published research, please cite:
    Zhu, A., Ibrahim, J.G., Love, M.I. (2018) Heavy-tailed prior distributions for
    sequence count data: removing the noise and preserving large differences.
    Bioinformatics. https://doi.org/10.1093/bioinformatics/bty895
using 'apeglm' for LFC shrinkage. If used in published research, please cite:
    Zhu, A., Ibrahim, J.G., Love, M.I. (2018) Heavy-tailed prior distributions for
    sequence count data: removing the noise and preserving large differences.
    Bioinformatics. https://doi.org/10.1093/bioinformatics/bty895
using 'apeglm' for LFC shrinkage. If used in published research, please cite:
    Zhu, A., Ibrahim, J.G., Love, M.I. (2018) Heavy-tailed prior distributions for
    sequence count data: removing the noise and preserving large differences.
    Bioinformatics. https://doi.org/10.1093/bioinformatics/bty895
using 'apeglm' for LFC shrinkage. If used in published research, please cite:
    Zhu, A., Ibrahim, J.G., Love, M.I. (2018) Heavy-tailed prior distributions for
    sequence count data: removing the noise and preserving large differences.
    Bioinformatics. https://doi.org/10.1093/bioinformatics/bty895
```

```r
group <- levels(as.factor(dds.sva$group))
set_sva <- compare_reciprocal_contrasts(group,folder)
```

```
Warning: Removed 2775 rows containing non-finite outside the scale range
(`stat_density()`).
```

```
saveRDS(set_sva,"cell_sva.rds")
```

Question: How does this compare to edgeR? Are the discordant DEGs found to be DEGs with edgeR? Are the concordant DEGs also found with edgeR?

edgeR is another well-regarded tool for differential expression analysis. Filtering the data to eliminate low count genes is an important part of the standard edgeR workflow, and edgeR includes a specialised function to help make the filtering more data-dependent and sophisticated (which is why we borrowed it to test prefiltering of the dds, above). While edgeR does adjust the raw log fold change values using a "prior count" strategy, this is not intended to replace filtration. Here (and in the associated Quartos) we ran edgeR as recommended and with default parameters and did not observe discordant results for reciprocal contrasts. Therefore, after checking this is true, we compare two sets of DESeq2 results for a reciprocal contrast to the only set of edgeR DEGs.

Run edgeR

```
folder <- "edgeR_for_pub"
dir.create(folder)
```

```
Warning in dir.create(folder): 'edgeR_for_pub' already exists
```

First, CLA_T1 v CLA_T0:

```
group <- dds$group
y <- DGEList(counts=mat.all,group=group)
dim(y$counts)
```

```
[1] 57905    18
```

```
keep <- filterByExpr(y)
y <- y[keep,,keep.lib.sizes=FALSE]
dim(y$counts)
```

```
[1] 17772    18
```

```
y <- normLibSizes(y)

design <- model.matrix(~group)
rownames(design) <- colnames(y)

y <- estimateDisp(y, design)
fit <- glmQLFit(y,design)
colnames(fit$design)
```

```
[1] "(Intercept)" "groupCLA_T1" "groupCLA_T2" "groupCLB_T0" "groupCLB_T1"
[6] "groupCLB_T2"
```

```
# coef = 2 is CLA_T1 vs CLA_T0
qlf <- glmQLFTest(fit,coef=2)
result_CLAT1vsCLAT0 <- topTags(qlf, n=nrow(y))
# check for missing adjusted p-values
any(is.na(as.data.frame(result_CLAT1vsCLAT0)$FDR))
```

```
[1] FALSE
```

```
# Adapt mirrorCheck's (private) plot_Volcano() to work for edgeR results
plot_Volcano_edgeR <- function(r,title, rowname2symbol = NULL,
                         p.cutoff = 0.1, fc.cutoff = 2, top.n = 30) {
  padj <- as.data.frame(r) %>%
    dplyr::filter(!is.na(FDR)) %>%
    dplyr::mutate(log10padj = -log10(FDR),
                  diff.expressed = dplyr::case_when(
                    logFC > fc.cutoff & FDR < p.cutoff ~ "UP",
                    logFC < -fc.cutoff & FDR < p.cutoff ~ "DOWN",
                    .default = "NO")) %>%
    tibble::rownames_to_column("rowname")
  if (is.null(rowname2symbol)) {
    padj <- padj %>%
      dplyr::mutate(g_symbol = rowname)
  } else {
    padj <- padj  %>%
      dplyr::left_join(rowname2symbol)
  }
  diff.ex <- padj %>% dplyr::filter(diff.expressed != "NO")
```

```r
  sums <- table(diff.ex$diff.expressed)
  maxy <- max(padj$log10padj)
  minx <- min(padj$logFC)
  maxx <- max(padj$logFC)
  padj$labels <- ifelse(padj$rowname %in% head(diff.ex[order(diff.ex$FDR),
                                                      "rowname"], top.n),
                        padj$g_symbol, NA)
  group.colors <- c(DOWN = '#CC3311',NO = "grey",UP = '#009988')
  group.labels <- c(DOWN = "Downregulated",
                    NO = "Not significant",
                    UP = "Upregulated")

  p <- ggplot2::ggplot(padj, ggplot2::aes(x = logFC,
                                          y = log10padj,
                                          col = diff.expressed,
                                          label = labels)) +
    ggplot2::theme_classic(base_size = 14) +
    ggplot2::labs(title = title,
                  x = expression("log"[2]*"FC"),
                  y = expression("-log"[10]*"adj-pvalue")) +
    ggplot2::theme(legend.title = element_blank()) +
    ggplot2::geom_vline(xintercept = c(-fc.cutoff, fc.cutoff),
                        col = "gray", linetype = 'dashed') +
    ggplot2::geom_hline(yintercept = -log10(p.cutoff),
                        col = "gray", linetype = 'dashed') +
    ggplot2::geom_point(size = 1.5) +
    ggplot2::scale_color_manual(values = group.colors,
                                labels = group.labels) +
    ggrepel::geom_text_repel(max.overlaps = Inf, size = 3,
                             color = "black", na.rm = T) +
    ggplot2::annotate("text",x = minx + 2, y = maxy - 1,
                      label = paste("DOWN",sums["DOWN"]),size = 4.5) +
    ggplot2::annotate("text",x = maxx - 1, y = maxy - 1,
                      label = paste("UP",sums["UP"]), size = 4.5)
}

p <- plot_Volcano_edgeR(result_CLAT1vsCLAT0, "CLA_T1_vs_CLA_T0 edgeR",
                        p.cutoff = 0.01,
                        top.n = 0)
p
```

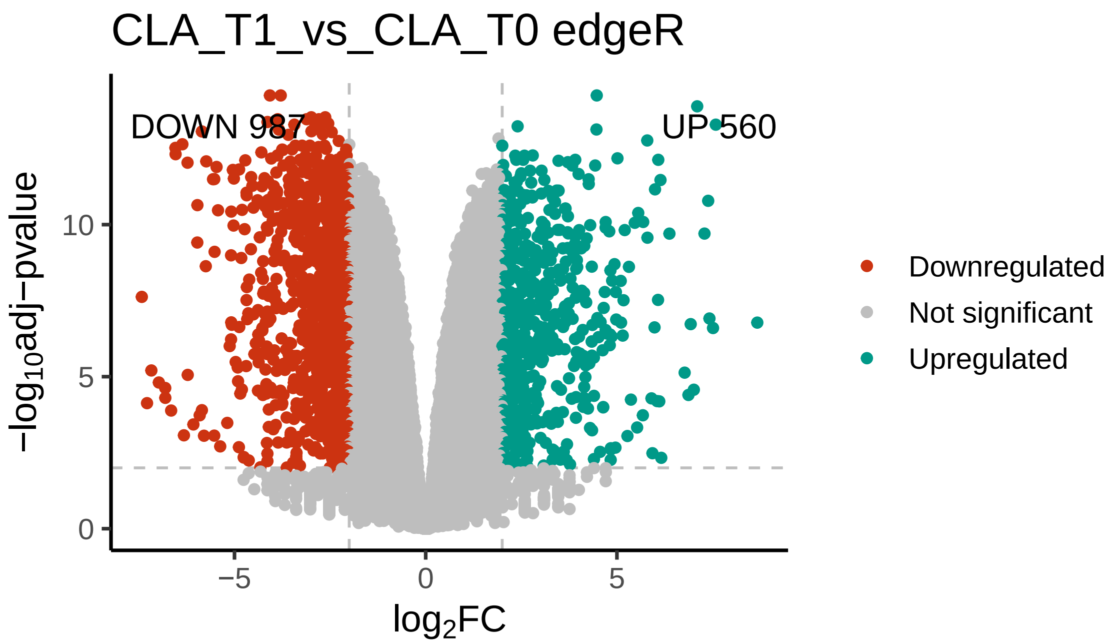

Second, relevel for comparison to CLA_T1

```
group <- relevel(group, ref = "CLA_T1")
levels(group)
```

```
[1] "CLA_T1" "CLA_T0" "CLA_T2" "CLB_T0" "CLB_T1" "CLB_T2"
```

```
y <- DGEList(counts=mat.all,group=group)
dim(y$counts)
```

```
[1] 57905    18
```

```
keep <- filterByExpr(y)
y <- y[keep,,keep.lib.sizes=FALSE]
dim(y$counts)
```

```
[1] 17772    18
```

```r
y <- normLibSizes(y)

design <- model.matrix(~group)
rownames(design) <- colnames(y)

y <- estimateDisp(y, design)
fit <- glmQLFit(y,design)
colnames(fit$design)
```

```
[1] "(Intercept)" "groupCLA_T0" "groupCLA_T2" "groupCLB_T0" "groupCLB_T1"
[6] "groupCLB_T2"
```

```r
# coef = 2 is  CLA_T0 vs CLA_T1
qlf2 <- glmQLFTest(fit,coef=2)
result_CLAT0vsCLAT1 <- topTags(qlf2, n=nrow(y))
# check for missing adjusted p-values
any(is.na(as.data.frame(result_CLAT0vsCLAT1)$FDR))
```

```
[1] FALSE
```

```r
p <- plot_Volcano_edgeR(result_CLAT0vsCLAT1, "CLA_T0_vs_CLA_T1 edgeR",
                        p.cutoff = 0.01,
                        top.n = 0)
p
```

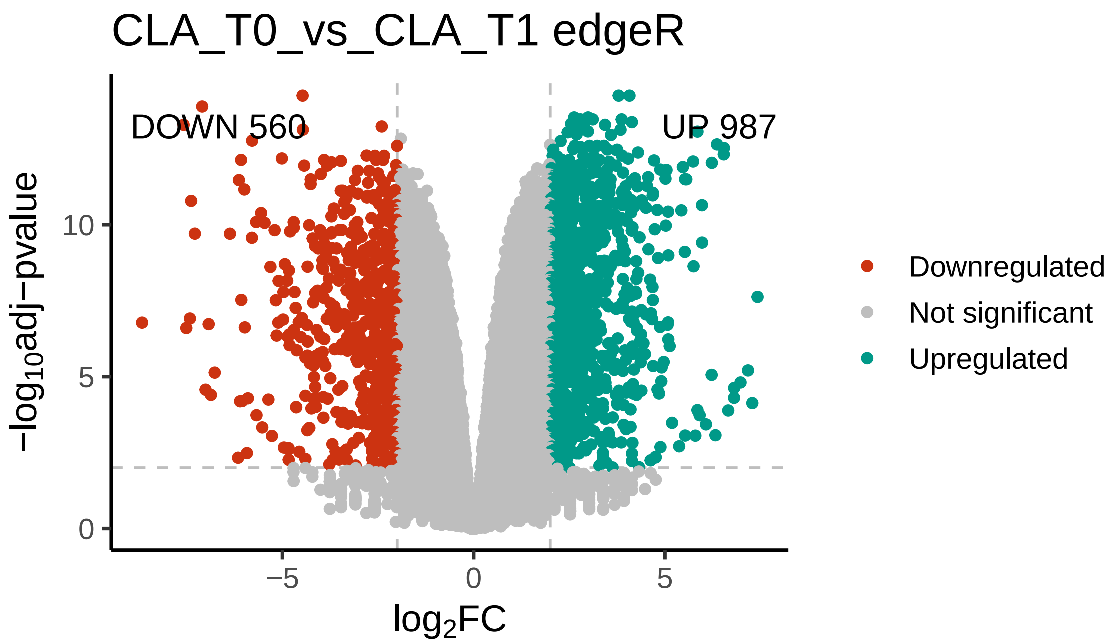


```
# Write results tables
write.csv(as.data.frame(result_CLAT1vsCLAT0),
          file = file.path(folder,"CLA_T1vsCLA_T0.csv"))
write.csv(as.data.frame(result_CLAT0vsCLAT1),
          file = file.path(folder,"CLA_T0vsCLA_T1.csv"))
```

Check: these results are perfectly mirrored so they give the same set of DEGs at FDR < 0.01 and logFC > 2 | logFC < 2

```
deg_1v0 <- as.data.frame(result_CLAT1vsCLAT0) %>%
  filter(FDR < 0.01 & (logFC < -2 | logFC > 2)) %>%
  rownames_to_column("name") %>%
  pull(name)
str(deg_1v0)
```

```
 chr [1:1547] "ENSG00000114779" "ENSG00000196700" "ENSG00000168237" ...
```

```
deg_0v1 <- as.data.frame(result_CLAT0vsCLAT1) %>%
  filter(FDR < 0.01 & (logFC < -2 | logFC > 2)) %>%
  rownames_to_column("name") %>%
  pull(name)
all(deg_1v0 == deg_0v1)
```

```
[1] TRUE
```

```
edger_DEG <- deg_1v0
```

DESeq2 DEGs compared to edgeR DEGs - without prefiltering dds

```
deseq_1v0 <- read.csv(file.path("DESeq_noclean","CLA_T1_vs_CLA_T0_DG.csv"))
table(deseq_1v0$padj < 0.01 &
        (deseq_1v0$log2FoldChange < -2 | deseq_1v0$log2FoldChange > 2))
```

```
TRUE
1431
```

```
deseq_0v1 <- read.csv(file.path("DESeq_noclean","CLA_T0_vs_CLA_T1_DG.csv"))
table(deseq_0v1$padj < 0.01 &
        (deseq_0v1$log2FoldChange < -2 | deseq_0v1$log2FoldChange > 2))
```

```
TRUE
1436
```

```
deseq_1v0_deg <- deseq_1v0 %>% pull(name)
deseq_0v1_deg <- deseq_0v1 %>% pull(name)

#unfiltered upset
list_with_unfiltdeseq <- list(edgeR = edger_DEG,
                              DESeq2_vCLAT0 = deseq_1v0_deg,
                              DESeq2_vCLAT1 = deseq_0v1_deg)
str(list_with_unfiltdeseq)
```

```
List of 3
 $ edgeR        : chr [1:1547] "ENSG00000114779" "ENSG00000196700" "ENSG00000168237" "ENSG00
 $ DESeq2_vCLAT0: chr [1:1431] "ENSG00000114779" "ENSG00000100596" "ENSG00000198938" "ENSG00
 $ DESeq2_vCLAT1: chr [1:1436] "ENSG00000114779" "ENSG00000100596" "ENSG00000198938" "ENSG00
```

```r
# the following function was sourced from github UpsetR issue 85, solution
# provided by 'docmanny' in September 2017
newFromList <- function (input) {
  # Same as original fromList()...
  elements <- unique(unlist(input))
  data <- unlist(lapply(input, function(x) {
    x <- as.vector(match(elements, x))
  }))
  data[is.na(data)] <- as.integer(0)
  data[data != 0] <- as.integer(1)
  data <- data.frame(matrix(data, ncol = length(input), byrow = F))
  data <- data[which(rowSums(data) != 0), ]
  names(data) <- names(input)
  # ... Except now it conserves your original value names!
  row.names(data) <- elements
  return(data)
}

for_upset_unfilt <- newFromList(list_with_unfiltdeseq) %>%
  mutate(concordant = if_else(DESeq2_vCLAT0 == 1 & DESeq2_vCLAT1 == 1, T, F))

upset_unfilt <- UpSetR::upset(for_upset_unfilt,
                              sets = c("edgeR",
                                       "DESeq2_vCLAT0",
                                       "DESeq2_vCLAT1"),
                              nintersects = NA,
                              mainbar.y.label = "DEGs per\nintersection",
                              sets.x.label = "Results set",
                              text.scale = 2,
                              empty.intersections = T,
                              keep.order = T,
                              mb.ratio = c(0.65, 0.35))
upset_unfilt
```

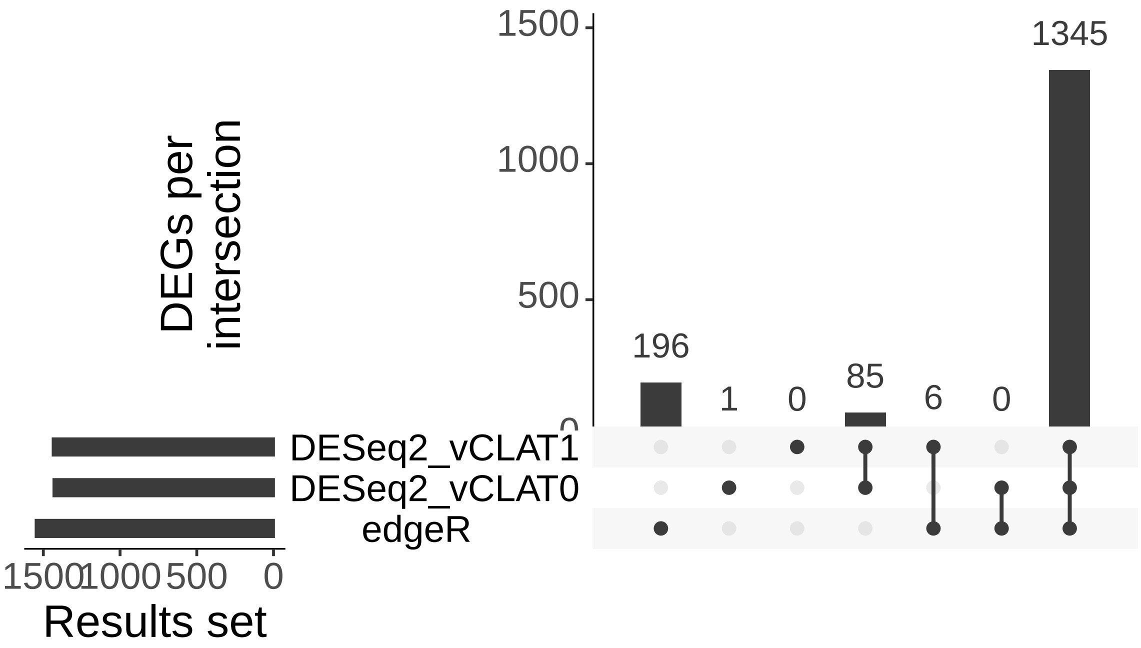

DESeq2 DEGs compared to edgeR DEGs - prefiltering dds

```
deseq_1v0_filt <- read.csv(file.path("DESeq_prefilt","CLA_T1_vs_CLA_T0_DG.csv"))
table(deseq_1v0_filt$padj < 0.01 & (deseq_1v0_filt$log2FoldChange < -2 |
                                      deseq_1v0_filt$log2FoldChange > 2))
```

```
TRUE
1379
```

```
deseq_0v1_filt <- read.csv(file.path("DESeq_prefilt","CLA_T0_vs_CLA_T1_DG.csv"))
table(deseq_0v1_filt$padj < 0.01 & (deseq_0v1_filt$log2FoldChange < -2 |
                                      deseq_0v1_filt$log2FoldChange > 2))
```

```
TRUE
1382
```

```r
deseq_1v0_deg_filt <- deseq_1v0_filt %>% pull(name)
deseq_0v1_deg_filt <- deseq_0v1_filt %>% pull(name)

#filtered upset
list_with_filtdeseq <- list(edgeR = edger_DEG,
                            DESeq2_vCLAT0 = deseq_1v0_deg_filt,
                            DESeq2_vCLAT1 = deseq_0v1_deg_filt)
str(list_with_filtdeseq)
```

```
List of 3
 $ edgeR        : chr [1:1547] "ENSG00000114779" "ENSG00000196700" "ENSG00000168237" "ENSG000
 $ DESeq2_vCLAT0: chr [1:1379] "ENSG00000114779" "ENSG00000100596" "ENSG00000198938" "ENSG000
 $ DESeq2_vCLAT1: chr [1:1382] "ENSG00000114779" "ENSG00000100596" "ENSG00000198938" "ENSG000
```

```r
for_upset_filt <- newFromList(list_with_filtdeseq) %>%
  mutate(concordant = if_else(DESeq2_vCLAT0 == 1 & DESeq2_vCLAT1 == 1, T, F))

upset_filt <- UpSetR::upset(for_upset_filt, sets = c("edgeR",
                                                     "DESeq2_vCLAT0",
                                                     "DESeq2_vCLAT1"),
                            nintersects = NA,
                            mainbar.y.label = "DEGs per\nintersection",
                            sets.x.label = "Results set",
                            text.scale = 2,
                            empty.intersections = T,
                            keep.order = T,
                            mb.ratio = c(0.65, 0.35))
upset_filt
```

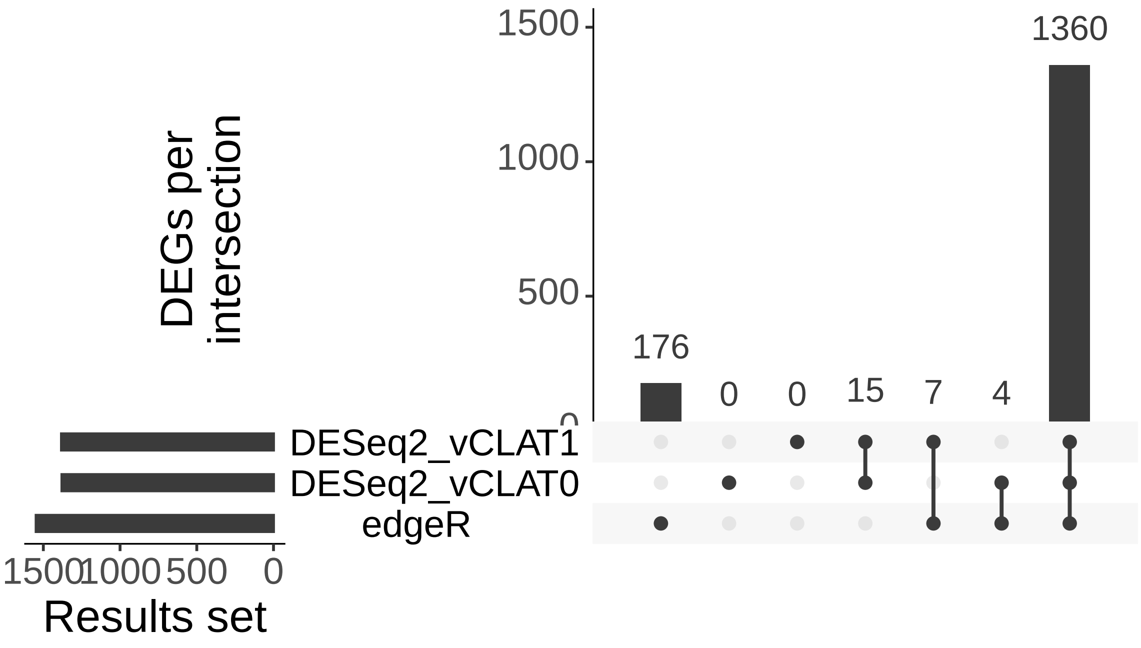

DESeq2 DEGs from prefiltered and non-prefiltered dds compared to edgeR DEGs on the same UpSet plot, with gene expression levels

```
list_with_bothdeseq <- list(edgeR = edger_DEG,
                            vCLAT0 = deseq_1v0_deg,
                            vCLAT1 = deseq_0v1_deg,
                            vCLAT0_filt = deseq_1v0_deg_filt,
                            vCLAT1_filt = deseq_0v1_deg_filt)
str(list_with_bothdeseq)
```

```
List of 5
 $ edgeR      : chr [1:1547] "ENSG00000114779" "ENSG00000196700" "ENSG00000168237" "ENSG0000(
 $ vCLAT0     : chr [1:1431] "ENSG00000114779" "ENSG00000100596" "ENSG00000198938" "ENSG0000(
 $ vCLAT1     : chr [1:1436] "ENSG00000114779" "ENSG00000100596" "ENSG00000198938" "ENSG0000(
 $ vCLAT0_filt: chr [1:1379] "ENSG00000114779" "ENSG00000100596" "ENSG00000198938" "ENSG0000(
 $ vCLAT1_filt: chr [1:1382] "ENSG00000114779" "ENSG00000100596" "ENSG00000198938" "ENSG0000(
```

```
for_upset_both <- newFromList(list_with_bothdeseq) %>%
  mutate(Concordant = if_else((vCLAT0 == 1 & vCLAT1 == 1) |
                              (vCLAT0_filt == 1 & vCLAT1_filt == 1),
```

```
                              T, F))
write.csv(for_upset_both,
              file = file.path(folder,"DESeq_edger_DEG_intersections.csv"),
              na="")

#Get results to extract mean normalised counts (baseMeans) for genes across
#samples
dds <- DESeq(dds)
```

```
using pre-existing size factors
```

```
estimating dispersions
```

```
found already estimated dispersions, replacing these
```

```
gene-wise dispersion estimates
```

```
mean-dispersion relationship
```

```
final dispersion estimates
```

```
fitting model and testing
```

```
res <- results(dds)
baseMeans <- as.data.frame(res) %>%
  rownames_to_column("name") %>%
  select(name,baseMean)
head(baseMeans)
```

```
             name    baseMean
1 ENSG00000000003    0.000000
2 ENSG00000000005    0.000000
3 ENSG00000000419 2200.418462
4 ENSG00000000457  748.402329
5 ENSG00000000460 1233.809647
6 ENSG00000000938    2.322733
```

```r
withNames <- for_upset_both %>% rownames_to_column("name")
forComplex <- withNames %>% left_join(baseMeans)
```

```
Joining with `by = join_by(name)`
```

```r
head(forComplex)
```

```
             name edgeR vCLAT0 vCLAT1 vCLAT0_filt vCLAT1_filt Concordant
1 ENSG00000114779     1      1      1           1           1       TRUE
2 ENSG00000196700     1      1      1           1           1       TRUE
3 ENSG00000168237     1      1      1           1           1       TRUE
4 ENSG00000015520     1      1      1           1           1       TRUE
5 ENSG00000137404     1      1      1           1           1       TRUE
6 ENSG00000100596     1      1      1           1           1       TRUE
   baseMean
1 2583.6836
2 2288.5970
3  520.0761
4  129.4638
5 2110.5559
6 1426.0118
```

```r
my_comparisons <- list( c(2,12), c(11,12))

ComplexUpset::upset(forComplex,c("edgeR",
                                  "vCLAT0",
                                  "vCLAT1",
                                  "vCLAT0_filt",
                                  "vCLAT1_filt"),
                    name = element_blank(),
                    sort_intersections_by=c("degree","ratio"),
                    sort_intersections = "ascending",
                    sort_sets=F,
                    annotations = list(
                      "log10 mean of\nnormalised counts"=list(
                        aes=aes(x=intersection, y=baseMean),
                        geom=list(geom_boxplot(),
                                  scale_y_continuous(trans='log10'),
                                  expand_limits(y = 1000000),
                                  stat_compare_means(comparisons=my_comparisons,
```

```
                                            label = "p.signif",
                                            size = 4,
                                            label.y = c(5,5.5)),
                              theme(axis.text.y = element_text(size = 10),
                                    axis.title.y=element_text(face="bold"))
                              )
                  )
                ),
                base_annotations=list(
                  'DEGs\nper intersection'=intersection_size(
                    mapping=aes(fill=Concordant),
                    text_colors = c(on_background = "black",
                                    on_bar = "black")) +
                    scale_fill_manual(values =c("TRUE" = "#009988",
                                                "FALSE" = "black")) +
                    theme(axis.text.y = element_text(size = 10),
                          axis.title.y = element_text(face="bold"))
                ),
                set_sizes=(
                  upset_set_size(position='right')
                  + ylab("DEGs per set")
                ),
                themes=upset_modify_themes(
                  list('intersections_matrix'=theme(
                    axis.text.y=element_text(size=12),
                    ))
                  ),

                width_ratio=0.2,
                height_ratio=0.9,
                guides='over'
              ) & theme(legend.margin=margin(margin(t = 180, r = 0,
                                                       b = 0, l = 0,
                                                       unit = "pt")))
```

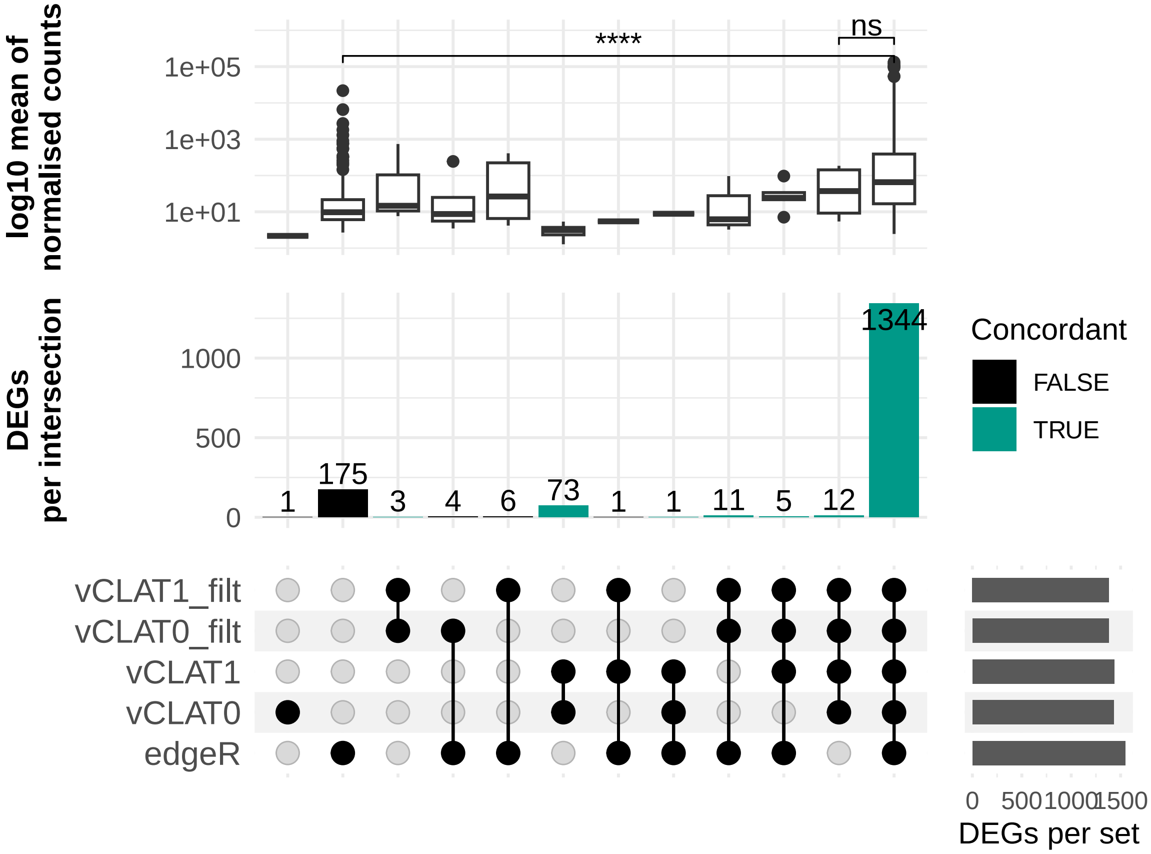

Session info and citations

Corchete, L. A., Rojas, E. A., Alonso-López, D., de Las Rivas, J., Gutiérrez, N. C., & Burguillo, F. J. (2020). Systematic comparison and assessment of RNA-seq procedures for gene expression quantitative analysis. Scientific Reports 2020 10:1, 10(1), 1–15. https://doi.org/10.1038/s41598-020-76881-x Love, M. I., Anders, S., Kim, V., & Huber, W. (2016). RNA-Seq workflow: gene-level exploratory analysis and differential expression. F1000Research 2016 4:1070, 4, 1070. https://doi.org/10.12688/f1000research.7035.2

Love, M. I., Anders, S., Kim, V., & Huber, W. (2016). RNA-Seq workflow: gene-level exploratory analysis and differential expression. F1000Research 2016 4:1070, 4, 1070. https://doi.org/10.12688/f1000research.7035.2

```
citation("DESeq2")
```

```
To cite package 'DESeq2' in publications use:
```

```
  Love, M.I., Huber, W., Anders, S. Moderated estimation of fold change
  and dispersion for RNA-seq data with DESeq2 Genome Biology 15(12):550
  (2014)

A BibTeX entry for LaTeX users is

  @Article{,
    title = {Moderated estimation of fold change and dispersion for RNA-seq data with DESeq2
    author = {Michael I. Love and Wolfgang Huber and Simon Anders},
    year = {2014},
    journal = {Genome Biology},
    doi = {10.1186/s13059-014-0550-8},
    volume = {15},
    issue = {12},
    pages = {550},
  }
```

```r
citation("edgeR")
```

```
See Section 1.2 in the User's Guide for more detail about how to cite
the different edgeR pipelines.

  Chen Y, Chen L, Lun ATL, Baldoni PL, Smyth GK (2024). edgeR 4.0:
  powerful differential analysis of sequencing data with expanded
  functionality and improved support for small counts and larger
  datasets. bioRxiv doi: 10.1101/2024.01.21.576131

  Chen Y, Lun ATL, Smyth GK (2016). From reads to genes to pathways:
  differential expression analysis of RNA-Seq experiments using
  Rsubread and the edgeR quasi-likelihood pipeline. F1000Research 5,
  1438

  McCarthy DJ, Chen Y and Smyth GK (2012). Differential expression
  analysis of multifactor RNA-Seq experiments with respect to
  biological variation. Nucleic Acids Research 40(10), 4288-4297

  Robinson MD, McCarthy DJ and Smyth GK (2010). edgeR: a Bioconductor
  package for differential expression analysis of digital gene
  expression data. Bioinformatics 26(1), 139-140

To see these entries in BibTeX format, use 'print(<citation>,
```

```
bibtex=TRUE)', 'toBibtex(.)', or set
'options(citation.bibtex.max=999)'.
```

```r
citation("apeglm")
```

```
To cite package 'apeglm' in publications use:

  Zhu, A., Ibrahim, J.G., Love, M.I. Heavy-tailed prior distributions
  for sequence count data: removing the noise and preserving large
  differences Bioinformatics (2018)

A BibTeX entry for LaTeX users is

  @Article{,
    title = {Heavy-tailed prior distributions for sequence count data: removing the noise and
    author = {Anqi Zhu and Joseph G. Ibrahim and Michael I. Love},
    year = {2018},
    journal = {Bioinformatics},
    doi = {10.1093/bioinformatics/bty895},
  }
```

```r
citation("sva")
```

```
To cite package 'sva' in publications use:

  Leek JT, Johnson WE, Parker HS, Fertig EJ, Jaffe AE, Zhang Y, Storey
  JD, Torres LC (2024). _sva: Surrogate Variable Analysis_.
  doi:10.18129/B9.bioc.sva <https://doi.org/10.18129/B9.bioc.sva>, R
  package version 3.52.0, <https://bioconductor.org/packages/sva>.

A BibTeX entry for LaTeX users is

  @Manual{,
    title = {sva: Surrogate Variable Analysis},
    author = {Jeffrey T. Leek and W. Evan Johnson and Hilary S. Parker and Elana J. Fertig a
    year = {2024},
    note = {R package version 3.52.0},
    url = {https://bioconductor.org/packages/sva},
    doi = {10.18129/B9.bioc.sva},
  }
```

```
ATTENTION: This citation information has been auto-generated from the
package DESCRIPTION file and may need manual editing, see
'help("citation")'.
```

```r
sessionInfo()
```

```
R version 4.4.2 (2024-10-31)
Platform: x86_64-pc-linux-gnu
Running under: Ubuntu 22.04.5 LTS

Matrix products: default
BLAS:   /usr/lib/x86_64-linux-gnu/blas/libblas.so.3.10.0
LAPACK: /usr/lib/x86_64-linux-gnu/lapack/liblapack.so.3.10.0

locale:
 [1] LC_CTYPE=en_AU.UTF-8       LC_NUMERIC=C
 [3] LC_TIME=en_AU.UTF-8        LC_COLLATE=en_AU.UTF-8
 [5] LC_MONETARY=en_AU.UTF-8    LC_MESSAGES=en_AU.UTF-8
 [7] LC_PAPER=en_AU.UTF-8       LC_NAME=C
 [9] LC_ADDRESS=C               LC_TELEPHONE=C
[11] LC_MEASUREMENT=en_AU.UTF-8 LC_IDENTIFICATION=C

time zone: Australia/Melbourne
tzcode source: system (glibc)

attached base packages:
[1] stats4    stats     graphics  grDevices utils     datasets  methods
[8] base

other attached packages:
 [1] sva_3.52.0                  BiocParallel_1.38.0
 [3] genefilter_1.86.0           mgcv_1.9-1
 [5] nlme_3.1-167                ggh4x_0.3.0
 [7] ggpubr_0.6.0                ComplexUpset_1.3.3
 [9] UpSetR_1.4.0                edgeR_4.2.2
[11] limma_3.60.6                lubridate_1.9.4
[13] forcats_1.0.0               stringr_1.5.1
[15] dplyr_1.1.4                 purrr_1.0.2
[17] readr_2.1.5                 tidyr_1.3.1
[19] tibble_3.2.1                ggplot2_3.5.1
[21] tidyverse_2.0.0             DESeq2_1.44.0
[23] SummarizedExperiment_1.34.0 Biobase_2.64.0
```

```
[25] MatrixGenerics_1.16.0       matrixStats_1.5.0
[27] GenomicRanges_1.56.2        GenomeInfoDb_1.40.1
[29] IRanges_2.38.1              S4Vectors_0.42.1
[31] BiocGenerics_0.50.0         mirrorCheck_0.0.1.0

loaded via a namespace (and not attached):
 [1] DBI_1.2.3               formatR_1.14            gridExtra_2.3
 [4] rlang_1.1.5             magrittr_2.0.3          compiler_4.4.2
 [7] RSQLite_2.3.9           png_0.1-8               vctrs_0.6.5
[10] pkgconfig_2.0.3         crayon_1.5.3            fastmap_1.2.0
[13] backports_1.5.0         XVector_0.44.0          labeling_0.4.3
[16] rmarkdown_2.29          tzdb_0.4.0              UCSC.utils_1.0.0
[19] bit_4.5.0.1             xfun_0.50               cachem_1.1.0
[22] zlibbioc_1.50.0         jsonlite_1.8.9          blob_1.2.4
[25] DelayedArray_0.30.1     broom_1.0.7             parallel_4.4.2
[28] R6_2.5.1                RColorBrewer_1.1-3      stringi_1.8.4
[31] car_3.1-3               numDeriv_2016.8-1.1     Rcpp_1.0.14
[34] knitr_1.49              VennDiagram_1.7.3       Matrix_1.7-2
[37] splines_4.4.2           timechange_0.3.0        tidyselect_1.2.1
[40] rstudioapi_0.17.1       abind_1.4-8             yaml_2.3.10
[43] codetools_0.2-19        lattice_0.22-5          plyr_1.8.9
[46] KEGGREST_1.44.1         withr_3.0.2             coda_0.19-4.1
[49] evaluate_1.0.3          lambda.r_1.2.4          survival_3.8-3
[52] futile.logger_1.4.3     Biostrings_2.72.1       pillar_1.10.1
[55] carData_3.0-5           generics_0.1.3          emdbook_1.3.13
[58] hms_1.1.3               munsell_0.5.1           scales_1.3.0
[61] xtable_1.8-4            glue_1.8.0              pheatmap_1.0.12
[64] apeglm_1.26.1           tools_4.4.2             annotate_1.82.0
[67] locfit_1.5-9.11         ggsignif_0.6.4          mvtnorm_1.3-3
[70] XML_3.99-0.18           grid_4.4.2              bbmle_1.0.25.1
[73] bdsmatrix_1.3-7         AnnotationDbi_1.66.0    colorspace_2.1-1
[76] GenomeInfoDbData_1.2.12 patchwork_1.3.0         Formula_1.2-5
[79] cli_3.6.3               futile.options_1.0.1    S4Arrays_1.4.1
[82] gtable_0.3.6            rstatix_0.7.2           digest_0.6.37
[85] ggrepel_0.9.6           SparseArray_1.4.8       farver_2.1.2
[88] memoise_2.0.1           htmltools_0.5.8.1       lifecycle_1.0.4
[91] httr_1.4.7              statmod_1.5.0           MASS_7.3-64
[94] bit64_4.6.0-1
```
