# Supplementary material for: mirrorCheck: an R package facilitating informed use of DESeq2’s lfcShrink() function for differential gene expression analysis of clinical samples
Source: Bioinform Adv. 2025 Apr 2;5(1):vbaf070. doi: 10.1093/bioadv/vbaf070 (PMC12089695; doi:10.1093/bioadv/vbaf070)
Supplement: vbaf070_Supplementary_Data [file vbaf070_supplementary_data.zip › final supps/S8_Supplementary_parathyroid_report.pdf]

# Parathyroid

Kate Scull

## ParathyroidSE

This analysis uses data from the Bioconductor experiment package "parathyroidSE", which contains data published in Haglund F, Ma R, Huss M, Sulaiman L, Lu M, Nilsson IL, Hoog A, Juhlin CC, Hartman J, Larsson C. 'Evidence of a Functional Estrogen Receptor in Parathyroid Adenomas.' J Clin Endocrinol Metab. jc.2012-2484, Epub 2012 Sep 28, PMID: 23024189. Since this data does not include gene symbols, this information is added using the biomaRt package. You will need to ensure to following R libraries are installed, including mirrorCheck from the github repository kescull/mirrorCheck: Facilitator functions for getting and assessing DESeq2 lfcShrink results (github.com).

## MirrorCheck

Setup and download:

```
library(mirrorCheck)
library(DESeq2)
library(tidyverse)
library(edgeR)
library(UpSetR)
library(ComplexUpset)
library(ggpubr)
library(parathyroidSE)
library(ggrepel)
library(biomaRt)
library(sva)
```

```
data("parathyroidGenesSE")
dds <- DESeqDataSet(parathyroidGenesSE, design = ~1)
meta <- data.frame(colData(dds)) %>%
  mutate(group = as.factor(paste(treatment,time,sep = "_"))) %>%
```

```
  rownames_to_column("name")
dds$group <- meta$group
dds$patient <- as.factor(dds$patient)
colData(dds)
```

```
DataFrame with 27 rows and 9 columns
            run experiment  patient treatment     time submission     study
    <character>   <factor> <factor> <factor> <factor>   <factor>  <factor>
1     SRR479052  SRX140503        1   Control      24h  SRA051611 SRP012167
2     SRR479053  SRX140504        1   Control      48h  SRA051611 SRP012167
3     SRR479054  SRX140505        1   DPN          24h  SRA051611 SRP012167
4     SRR479055  SRX140506        1   DPN          48h  SRA051611 SRP012167
5     SRR479056  SRX140507        1   OHT          24h  SRA051611 SRP012167
...         ...        ...      ...       ...      ...        ...       ...
23    SRR479074  SRX140523        4       DPN      48h  SRA051611 SRP012167
24    SRR479075  SRX140523        4       DPN      48h  SRA051611 SRP012167
25    SRR479076  SRX140524        4       OHT      24h  SRA051611 SRP012167
26    SRR479077  SRX140525        4       OHT      48h  SRA051611 SRP012167
27    SRR479078  SRX140525        4       OHT      48h  SRA051611 SRP012167
       sample       group
     <factor>    <factor>
1   SRS308865 Control_24h
2   SRS308866 Control_48h
3   SRS308867 DPN_24h
4   SRS308868 DPN_48h
5   SRS308869 OHT_24h
...       ...         ...
23  SRS308885     DPN_48h
24  SRS308885     DPN_48h
25  SRS308886     OHT_24h
26  SRS308887     OHT_48h
27  SRS308887     OHT_48h
```

```
design(dds) <- ~patient + group

mart <- useDataset("hsapiens_gene_ensembl", useMart("ensembl"))
genes <- rownames(dds)
G_list <- getBM(filters= "ensembl_gene_id",
                attributes= c("ensembl_gene_id","hgnc_symbol"),
                values=genes,
                mart= mart)
```

```
rowname2symbol <- G_list %>% rename(rowname = ensembl_gene_id,
                                    g_symbol = hgnc_symbol)
```

**Principal component analysis:**

```
vsd <- vst(dds)
pdata <- DESeq2::plotPCA(vsd, ntop = length(vsd), intgroup= "group",
                         returnData=T)
```

```
using ntop=63193 top features by variance
```

```
percentVar <- attr(pdata,"percentVar")
colour_blind_friendly <- c('#EE7733', '#0077BB','#BBBBBB','#EE3377',
                           '#33BBEE', '#CC3311', '#009988' )
joiner <- meta %>% dplyr::select(name, patient,treatment,time)
pdata <- pdata %>% left_join(joiner, by = "name") %>%
  group_by(patient) %>%
  mutate(id = row_number(),
         label = if_else(id == 3, paste("Patient",patient),NA))

p <- ggplot(pdata, aes(x=PC1,y=PC2,color=treatment,shape = time, label = label)) +
  geom_point(size =2, alpha = 0.7) +
  ggtitle("parathyroid") +
  labs(x = paste0("PC1: ",round(percentVar[1]*100),"% variance"),
       y = paste0("PC2: ",round(percentVar[2]*100),"% variance"),
       shape = "Time",
       color = "Treatment") +
  theme_classic(base_size = 16) +
  theme(plot.title = element_text(face = "bold")) +
  scale_colour_manual(values = colour_blind_friendly) +
  geom_text_repel(nudge_y = 5,size = 3, color = "black", na.rm = T, min.segment.length = 5)
p
```

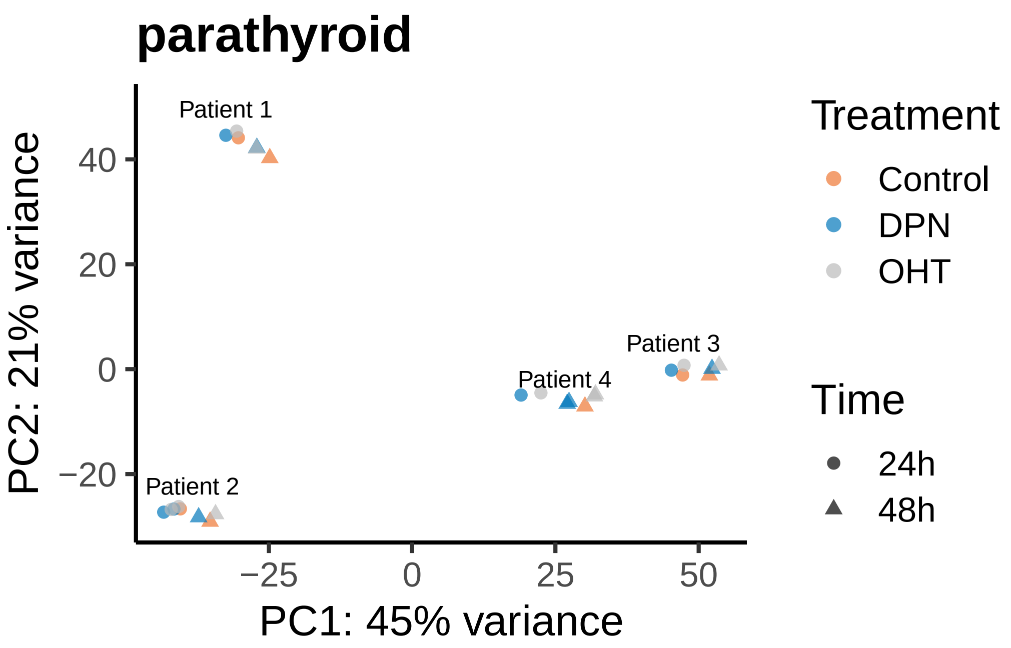


### Run DESeq2

Run DESeq2 using lfcShrink, facilitated by mirrorCheck, *without prefiltering or sva*. This creates output csv tables and pdf reports in a folder alongside this qmd file. From the PCA it is clear that there is high variability between patients, but here we are not interested in this variation; following the original paper, we are most interested in the differences between the treatments and control groups and how the different timing affects this. Above, we made a new group combining the treatment and time factors, as suggested by the DESeq2 vignette as an alternative to interaction terms. We have added 'patient' to the design so the differences attributable to this factor will be accounted for (`design = ~patient + group`).

#### DESeq on dds without data cleaning

First, we run DESeq on the whole dataset using mirrorCheck:

```
folder <- "DESeq_noclean"
dir.create(folder)
dds
```

```
class: DESeqDataSet
dim: 63193 27
metadata(2): MIAME version
```

```
assays(1): counts
rownames(63193): ENSG00000000003 ENSG00000000005 ... LRG_98 LRG_99
rowData names(0):
colnames: NULL
colData names(9): run experiment ... sample group
```

```r
run_DESeq_all_contrasts(dds,folder,
                        condition = "group",
                        mode="apeglm",p.cutoff = 0.05,
                        rowname2symbol = rowname2symbol,
                        top.n = 30,useDingbats = T,
                        fc.cutoff = 1,
                        print.all = T)
```

```
estimating size factors

estimating dispersions

gene-wise dispersion estimates

mean-dispersion relationship

final dispersion estimates

fitting model and testing

using 'apeglm' for LFC shrinkage. If used in published research, please cite:
    Zhu, A., Ibrahim, J.G., Love, M.I. (2018) Heavy-tailed prior distributions for
    sequence count data: removing the noise and preserving large differences.
    Bioinformatics. https://doi.org/10.1093/bioinformatics/bty895
using 'apeglm' for LFC shrinkage. If used in published research, please cite:
    Zhu, A., Ibrahim, J.G., Love, M.I. (2018) Heavy-tailed prior distributions for
    sequence count data: removing the noise and preserving large differences.
    Bioinformatics. https://doi.org/10.1093/bioinformatics/bty895
using 'apeglm' for LFC shrinkage. If used in published research, please cite:
    Zhu, A., Ibrahim, J.G., Love, M.I. (2018) Heavy-tailed prior distributions for
    sequence count data: removing the noise and preserving large differences.
    Bioinformatics. https://doi.org/10.1093/bioinformatics/bty895
using 'apeglm' for LFC shrinkage. If used in published research, please cite:
    Zhu, A., Ibrahim, J.G., Love, M.I. (2018) Heavy-tailed prior distributions for
```

```
    sequence count data: removing the noise and preserving large differences.
    Bioinformatics. https://doi.org/10.1093/bioinformatics/bty895
using 'apeglm' for LFC shrinkage. If used in published research, please cite:
    Zhu, A., Ibrahim, J.G., Love, M.I. (2018) Heavy-tailed prior distributions for
    sequence count data: removing the noise and preserving large differences.
    Bioinformatics. https://doi.org/10.1093/bioinformatics/bty895


Joining with `by = join_by(rowname)`
Joining with `by = join_by(rowname)`
Joining with `by = join_by(rowname)`
Joining with `by = join_by(rowname)`
Joining with `by = join_by(rowname)`
Joining with `by = join_by(name)`
Joining with `by = join_by(name)`
Joining with `by = join_by(name)`
Joining with `by = join_by(name)`
Joining with `by = join_by(name)`
Joining with `by = join_by(name)`
Joining with `by = join_by(name)`
Joining with `by = join_by(name)`
Joining with `by = join_by(name)`
Joining with `by = join_by(name)`
found results columns, replacing these
using 'apeglm' for LFC shrinkage. If used in published research, please cite:
Zhu, A., Ibrahim, J.G., Love, M.I. (2018) Heavy-tailed prior distributions for
sequence count data: removing the noise and preserving large differences.
Bioinformatics. https://doi.org/10.1093/bioinformatics/bty895
using 'apeglm' for LFC shrinkage. If used in published research, please cite:
Zhu, A., Ibrahim, J.G., Love, M.I. (2018) Heavy-tailed prior distributions for
sequence count data: removing the noise and preserving large differences.
Bioinformatics. https://doi.org/10.1093/bioinformatics/bty895
using 'apeglm' for LFC shrinkage. If used in published research, please cite:
Zhu, A., Ibrahim, J.G., Love, M.I. (2018) Heavy-tailed prior distributions for
sequence count data: removing the noise and preserving large differences.
Bioinformatics. https://doi.org/10.1093/bioinformatics/bty895
using 'apeglm' for LFC shrinkage. If used in published research, please cite:
Zhu, A., Ibrahim, J.G., Love, M.I. (2018) Heavy-tailed prior distributions for
sequence count data: removing the noise and preserving large differences.
Bioinformatics. https://doi.org/10.1093/bioinformatics/bty895
using 'apeglm' for LFC shrinkage. If used in published research, please cite:
Zhu, A., Ibrahim, J.G., Love, M.I. (2018) Heavy-tailed prior distributions for
sequence count data: removing the noise and preserving large differences.
```

```
Bioinformatics. https://doi.org/10.1093/bioinformatics/bty895
Joining with `by = join_by(rowname)`
Joining with `by = join_by(rowname)`
Joining with `by = join_by(rowname)`
Joining with `by = join_by(rowname)`
Joining with `by = join_by(rowname)`
Joining with `by = join_by(name)`
Joining with `by = join_by(name)`
Joining with `by = join_by(name)`
Joining with `by = join_by(name)`
Joining with `by = join_by(name)`
Joining with `by = join_by(name)`
Joining with `by = join_by(name)`
Joining with `by = join_by(name)`
Joining with `by = join_by(name)`
Joining with `by = join_by(name)`
found results columns, replacing these
using 'apeglm' for LFC shrinkage. If used in published research, please cite:
Zhu, A., Ibrahim, J.G., Love, M.I. (2018) Heavy-tailed prior distributions for
sequence count data: removing the noise and preserving large differences.
Bioinformatics. https://doi.org/10.1093/bioinformatics/bty895
using 'apeglm' for LFC shrinkage. If used in published research, please cite:
Zhu, A., Ibrahim, J.G., Love, M.I. (2018) Heavy-tailed prior distributions for
sequence count data: removing the noise and preserving large differences.
Bioinformatics. https://doi.org/10.1093/bioinformatics/bty895
using 'apeglm' for LFC shrinkage. If used in published research, please cite:
Zhu, A., Ibrahim, J.G., Love, M.I. (2018) Heavy-tailed prior distributions for
sequence count data: removing the noise and preserving large differences.
Bioinformatics. https://doi.org/10.1093/bioinformatics/bty895
using 'apeglm' for LFC shrinkage. If used in published research, please cite:
Zhu, A., Ibrahim, J.G., Love, M.I. (2018) Heavy-tailed prior distributions for
sequence count data: removing the noise and preserving large differences.
Bioinformatics. https://doi.org/10.1093/bioinformatics/bty895
using 'apeglm' for LFC shrinkage. If used in published research, please cite:
Zhu, A., Ibrahim, J.G., Love, M.I. (2018) Heavy-tailed prior distributions for
sequence count data: removing the noise and preserving large differences.
Bioinformatics. https://doi.org/10.1093/bioinformatics/bty895
Joining with `by = join_by(rowname)`
Joining with `by = join_by(rowname)`
Joining with `by = join_by(rowname)`
Joining with `by = join_by(rowname)`
Joining with `by = join_by(rowname)`
Joining with `by = join_by(name)`
```

```
Joining with `by = join_by(name)`
Joining with `by = join_by(name)`
Joining with `by = join_by(name)`
Joining with `by = join_by(name)`
Joining with `by = join_by(name)`
Joining with `by = join_by(name)`
Joining with `by = join_by(name)`
Joining with `by = join_by(name)`
Joining with `by = join_by(name)`
found results columns, replacing these
using 'apeglm' for LFC shrinkage. If used in published research, please cite:
Zhu, A., Ibrahim, J.G., Love, M.I. (2018) Heavy-tailed prior distributions for
sequence count data: removing the noise and preserving large differences.
Bioinformatics. https://doi.org/10.1093/bioinformatics/bty895
using 'apeglm' for LFC shrinkage. If used in published research, please cite:
Zhu, A., Ibrahim, J.G., Love, M.I. (2018) Heavy-tailed prior distributions for
sequence count data: removing the noise and preserving large differences.
Bioinformatics. https://doi.org/10.1093/bioinformatics/bty895
using 'apeglm' for LFC shrinkage. If used in published research, please cite:
Zhu, A., Ibrahim, J.G., Love, M.I. (2018) Heavy-tailed prior distributions for
sequence count data: removing the noise and preserving large differences.
Bioinformatics. https://doi.org/10.1093/bioinformatics/bty895
using 'apeglm' for LFC shrinkage. If used in published research, please cite:
Zhu, A., Ibrahim, J.G., Love, M.I. (2018) Heavy-tailed prior distributions for
sequence count data: removing the noise and preserving large differences.
Bioinformatics. https://doi.org/10.1093/bioinformatics/bty895
using 'apeglm' for LFC shrinkage. If used in published research, please cite:
Zhu, A., Ibrahim, J.G., Love, M.I. (2018) Heavy-tailed prior distributions for
sequence count data: removing the noise and preserving large differences.
Bioinformatics. https://doi.org/10.1093/bioinformatics/bty895
Joining with `by = join_by(rowname)`
Joining with `by = join_by(rowname)`
Joining with `by = join_by(rowname)`
Joining with `by = join_by(rowname)`
Joining with `by = join_by(rowname)`
Joining with `by = join_by(name)`
Joining with `by = join_by(name)`
Joining with `by = join_by(name)`
Joining with `by = join_by(name)`
Joining with `by = join_by(name)`
Joining with `by = join_by(name)`
Joining with `by = join_by(name)`
Joining with `by = join_by(name)`
```

```
Joining with `by = join_by(name)`
Joining with `by = join_by(name)`
found results columns, replacing these
using 'apeglm' for LFC shrinkage. If used in published research, please cite:
Zhu, A., Ibrahim, J.G., Love, M.I. (2018) Heavy-tailed prior distributions for
sequence count data: removing the noise and preserving large differences.
Bioinformatics. https://doi.org/10.1093/bioinformatics/bty895
using 'apeglm' for LFC shrinkage. If used in published research, please cite:
Zhu, A., Ibrahim, J.G., Love, M.I. (2018) Heavy-tailed prior distributions for
sequence count data: removing the noise and preserving large differences.
Bioinformatics. https://doi.org/10.1093/bioinformatics/bty895
using 'apeglm' for LFC shrinkage. If used in published research, please cite:
Zhu, A., Ibrahim, J.G., Love, M.I. (2018) Heavy-tailed prior distributions for
sequence count data: removing the noise and preserving large differences.
Bioinformatics. https://doi.org/10.1093/bioinformatics/bty895
using 'apeglm' for LFC shrinkage. If used in published research, please cite:
Zhu, A., Ibrahim, J.G., Love, M.I. (2018) Heavy-tailed prior distributions for
sequence count data: removing the noise and preserving large differences.
Bioinformatics. https://doi.org/10.1093/bioinformatics/bty895
using 'apeglm' for LFC shrinkage. If used in published research, please cite:
Zhu, A., Ibrahim, J.G., Love, M.I. (2018) Heavy-tailed prior distributions for
sequence count data: removing the noise and preserving large differences.
Bioinformatics. https://doi.org/10.1093/bioinformatics/bty895
Joining with `by = join_by(rowname)`
Joining with `by = join_by(rowname)`
Joining with `by = join_by(rowname)`
Joining with `by = join_by(rowname)`
Joining with `by = join_by(rowname)`
Joining with `by = join_by(name)`
Joining with `by = join_by(name)`
Joining with `by = join_by(name)`
Joining with `by = join_by(name)`
Joining with `by = join_by(name)`
Joining with `by = join_by(name)`
Joining with `by = join_by(name)`
Joining with `by = join_by(name)`
Joining with `by = join_by(name)`
Joining with `by = join_by(name)`
found results columns, replacing these
using 'apeglm' for LFC shrinkage. If used in published research, please cite:
Zhu, A., Ibrahim, J.G., Love, M.I. (2018) Heavy-tailed prior distributions for
sequence count data: removing the noise and preserving large differences.
Bioinformatics. https://doi.org/10.1093/bioinformatics/bty895
```

```
using 'apeglm' for LFC shrinkage. If used in published research, please cite:
Zhu, A., Ibrahim, J.G., Love, M.I. (2018) Heavy-tailed prior distributions for
sequence count data: removing the noise and preserving large differences.
Bioinformatics. https://doi.org/10.1093/bioinformatics/bty895
using 'apeglm' for LFC shrinkage. If used in published research, please cite:
Zhu, A., Ibrahim, J.G., Love, M.I. (2018) Heavy-tailed prior distributions for
sequence count data: removing the noise and preserving large differences.
Bioinformatics. https://doi.org/10.1093/bioinformatics/bty895
using 'apeglm' for LFC shrinkage. If used in published research, please cite:
Zhu, A., Ibrahim, J.G., Love, M.I. (2018) Heavy-tailed prior distributions for
sequence count data: removing the noise and preserving large differences.
Bioinformatics. https://doi.org/10.1093/bioinformatics/bty895
using 'apeglm' for LFC shrinkage. If used in published research, please cite:
Zhu, A., Ibrahim, J.G., Love, M.I. (2018) Heavy-tailed prior distributions for
sequence count data: removing the noise and preserving large differences.
Bioinformatics. https://doi.org/10.1093/bioinformatics/bty895
Joining with `by = join_by(rowname)`
Joining with `by = join_by(rowname)`
Joining with `by = join_by(rowname)`
Joining with `by = join_by(rowname)`
Joining with `by = join_by(rowname)`
Joining with `by = join_by(name)`
Joining with `by = join_by(name)`
Joining with `by = join_by(name)`
Joining with `by = join_by(name)`
Joining with `by = join_by(name)`
Joining with `by = join_by(name)`
Joining with `by = join_by(name)`
Joining with `by = join_by(name)`
Joining with `by = join_by(name)`
Joining with `by = join_by(name)`
```

```r
group <- levels(as.factor(dds$group))
noclean_set <- compare_reciprocal_contrasts(group,folder)
```

```
[1] "Found no regulated genes for  OHT_48h  and  DPN_48h"
```

```
[1] "Found no regulated genes for  OHT_48h  and  Control_48h"
```

```
[1] "Found no regulated genes for  OHT_24h  and  Control_24h"
```

```
[1] "No overlap of regulated genes using different reference level for DPN_48h and Control_48
```

```
[1] "Found no regulated genes for  DPN_24h  and  Control_24h"
```

```
Warning: Groups with fewer than two data points have been dropped.
```

```
Warning in max(ids, na.rm = TRUE): no non-missing arguments to max; returning
-Inf
```

```
saveRDS(noclean_set, "noclean_set.rds")
```

### DESeq on prefiltered dds

Second, we prefilter the dds to see if that improves consensus in mirrorCheck or helps identify more DEGs.

```
folder <- "DESeq_prefilt"
dir.create(folder)
expr.filter <- edgeR::filterByExpr(dds, group = dds$group)
dds.filt <- dds[expr.filter, ]
dds.filt
```

```
class: DESeqDataSet
dim: 16641 27
metadata(2): MIAME version
assays(1): counts
rownames(16641): ENSG00000000003 ENSG00000000419 ... ENSG00000271646
  ENSG00000271707
rowData names(0):
colnames: NULL
colData names(9): run experiment ... sample group
```

```
run_DESeq_all_contrasts(dds.filt,folder,
                        condition = "group",
                        mode="apeglm",p.cutoff = 0.05,
                        rowname2symbol = rowname2symbol,
                        top.n = 30,useDingbats = T,
                        fc.cutoff = 1,
                        print.all = T)
```

```
estimating size factors

estimating dispersions

gene-wise dispersion estimates

mean-dispersion relationship

final dispersion estimates

fitting model and testing

using 'apeglm' for LFC shrinkage. If used in published research, please cite:
    Zhu, A., Ibrahim, J.G., Love, M.I. (2018) Heavy-tailed prior distributions for
    sequence count data: removing the noise and preserving large differences.
    Bioinformatics. https://doi.org/10.1093/bioinformatics/bty895
using 'apeglm' for LFC shrinkage. If used in published research, please cite:
    Zhu, A., Ibrahim, J.G., Love, M.I. (2018) Heavy-tailed prior distributions for
    sequence count data: removing the noise and preserving large differences.
    Bioinformatics. https://doi.org/10.1093/bioinformatics/bty895
using 'apeglm' for LFC shrinkage. If used in published research, please cite:
    Zhu, A., Ibrahim, J.G., Love, M.I. (2018) Heavy-tailed prior distributions for
    sequence count data: removing the noise and preserving large differences.
    Bioinformatics. https://doi.org/10.1093/bioinformatics/bty895
using 'apeglm' for LFC shrinkage. If used in published research, please cite:
    Zhu, A., Ibrahim, J.G., Love, M.I. (2018) Heavy-tailed prior distributions for
    sequence count data: removing the noise and preserving large differences.
    Bioinformatics. https://doi.org/10.1093/bioinformatics/bty895
using 'apeglm' for LFC shrinkage. If used in published research, please cite:
    Zhu, A., Ibrahim, J.G., Love, M.I. (2018) Heavy-tailed prior distributions for
    sequence count data: removing the noise and preserving large differences.
    Bioinformatics. https://doi.org/10.1093/bioinformatics/bty895

Joining with `by = join_by(rowname)`
Joining with `by = join_by(rowname)`
Joining with `by = join_by(rowname)`
Joining with `by = join_by(rowname)`
Joining with `by = join_by(rowname)`
Joining with `by = join_by(name)`
Joining with `by = join_by(name)`
```

```
Joining with `by = join_by(name)`
Joining with `by = join_by(name)`
Joining with `by = join_by(name)`
Joining with `by = join_by(name)`
Joining with `by = join_by(name)`
Joining with `by = join_by(name)`
Joining with `by = join_by(name)`
Joining with `by = join_by(name)`
found results columns, replacing these
using 'apeglm' for LFC shrinkage. If used in published research, please cite:
Zhu, A., Ibrahim, J.G., Love, M.I. (2018) Heavy-tailed prior distributions for
sequence count data: removing the noise and preserving large differences.
Bioinformatics. https://doi.org/10.1093/bioinformatics/bty895
using 'apeglm' for LFC shrinkage. If used in published research, please cite:
Zhu, A., Ibrahim, J.G., Love, M.I. (2018) Heavy-tailed prior distributions for
sequence count data: removing the noise and preserving large differences.
Bioinformatics. https://doi.org/10.1093/bioinformatics/bty895
using 'apeglm' for LFC shrinkage. If used in published research, please cite:
Zhu, A., Ibrahim, J.G., Love, M.I. (2018) Heavy-tailed prior distributions for
sequence count data: removing the noise and preserving large differences.
Bioinformatics. https://doi.org/10.1093/bioinformatics/bty895
using 'apeglm' for LFC shrinkage. If used in published research, please cite:
Zhu, A., Ibrahim, J.G., Love, M.I. (2018) Heavy-tailed prior distributions for
sequence count data: removing the noise and preserving large differences.
Bioinformatics. https://doi.org/10.1093/bioinformatics/bty895
using 'apeglm' for LFC shrinkage. If used in published research, please cite:
Zhu, A., Ibrahim, J.G., Love, M.I. (2018) Heavy-tailed prior distributions for
sequence count data: removing the noise and preserving large differences.
Bioinformatics. https://doi.org/10.1093/bioinformatics/bty895
Joining with `by = join_by(rowname)`
Joining with `by = join_by(rowname)`
Joining with `by = join_by(rowname)`
Joining with `by = join_by(rowname)`
Joining with `by = join_by(rowname)`
Joining with `by = join_by(name)`
Joining with `by = join_by(name)`
Joining with `by = join_by(name)`
Joining with `by = join_by(name)`
Joining with `by = join_by(name)`
Joining with `by = join_by(name)`
Joining with `by = join_by(name)`
Joining with `by = join_by(name)`
Joining with `by = join_by(name)`
```

```
Joining with `by = join_by(name)`
found results columns, replacing these
using 'apeglm' for LFC shrinkage. If used in published research, please cite:
Zhu, A., Ibrahim, J.G., Love, M.I. (2018) Heavy-tailed prior distributions for
sequence count data: removing the noise and preserving large differences.
Bioinformatics. https://doi.org/10.1093/bioinformatics/bty895
using 'apeglm' for LFC shrinkage. If used in published research, please cite:
Zhu, A., Ibrahim, J.G., Love, M.I. (2018) Heavy-tailed prior distributions for
sequence count data: removing the noise and preserving large differences.
Bioinformatics. https://doi.org/10.1093/bioinformatics/bty895
using 'apeglm' for LFC shrinkage. If used in published research, please cite:
Zhu, A., Ibrahim, J.G., Love, M.I. (2018) Heavy-tailed prior distributions for
sequence count data: removing the noise and preserving large differences.
Bioinformatics. https://doi.org/10.1093/bioinformatics/bty895
using 'apeglm' for LFC shrinkage. If used in published research, please cite:
Zhu, A., Ibrahim, J.G., Love, M.I. (2018) Heavy-tailed prior distributions for
sequence count data: removing the noise and preserving large differences.
Bioinformatics. https://doi.org/10.1093/bioinformatics/bty895
using 'apeglm' for LFC shrinkage. If used in published research, please cite:
Zhu, A., Ibrahim, J.G., Love, M.I. (2018) Heavy-tailed prior distributions for
sequence count data: removing the noise and preserving large differences.
Bioinformatics. https://doi.org/10.1093/bioinformatics/bty895
Joining with `by = join_by(rowname)`
Joining with `by = join_by(rowname)`
Joining with `by = join_by(rowname)`
Joining with `by = join_by(rowname)`
Joining with `by = join_by(rowname)`
Joining with `by = join_by(name)`
Joining with `by = join_by(name)`
Joining with `by = join_by(name)`
Joining with `by = join_by(name)`
Joining with `by = join_by(name)`
Joining with `by = join_by(name)`
Joining with `by = join_by(name)`
Joining with `by = join_by(name)`
Joining with `by = join_by(name)`
Joining with `by = join_by(name)`
found results columns, replacing these
using 'apeglm' for LFC shrinkage. If used in published research, please cite:
Zhu, A., Ibrahim, J.G., Love, M.I. (2018) Heavy-tailed prior distributions for
sequence count data: removing the noise and preserving large differences.
Bioinformatics. https://doi.org/10.1093/bioinformatics/bty895
using 'apeglm' for LFC shrinkage. If used in published research, please cite:
```

```
Zhu, A., Ibrahim, J.G., Love, M.I. (2018) Heavy-tailed prior distributions for
sequence count data: removing the noise and preserving large differences.
Bioinformatics. https://doi.org/10.1093/bioinformatics/bty895
using 'apeglm' for LFC shrinkage. If used in published research, please cite:
Zhu, A., Ibrahim, J.G., Love, M.I. (2018) Heavy-tailed prior distributions for
sequence count data: removing the noise and preserving large differences.
Bioinformatics. https://doi.org/10.1093/bioinformatics/bty895
using 'apeglm' for LFC shrinkage. If used in published research, please cite:
Zhu, A., Ibrahim, J.G., Love, M.I. (2018) Heavy-tailed prior distributions for
sequence count data: removing the noise and preserving large differences.
Bioinformatics. https://doi.org/10.1093/bioinformatics/bty895
using 'apeglm' for LFC shrinkage. If used in published research, please cite:
Zhu, A., Ibrahim, J.G., Love, M.I. (2018) Heavy-tailed prior distributions for
sequence count data: removing the noise and preserving large differences.
Bioinformatics. https://doi.org/10.1093/bioinformatics/bty895
Joining with `by = join_by(rowname)`
Joining with `by = join_by(rowname)`
Joining with `by = join_by(rowname)`
Joining with `by = join_by(rowname)`
Joining with `by = join_by(rowname)`
Joining with `by = join_by(name)`
Joining with `by = join_by(name)`
Joining with `by = join_by(name)`
Joining with `by = join_by(name)`
Joining with `by = join_by(name)`
Joining with `by = join_by(name)`
Joining with `by = join_by(name)`
Joining with `by = join_by(name)`
Joining with `by = join_by(name)`
Joining with `by = join_by(name)`
found results columns, replacing these
using 'apeglm' for LFC shrinkage. If used in published research, please cite:
Zhu, A., Ibrahim, J.G., Love, M.I. (2018) Heavy-tailed prior distributions for
sequence count data: removing the noise and preserving large differences.
Bioinformatics. https://doi.org/10.1093/bioinformatics/bty895
using 'apeglm' for LFC shrinkage. If used in published research, please cite:
Zhu, A., Ibrahim, J.G., Love, M.I. (2018) Heavy-tailed prior distributions for
sequence count data: removing the noise and preserving large differences.
Bioinformatics. https://doi.org/10.1093/bioinformatics/bty895
using 'apeglm' for LFC shrinkage. If used in published research, please cite:
Zhu, A., Ibrahim, J.G., Love, M.I. (2018) Heavy-tailed prior distributions for
sequence count data: removing the noise and preserving large differences.
Bioinformatics. https://doi.org/10.1093/bioinformatics/bty895
```

```
using 'apeglm' for LFC shrinkage. If used in published research, please cite:
Zhu, A., Ibrahim, J.G., Love, M.I. (2018) Heavy-tailed prior distributions for
sequence count data: removing the noise and preserving large differences.
Bioinformatics. https://doi.org/10.1093/bioinformatics/bty895
using 'apeglm' for LFC shrinkage. If used in published research, please cite:
Zhu, A., Ibrahim, J.G., Love, M.I. (2018) Heavy-tailed prior distributions for
sequence count data: removing the noise and preserving large differences.
Bioinformatics. https://doi.org/10.1093/bioinformatics/bty895
Joining with `by = join_by(rowname)`
Joining with `by = join_by(rowname)`
Joining with `by = join_by(rowname)`
Joining with `by = join_by(rowname)`
Joining with `by = join_by(rowname)`
Joining with `by = join_by(name)`
Joining with `by = join_by(name)`
Joining with `by = join_by(name)`
Joining with `by = join_by(name)`
Joining with `by = join_by(name)`
Joining with `by = join_by(name)`
Joining with `by = join_by(name)`
Joining with `by = join_by(name)`
Joining with `by = join_by(name)`
Joining with `by = join_by(name)`
found results columns, replacing these
using 'apeglm' for LFC shrinkage. If used in published research, please cite:
Zhu, A., Ibrahim, J.G., Love, M.I. (2018) Heavy-tailed prior distributions for
sequence count data: removing the noise and preserving large differences.
Bioinformatics. https://doi.org/10.1093/bioinformatics/bty895
using 'apeglm' for LFC shrinkage. If used in published research, please cite:
Zhu, A., Ibrahim, J.G., Love, M.I. (2018) Heavy-tailed prior distributions for
sequence count data: removing the noise and preserving large differences.
Bioinformatics. https://doi.org/10.1093/bioinformatics/bty895
using 'apeglm' for LFC shrinkage. If used in published research, please cite:
Zhu, A., Ibrahim, J.G., Love, M.I. (2018) Heavy-tailed prior distributions for
sequence count data: removing the noise and preserving large differences.
Bioinformatics. https://doi.org/10.1093/bioinformatics/bty895
using 'apeglm' for LFC shrinkage. If used in published research, please cite:
Zhu, A., Ibrahim, J.G., Love, M.I. (2018) Heavy-tailed prior distributions for
sequence count data: removing the noise and preserving large differences.
Bioinformatics. https://doi.org/10.1093/bioinformatics/bty895
using 'apeglm' for LFC shrinkage. If used in published research, please cite:
Zhu, A., Ibrahim, J.G., Love, M.I. (2018) Heavy-tailed prior distributions for
sequence count data: removing the noise and preserving large differences.
```

```
Bioinformatics. https://doi.org/10.1093/bioinformatics/bty895
Joining with `by = join_by(rowname)`
Joining with `by = join_by(rowname)`
Joining with `by = join_by(rowname)`
Joining with `by = join_by(rowname)`
Joining with `by = join_by(rowname)`
Joining with `by = join_by(name)`
Joining with `by = join_by(name)`
Joining with `by = join_by(name)`
Joining with `by = join_by(name)`
Joining with `by = join_by(name)`
Joining with `by = join_by(name)`
Joining with `by = join_by(name)`
Joining with `by = join_by(name)`
Joining with `by = join_by(name)`
Joining with `by = join_by(name)`
```

```
group <- levels(as.factor(dds.filt$group))
prefilt_set <- compare_reciprocal_contrasts(group,folder)
```

```
[1] "Found no regulated genes for  OHT_48h  and  DPN_48h"

[1] "Found no regulated genes for  OHT_48h  and  Control_48h"

[1] "Found no regulated genes for  OHT_24h  and  DPN_24h"

[1] "Found no regulated genes for  OHT_24h  and  Control_24h"

[1] "No overlap of regulated genes using different reference level for DPN_48h and Control_48

[1] "Found no regulated genes for  DPN_24h  and  Control_24h"
```

```
saveRDS(prefilt_set, "prefilt_set.rds")
```

### DESeq on dds with sva

The presence of unknown sources of variation can interfere with DGEA and increase discordance in reciprocal contrasts, so here we try surrogate variate analysis (SVA) before running DESeq with mirrorCheck, as per the method in Love et al. (2016).

```
folder <- "DESeq_sva"
dir.create(folder)

dds <- DESeq(dds)
```

```
estimating size factors

estimating dispersions

gene-wise dispersion estimates

mean-dispersion relationship

final dispersion estimates

fitting model and testing
```

```
dat <- counts(dds, normalized=TRUE)
idx <- rowMeans(dat) > 1
dat <- dat[idx,]
mod <- model.matrix(~ patient + group, colData(dds))
mod0 <- model.matrix(~ patient, colData(dds))

#find probable number of unwanted variables
num_sv <- num.sv(dat,mod,method = "be")
num_sv
```

```
[1] 3
```

```
#num_sv = 3
svseq <- svaseq(dat, mod, mod0, n.sv=num_sv)
```

```
Number of significant surrogate variables is:  3
Iteration (out of 5 ):1  2  3  4  5
```

```r
dds.sva <- dds
dds.sva$SV1 <- svseq$sv[,1]
dds.sva$SV2 <- svseq$sv[,2]
dds.sva$SV3 <- svseq$sv[,3]
design(dds.sva) <- ~ SV1 + SV2 + SV3 + patient + group

run_DESeq_all_contrasts(dds.sva,folder,
                        condition = "group",
                        mode="apeglm",p.cutoff = 0.05,
                        rowname2symbol = rowname2symbol,
                        top.n = 30,useDingbats = T,
                        fc.cutoff = 1,
                        print.all = T)
```

```
using pre-existing size factors

estimating dispersions

found already estimated dispersions, replacing these

gene-wise dispersion estimates

mean-dispersion relationship

final dispersion estimates

fitting model and testing

1313 rows did not converge in beta, labelled in mcols(object)$betaConv. Use larger maxit argu

using 'apeglm' for LFC shrinkage. If used in published research, please cite:
    Zhu, A., Ibrahim, J.G., Love, M.I. (2018) Heavy-tailed prior distributions for
    sequence count data: removing the noise and preserving large differences.
    Bioinformatics. https://doi.org/10.1093/bioinformatics/bty895
using 'apeglm' for LFC shrinkage. If used in published research, please cite:
    Zhu, A., Ibrahim, J.G., Love, M.I. (2018) Heavy-tailed prior distributions for
    sequence count data: removing the noise and preserving large differences.
    Bioinformatics. https://doi.org/10.1093/bioinformatics/bty895
using 'apeglm' for LFC shrinkage. If used in published research, please cite:
```

```
    Zhu, A., Ibrahim, J.G., Love, M.I. (2018) Heavy-tailed prior distributions for
    sequence count data: removing the noise and preserving large differences.
    Bioinformatics. https://doi.org/10.1093/bioinformatics/bty895
using 'apeglm' for LFC shrinkage. If used in published research, please cite:
    Zhu, A., Ibrahim, J.G., Love, M.I. (2018) Heavy-tailed prior distributions for
    sequence count data: removing the noise and preserving large differences.
    Bioinformatics. https://doi.org/10.1093/bioinformatics/bty895
using 'apeglm' for LFC shrinkage. If used in published research, please cite:
    Zhu, A., Ibrahim, J.G., Love, M.I. (2018) Heavy-tailed prior distributions for
    sequence count data: removing the noise and preserving large differences.
    Bioinformatics. https://doi.org/10.1093/bioinformatics/bty895


some rows did not converge in finding the MAP


Joining with `by = join_by(rowname)`
Joining with `by = join_by(rowname)`
Joining with `by = join_by(rowname)`
Joining with `by = join_by(rowname)`
Joining with `by = join_by(rowname)`
Joining with `by = join_by(name)`
Joining with `by = join_by(name)`
Joining with `by = join_by(name)`
Joining with `by = join_by(name)`
Joining with `by = join_by(name)`
Joining with `by = join_by(name)`
Joining with `by = join_by(name)`
Joining with `by = join_by(name)`
Joining with `by = join_by(name)`
Joining with `by = join_by(name)`
found results columns, replacing these
1291 rows did not converge in beta, labelled in mcols(object)$betaConv. Use
larger maxit argument with nbinomWaldTest
using 'apeglm' for LFC shrinkage. If used in published research, please cite:
Zhu, A., Ibrahim, J.G., Love, M.I. (2018) Heavy-tailed prior distributions for
sequence count data: removing the noise and preserving large differences.
Bioinformatics. https://doi.org/10.1093/bioinformatics/bty895
some rows did not converge in finding the MAP
using 'apeglm' for LFC shrinkage. If used in published research, please cite:
Zhu, A., Ibrahim, J.G., Love, M.I. (2018) Heavy-tailed prior distributions for
sequence count data: removing the noise and preserving large differences.
Bioinformatics. https://doi.org/10.1093/bioinformatics/bty895
some rows did not converge in finding the MAP
```

```
using 'apeglm' for LFC shrinkage. If used in published research, please cite:
Zhu, A., Ibrahim, J.G., Love, M.I. (2018) Heavy-tailed prior distributions for
sequence count data: removing the noise and preserving large differences.
Bioinformatics. https://doi.org/10.1093/bioinformatics/bty895
using 'apeglm' for LFC shrinkage. If used in published research, please cite:
Zhu, A., Ibrahim, J.G., Love, M.I. (2018) Heavy-tailed prior distributions for
sequence count data: removing the noise and preserving large differences.
Bioinformatics. https://doi.org/10.1093/bioinformatics/bty895
using 'apeglm' for LFC shrinkage. If used in published research, please cite:
Zhu, A., Ibrahim, J.G., Love, M.I. (2018) Heavy-tailed prior distributions for
sequence count data: removing the noise and preserving large differences.
Bioinformatics. https://doi.org/10.1093/bioinformatics/bty895
Joining with `by = join_by(rowname)`
Joining with `by = join_by(rowname)`
Joining with `by = join_by(rowname)`
Joining with `by = join_by(rowname)`
Joining with `by = join_by(rowname)`
Joining with `by = join_by(name)`
Joining with `by = join_by(name)`
Joining with `by = join_by(name)`
Joining with `by = join_by(name)`
Joining with `by = join_by(name)`
Joining with `by = join_by(name)`
Joining with `by = join_by(name)`
Joining with `by = join_by(name)`
Joining with `by = join_by(name)`
Joining with `by = join_by(name)`
found results columns, replacing these
1185 rows did not converge in beta, labelled in mcols(object)$betaConv. Use
larger maxit argument with nbinomWaldTest
using 'apeglm' for LFC shrinkage. If used in published research, please cite:
Zhu, A., Ibrahim, J.G., Love, M.I. (2018) Heavy-tailed prior distributions for
sequence count data: removing the noise and preserving large differences.
Bioinformatics. https://doi.org/10.1093/bioinformatics/bty895
some rows did not converge in finding the MAP
using 'apeglm' for LFC shrinkage. If used in published research, please cite:
Zhu, A., Ibrahim, J.G., Love, M.I. (2018) Heavy-tailed prior distributions for
sequence count data: removing the noise and preserving large differences.
Bioinformatics. https://doi.org/10.1093/bioinformatics/bty895
using 'apeglm' for LFC shrinkage. If used in published research, please cite:
Zhu, A., Ibrahim, J.G., Love, M.I. (2018) Heavy-tailed prior distributions for
sequence count data: removing the noise and preserving large differences.
Bioinformatics. https://doi.org/10.1093/bioinformatics/bty895
```

```
using 'apeglm' for LFC shrinkage. If used in published research, please cite:
Zhu, A., Ibrahim, J.G., Love, M.I. (2018) Heavy-tailed prior distributions for
sequence count data: removing the noise and preserving large differences.
Bioinformatics. https://doi.org/10.1093/bioinformatics/bty895
using 'apeglm' for LFC shrinkage. If used in published research, please cite:
Zhu, A., Ibrahim, J.G., Love, M.I. (2018) Heavy-tailed prior distributions for
sequence count data: removing the noise and preserving large differences.
Bioinformatics. https://doi.org/10.1093/bioinformatics/bty895
Joining with `by = join_by(rowname)`
Joining with `by = join_by(rowname)`
Joining with `by = join_by(rowname)`
Joining with `by = join_by(rowname)`
Joining with `by = join_by(rowname)`
Joining with `by = join_by(name)`
Joining with `by = join_by(name)`
Joining with `by = join_by(name)`
Joining with `by = join_by(name)`
Joining with `by = join_by(name)`
Joining with `by = join_by(name)`
Joining with `by = join_by(name)`
Joining with `by = join_by(name)`
Joining with `by = join_by(name)`
Joining with `by = join_by(name)`
found results columns, replacing these
1201 rows did not converge in beta, labelled in mcols(object)$betaConv. Use
larger maxit argument with nbinomWaldTest
using 'apeglm' for LFC shrinkage. If used in published research, please cite:
Zhu, A., Ibrahim, J.G., Love, M.I. (2018) Heavy-tailed prior distributions for
sequence count data: removing the noise and preserving large differences.
Bioinformatics. https://doi.org/10.1093/bioinformatics/bty895
using 'apeglm' for LFC shrinkage. If used in published research, please cite:
Zhu, A., Ibrahim, J.G., Love, M.I. (2018) Heavy-tailed prior distributions for
sequence count data: removing the noise and preserving large differences.
Bioinformatics. https://doi.org/10.1093/bioinformatics/bty895
using 'apeglm' for LFC shrinkage. If used in published research, please cite:
Zhu, A., Ibrahim, J.G., Love, M.I. (2018) Heavy-tailed prior distributions for
sequence count data: removing the noise and preserving large differences.
Bioinformatics. https://doi.org/10.1093/bioinformatics/bty895
using 'apeglm' for LFC shrinkage. If used in published research, please cite:
Zhu, A., Ibrahim, J.G., Love, M.I. (2018) Heavy-tailed prior distributions for
sequence count data: removing the noise and preserving large differences.
Bioinformatics. https://doi.org/10.1093/bioinformatics/bty895
using 'apeglm' for LFC shrinkage. If used in published research, please cite:
```

```
Zhu, A., Ibrahim, J.G., Love, M.I. (2018) Heavy-tailed prior distributions for
sequence count data: removing the noise and preserving large differences.
Bioinformatics. https://doi.org/10.1093/bioinformatics/bty895
Joining with `by = join_by(rowname)`
Joining with `by = join_by(rowname)`
Joining with `by = join_by(rowname)`
Joining with `by = join_by(rowname)`
Joining with `by = join_by(rowname)`
Joining with `by = join_by(name)`
Joining with `by = join_by(name)`
Joining with `by = join_by(name)`
Joining with `by = join_by(name)`
Joining with `by = join_by(name)`
Joining with `by = join_by(name)`
Joining with `by = join_by(name)`
Joining with `by = join_by(name)`
Joining with `by = join_by(name)`
Joining with `by = join_by(name)`
found results columns, replacing these
1224 rows did not converge in beta, labelled in mcols(object)$betaConv. Use
larger maxit argument with nbinomWaldTest
using 'apeglm' for LFC shrinkage. If used in published research, please cite:
Zhu, A., Ibrahim, J.G., Love, M.I. (2018) Heavy-tailed prior distributions for
sequence count data: removing the noise and preserving large differences.
Bioinformatics. https://doi.org/10.1093/bioinformatics/bty895
using 'apeglm' for LFC shrinkage. If used in published research, please cite:
Zhu, A., Ibrahim, J.G., Love, M.I. (2018) Heavy-tailed prior distributions for
sequence count data: removing the noise and preserving large differences.
Bioinformatics. https://doi.org/10.1093/bioinformatics/bty895
using 'apeglm' for LFC shrinkage. If used in published research, please cite:
Zhu, A., Ibrahim, J.G., Love, M.I. (2018) Heavy-tailed prior distributions for
sequence count data: removing the noise and preserving large differences.
Bioinformatics. https://doi.org/10.1093/bioinformatics/bty895
using 'apeglm' for LFC shrinkage. If used in published research, please cite:
Zhu, A., Ibrahim, J.G., Love, M.I. (2018) Heavy-tailed prior distributions for
sequence count data: removing the noise and preserving large differences.
Bioinformatics. https://doi.org/10.1093/bioinformatics/bty895
using 'apeglm' for LFC shrinkage. If used in published research, please cite:
Zhu, A., Ibrahim, J.G., Love, M.I. (2018) Heavy-tailed prior distributions for
sequence count data: removing the noise and preserving large differences.
Bioinformatics. https://doi.org/10.1093/bioinformatics/bty895
Joining with `by = join_by(rowname)`
Joining with `by = join_by(rowname)`
```

```
Joining with `by = join_by(rowname)`
Joining with `by = join_by(rowname)`
Joining with `by = join_by(rowname)`
Joining with `by = join_by(name)`
Joining with `by = join_by(name)`
Joining with `by = join_by(name)`
Joining with `by = join_by(name)`
Joining with `by = join_by(name)`
Joining with `by = join_by(name)`
Joining with `by = join_by(name)`
Joining with `by = join_by(name)`
Joining with `by = join_by(name)`
Joining with `by = join_by(name)`
found results columns, replacing these
1204 rows did not converge in beta, labelled in mcols(object)$betaConv. Use
larger maxit argument with nbinomWaldTest
using 'apeglm' for LFC shrinkage. If used in published research, please cite:
Zhu, A., Ibrahim, J.G., Love, M.I. (2018) Heavy-tailed prior distributions for
sequence count data: removing the noise and preserving large differences.
Bioinformatics. https://doi.org/10.1093/bioinformatics/bty895
some rows did not converge in finding the MAP
using 'apeglm' for LFC shrinkage. If used in published research, please cite:
Zhu, A., Ibrahim, J.G., Love, M.I. (2018) Heavy-tailed prior distributions for
sequence count data: removing the noise and preserving large differences.
Bioinformatics. https://doi.org/10.1093/bioinformatics/bty895
using 'apeglm' for LFC shrinkage. If used in published research, please cite:
Zhu, A., Ibrahim, J.G., Love, M.I. (2018) Heavy-tailed prior distributions for
sequence count data: removing the noise and preserving large differences.
Bioinformatics. https://doi.org/10.1093/bioinformatics/bty895
some rows did not converge in finding the MAP
using 'apeglm' for LFC shrinkage. If used in published research, please cite:
Zhu, A., Ibrahim, J.G., Love, M.I. (2018) Heavy-tailed prior distributions for
sequence count data: removing the noise and preserving large differences.
Bioinformatics. https://doi.org/10.1093/bioinformatics/bty895
using 'apeglm' for LFC shrinkage. If used in published research, please cite:
Zhu, A., Ibrahim, J.G., Love, M.I. (2018) Heavy-tailed prior distributions for
sequence count data: removing the noise and preserving large differences.
Bioinformatics. https://doi.org/10.1093/bioinformatics/bty895
Joining with `by = join_by(rowname)`
Joining with `by = join_by(rowname)`
Joining with `by = join_by(rowname)`
Joining with `by = join_by(rowname)`
Joining with `by = join_by(rowname)`
```

```
Joining with `by = join_by(name)`
Joining with `by = join_by(name)`
Joining with `by = join_by(name)`
Joining with `by = join_by(name)`
Joining with `by = join_by(name)`
Joining with `by = join_by(name)`
Joining with `by = join_by(name)`
Joining with `by = join_by(name)`
Joining with `by = join_by(name)`
Joining with `by = join_by(name)`
```

```
group <- levels(as.factor(dds.sva$group))
sva_set <- compare_reciprocal_contrasts(group,folder)
```

```
[1] "Found no regulated genes for  OHT_48h  and  DPN_48h"

[1] "No overlap of regulated genes using different reference level for OHT_24h and DPN_24h"

[1] "No overlap of regulated genes using different reference level for OHT_24h and Control_2[illegible]

[1] "No overlap of regulated genes using different reference level for DPN_48h and Control_4[illegible]

[1] "Found no regulated genes for  DPN_24h  and  Control_24h"
```

```
saveRDS(sva_set, "sva_set.rds")
```

### Summary figure

We can open the folders and see the diagnostic plots for each analysis above. However, the information can also be graphed from the results sets returned by `compare_reciprocal_contrasts()`. For visualisation and to make the important changes more obvious, we remove contrasts that totaled <4 DEGs across all the results sets.

```
sets <- list("noclean" = noclean_set,"prefilt" = prefilt_set, "sva" = sva_set)
sets <- lapply(sets,
               function(x) lapply(x,
                                   function(y) y %>% dplyr::select(partition)))
sets <- lapply(sets,function(x) x %>% bind_rows(.id = "contrast"))
all.sets <- bind_rows(sets, .id = "group")
```

```r
  totals <- all.sets %>%
  group_by(group,contrast) %>%
  summarise(total.per.contrast = n()) %>%
  ungroup()
```

```
`summarise()` has grouped output by 'group'. You can override using the
`.groups` argument.
```

```r
head(totals)
```

```
# A tibble: 6 x 3
  group   contrast                total.per.contrast
  <chr>   <chr>                                <int>
1 noclean Control_48h.Control_24h                172
2 noclean DPN_24h.Control_48h                    337
3 noclean DPN_48h.Control_24h                    109
4 noclean DPN_48h.Control_48h                      1
5 noclean DPN_48h.DPN_24h                        175
6 noclean OHT_24h.Control_48h                    291
```

```r
  plottable <- all.sets %>%
  group_by(group,contrast) %>%
  count(partition) %>%
  pivot_wider(values_from = n, names_from = partition, values_fill = 0) %>%
  pivot_longer(c(concordant,group1ref,group2ref),names_to = "partition",
               values_to = "n") %>%
  left_join(totals) %>%
  filter(partition == "concordant") %>%
  dplyr::select(-partition) %>%
  rowwise() %>%
  mutate(discordance = 100 - (n/total.per.contrast*100)) %>%
  ungroup() %>%
  mutate(across(c(group, contrast), as_factor))
```

```
Joining with `by = join_by(group, contrast)`
```

```r
#for ease of visualisation and understanding, we remove the contrasts with < 4
#total DEGs
plottable <- plottable %>%
  filter(total.per.contrast > 3)
head(plottable)
```

```
# A tibble: 6 x 5
  group   contrast                     n total.per.contrast discordance
  <fct>   <fct>                    <int>              <int>       <dbl>
1 noclean Control_48h.Control_24h    161                172        6.40
2 noclean DPN_24h.Control_48h        327                337        2.97
3 noclean DPN_48h.Control_24h        100                109        8.26
4 noclean DPN_48h.DPN_24h            171                175        2.29
5 noclean OHT_24h.Control_48h        283                291        2.75
6 noclean OHT_24h.DPN_48h            162                171        5.26
```

```
colours <- c(sva = '#EE7733', prefilt = '#0077BB', noclean = '#BBBBBB')
discord <- ggplot(plottable, aes(x=group, y=discordance, fill = group)) +
  geom_violin(scale = "count") +
  geom_point(size = 2, show.legend = F) +
  scale_fill_manual(values = colours) +
  geom_line(aes(group = contrast)) +
  labs(y = "% discordance\nin total DEGs per contrast") +
  theme_classic(base_size = 20) +
  theme(axis.ticks.x = element_blank(),
        axis.title.x = element_blank(),
        axis.text.x = element_blank(),
        legend.title = element_blank()) +
  guides(color = "none")
  summed.concordant <- plottable %>%
  summarise(total = sum(n), .by = c(group))
summed.concordant
```

```
# A tibble: 3 x 2
  group   total
  <fct>   <int>
1 noclean  1709
2 prefilt  1772
3 sva       528
```

```
summed <- ggplot(summed.concordant,aes(y = total, x = group, fill = group)) +
  scale_fill_manual(values = colours) +
  geom_col() +
  expand_limits(y = 2250) +
  geom_text(aes(label=total), vjust=-0.5, size = 5) +
  labs(y = "total\nconcordant\nDEGs") +
  theme_classic(base_size = 20) +
  theme(legend.position = "none",
```

```
        axis.ticks.x = element_blank(),
        axis.title.x = element_blank(),
        axis.text.x = element_blank(),
        strip.background = element_blank(),
        strip.text.x = element_blank())
#for change in concordant DEGs per contrast
changes <- plottable %>%
  dplyr::select(contrast,n,group) %>%
  pivot_wider(names_from = group,values_from = n) %>% #, values_fill = 0) %>%
  rowwise() %>%
  mutate(change_prefilt = (prefilt-noclean)/noclean*100,
         direction_prefilt = if_else(change_prefilt > 0, "UP","DOWN"),
         change_sva = (sva-noclean)/noclean*100,
         direction_sva =  if_else(change_sva > 0, "UP","DOWN")) %>%
  dplyr::select(-c(prefilt,sva,noclean)) %>%
  pivot_longer(!contrast, names_to = c(".value","cleanup"), names_sep = "_")
  diff <- ggplot(changes,aes(y = change,
                           x = factor(cleanup,
                                      levels = c("noclean","prefilt","sva")),
                           shape = direction, fill = cleanup)) +
  geom_point(size = 3, stroke = 0.8) +
  scale_shape_manual(values = c("UP" = 24,"DOWN" = 25)) +
  scale_fill_manual(values = colours) +
  scale_x_discrete("cleanup", drop=FALSE) +
  theme_classic(base_size = 20) +
  theme(legend.position = "none",
        axis.ticks.x = element_blank(),
        axis.title.x = element_blank(),
        axis.text.x = element_blank()) +
  ylab("% change in\nconcordant DEGs after\nclean-up per contrast") +
  geom_hline(yintercept = 0, linetype = "dashed") +
  geom_line(aes(group = contrast))

figure <- ggarrange(summed,diff,discord,
                    ncol = 1, nrow = 3, heights = c(1,1.5,2), align = "v")
figure
```

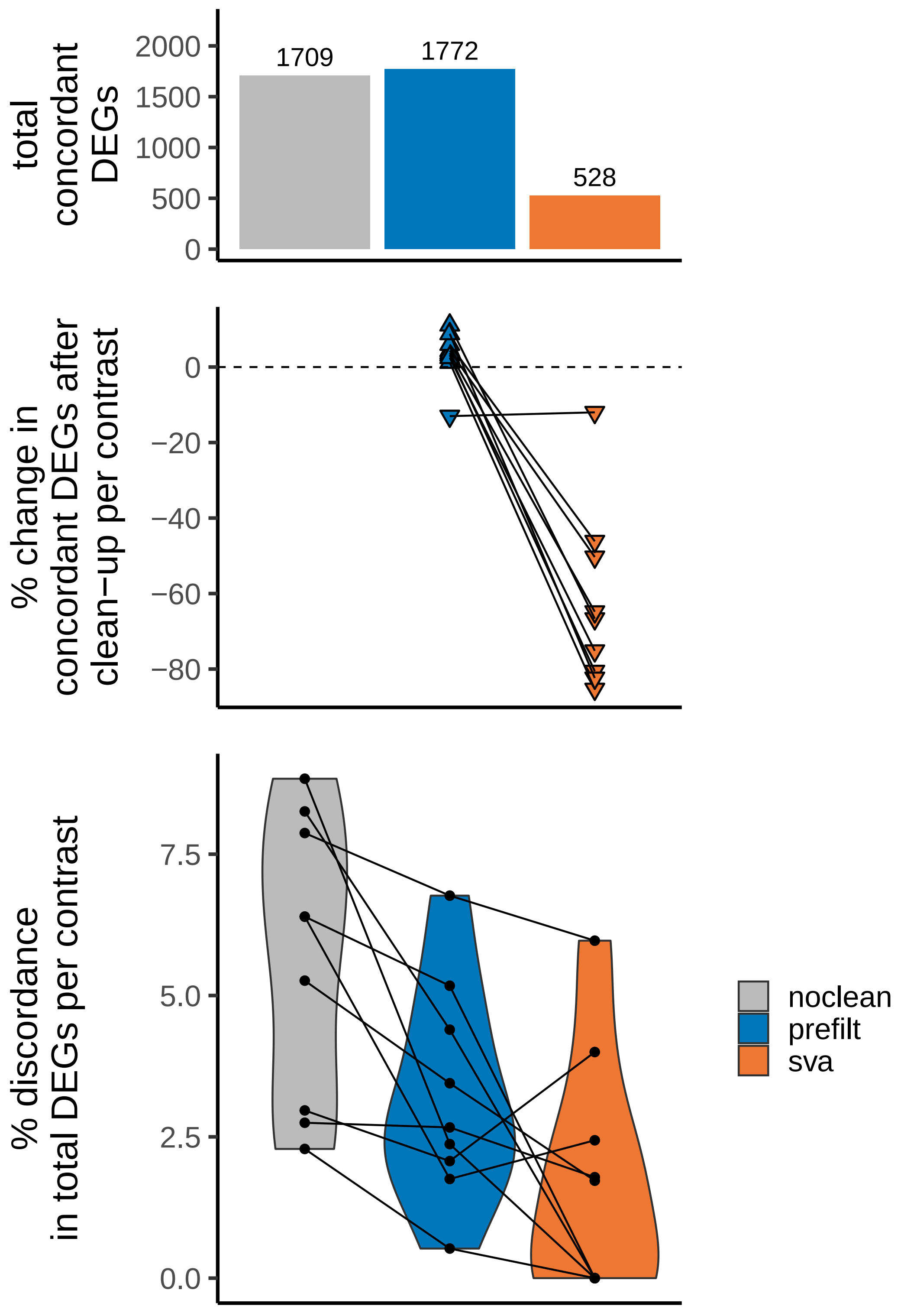
total
concordant
DEGs
2000
1500
1000
500
0
1709
1772
528
% change in
concordant DEGs after
clean−up per contrast
0
−20
−40
−60
−80
% discordance
in total DEGs per contrast
7.5
5.0
2.5
0.0
noclean
prefilt
sva

We can see that the discordance is not bad even without prefiltering, but prefiltering may improve the results - it reduced discordance even further in a number of contrasts and increased number of DEGs, both in total and in most individual contrasts. Using SVA, the discordance again improves slightly, but the number of consensus DEGs is dramatically reduced.

### What if we hadn't accounted for 'patient'?

How bad would the analysis be if we hadn't known about the patient factor? We reset the design as `design=~group` and rerun mirrorCheck to find out.

```
folder <- "DESeq_no_patient"
dir.create(folder)
design(dds) <- ~group
run_DESeq_all_contrasts(dds,folder,
                        condition = "group",
                        mode="apeglm",p.cutoff = 0.05,
                        rowname2symbol = rowname2symbol,
                        top.n = 30,useDingbats = T,
                        fc.cutoff = 1,
                        print.all = T)
```

```
using pre-existing size factors

estimating dispersions

found already estimated dispersions, replacing these

gene-wise dispersion estimates

mean-dispersion relationship

final dispersion estimates

fitting model and testing
```

```
using 'apeglm' for LFC shrinkage. If used in published research, please cite:
    Zhu, A., Ibrahim, J.G., Love, M.I. (2018) Heavy-tailed prior distributions for
    sequence count data: removing the noise and preserving large differences.
    Bioinformatics. https://doi.org/10.1093/bioinformatics/bty895
using 'apeglm' for LFC shrinkage. If used in published research, please cite:
    Zhu, A., Ibrahim, J.G., Love, M.I. (2018) Heavy-tailed prior distributions for
    sequence count data: removing the noise and preserving large differences.
    Bioinformatics. https://doi.org/10.1093/bioinformatics/bty895
using 'apeglm' for LFC shrinkage. If used in published research, please cite:
    Zhu, A., Ibrahim, J.G., Love, M.I. (2018) Heavy-tailed prior distributions for
    sequence count data: removing the noise and preserving large differences.
    Bioinformatics. https://doi.org/10.1093/bioinformatics/bty895
using 'apeglm' for LFC shrinkage. If used in published research, please cite:
    Zhu, A., Ibrahim, J.G., Love, M.I. (2018) Heavy-tailed prior distributions for
    sequence count data: removing the noise and preserving large differences.
    Bioinformatics. https://doi.org/10.1093/bioinformatics/bty895
using 'apeglm' for LFC shrinkage. If used in published research, please cite:
    Zhu, A., Ibrahim, J.G., Love, M.I. (2018) Heavy-tailed prior distributions for
    sequence count data: removing the noise and preserving large differences.
    Bioinformatics. https://doi.org/10.1093/bioinformatics/bty895

Joining with `by = join_by(rowname)`
Joining with `by = join_by(rowname)`
Joining with `by = join_by(rowname)`
Joining with `by = join_by(rowname)`
Joining with `by = join_by(rowname)`
Joining with `by = join_by(name)`
Joining with `by = join_by(name)`
Joining with `by = join_by(name)`
Joining with `by = join_by(name)`
Joining with `by = join_by(name)`
Joining with `by = join_by(name)`
Joining with `by = join_by(name)`
Joining with `by = join_by(name)`
Joining with `by = join_by(name)`
Joining with `by = join_by(name)`
found results columns, replacing these
using 'apeglm' for LFC shrinkage. If used in published research, please cite:
Zhu, A., Ibrahim, J.G., Love, M.I. (2018) Heavy-tailed prior distributions for
sequence count data: removing the noise and preserving large differences.
Bioinformatics. https://doi.org/10.1093/bioinformatics/bty895
using 'apeglm' for LFC shrinkage. If used in published research, please cite:
```

```
Zhu, A., Ibrahim, J.G., Love, M.I. (2018) Heavy-tailed prior distributions for
sequence count data: removing the noise and preserving large differences.
Bioinformatics. https://doi.org/10.1093/bioinformatics/bty895
using 'apeglm' for LFC shrinkage. If used in published research, please cite:
Zhu, A., Ibrahim, J.G., Love, M.I. (2018) Heavy-tailed prior distributions for
sequence count data: removing the noise and preserving large differences.
Bioinformatics. https://doi.org/10.1093/bioinformatics/bty895
using 'apeglm' for LFC shrinkage. If used in published research, please cite:
Zhu, A., Ibrahim, J.G., Love, M.I. (2018) Heavy-tailed prior distributions for
sequence count data: removing the noise and preserving large differences.
Bioinformatics. https://doi.org/10.1093/bioinformatics/bty895
using 'apeglm' for LFC shrinkage. If used in published research, please cite:
Zhu, A., Ibrahim, J.G., Love, M.I. (2018) Heavy-tailed prior distributions for
sequence count data: removing the noise and preserving large differences.
Bioinformatics. https://doi.org/10.1093/bioinformatics/bty895

Warning in nbinomGLM(x = x, Y = YNZ, size = size, weights = weightsNZ, offset =
offsetNZ, : the line search routine failed, unable to sufficiently decrease the
function value

Joining with `by = join_by(rowname)`
Joining with `by = join_by(rowname)`
Joining with `by = join_by(rowname)`
Joining with `by = join_by(rowname)`
Joining with `by = join_by(rowname)`
Joining with `by = join_by(name)`
Joining with `by = join_by(name)`
Joining with `by = join_by(name)`
Joining with `by = join_by(name)`
Joining with `by = join_by(name)`
Joining with `by = join_by(name)`
Joining with `by = join_by(name)`
Joining with `by = join_by(name)`
Joining with `by = join_by(name)`
Joining with `by = join_by(name)`
found results columns, replacing these
using 'apeglm' for LFC shrinkage. If used in published research, please cite:
Zhu, A., Ibrahim, J.G., Love, M.I. (2018) Heavy-tailed prior distributions for
sequence count data: removing the noise and preserving large differences.
Bioinformatics. https://doi.org/10.1093/bioinformatics/bty895
using 'apeglm' for LFC shrinkage. If used in published research, please cite:
Zhu, A., Ibrahim, J.G., Love, M.I. (2018) Heavy-tailed prior distributions for
```

```
sequence count data: removing the noise and preserving large differences.
Bioinformatics. https://doi.org/10.1093/bioinformatics/bty895
using 'apeglm' for LFC shrinkage. If used in published research, please cite:
Zhu, A., Ibrahim, J.G., Love, M.I. (2018) Heavy-tailed prior distributions for
sequence count data: removing the noise and preserving large differences.
Bioinformatics. https://doi.org/10.1093/bioinformatics/bty895
using 'apeglm' for LFC shrinkage. If used in published research, please cite:
Zhu, A., Ibrahim, J.G., Love, M.I. (2018) Heavy-tailed prior distributions for
sequence count data: removing the noise and preserving large differences.
Bioinformatics. https://doi.org/10.1093/bioinformatics/bty895

Warning in nbinomGLM(x = x, Y = YNZ, size = size, weights = weightsNZ, offset =
offsetNZ, : the line search routine failed, unable to sufficiently decrease the
function value

using 'apeglm' for LFC shrinkage. If used in published research, please cite:
    Zhu, A., Ibrahim, J.G., Love, M.I. (2018) Heavy-tailed prior distributions for
    sequence count data: removing the noise and preserving large differences.
    Bioinformatics. https://doi.org/10.1093/bioinformatics/bty895
Joining with `by = join_by(rowname)`Joining with `by = join_by(rowname)`Joining with `by = jo
using 'apeglm' for LFC shrinkage. If used in published research, please cite:
    Zhu, A., Ibrahim, J.G., Love, M.I. (2018) Heavy-tailed prior distributions for
    sequence count data: removing the noise and preserving large differences.
    Bioinformatics. https://doi.org/10.1093/bioinformatics/bty895
using 'apeglm' for LFC shrinkage. If used in published research, please cite:
    Zhu, A., Ibrahim, J.G., Love, M.I. (2018) Heavy-tailed prior distributions for
    sequence count data: removing the noise and preserving large differences.
    Bioinformatics. https://doi.org/10.1093/bioinformatics/bty895

Warning in nbinomGLM(x = x, Y = YNZ, size = size, weights = weightsNZ, offset =
offsetNZ, : the line search routine failed, unable to sufficiently decrease the
function value

using 'apeglm' for LFC shrinkage. If used in published research, please cite:
    Zhu, A., Ibrahim, J.G., Love, M.I. (2018) Heavy-tailed prior distributions for
    sequence count data: removing the noise and preserving large differences.
    Bioinformatics. https://doi.org/10.1093/bioinformatics/bty895
using 'apeglm' for LFC shrinkage. If used in published research, please cite:
    Zhu, A., Ibrahim, J.G., Love, M.I. (2018) Heavy-tailed prior distributions for
    sequence count data: removing the noise and preserving large differences.
    Bioinformatics. https://doi.org/10.1093/bioinformatics/bty895
```

```
using 'apeglm' for LFC shrinkage. If used in published research, please cite:
    Zhu, A., Ibrahim, J.G., Love, M.I. (2018) Heavy-tailed prior distributions for
    sequence count data: removing the noise and preserving large differences.
    Bioinformatics. https://doi.org/10.1093/bioinformatics/bty895
Joining with `by = join_by(rowname)`Joining with `by = join_by(rowname)`Joining with `by = jo
using 'apeglm' for LFC shrinkage. If used in published research, please cite:
    Zhu, A., Ibrahim, J.G., Love, M.I. (2018) Heavy-tailed prior distributions for
    sequence count data: removing the noise and preserving large differences.
    Bioinformatics. https://doi.org/10.1093/bioinformatics/bty895
using 'apeglm' for LFC shrinkage. If used in published research, please cite:
    Zhu, A., Ibrahim, J.G., Love, M.I. (2018) Heavy-tailed prior distributions for
    sequence count data: removing the noise and preserving large differences.
    Bioinformatics. https://doi.org/10.1093/bioinformatics/bty895
using 'apeglm' for LFC shrinkage. If used in published research, please cite:
    Zhu, A., Ibrahim, J.G., Love, M.I. (2018) Heavy-tailed prior distributions for
    sequence count data: removing the noise and preserving large differences.
    Bioinformatics. https://doi.org/10.1093/bioinformatics/bty895
using 'apeglm' for LFC shrinkage. If used in published research, please cite:
    Zhu, A., Ibrahim, J.G., Love, M.I. (2018) Heavy-tailed prior distributions for
    sequence count data: removing the noise and preserving large differences.
    Bioinformatics. https://doi.org/10.1093/bioinformatics/bty895
using 'apeglm' for LFC shrinkage. If used in published research, please cite:
    Zhu, A., Ibrahim, J.G., Love, M.I. (2018) Heavy-tailed prior distributions for
    sequence count data: removing the noise and preserving large differences.
    Bioinformatics. https://doi.org/10.1093/bioinformatics/bty895
Joining with `by = join_by(rowname)`Joining with `by = join_by(rowname)`Joining with `by = jo
using 'apeglm' for LFC shrinkage. If used in published research, please cite:
    Zhu, A., Ibrahim, J.G., Love, M.I. (2018) Heavy-tailed prior distributions for
    sequence count data: removing the noise and preserving large differences.
    Bioinformatics. https://doi.org/10.1093/bioinformatics/bty895
using 'apeglm' for LFC shrinkage. If used in published research, please cite:
    Zhu, A., Ibrahim, J.G., Love, M.I. (2018) Heavy-tailed prior distributions for
    sequence count data: removing the noise and preserving large differences.
    Bioinformatics. https://doi.org/10.1093/bioinformatics/bty895
using 'apeglm' for LFC shrinkage. If used in published research, please cite:
    Zhu, A., Ibrahim, J.G., Love, M.I. (2018) Heavy-tailed prior distributions for
    sequence count data: removing the noise and preserving large differences.
    Bioinformatics. https://doi.org/10.1093/bioinformatics/bty895
using 'apeglm' for LFC shrinkage. If used in published research, please cite:
    Zhu, A., Ibrahim, J.G., Love, M.I. (2018) Heavy-tailed prior distributions for
    sequence count data: removing the noise and preserving large differences.
    Bioinformatics. https://doi.org/10.1093/bioinformatics/bty895
using 'apeglm' for LFC shrinkage. If used in published research, please cite:
```

```
    Zhu, A., Ibrahim, J.G., Love, M.I. (2018) Heavy-tailed prior distributions for
    sequence count data: removing the noise and preserving large differences.
    Bioinformatics. https://doi.org/10.1093/bioinformatics/bty895
Joining with `by = join_by(rowname)`Joining with `by = join_by(rowname)`Joining with `by = jo
```

```
group <- levels(as.factor(dds$group))
no_patient_set <- compare_reciprocal_contrasts(group,folder)
```

```
[1] "Found no regulated genes for  OHT_48h  and  DPN_48h"

[1] "Found no regulated genes for  OHT_48h  and  Control_48h"

[1] "Found no regulated genes for  OHT_24h  and  DPN_24h"

[1] "Found no regulated genes for  OHT_24h  and  Control_24h"

[1] "Found no regulated genes for  DPN_48h  and  Control_48h"

[1] "Found no regulated genes for  DPN_24h  and  Control_24h"

Warning: Groups with fewer than two data points have been dropped.

Warning in max(ids, na.rm = TRUE): no non-missing arguments to max; returning
-Inf
```

```
saveRDS(no_patient_set, "no_patient_set.rds")
```

So, from the diagnostic plots, it's pretty bad - we don't identify many DEGs and also there is poor consensus in reciprocal contrasts.

#### Showing the value of sva - identifying the 'patient' factor even if we didn't have that information

It was pretty obvious from the PCA that there's strong clustering by a variable that isn't treatment or time. Let's see if we can account for the unwanted variable using sva. (This approach copies how Love et al. (2016) illustrate the use of sva.)

```
folder <- "DESeq_no_patient_sva"
dir.create(folder)

dds <- DESeq(dds)
```

```
using pre-existing size factors

estimating dispersions

found already estimated dispersions, replacing these

gene-wise dispersion estimates

mean-dispersion relationship

final dispersion estimates

fitting model and testing
```

```
dat <- counts(dds, normalized=TRUE)
idx <- rowMeans(dat) > 1
dat <- dat[idx,]
mod <- model.matrix(~ group, colData(dds))
mod0 <- model.matrix(~ 1, colData(dds))

#find probable number of unwanted variables
num_sv <- num.sv(dat,mod,method = "be")
num_sv
```

```
[1] 1
```

```
svseq <- svaseq(dat, mod, mod0, n.sv=num_sv)
```

```
Number of significant surrogate variables is:  1
Iteration (out of 5 ):1  2  3  4  5
```

```r
dds.sva <- dds
dds.sva$SV1 <- svseq$sv[,1]
design(dds.sva) <- ~ SV1 + group

stripchart(svseq$sv[,1] ~ dds$patient,vertical=TRUE,main="SV1")
abline(h=0)
```

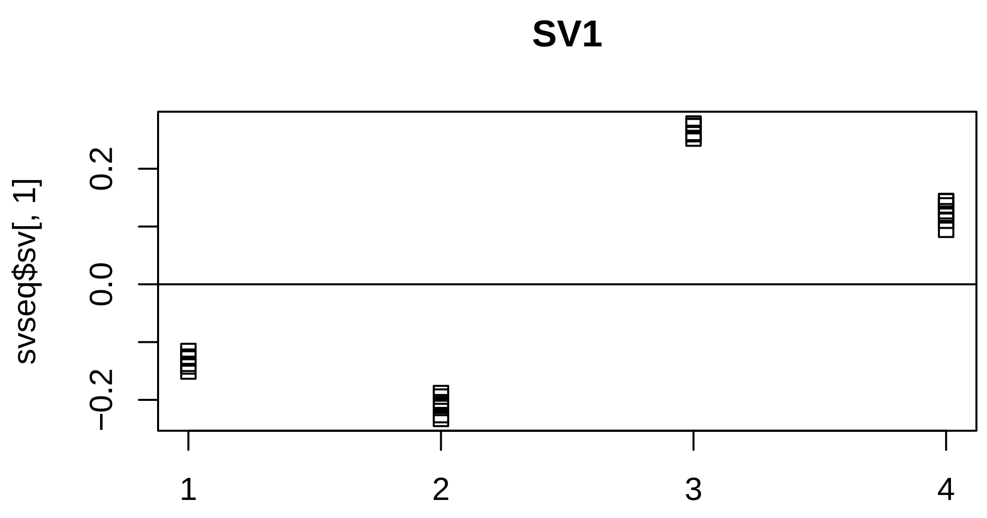


```r
run_DESeq_all_contrasts(dds.sva,folder,
                        condition = "group",
                        mode="apeglm",p.cutoff = 0.05,
                        rowname2symbol = rowname2symbol,
                        top.n = 30,useDingbats = T,
                        fc.cutoff = 1,
                        print.all = T)
```

```
using pre-existing size factors
```

```
estimating dispersions
```

```
found already estimated dispersions, replacing these
```

```
gene-wise dispersion estimates

mean-dispersion relationship

final dispersion estimates

fitting model and testing

using 'apeglm' for LFC shrinkage. If used in published research, please cite:
    Zhu, A., Ibrahim, J.G., Love, M.I. (2018) Heavy-tailed prior distributions for
    sequence count data: removing the noise and preserving large differences.
    Bioinformatics. https://doi.org/10.1093/bioinformatics/bty895
using 'apeglm' for LFC shrinkage. If used in published research, please cite:
    Zhu, A., Ibrahim, J.G., Love, M.I. (2018) Heavy-tailed prior distributions for
    sequence count data: removing the noise and preserving large differences.
    Bioinformatics. https://doi.org/10.1093/bioinformatics/bty895
using 'apeglm' for LFC shrinkage. If used in published research, please cite:
    Zhu, A., Ibrahim, J.G., Love, M.I. (2018) Heavy-tailed prior distributions for
    sequence count data: removing the noise and preserving large differences.
    Bioinformatics. https://doi.org/10.1093/bioinformatics/bty895
using 'apeglm' for LFC shrinkage. If used in published research, please cite:
    Zhu, A., Ibrahim, J.G., Love, M.I. (2018) Heavy-tailed prior distributions for
    sequence count data: removing the noise and preserving large differences.
    Bioinformatics. https://doi.org/10.1093/bioinformatics/bty895
using 'apeglm' for LFC shrinkage. If used in published research, please cite:
    Zhu, A., Ibrahim, J.G., Love, M.I. (2018) Heavy-tailed prior distributions for
    sequence count data: removing the noise and preserving large differences.
    Bioinformatics. https://doi.org/10.1093/bioinformatics/bty895

Joining with `by = join_by(rowname)`
Joining with `by = join_by(rowname)`
Joining with `by = join_by(rowname)`
Joining with `by = join_by(rowname)`
Joining with `by = join_by(rowname)`
Joining with `by = join_by(name)`
Joining with `by = join_by(name)`
Joining with `by = join_by(name)`
Joining with `by = join_by(name)`
Joining with `by = join_by(name)`
Joining with `by = join_by(name)`
Joining with `by = join_by(name)`
```

```
Joining with `by = join_by(name)`
Joining with `by = join_by(name)`
Joining with `by = join_by(name)`
found results columns, replacing these
using 'apeglm' for LFC shrinkage. If used in published research, please cite:
Zhu, A., Ibrahim, J.G., Love, M.I. (2018) Heavy-tailed prior distributions for
sequence count data: removing the noise and preserving large differences.
Bioinformatics. https://doi.org/10.1093/bioinformatics/bty895
using 'apeglm' for LFC shrinkage. If used in published research, please cite:
Zhu, A., Ibrahim, J.G., Love, M.I. (2018) Heavy-tailed prior distributions for
sequence count data: removing the noise and preserving large differences.
Bioinformatics. https://doi.org/10.1093/bioinformatics/bty895
using 'apeglm' for LFC shrinkage. If used in published research, please cite:
Zhu, A., Ibrahim, J.G., Love, M.I. (2018) Heavy-tailed prior distributions for
sequence count data: removing the noise and preserving large differences.
Bioinformatics. https://doi.org/10.1093/bioinformatics/bty895
using 'apeglm' for LFC shrinkage. If used in published research, please cite:
Zhu, A., Ibrahim, J.G., Love, M.I. (2018) Heavy-tailed prior distributions for
sequence count data: removing the noise and preserving large differences.
Bioinformatics. https://doi.org/10.1093/bioinformatics/bty895
using 'apeglm' for LFC shrinkage. If used in published research, please cite:
Zhu, A., Ibrahim, J.G., Love, M.I. (2018) Heavy-tailed prior distributions for
sequence count data: removing the noise and preserving large differences.
Bioinformatics. https://doi.org/10.1093/bioinformatics/bty895
Joining with `by = join_by(rowname)`
Joining with `by = join_by(rowname)`
Joining with `by = join_by(rowname)`
Joining with `by = join_by(rowname)`
Joining with `by = join_by(rowname)`
Joining with `by = join_by(name)`
Joining with `by = join_by(name)`
Joining with `by = join_by(name)`
Joining with `by = join_by(name)`
Joining with `by = join_by(name)`
Joining with `by = join_by(name)`
Joining with `by = join_by(name)`
Joining with `by = join_by(name)`
Joining with `by = join_by(name)`
Joining with `by = join_by(name)`
found results columns, replacing these
using 'apeglm' for LFC shrinkage. If used in published research, please cite:
Zhu, A., Ibrahim, J.G., Love, M.I. (2018) Heavy-tailed prior distributions for
sequence count data: removing the noise and preserving large differences.
```

```
Bioinformatics. https://doi.org/10.1093/bioinformatics/bty895
using 'apeglm' for LFC shrinkage. If used in published research, please cite:
Zhu, A., Ibrahim, J.G., Love, M.I. (2018) Heavy-tailed prior distributions for
sequence count data: removing the noise and preserving large differences.
Bioinformatics. https://doi.org/10.1093/bioinformatics/bty895
using 'apeglm' for LFC shrinkage. If used in published research, please cite:
Zhu, A., Ibrahim, J.G., Love, M.I. (2018) Heavy-tailed prior distributions for
sequence count data: removing the noise and preserving large differences.
Bioinformatics. https://doi.org/10.1093/bioinformatics/bty895
using 'apeglm' for LFC shrinkage. If used in published research, please cite:
Zhu, A., Ibrahim, J.G., Love, M.I. (2018) Heavy-tailed prior distributions for
sequence count data: removing the noise and preserving large differences.
Bioinformatics. https://doi.org/10.1093/bioinformatics/bty895
using 'apeglm' for LFC shrinkage. If used in published research, please cite:
Zhu, A., Ibrahim, J.G., Love, M.I. (2018) Heavy-tailed prior distributions for
sequence count data: removing the noise and preserving large differences.
Bioinformatics. https://doi.org/10.1093/bioinformatics/bty895
Joining with `by = join_by(rowname)`
Joining with `by = join_by(rowname)`
Joining with `by = join_by(rowname)`
Joining with `by = join_by(rowname)`
Joining with `by = join_by(rowname)`
Joining with `by = join_by(name)`
Joining with `by = join_by(name)`
Joining with `by = join_by(name)`
Joining with `by = join_by(name)`
Joining with `by = join_by(name)`
Joining with `by = join_by(name)`
Joining with `by = join_by(name)`
Joining with `by = join_by(name)`
Joining with `by = join_by(name)`
Joining with `by = join_by(name)`
found results columns, replacing these
using 'apeglm' for LFC shrinkage. If used in published research, please cite:
Zhu, A., Ibrahim, J.G., Love, M.I. (2018) Heavy-tailed prior distributions for
sequence count data: removing the noise and preserving large differences.
Bioinformatics. https://doi.org/10.1093/bioinformatics/bty895
using 'apeglm' for LFC shrinkage. If used in published research, please cite:
Zhu, A., Ibrahim, J.G., Love, M.I. (2018) Heavy-tailed prior distributions for
sequence count data: removing the noise and preserving large differences.
Bioinformatics. https://doi.org/10.1093/bioinformatics/bty895
using 'apeglm' for LFC shrinkage. If used in published research, please cite:
Zhu, A., Ibrahim, J.G., Love, M.I. (2018) Heavy-tailed prior distributions for
```

```
sequence count data: removing the noise and preserving large differences.
Bioinformatics. https://doi.org/10.1093/bioinformatics/bty895
using 'apeglm' for LFC shrinkage. If used in published research, please cite:
Zhu, A., Ibrahim, J.G., Love, M.I. (2018) Heavy-tailed prior distributions for
sequence count data: removing the noise and preserving large differences.
Bioinformatics. https://doi.org/10.1093/bioinformatics/bty895
using 'apeglm' for LFC shrinkage. If used in published research, please cite:
Zhu, A., Ibrahim, J.G., Love, M.I. (2018) Heavy-tailed prior distributions for
sequence count data: removing the noise and preserving large differences.
Bioinformatics. https://doi.org/10.1093/bioinformatics/bty895
Joining with `by = join_by(rowname)`
Joining with `by = join_by(rowname)`
Joining with `by = join_by(rowname)`
Joining with `by = join_by(rowname)`
Joining with `by = join_by(rowname)`
Joining with `by = join_by(name)`
Joining with `by = join_by(name)`
Joining with `by = join_by(name)`
Joining with `by = join_by(name)`
Joining with `by = join_by(name)`
Joining with `by = join_by(name)`
Joining with `by = join_by(name)`
Joining with `by = join_by(name)`
Joining with `by = join_by(name)`
Joining with `by = join_by(name)`
found results columns, replacing these
using 'apeglm' for LFC shrinkage. If used in published research, please cite:
Zhu, A., Ibrahim, J.G., Love, M.I. (2018) Heavy-tailed prior distributions for
sequence count data: removing the noise and preserving large differences.
Bioinformatics. https://doi.org/10.1093/bioinformatics/bty895
using 'apeglm' for LFC shrinkage. If used in published research, please cite:
Zhu, A., Ibrahim, J.G., Love, M.I. (2018) Heavy-tailed prior distributions for
sequence count data: removing the noise and preserving large differences.
Bioinformatics. https://doi.org/10.1093/bioinformatics/bty895


Warning in nbinomGLM(x = x, Y = YNZ, size = size, weights = weightsNZ, offset =
offsetNZ, : the line search routine failed, unable to sufficiently decrease the
function value


using 'apeglm' for LFC shrinkage. If used in published research, please cite:
    Zhu, A., Ibrahim, J.G., Love, M.I. (2018) Heavy-tailed prior distributions for
    sequence count data: removing the noise and preserving large differences.
```

```
    Bioinformatics. https://doi.org/10.1093/bioinformatics/bty895
using 'apeglm' for LFC shrinkage. If used in published research, please cite:
    Zhu, A., Ibrahim, J.G., Love, M.I. (2018) Heavy-tailed prior distributions for
    sequence count data: removing the noise and preserving large differences.
    Bioinformatics. https://doi.org/10.1093/bioinformatics/bty895
using 'apeglm' for LFC shrinkage. If used in published research, please cite:
    Zhu, A., Ibrahim, J.G., Love, M.I. (2018) Heavy-tailed prior distributions for
    sequence count data: removing the noise and preserving large differences.
    Bioinformatics. https://doi.org/10.1093/bioinformatics/bty895
Joining with `by = join_by(rowname)`Joining with `by = join_by(rowname)`Joining with `by = jo
using 'apeglm' for LFC shrinkage. If used in published research, please cite:
    Zhu, A., Ibrahim, J.G., Love, M.I. (2018) Heavy-tailed prior distributions for
    sequence count data: removing the noise and preserving large differences.
    Bioinformatics. https://doi.org/10.1093/bioinformatics/bty895
using 'apeglm' for LFC shrinkage. If used in published research, please cite:
    Zhu, A., Ibrahim, J.G., Love, M.I. (2018) Heavy-tailed prior distributions for
    sequence count data: removing the noise and preserving large differences.
    Bioinformatics. https://doi.org/10.1093/bioinformatics/bty895
using 'apeglm' for LFC shrinkage. If used in published research, please cite:
    Zhu, A., Ibrahim, J.G., Love, M.I. (2018) Heavy-tailed prior distributions for
    sequence count data: removing the noise and preserving large differences.
    Bioinformatics. https://doi.org/10.1093/bioinformatics/bty895
using 'apeglm' for LFC shrinkage. If used in published research, please cite:
    Zhu, A., Ibrahim, J.G., Love, M.I. (2018) Heavy-tailed prior distributions for
    sequence count data: removing the noise and preserving large differences.
    Bioinformatics. https://doi.org/10.1093/bioinformatics/bty895
using 'apeglm' for LFC shrinkage. If used in published research, please cite:
    Zhu, A., Ibrahim, J.G., Love, M.I. (2018) Heavy-tailed prior distributions for
    sequence count data: removing the noise and preserving large differences.
    Bioinformatics. https://doi.org/10.1093/bioinformatics/bty895
Joining with `by = join_by(rowname)`Joining with `by = join_by(rowname)`Joining with `by = jo
```

```
group <- levels(as.factor(dds.sva$group))
no_patient_sva_set <- compare_reciprocal_contrasts(group,folder)
```

```
[1] "Found no regulated genes for  OHT_48h  and  DPN_48h"
```

```
[1] "Found no regulated genes for  OHT_48h  and  Control_48h"
```

```
[1] "Found no regulated genes for  OHT_24h  and  DPN_24h"
```

```
[1] "Found no regulated genes for  OHT_24h  and  Control_24h"

[1] "Found no regulated genes for  DPN_48h  and  Control_48h"

[1] "Found no regulated genes for  DPN_24h  and  Control_24h"
```

```
saveRDS(no_patient_sva_set, "no_patient_sva_set.rds")
```

Plot it:

```
sets <- list("no_patient" = no_patient_set,"no_patient_sva" = no_patient_sva_set)
sets <- lapply(sets,
             function(x) lapply(x,
                                function(y) y %>% dplyr::select(partition)))
sets <- lapply(sets,function(x) x %>% bind_rows(.id = "contrast"))
all.sets <- bind_rows(sets, .id = "group")
totals <- all.sets %>%
  group_by(group,contrast) %>%
  summarise(total.per.contrast = n()) %>%
  ungroup()
```

```
`summarise()` has grouped output by 'group'. You can override using the
`.groups` argument.
```

```
head(totals)
```

```
# A tibble: 6 x 3
  group      contrast                 total.per.contrast
  <chr>      <chr>                                 <int>
1 no_patient Control_48h.Control_24h                   7
2 no_patient DPN_24h.Control_48h                      45
3 no_patient DPN_48h.Control_24h                      10
4 no_patient DPN_48h.DPN_24h                          30
5 no_patient OHT_24h.Control_48h                      43
6 no_patient OHT_24h.DPN_48h                          36
```

```
plottable <- all.sets %>%
  group_by(group,contrast) %>%
  count(partition) %>%
  pivot_wider(values_from = n, names_from = partition, values_fill = 0) %>%
  pivot_longer(c(concordant,group1ref,group2ref),names_to = "partition",
               values_to = "n") %>%
  left_join(totals) %>%
  filter(partition == "concordant") %>%
  dplyr::select(-partition) %>%
  rowwise() %>%
  mutate(discordance = 100 - (n/total.per.contrast*100)) %>%
  ungroup() %>%
  mutate(across(c(group, contrast), as_factor))
```

```
Joining with `by = join_by(group, contrast)`
```

```
#for ease of visualisation and understanding, we remove the contrasts with < 4
#total DEGs
plottable <- plottable %>%
  filter(total.per.contrast > 3)
head(plottable)
```

```
# A tibble: 6 x 5
  group      contrast                      n total.per.contrast discordance
  <fct>      <fct>                     <int>              <int>       <dbl>
1 no_patient Control_48h.Control_24h       5                  7        28.6
2 no_patient DPN_24h.Control_48h          24                 45        46.7
3 no_patient DPN_48h.Control_24h           3                 10        70
4 no_patient DPN_48h.DPN_24h              12                 30        60
5 no_patient OHT_24h.Control_48h          22                 43        48.8
6 no_patient OHT_24h.DPN_48h              18                 36        50
```

```
colours <- c(no_patient_sva = '#EE7733',  no_patient = '#BBBBBB')
discord <- ggplot(plottable, aes(x=group, y=discordance, fill = group)) +
  geom_violin(scale = "count") +
  geom_point(size = 2, show.legend = F) +
  scale_fill_manual(values = colours) +
  geom_line(aes(group = contrast)) +
  labs(y = "% discordance\nin total DEGs per contrast") +
  theme_classic(base_size = 20) +
```

```r
  theme(axis.ticks.x = element_blank(),
        axis.title.x = element_blank(),
        axis.text.x = element_blank(),
        legend.title = element_blank()) +
  guides(color = "none")
summed.concordant <- plottable %>%
  summarise(total = sum(n), .by = c(group))
summed.concordant
```

```
# A tibble: 2 x 2
  group          total
  <fct>          <int>
1 no_patient        99
2 no_patient_sva   556
```

```r
summed <- ggplot(summed.concordant,aes(y = total, x = group, fill = group)) +
  scale_fill_manual(values = colours) +
  geom_col() +
  expand_limits(y = 2250) +
  geom_text(aes(label=total), vjust=-0.5, size = 5) +
  labs(y = "total\nconcordant\nDEGs") +
  theme_classic(base_size = 20) +
  theme(legend.position = "none",
        axis.ticks.x = element_blank(),
        axis.title.x = element_blank(),
        axis.text.x = element_blank(),
        strip.background = element_blank(),
        strip.text.x = element_blank())
#for change in concordant DEGs per contrast
changes <- plottable %>%
  dplyr::select(contrast,n,group) %>%
  pivot_wider(names_from = group,values_from = n) %>% #, values_fill = 0) %>%
  rowwise() %>%
  mutate(change = (no_patient_sva-no_patient)/no_patient_sva*100,
         direction = if_else(change > 0, "UP","DOWN")) %>%
  dplyr::select(-c(no_patient,no_patient_sva)) %>%
  mutate(cleanup = "no_patient_sva")
placeholder <- changes %>% dplyr::select(contrast) %>%
  mutate(cleanup = "no_patient")
changes <- changes %>% full_join(placeholder)
```

```
Joining with `by = join_by(contrast, cleanup)`
```

```r
diff <- ggplot(changes,aes(y = change,
                           x = factor(cleanup,
                                      levels = c("no_patient","no_patient_sva")),
                           shape = direction, fill = cleanup)) +
  geom_point(size = 3, stroke = 0.8) +
  scale_shape_manual(values = c("UP" = 24,"DOWN" = 25)) +
  scale_fill_manual(values = colours) +
  scale_x_discrete("cleanup", drop=FALSE) +
  theme_classic(base_size = 20) +
  theme(legend.position = "none",
        axis.ticks.x = element_blank(),
        axis.title.x = element_blank(),
        axis.text.x = element_blank()) +
  ylab("% change in\nconcordant DEGs after\nclean-up per contrast") +
  geom_hline(yintercept = 0, linetype = "dashed") +
  geom_line(aes(group = contrast))

figure <- ggarrange(summed,diff,discord,
                    ncol = 1, nrow = 3, heights = c(1,1.5,2), align = "v")
```

```
Warning: Removed 9 rows containing missing values or values outside the scale range
(`geom_point()`).
```

```
Warning: Removed 9 rows containing missing values or values outside the scale range
(`geom_line()`).
```

```
`geom_line()`: Each group consists of only one observation.
i Do you need to adjust the group aesthetic?
```

```r
figure
```

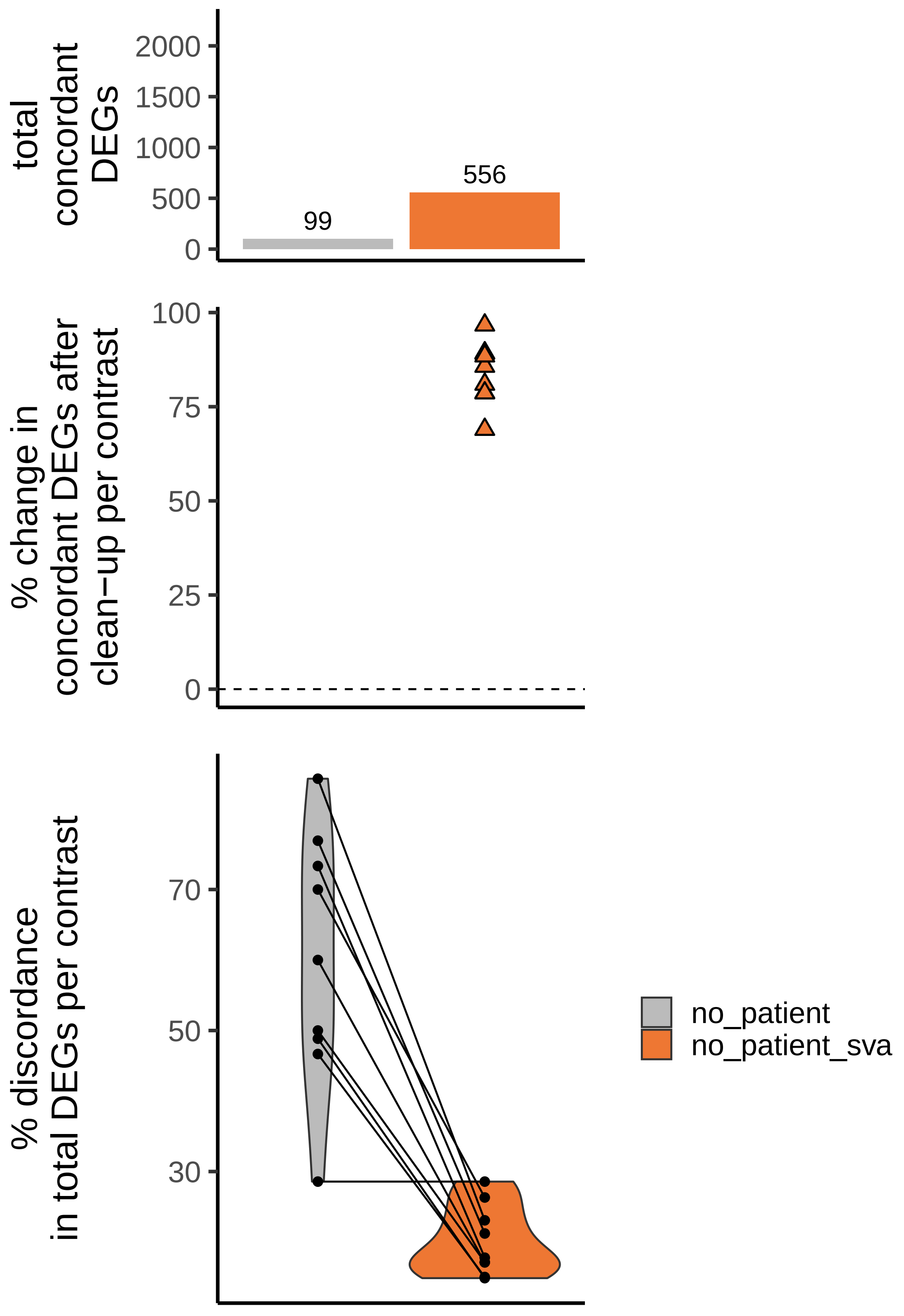
total concordant DEGs
2000
1500
1000
500
0
99
556
% change in concordant DEGs after clean−up per contrast
100
75
50
25
0
% discordance in total DEGs per contrast
70
50
30
no_patient
no_patient_sva

It certainly appears that our identified surrogate variable SV1 is correlated with 'patient', and the results show much less discordance that the 'DESeq_no_patient' results. However, there is still more discordance and fewer identified DEGS than when we were able to include 'patient' in the design (see results in DESeq_noclean).

## Comparison to the original paper

Since SVA was designed for high throughput experiments (whereas this dataset has a low number of samples), and here may be removing variation of biological interest, we choose prefiltering as the most promising clean-up strategy, with `~patient + group` as the design. The original paper (Haglund et al.(2012)) analysed this data with edgeR and made 4 comparisons, corresponding to our DPN_24h_vs_control_24h, DPN_48h_vs_control_48h, OHT_24h_vs_control_24h, and OHT_48h_vs_control_48h, with an adjusted p-value $<$ 0.05 and without using a log fold change threshold. Our concordant gene sets use a $|LFC| > 1$ threshold, but we also used `print.all = T` in `run_DESeq_all_contrasts()`, so we can also look at the full gene lists for these contrasts to find DEGs using only the adjusted p-value. Like Haglund et al. we saw fewer DEGs in the OHT24h contrast (we saw none), whereas we found 25 DEGs for the OHT48hr contrast, including *JUN* and *VDR* which were mentioned in the paper. Regarding DPN, we also observed more DEGs at 48hr (85 at 48h, 16 at 24h), similar to Haglund et al.'s report. Unfortunately, the Supplementary tables for this paper are no longer accessible so we can't do an in-depth comparison of results.

## Session info and citations

Love, M. I., Anders, S., Kim, V., & Huber, W. (2016). RNA-Seq workflow: gene-level exploratory analysis and differential expression. F1000Research 2016 4:1070, 4, 1070. https://doi.org/10.12688/f1000research.7035.2

Reimand, J., Isserlin, R., Voisin, V., Kucera, M., Tannus-Lopes, C., Rostamianfar, A., Wadi, L., Meyer, M., Wong, J., Xu, C., Merico, D., & Bader, G. D. (2019). Pathway enrichment analysis and visualization of omics data using g:Profiler, GSEA, Cytoscape and EnrichmentMap. Nature Protocols, 14(2), 482–517. https://doi.org/10.1038/s41596-018-0103-9

```
citation("parathyroidSE")
```

```
To cite package 'parathyroidSE' in publications use:

  Haglund F, Ma R, Huss M, Sulaiman L, Lu M, Nilsson IL, Hoog A, Juhlin
  CC, Hartman J, Larsson C. 'Evidence of a Functional Estrogen Receptor
  in Parathyroid Adenomas.' J Clin Endocrinol Metab. jc.2012-2484, Epub
  2012 Sep 28, PMID: 23024189
```

```
A BibTeX entry for LaTeX users is

  @Article{,
    author = {{Haglund} and {F.} and {Ma} and {R.} and {Huss} and {M.} and {Sulaiman} and {L
    title = {Evidence of a functional estrogen receptor in parathyroid adenomas},
    journal = {J. Clin. Endocrinol. Metab.},
    year = {2012},
    volume = {97},
    number = {12},
    pages = {4631--4639},
    month = {Dec},
    url = {http://www.ncbi.nlm.nih.gov/pubmed/23024189},
  }
```

```r
citation("sva")
```

```
To cite package 'sva' in publications use:

  Leek JT, Johnson WE, Parker HS, Fertig EJ, Jaffe AE, Zhang Y, Storey
  JD, Torres LC (2024). _sva: Surrogate Variable Analysis_.
  doi:10.18129/B9.bioc.sva <https://doi.org/10.18129/B9.bioc.sva>, R
  package version 3.52.0, <https://bioconductor.org/packages/sva>.

A BibTeX entry for LaTeX users is

  @Manual{,
    title = {sva: Surrogate Variable Analysis},
    author = {Jeffrey T. Leek and W. Evan Johnson and Hilary S. Parker and Elana J. Fertig a
    year = {2024},
    note = {R package version 3.52.0},
    url = {https://bioconductor.org/packages/sva},
    doi = {10.18129/B9.bioc.sva},
  }

ATTENTION: This citation information has been auto-generated from the
package DESCRIPTION file and may need manual editing, see
'help("citation")'.
```

```r
citation("DESeq2")
```

```
To cite package 'DESeq2' in publications use:
```

```
  Love, M.I., Huber, W., Anders, S. Moderated estimation of fold change
  and dispersion for RNA-seq data with DESeq2 Genome Biology 15(12):550
  (2014)

A BibTeX entry for LaTeX users is

  @Article{,
    title = {Moderated estimation of fold change and dispersion for RNA-seq data with DESeq2
    author = {Michael I. Love and Wolfgang Huber and Simon Anders},
    year = {2014},
    journal = {Genome Biology},
    doi = {10.1186/s13059-014-0550-8},
    volume = {15},
    issue = {12},
    pages = {550},
  }
```

```r
citation("edgeR")
```

```
See Section 1.2 in the User's Guide for more detail about how to cite
the different edgeR pipelines.

  Chen Y, Chen L, Lun ATL, Baldoni PL, Smyth GK (2024). edgeR 4.0:
  powerful differential analysis of sequencing data with expanded
  functionality and improved support for small counts and larger
  datasets. bioRxiv doi: 10.1101/2024.01.21.576131

  Chen Y, Lun ATL, Smyth GK (2016). From reads to genes to pathways:
  differential expression analysis of RNA-Seq experiments using
  Rsubread and the edgeR quasi-likelihood pipeline. F1000Research 5,
  1438

  McCarthy DJ, Chen Y and Smyth GK (2012). Differential expression
  analysis of multifactor RNA-Seq experiments with respect to
  biological variation. Nucleic Acids Research 40(10), 4288-4297

  Robinson MD, McCarthy DJ and Smyth GK (2010). edgeR: a Bioconductor
  package for differential expression analysis of digital gene
  expression data. Bioinformatics 26(1), 139-140

To see these entries in BibTeX format, use 'print(<citation>,
```

```
bibtex=TRUE)', 'toBibtex(.)', or set
'options(citation.bibtex.max=999)'.
```

```r
citation("apeglm")
```

```
To cite package 'apeglm' in publications use:

  Zhu, A., Ibrahim, J.G., Love, M.I. Heavy-tailed prior distributions
  for sequence count data: removing the noise and preserving large
  differences Bioinformatics (2018)

A BibTeX entry for LaTeX users is

  @Article{,
    title = {Heavy-tailed prior distributions for sequence count data: removing the noise and
    author = {Anqi Zhu and Joseph G. Ibrahim and Michael I. Love},
    year = {2018},
    journal = {Bioinformatics},
    doi = {10.1093/bioinformatics/bty895},
  }
```

```r
sessionInfo()
```

```
R version 4.4.2 (2024-10-31)
Platform: x86_64-pc-linux-gnu
Running under: Ubuntu 22.04.5 LTS

Matrix products: default
BLAS:   /usr/lib/x86_64-linux-gnu/blas/libblas.so.3.10.0
LAPACK: /usr/lib/x86_64-linux-gnu/lapack/liblapack.so.3.10.0

locale:
 [1] LC_CTYPE=en_AU.UTF-8       LC_NUMERIC=C
 [3] LC_TIME=en_AU.UTF-8        LC_COLLATE=en_AU.UTF-8
 [5] LC_MONETARY=en_AU.UTF-8    LC_MESSAGES=en_AU.UTF-8
 [7] LC_PAPER=en_AU.UTF-8       LC_NAME=C
 [9] LC_ADDRESS=C               LC_TELEPHONE=C
[11] LC_MEASUREMENT=en_AU.UTF-8 LC_IDENTIFICATION=C

time zone: Australia/Melbourne
tzcode source: system (glibc)
```

```
attached base packages:
[1] stats4    stats     graphics  grDevices utils     datasets  methods  
[8] base     

other attached packages:
 [1] sva_3.52.0                  BiocParallel_1.38.0        
 [3] genefilter_1.86.0           mgcv_1.9-1                 
 [5] nlme_3.1-167                biomaRt_2.60.1             
 [7] ggrepel_0.9.6               parathyroidSE_1.42.0       
 [9] ggpubr_0.6.0                ComplexUpset_1.3.3         
[11] UpSetR_1.4.0                edgeR_4.2.2                
[13] limma_3.60.6                lubridate_1.9.4            
[15] forcats_1.0.0               stringr_1.5.1              
[17] dplyr_1.1.4                 purrr_1.0.2                
[19] readr_2.1.5                 tidyr_1.3.1                
[21] tibble_3.2.1                ggplot2_3.5.1              
[23] tidyverse_2.0.0             DESeq2_1.44.0              
[25] SummarizedExperiment_1.34.0 Biobase_2.64.0             
[27] MatrixGenerics_1.16.0       matrixStats_1.5.0          
[29] GenomicRanges_1.56.2        GenomeInfoDb_1.40.1        
[31] IRanges_2.38.1              S4Vectors_0.42.1           
[33] BiocGenerics_0.50.0         mirrorCheck_0.0.1.0        

loaded via a namespace (and not attached):
  [1] RColorBrewer_1.1-3      rstudioapi_0.17.1       jsonlite_1.8.9         
  [4] magrittr_2.0.3          farver_2.1.2            rmarkdown_2.29         
  [7] zlibbioc_1.50.0         vctrs_0.6.5             memoise_2.0.1          
 [10] rstatix_0.7.2           htmltools_0.5.8.1       S4Arrays_1.4.1         
 [13] progress_1.2.3          lambda.r_1.2.4          curl_6.2.0             
 [16] broom_1.0.7             SparseArray_1.4.8       Formula_1.2-5          
 [19] plyr_1.8.9              httr2_1.1.0             futile.options_1.0.1   
 [22] cachem_1.1.0            lifecycle_1.0.4         pkgconfig_2.0.3        
 [25] Matrix_1.7-2            R6_2.6.1                fastmap_1.2.0          
 [28] GenomeInfoDbData_1.2.12 digest_0.6.37           numDeriv_2016.8-1.1    
 [31] colorspace_2.1-1        patchwork_1.3.0         AnnotationDbi_1.66.0   
 [34] RSQLite_2.3.9           filelock_1.0.3          labeling_0.4.3         
 [37] timechange_0.3.0        httr_1.4.7              abind_1.4-8            
 [40] compiler_4.4.2          bit64_4.6.0-1           withr_3.0.2            
 [43] backports_1.5.0         carData_3.0-5           DBI_1.2.3              
 [46] ggsignif_0.6.4          MASS_7.3-64             rappdirs_0.3.3         
 [49] DelayedArray_0.30.1     tools_4.4.2             VennDiagram_1.7.3      
 [52] glue_1.8.0              grid_4.4.2              generics_0.1.3         
```

```
 [55] gtable_0.3.6            tzdb_0.4.0              hms_1.1.3
 [58] utf8_1.2.4              xml2_1.3.6              car_3.1-3
 [61] XVector_0.44.0          pillar_1.10.1           emdbook_1.3.13
 [64] splines_4.4.2           BiocFileCache_2.12.0    lattice_0.22-5
 [67] survival_3.8-3          bit_4.5.0.1             annotate_1.82.0
 [70] tidyselect_1.2.1        locfit_1.5-9.11         Biostrings_2.72.1
 [73] knitr_1.49              gridExtra_2.3           futile.logger_1.4.3
 [76] xfun_0.50               statmod_1.5.0           pheatmap_1.0.12
 [79] stringi_1.8.4           UCSC.utils_1.0.0        yaml_2.3.10
 [82] evaluate_1.0.3          codetools_0.2-19        bbmle_1.0.25.1
 [85] cli_3.6.4               xtable_1.8-4            munsell_0.5.1
 [88] Rcpp_1.0.14             dbplyr_2.5.0            coda_0.19-4.1
 [91] png_0.1-8               bdsmatrix_1.3-7         XML_3.99-0.18
 [94] parallel_4.4.2          blob_1.2.4              prettyunits_1.2.0
 [97] mvtnorm_1.3-3           apeglm_1.26.1           scales_1.3.0
[100] crayon_1.5.3            rlang_1.1.5             cowplot_1.1.3
[103] formatR_1.14            KEGGREST_1.44.1
```
